# Toward high-resolution modeling of small molecule–ion channel interactions

**DOI:** 10.3389/fphar.2024.1411428

**Published:** 2024-06-11

**Authors:** Brandon J. Harris, Phuong T. Nguyen, Guangfeng Zhou, Heike Wulff, Frank DiMaio, Vladimir Yarov-Yarovoy

**Affiliations:** ^1^ Department of Physiology and Membrane Biology, University of California, Davis, Davis, CA, United States; ^2^ Biophysics Graduate Group, University of California, Davis, Davis, CA, United States; ^3^ Department of Biochemistry, University of Washington, Seattle, WA, United States; ^4^ Institute for Protein Design, University of Washington, Seattle, WA, United States; ^5^ Department of Pharmacology, School of Medicine, University of California, Davis, Davis, CA, United States; ^6^ Department of Anesthesiology and Pain Medicine, University of California, Davis, Davis, CA, United States

**Keywords:** Rosetta, ligand docking, ion channel, computational modeling, ligand–protein interactions, computer-aided drug discovery

## Abstract

Ion channels are critical drug targets for a range of pathologies, such as epilepsy, pain, itch, autoimmunity, and cardiac arrhythmias. To develop effective and safe therapeutics, it is necessary to design small molecules with high potency and selectivity for specific ion channel subtypes. There has been increasing implementation of structure-guided drug design for the development of small molecules targeting ion channels. We evaluated the performance of two RosettaLigand docking methods, RosettaLigand and GALigandDock, on the structures of known ligand–cation channel complexes. Ligands were docked to voltage-gated sodium (Na_V_), voltage-gated calcium (Ca_V_), and transient receptor potential vanilloid (TRPV) channel families. For each test case, RosettaLigand and GALigandDock methods frequently sampled a ligand-binding pose within a root mean square deviation (RMSD) of 1–2 Å relative to the experimental ligand coordinates. However, RosettaLigand and GALigandDock scoring functions cannot consistently identify experimental ligand coordinates as top-scoring models. Our study reveals that the proper scoring criteria for RosettaLigand and GALigandDock modeling of ligand–ion channel complexes should be assessed on a case-by-case basis using sufficient ligand and receptor interface sampling, knowledge about state-specific interactions of the ion channel, and inherent receptor site flexibility that could influence ligand binding.

## Introduction

Voltage-gated cation channel families consist of pore-forming transmembrane proteins that selectively conduct ions across lipid bilayers and mediate physiological processes such as signal transduction, gene expression, synaptic transmission, and the activation and proliferation of cells in the immune system (Catterall, 1995; Hille, 2001; Catterall, 2011; Feske et al., 2015; Nanou and Catterall, 2018). Cation channels function in a finely regulated manner across spatial and temporal domains to complete these cellular functions. Current drug discovery efforts aim to modulate channel activity by targeting specific channel domains. For instance, the therapeutically relevant structural domains of a voltage-gated sodium channel are the selectivity filter (otherwise known as the outer-pore vestibule), the central pore cavity (otherwise known as the inner-pore vestibule), and the voltage-sensing domain (VSD) (Nguyen and Yarov-Yarovoy, 2022). Considerable academic and industrial efforts have been taken to identify therapeutically relevant small molecules that selectively target ion channels (Bagal et al., 2013; Zhang et al., 2020). However, developing effective and safe therapeutics targeting ion channels has been challenging (Wulff et al., 2019).

To address these challenges, drug discovery pipelines are trending toward the incorporation of virtual drug screening and computer-aided drug design processes for their ability to minimize drug development time and cost (Maia et al., 2020). Among these processes, molecular docking has demonstrated its usefulness in structure-based drug discovery. Molecular docking involves predicting the conformations and orientation of the small molecule with respect to the protein (known as the pose) and scoring the poses to rank the likely protein–ligand interaction (Meng et al., 2011).

Among the numerous molecular docking software packages, Rosetta is a protein modeling software application and design suite with two established small molecule docking methods: RosettaLigand (Meiler and Baker, 2006; Davis and Baker, 2009; Smith and Meiler, 2020) and GALigandDock (Park et al., 2021). RosettaLigand uses a Monte Carlo minimization procedure using the Rosetta energy function (Alford et al., 2017) to dock a pre-generated set of ligand conformers while allowing sidechain flexibility within a protein-receptor site. GALigandDock utilizes a different approach with two distinct features. First, the scoring function, RosetteGenFF, is a new generalized energy function tailored for small molecules. RosettaGenFF was trained from the Cambridge Structural Database (Groom et al., 2016), which, at the time, contained 1,386 small molecule crystal lattice arrangements, to create a balanced force field that discriminates true-lattice packing arrangements of the ligand from decoy (alternative lattice packing and conformational) arrangements. During docking, an orientation-dependent water-bridging energy term is incorporated within RosettaGenFF to further discriminate the protein–ligand orientation (Pavlovicz et al., 2020). Second, GALigandDock samples conformational space using a genetic algorithm. The ligand rigid-body degrees of freedom and rotatable torsions are encoded as “genes” to generate new ligand inputs for successive docking iterations. This allows the efficient sampling of the protein–ligand energetic landscape when paired with the RosettaGenFF score function, canonical Monte Carlo optimization, and quasi-Newtonian minimization procedures within the Rosetta framework (Park et al., 2021).

Although Rosetta protein–ligand docking methods perform well with soluble protein–ligand benchmarks, the application of these methods to membrane-embedded ion channels has not been explored. Since there is a need to better assess and screen small molecules targeting different ion channel domains, we selected a diverse set of 11 identified cation channel–ligand structures for evaluation. From this set of high-resolution ion channel–small molecule complexes, we assessed the accuracy of the RosettaLigand and GALigandDock methods in sampling ligand poses near the experimental ligand coordinates and predicting the closest matching pose by energy ranking.

Our case studies include four voltage-gated sodium (Na_V_) channel structures, five voltage-gated calcium (Ca_V_) channel structures, and one transient receptor potential vanilloid (TRPV) channel structure. The ion channel–ligand-binding sites include the voltage-sensing domain, selectivity filter, and central pore cavity. Our results demonstrate that RosettaLigand and GALigandDock methods can frequently sample ligand-binding poses within a root mean square deviation (RMSD) of 1–2 Å from the experimental ligand coordinates. However, the ability to identify a pose near the experimental ligand coordinates with energy ranking remains a challenge. When considering factors like the targeted ion channel domain, the ligand library features, and the sampling of ligand and receptor site conformations, our work demonstrates that high-resolution structures paired with RosettaLigand or GALigandDock can support drug discovery pipelines on a case-by-case basis.

## Materials and methods

### Ligand generation

Ligands were extracted as Structure Data Files (.sdf) from PubChem (Kim et al., 2023). Using Avogadro software (Hanwell et al., 2012), each ligand structure underwent bond correction, protonation at pH 7.4, and energy minimization using the Merck molecular force field (Halgren, 1996a; b; c; Halgren and Nachbar, 1996; Halgren, 1999a; b). The resulting models were saved as Tripos Mol2 (.mol2) files. The protonation and bond order of saxitoxin (STX) and tetrodotoxin (TTX) were matched to those in experimentally reported work (Hinman and Du Bois, 2003; Thomas-Tran and Du Bois, 2016). Both experimentally resolved structures of verapamil docked to rabbit Ca_V_1.1 were tested (Zhao et al., 2019b).

Next, using the Antechamber protocol of AmberTools, the partial atomic charge, atom, and bond-type assignments for each ligand were AM1-BCC corrected (Case et al., 2021; Salomon-Ferrer et al., 2012; Supplementary Appendix S1). The AM1-BCC method is commonly used in Rosetta-based ligand docking protocols (Smith and Meiler, 2020; Park et al., 2021) and has demonstrated a similar performance correlation with other RosettaLigand input preparation protocols (Smith and Meiler, 2020).

The AM1-BCC-corrected ligands were used in subsequent steps specific to each method. For RosettaLigand, an in-house script (Supplementary Appendix S2) using the OpenEye Omega toolkit (Hawkins et al., 2010) was used to generate the conformer library, followed by using Rosetta to generate the associated ligand parameter files. For GALigandDock, the input conformer was generated using the RosettaGenFF crystal structure prediction protocol (Park et al., 2021; Supplementary Appendix S3), taking the lowest-energy packing arrangement as the input.

### Ion channel preparation

Ion channel structures were downloaded from the Protein Data Bank (PDB) (Berman et al., 2000). Prior to RosettaLigand docking, the structures were relaxed with backbone constraints using the RosettaRelax protocol (Nivón et al., 2013). This protocol allows the repacking of protein sidechains and minimization of the structure into the Rosetta score function for comparison between poses. The lowest-energy pose from 100 relaxed poses was used for docking.

### RosettaLigand docking

RosettaLigand docking was performed using previously described RosettaScript protocols (Davis and Baker, 2009; DeLuca et al., 2015; Supplementary Appendices S4–S7). Briefly, all ligands were placed into their respective ion channels by superimposing the initial ligand poses onto the experimental ligand coordinates.

RosettaLigand uses grid-based sampling to score the ion channel–ligand interface, with a low-resolution and high-resolution sampling phase. For an unbiased sampling of the local space, an initial transformation during the low-resolution sampling phase is performed on the initial ligand position using the Rosetta Transform mover; the low-resolution Transform mover is a grid-based Monte Carlo simulation, where the ligand is translated up to 0.2 Å and rotated up to 20° per iteration for a total of 500 iterations. A box size of 7–8 Å restrains the ligand center, while all ligand atoms are constrained to a grid with a user-defined width to prevent ligand scoring outside the target site. This scoring grid width was calculated uniquely for each ligand to ensure all ligand conformers would not automatically fail the Transform step. As used previously, the scoring grid width was calculated as the maximum conformer atom–atom distance plus twice the box size value used in the Transform mover (Moretti et al., 2016; Supplementary Appendix S4). The pose with the lowest score was then used as the starting pose for high-resolution docking. For high-resolution docking using the Rosetta HighResDocker mover, six docking cycles of rotamer sampling were performed, with the sidechains repacked every third iteration. Lastly, the ion channel–ligand complex is minimized using the Rosetta FinalMinimizer mover, and the interface scores are reported using the InterfaceScoreCalculator.

For all RosettaLigand docking runs, the ligand_soft_rep and hard_rep scoring functions were reweighted based on previous work assessing Rosetta score functions with the Comparative Assessment of Scoring Functions 2016 (CASF-2016) dataset (Su et al., 2019; Smith and Meiler, 2020). Specifically, reweights of Coulombic electrostatic potential (fa_elec), Lennard-Jones repulsive energy between atoms in the same residue (fa_intra_rep), sidechain-backbone hydrogen bond energy (hbond_bb_sc), sidechain–sidechain hydrogen bond energy (hbond_sc), and Ramachandran preferences (rama) were applied for the soft-repulsive and hard-repulsive docking phases (Supplementary Appendix S6).

For each docking run, either 20,000 poses or 100,000 poses were generated to assess whether there are statistically significant differences in the lowest recorded pose RMSD (RMSD_Min_) to experimental ligand coordinates. In RosettaLigand, a ligand interface is defined either by a representative ligand atom (a “neighbor atom,” defined as the geometric center of mass by default) or all ligand atoms relative to all ion channel C_β_ atoms within a specified radius from the ligand (commonly default to 6 or 7 Å). For RosettaLigand, two mutually exclusive ligand area interface modes are available for scoring the pose interface: the ligand neighbor atom cutoff mode (add_nbr_radius=“True”) and the all-ligand atom cutoff mode (all_atom_mode=“True”). In previous work, both modes were used (Moretti et al., 2016; Smith and Meiler, 2020); thus, both modes were evaluated for any differences in performance. Four individual RosettaLigand docking sets were performed for each PDB structure by combining different pose totals and ligand area interface modes for performance comparison.

### GALigandDock docking

As described in the original study, docking was performed using the GALigandDock mover of RosettaScripts in the dockflex mode (Park et al., 2021; Supplementary Appendices S8–S10). Replicate runs of GALigandDock were performed in parallel for each evaluated structure. Each run consisted of 20 generations with a pool of 100 poses, where each generation updates the pool by total energy. By default, GALigandDock outputs the top 20 structures from the final generation; however, for this study, the entire pool of poses was used. For each docking run, 20,000 poses (1,000 runs) or 100,000 poses (5,000 runs) were output with a padding value of 2, 4, or 7 Å to test for statistical differences in RMSD_Min_. In total, six individual GALigandDock docking sets were performed for each PDB structure by combining different pose totals and padding sizes for performance comparison.

### P_Near_ calculation

P_Near_ is a quantitative metric used to evaluate energy funnel quality from a population of poses, with the lowest-scoring pose as the converged minima or reference state. P_Near_ was calculated as previously defined (Bhardwaj, Mulligan, Bahl, et al., 2016) and applied as previously described for ligand docking (Smith and Meiler, 2020), with the reference state being the experimental ligand coordinates. P_Near_ ranges from 0 (the protein will not converge to the reference state) to 1 (the protein will always converge to the reference state).

$$P_{Near}=\frac{\sum_{i=1}^{N}e^{-\frac{{rmsd}_{i}^{2}}{\lambda^{2}}}e^{-\frac{E_{i}}{k_{B}T}}}{\sum_{j=1}^{N}e^{-\frac{E_{j}}{k_{B}T}}}\;.$$

The numerator is the Boltzmann probability of an individual pose being near the reference state, governed by the “likeness” parameter (*λ*), and the thermal energy, the product of the Boltzmann constant, and absolute temperature (*k*_*B*_ *T*). The denominator is the partition function of a canonical ensemble. For small molecule docking, the RMSD of the pose ligand coordinates relative to the experimental ligand coordinates when the protein pose is superimposed on the entire protein structure, or the receptor site is used. The energy scoring metric (*E*_*i*_) is the interface energy: the energy is solely composed of protein–ligand interactions.

A previous Rosetta small molecule docking study (Park et al., 2021) categorized reference-like poses at an RMSD of 1 or 2 Å without calculating P_Near_, while another study evaluating RosettaLigand performance calculated P_Near_ using *λ* = 1.5 Å and *k*_*B*_ *T* = 0.62 (Meiler and Smith, 2006). Therefore, we calculated P_Near_ with reference-like poses defined by *λ* = 2.0 Å and *k*_*B*_ *T* = 0.62 Rosetta energy units (REU); however, we calculated P_Near_ using all previously reported values for the parameters *λ* and *k*_*B*_ *T* (Supplementary Tables S4.1–S4.10). A specific P_Near_ cutoff indicative for drug discovery pipelines has not been established; hence, we refer to P_Near_ ≥ 0.5 as a “first-pass” cutoff for this study when evaluating energy funnel convergence to the experimental ligand coordinates.

### Statistical tests

Tests for normality, heteroscedasticity, and Pearson’s correlation between covariates were performed in Python using NumPy (Harris et al., 2020), SciPy (Virtanen et al., 2020), and Pingouin (Vallat, 2018) with a significance level (α) of 0.05. Population data consisted of RMSD_Min_ for each docking set. Not all RMSD_Min_ data for each docking set fit a normal distribution. Since RMSD_Min_ data should skew toward 0 Å, a lognormal base-10 transformation was applied to all RMSD_Min_ population data when comparing across docking sets. Shapiro–Wilk tests for normality and Levene heteroscedasticity tests were performed to ensure the transformed data were normal and of equal variance, respectively.

Covariates for both methods were the number of rotatable bonds of the ligand, the number of ligand heavy atoms, and the resolution of the structure. Statistical tests for RosettaLigand also included the number of conformers generated, transformed with a lognormal base-10, as a covariate. The ligand molecular weight was initially included as a covariate but was discarded after identifying strong Pearson’s correlation with the number of ligand heavy atoms (Supplementary Table S3; Supplementary Figure S1).

To assess RMSD_Min_ across all docking sets for a given method, a repeated measures factorial analysis of variance (ANOVA) with covariates was performed using IBM SPSS (version 29). For RosettaLigand, the two factors were sample size and ligand area interface mode, while for GALigandDock, the two factors were sample size and padding value. Mauchly’s test for sphericity was performed for levels of padding (*p* = 0.63).

The coordinates of top Rosetta score models of the ion channel–ligand complexes are available in the Dryad database (https://doi.org/10.5061/dryad.m63xsj49v).

## Results

**FIGURE 1:**
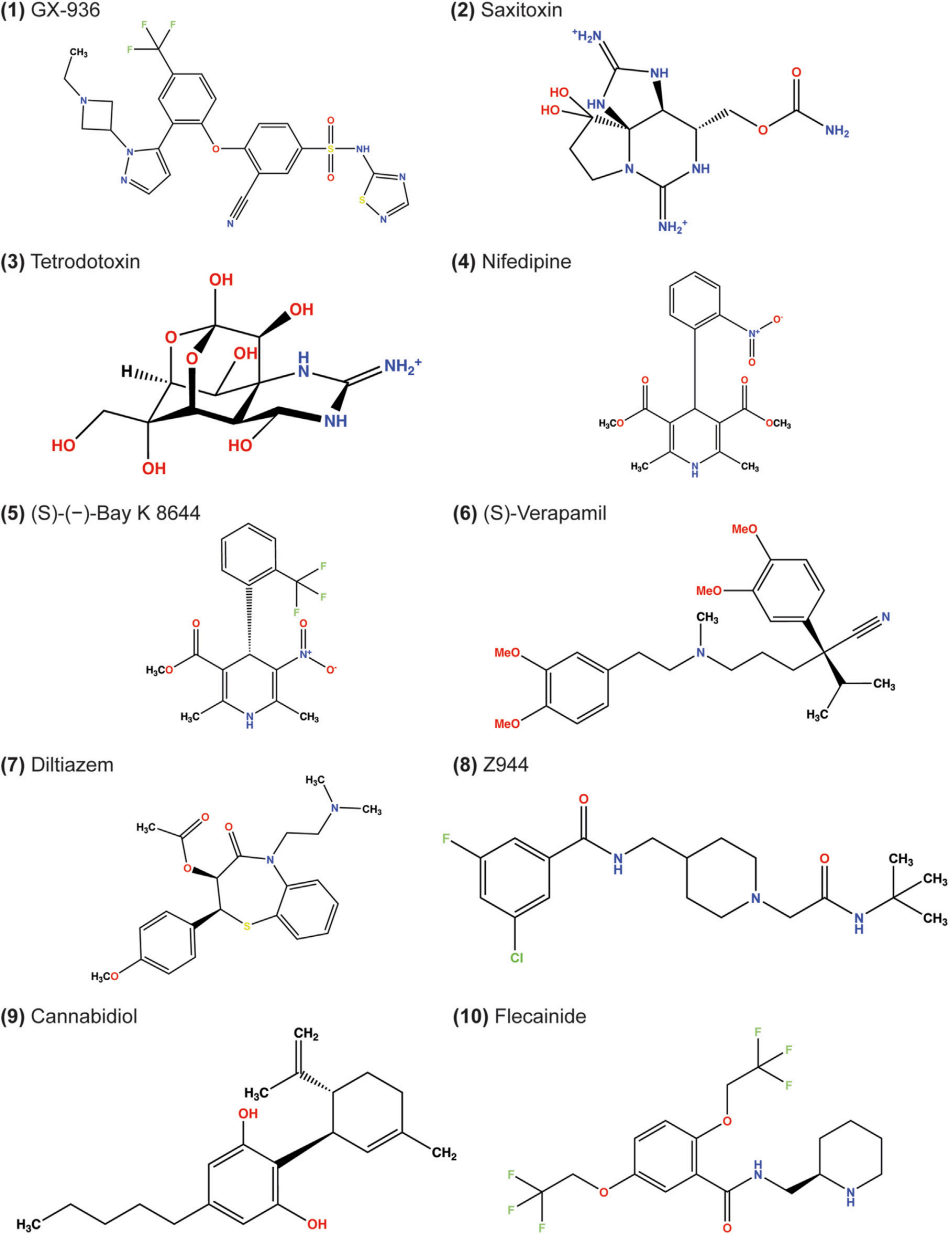
Chemical structures of ligands docked in this study. The depicted stereochemistry reflects that resolved from structures. The protonation and bond order of saxitoxin and tetrodotoxin from prior reported work were used (Hinman and Du Bois, 2003; Thomas-Tran and Du Bois, 2016).

In this study, we evaluated ligands that are suggested to modulate ion channel activity for a given clinical effect, such as antiarrhythmic, anticonvulsant, and antihypertensive (Table 1). Our study also includes canonical ion channel blockers, such as tetrodotoxin and saxitoxin, small molecules whose structures have been resolved for drug discovery campaigns, such as GX-936, Z944, and (S)-(−)-Bay K 8644, and drugs currently approved for therapy, such as nifedipine, diltiazem, and flecainide (Figure 1).

**TABLE 1:** Summary of ligand–ion channel structures docked, domain, and covariates included in the study.

PDB ID	Description	Ion channel modulation/therapeutic class	Domain/region	Total ligand rotatable bonds	Total ligand heavy atoms	Ligand molecular weight (Da)	Structure resolution (Å)	Total RosettaLigand conformers
5EK0	Human Na_V_1.7-VSD4-Na_V_Ab/GX-936	Inhibitor/NA	Voltage-sensing domain	8	39	575.6	3.53	2,179
6J8G	Human Na_V_1.7/saxitoxin	Blocker/NA	Selectivity filter	3	21	299.3	3.20	28
6J8I	Human Na_V_1.7/tetrodotoxin	Blocker/NA	Selectivity filter	1	22	319.3	3.20	2
6JP5	Rabbit Ca_V_1.1/nifedipine	Blocker/vasodilator, antihypertensive, and antianginal	CPC^a^	5	25	346.3	2.90	22
6JP8	Rabbit Ca_V_1.1/(S)-(−)-Bay K 8644	Activator/NA	CPC	3	25	356.3	2.70	3
6JPA	Rabbit Ca_V_1.1/(S)-verapamil	Blocker/antiarrhythmic vasodilator	CPC	13	33	454.6	2.60	785
6JPB	Rabbit Ca_V_1.1/diltiazem	Blocker/antihypertensive vasodilator	CPC	7	29	414.5	2.90	45
6KZP	Human Ca_V_3.1/Z944	Blocker/NA	CPC	6	26	383.9	3.10	1,992
6U88	Rat TRPV2/cannabidiol	Activator/anticonvulsants	CPC	6	23	314.5	3.20	71
6UZ0	Rat Na_V_1.5/flecainide	Blocker/antiarrhythmic class 1c	CPC	7	28	414.3	3.24	1,169

^a^ CPC, central pore cavity.

### Performance criteria for RosettaLigand docking

A previous study has emphasized that two criteria must be satisfied for the accurate modeling of protein–ligand interactions (Kaufmann and Meiler, 2012). First, the method must produce poses resembling the reference structure through sufficient sampling. For small molecule docking, a ligand pose that meets this criterion has an RMSD below a context-dependent predetermined value. Previously reported RMSD ranges when assessing whether a pose resembles the reference structure are 1.0 Å or 2.0 Å, with 2.0 Å being a common cutoff for drug discovery pipelines (Maia et al., 2020; Park et al., 2021). When calculating the RMSD, the protein pose is superimposed on the entire protein structure or the receptor site, and then the ligand coordinates of the pose are evaluated relative to the experimental ligand coordinates.

Second, the pose population must produce an energy funnel converging to the lowest-energy pose, which would closely match the experimental ligand coordinates (Meiler and Baker, 2006). Rather than the use the lowest-energy pose, we use empirical structural evidence in this study to define the reference pose as the experimental ligand coordinates. An energy funnel is qualitatively evaluated using the interface energy between the ligand and protein with respect to the ligand RMSD (Meiler and Baker, 2006; Smith and Meiler, 2020). A quantitative metric of funnel convergence, P_Near_, is utilized in Rosetta protein design (Bhardwaj et al., 2016; Mulligan et al., 2021) and has been adopted for small molecule docking (Smith and Meiler, 2020). Due to the large number of poses, funnel quality is often assessed with a population subset consisting of the best-scoring poses (Combs et al., 2011; Lemmon et al., 2012; Shim et al., 2019).

With these criteria in mind, we evaluated the performance of RosettaLigand and GALigandDock for the following ion channel receptor sites: the voltage-sensing domain, the outer pore, and the central pore cavity (Table 1). We evaluated RosettaLigand performance using combinations of the sample size (20,000 poses vs. 100,000 poses) and ligand area interface mode (ligand neighbor atom vs. all ligand atoms). We evaluated GALigandDock performance using combinations of the sample size (20,000 poses vs. 100,000 poses) and padding of the sampling grid (2 Å, 4 Å, and 7 Å). Increasing the padding of the sampling grid enables additional rotamer sampling around the receptor and increases translational sampling of the ligand around the receptor site.

### Covariates potentially influencing the RMSD

We controlled the following covariates that are dependent on the ligand or structure used for docking. We speculated that the number of ligand rotatable bonds and the number of heavy atoms could influence RMSD_Min_ by increasing the amount of internal sampling required during the docking run. We also speculated that a poorer, higher reported structural resolution could result in an increased overall RMSD_Min_. The total number of ligand heavy atoms was also used as a covariate for RMSD_Min_, which is strongly correlated to the molecular weight of each ligand (Supplementary Figure S1). Lastly, for RosettaLigand, the total number of conformers provided as the input could affect RMSD_Min_ since more conformers could inherently require more sampling.

### RosettaLigand and GALigandDock frequently sample experimental ligand coordinates in ion channel–ligand models

This study evaluated docking sets using population data consisting of the lowest RMSD pose (RMSD_Min_) from each channel–ligand test case. We provide four docking sets for RosettaLigand and six docking sets for GALigandDock, using combinations of the sample size with either the RosettaLigand-specific ligand area interface calculation or GALigandDock-specific padding value. For brevity, we discuss the RosettaLigand docking set using a sample size of 100,000 poses and the all-ligand atom interface mode and the GALigandDock docking set using a sample size of 100,000 poses and a padding value of 7 Å. These specific docking sets provide the greatest breadth of receptor sampling and scoring among our docking sets by appropriately encompassing the entirety of each ion channel domain/region tested (Table 2). The results for other docking sets are provided in Supplementary Tables S1.1–S1.2.

**TABLE 2:** Minimum RMSD (Å) pose for each ligand–channel complex that is detailed in the study.

PDB ID	Description	RosettaLigand, all-ligand atom interface cutoff, 100,000 poses	GALigandDock, padding 7 Å, 100,000 poses
5EK0	Human Na_V_1.7-VSD4-Na_V_Ab/GX-936	0.91	0.65
6J8G	Human Na_V_1.7/saxitoxin	0.70	0.76
6J8I	Human Na_V_1.7/tetrodotoxin	0.54	0.81
6JP5	Rabbit Ca_V_1.1/nifedipine	0.77	1.3
6JP8	Rabbit Ca_V_1.1/(S)-(−)-Bay K 8644	0.93	0.97
6JPA-1^a^	Rabbit Ca_V_1.1/(S)-verapamil	1.4	2.0
6JPA-2	Rabbit Ca_V_1.1/(S)-verapamil	2.2	2.1
6JPB	Rabbit Ca_V_1.1/diltiazem	2.5	1.1
6KZP	Human Ca_V_3.1/Z944	0.73	0.84
6U88	Rat TRPV2/cannabidiol	1.0	0.90
6UZ0	Rat Na_V_1.5/flecainide	1.2	0.94
Average		1.2 ± 0.6	1.1 ± 0.5
Median		0.93	0.94

^a^ Verapamil was resolved with two binding orientations to the rabbit Ca_V_1.1 central pore cavity in 6JPA, which are named 6JPA-1 (orientation 1) and 6JPA-2 (orientation 2) in this study.

For RosettaLigand, the average RMSD_Min_ across the docking set was 1.2 ± 0.6 Å (Table 2). When comparing RosettaLigand docking sets and using repeated measures ANOVA with covariates, an interaction from both factors (sample size and ligand area interface mode) did not result in the rejection of the null hypothesis of equivalent means from common logarithm-transformed RMSD_Min_ data (*p* = 0.71). Likewise, individual interactions from sample size (*p* = 0.38) and ligand area interface mode (*p* = 0.72) did not result in the rejection of the null hypothesis of equivalent means. Each RosettaLigand docking set produced comparable RMSD_Min_ averages and standard deviations within a few sub-Angstroms (Supplementary Table S1.1). Therefore, our results suggest that using ligand area interface mode with a total of either 20,000 or 100,000 poses could generate poses near the experimental ligand structural coordinates.

For GALigandDock, the average RMSD_Min_ across the docking set was 1.1 ± 0.5 Å (Table 2), while all docking sets produced similar RMSD_Min_ averages and standard deviations within a few sub-Angstroms (Supplementary Table S1.2). Again, using repeated measures ANOVA with covariates, an interaction from both factors (sample size and padding size) failed to result in a rejection of the null hypothesis of equivalent means (*p* = 0.91). Likewise, individual interactions from the sample size (*p* = 0.59) and padding size (*p* = 0.34) did not result in rejection of the null hypothesis of equivalent means. While there was no statistical advantage to using a specific padding value to achieve a lower average RMSD_Min_, we note that ion channel structures within the central-pore cavity had greater sampling with a padding value of 7 Å since a padding value of 5 Å did not encompass the entire pore. Therefore, the appropriate padding size is context dependent and should be adjusted when using GALigandDock to provide sufficient sampling grid space.

### RosettaLigand and GALigandDock energy funnels for ion channel–ligand docking

We evaluated whether the entire population of the generated poses and the top 10% of the lowest total energy-scoring poses would achieve P_Near_ values indicative of an energy funnel converging onto the experimental ligand coordinates (Supplementary Tables S4.1–S4.10). Since interface energy describes how a ligand interacts with the ion channel, P_Near_ was calculated with the interface energy rather than the total energy. A specific P_Near_ cutoff indicative of drug discovery pipelines has not been established. Hence, we refer to P_Near_ ≥ 0.5 as a “first-pass” cutoff for this study when evaluating energy funnel convergence to the experimental ligand coordinates. For brevity, we discuss P_Near_ with a “likeness” parameter (*λ*) of 2.0 and a thermal energy parameter (k_B_T) of 0.62. Other P_Near_ values matching parameter values reported in other work are provided in Supplementary Material (Bhardwaj et al., 2016; Smith and Meiler, 2020; Supplementary Tables S4.1–S4.10).

The P_Near_ value of the full population and the top 10% of total energy-scoring poses were similar within methods, with the bulk of P_Near_ values being in the thousandths or lower (Table 3). However, RosettaLigand and GALigandDock identified energy funnels for unique cases. For RosettaLigand, the human Ca_V_3.1-Z944 complex (PDB: 6KZP) achieved P_Near_ ≥ 0.5, while for GALigandDock, human Na_V_1.7-VSD4-Na_V_Ab-GX-936 (PDB: 5EK0) achieved P_Near_ ≥ 0.5. For both methods, rabbit Ca_V_1.1-(S)-(−)-Bay K 8644 (PDB: 6JP8) achieved P_Near_ ≥ 0.5.

**TABLE 3:** P_Near_ of RMSD_Min_ and interface energy for each ligand–channel complex that is detailed in the study, calculated with k_B_T = 0.62 and *λ* = 2.0.

		RosettaLigand, all-ligand atom interface cutoff, 100,000 poses		GALigandDock, padding 7 Å, 100,000 poses	
PDB ID	Description	Full population	Top 10% total_score	Full population	Top 10% total_score
5EK0	Human Na_V_1.7-VSD4-Na_V_Ab/GX-936	0.22	0.26	0.83	0.83
6J8G	Human Na_V_1.7/saxitoxin	1.2•10^−2^	9.3•10^−3^	1.6•10^−2^	1.6•10^−2^
6J8I	Human Na_V_1.7/tetrodotoxin	0.12	8.8•10^−2^	0.27	0.27
6JP5	Rabbit Ca_V_1.1/nifedipine	0.21	0.28	0.24	0.29
6JP8	Rabbit Ca_V_1.1/(S)-(−)-Bay K 8644	0.62	0.66	0.60	0.61
6JPA-1	Rabbit Ca_V_1.1/(S)-verapamil	4.7•10^−3^	4.3•10^−3^	2.4•10^−3^	2.8•10^−3^
6JPA-2	Rabbit Ca_V_1.1/(S)-verapamil	5.0•10^−4^	1.2•10^−4^	1.8•10^−5^	1.9•10^−5^
6JPB	Rabbit Ca_V_1.1/diltiazem	6.8•10^−4^	6.4•10^−4^	2.8•10^−6^	7.8•10^−6^
6KZP	Human Ca_V_3.1/Z944	0.50	0.62	1.2•10^−4^	1.1•10^−4^
6U88	Rat TRPV2/cannabidiol	8.3•10^−2^	4.3•10^−2^	5.0•10^−3^	3.8•10^−3^
6UZ0	Rat Na_V_1.5/flecainide	0.14	0.12	5.9•10^−6^	2.9•10^−5^

Following the standard of reporting a percentage of poses (Combs et al., 2011; Lemmon et al., 2012; Shim et al., 2019), specific P_Near_ values reported herein refer to the top 10% of total energy-scoring poses (Table 3).

Notably, there are visual distinctions in interface energy funnel plots between RosettaLigand and GALigandDock (Supplementary Figures S2–S12). Generally, RosettaLigand can sample more poses up to an RMSD of 2 Å than GALigandDock but with a less distinguishable energy funnel since some poses score greater than zero, indicating an unfavorable energy score. Since GALigandDock uses the lowest total energy poses as the input for future conformer generations, poses with interface energy greater than zero are infrequent.

Lastly, when using the lowest-interface energy pose from the full docking population as ranking criteria, RMSD_Min_ increases to a range between 1.2 Å and 10.8 Å (mean 4.5 ± 2.8 Å) with RosettaLigand (Supplementary Table S2.1) and a range between 0.83 Å and 14.5 Å (mean 6.4 ± 5.0 Å) with GALigandDock (Supplementary Table S2.5). This suggests that extracting the lowest energy pose is not a reliable indicator of a reference pose and does not necessarily reflect the binding mode for ion channel–ligand docking.

### Ligand docking into the voltage-sensing domain

The only ion channel–ligand structure evaluated for ligand docking into the VSD was GX-936 in complex with VSD4 of the hNa_V_1.7-Na_V_Ab chimera (PDB: 5EK0). The hNa_V_1.7 channel has been validated as a drug target for pain signaling, and aryl sulfonamides have been reported as selective inhibitors. Specifically, GX-936 exhibits selectivity for hNa_V_1.7 compared to other hNa_V_ isoforms (McCormack et al., 2013; Ahuja et al., 2015; Nguyen and Yarov-Yarovoy, 2022).

**FIGURE 2:**
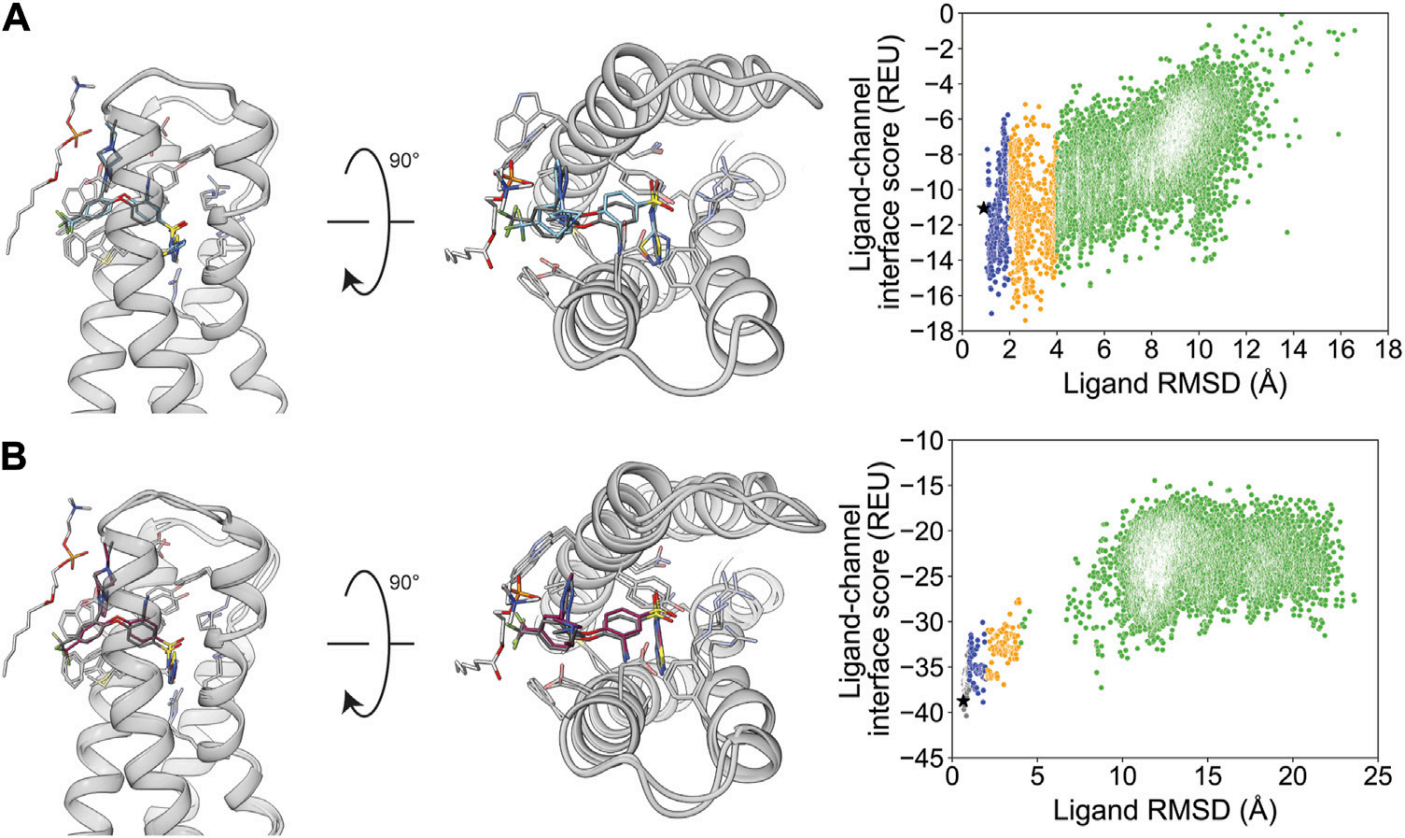
GX-936 docking to hNa_V_1.7-Na_V_Ab voltage-sensing domain 4 (PDB: 5EK0). **(A)** RosettaLigand (RMSD_Min_ = 0.91 Å and P_Near_ = 0.26) and **(B)** GALigandDock (RMSD_Min_ = 0.65 Å and P_Near_ = 0.83). Left: transmembrane view. Middle: extracellular view. Right: ligand RMSD vs. interface energy distribution of the top 10% of total energy poses. Gray dots: <1 Å; blue dots: 1–2 Å; yellow dots: 2–4 Å; and green dots: >4 Å. Carbon atoms of RosettaLigand molecules are shown in cyan, GALigandDock in dark pink, experimental structure in dark gray, and phospholipid head group resolved from the structure in light gray. Non-carbon atoms match the Corey–Pauling–Koltun coloring scheme. Black star indicates the RMSD_Min_ pose from the entire population.

After docking GX-936 to hNa_V_1.7 VSD4, the RMSD_Min_ poses were 0.91 Å using RosettaLigand and 0.65 Å using GALigandDock (Figure 2; Table 2). The largest deviations from the experimental ligand coordinates were observed at the peripheral pyrazole ring and the ethyl azetidine (Figure 2). Notably, GX-936 has eight rotatable bonds, making it the second most flexible ligand used in this study (Table 1; Figure 1). However, the sampled ligand poses were consistently below an RMSD of 2 Å for each docking run (Supplementary Tables S1.1, S1.2). RosettaLigand was unable to identify the RMSD_Min_ pose as the lowest-interaction energy pose (Supplementary Table S2.1), scoring the lowest energy pose 2.7 Å from the experimental ligand coordinates. This pose has the same binding orientation, but the pyrazole ring clashes with the structurally resolved lipid, while the ethyl azetidine within the VSD is orientated up toward the extracellular space rather than downward. Indeed, the lowest 10 interface energy poses all possess these features (Supplementary Figure S13.1). GALigandDock scored the lowest-energy pose 0.83 Å from the experimental ligand coordinates (Supplementary Table S2.5). Compared to RosettaLigand, the lowest 10 interface energy poses converge well with the RMSD_Min_ pose, with only 1 of the 10 poses clashing with the lipid (Supplementary Figure S13.2). Furthermore, GALigandDock discriminated with good confidence an energy funnel (P_Near_ = 0.83; Table 3), whereas RosettaLigand was unable to distinguish a clear energy funnel (P_Near_ = 0.26; Table 3).

### Ligand docking into the outer pore

TTX and STX are both guanidinium-based small molecules derived from puffer fish and shellfish and act as selective blockers of sodium channels by binding to the outer pore (Hille, 2001; Shen et al., 2019). Being potent pore blockers for only some Na_V_ channel isoforms, hNa_V_ channel isoforms are classified in physiology as TTX-resistant (hNa_V_1.5 and hNa_V_1.8–1.9) and TTX-sensitive (hNa_V_1.1–1.4, hNa_V_1.6–1.7) (Stevens et al., 2011). The discovery of TTX and STX binding to the outer pore of Na_V_ channels has spurred the design of similar blockers with greater selectivity for a specific hNa_V_ isoform, usually hNa_V_1.7 for pain therapy (Hagen et al., 2017; Pajouhesh et al., 2020).

**FIGURE 3:**
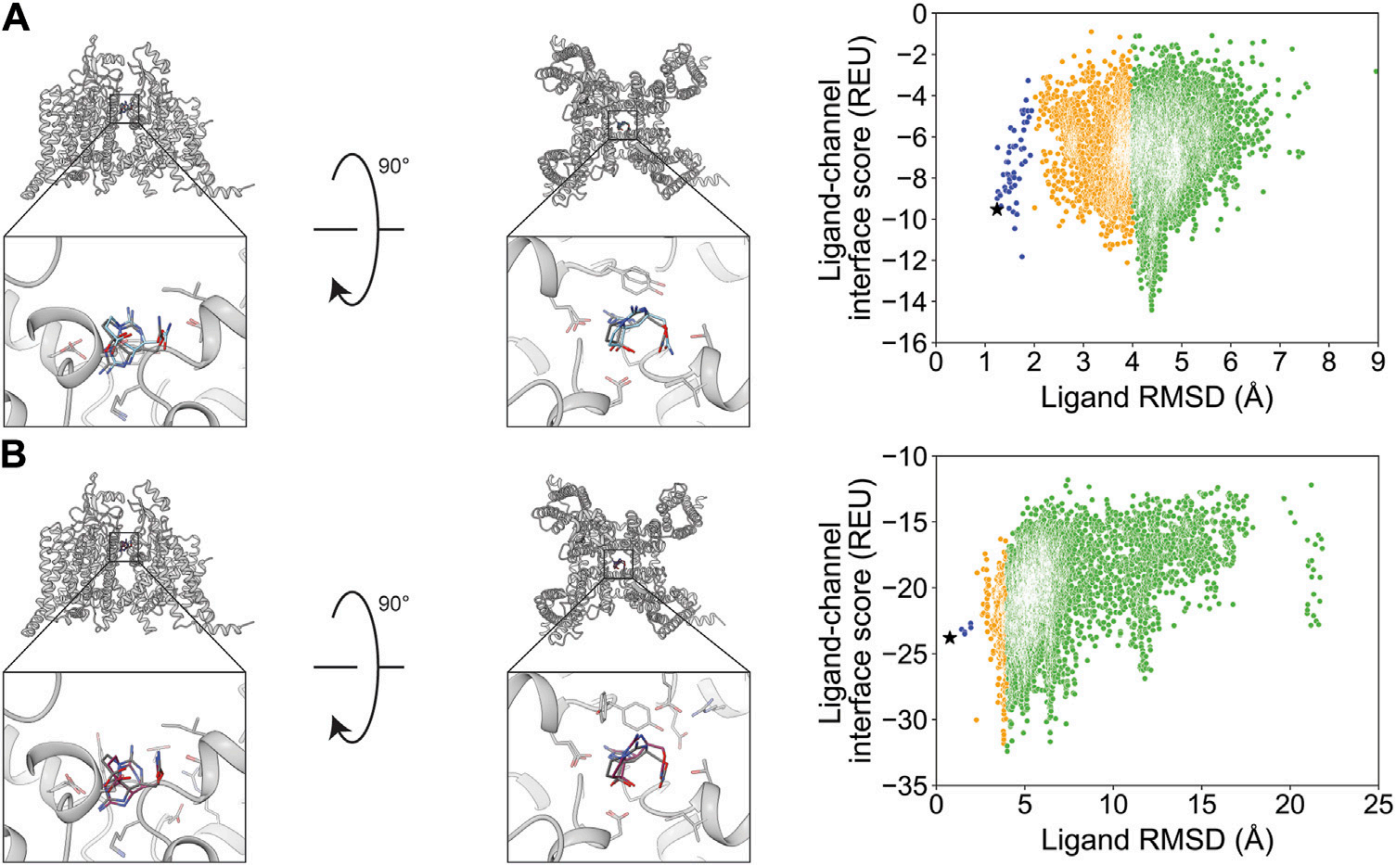
Saxitoxin docking to the hNa_V_1.7 selectivity filter (PDB: 6J8G). **(A)** RosettaLigand (RMSD_Min_ = 0.70 Å and P_Near_ = 9.3•10^−3^) and **(B)** GALigandDock (RMSD_Min_ = 0.76 Å and P_Near_ = 1.6•10^−2^). Left: transmembrane view. Middle: extracellular view. Right: ligand RMSD vs. interface energy distribution of the top 10% of total energy poses. Gray dots: <1 Å; blue dots: 1–2 Å; yellow dots: 2–4 Å; and green dots: >4 Å. Carbon atoms of RosettaLigand molecule are shown in cyan, GALigandDock in dark pink, and experimental structure in dark gray. Non-carbon atoms match the Corey–Pauling–Koltun coloring scheme. Black star indicates the RMSD_Min_ pose from the entire population.

**FIGURE 4:**
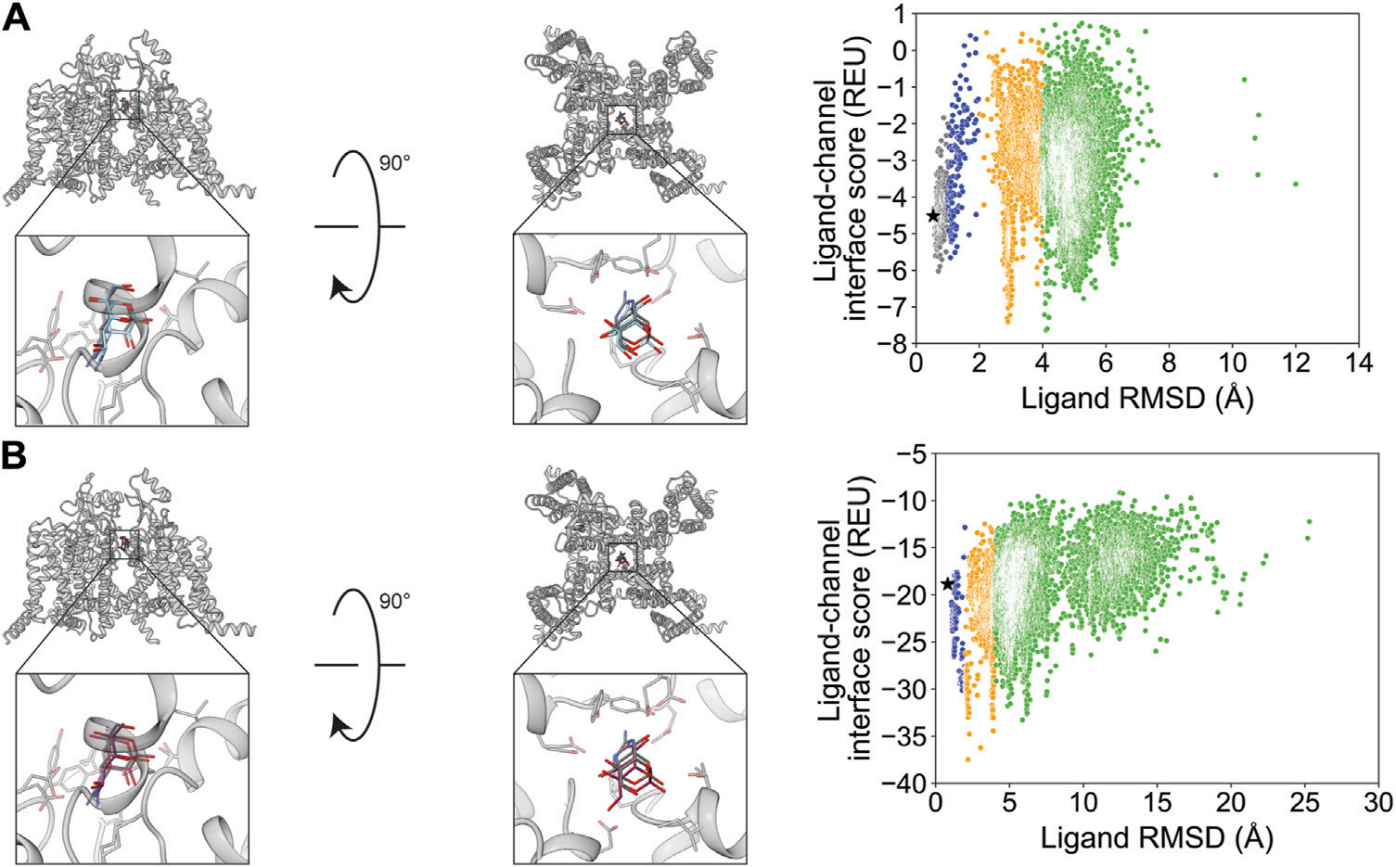
Tetrodotoxin docking to the hNa_V_1.7 selectivity filter (PDB: 6J8I). **(A)** (RMSD_Min_ = 0.54 Å and P_Near_ = 7.8•10^−2^) and **(B)** GALigandDock (RMSD_Min_ = 0.81 Å and P_Near_ = 0.27). Left: transmembrane view. Middle: extracellular view. Right: ligand RMSD vs. interface energy distribution of the top 10% of total energy poses. Gray dots: <1 Å; blue dots: 1–2 Å; yellow dots: 2–4 Å; and green dots: >4 Å. Carbon atoms of RosettaLigand molecules are shown in cyan, GALigandDock in dark pink, and experimental structure in dark gray. Non-carbon atoms match the Corey–Pauling–Koltun coloring scheme. Black star indicates the RMSD_Min_ pose from the entire population.

We chose STX and TTX as test cases to evaluate ligand docking into the outer pore of hNa_V_1.7 (PDB: 6J8G and 6J8I, respectively; Shen et al., 2019). The protonation and bond order of STX and TTX from a previously reported work were used (Thomas-Tran and Du Bois, 2016). Based on their cage-like structures, STX has only three rotatable bonds and TTX has only one rotatable bond, making them the most rigid ligands in this study (Table 1; Figure 1). In both cases, RosettaLigand docking resulted in RMSD_Min_ values of 0.70 Å for STX and 0.54 Å for TTX, while GALigandDock reported RMSD_Min_ values of 0.76 Å for STX and 0.81 Å for TTX (Table 2; Figures 3, 4). The P_Near_ values suggested little-to-no energy funnel convergence with STX (RosettaLigand P_Near_ = 9.3•10^−3^ and GALigandDock P_Near_ = 1.6•10^−2^) and TTX (RosettaLigand P_Near_ = 7.8•10^−2^ and GALigandDock P_Near_ = 0.27) (Table 3). This lack of convergence is due to other energy minima occurring within an RMSD of 3–6 Å (Figures 3, 4).

For STX, the lowest 10 interface energy poses with RosettaLigand converged at an RMSD value of 4.4 Å, with the carbamoyl group positioned deeper into the selectivity filter, while GALigandDock rendered an RMSD value of 3.8–6.4 Å, with the center of STX further away from the selectivity filter in various rotations (Supplementary Figures S14.1, S14.2). For TTX, the lowest 10 interface energy poses with RosettaLigand contained poses between an RMSD of 2.9 and 4.2 Å; 5 of the 10 poses converged to the experimental ligand coordinates, with the guanidinium group pointing away from the selectivity filter, while the other 5 poses converged to the experimental ligand coordinates, with the guanidinium group pointing perpendicular to the selectivity filter path (Supplementary Figure S15.1). With GALigandDock, the poses were between an RMSD of 2.2 and 5.9 Å, with 5 of 10 poses having the TTX guanidinium group pointing toward the pore and 1 set of P1/P2 helices, 3 of 10 poses with the TTX guanidinium group pointing toward the selectivity filter path, and 1 pose with the TTX guanidinium group pointing toward the extracellular space (Supplementary Figure S15.2).

### Ligand docking into the central pore cavity

Seven test cases were used to evaluate ligand docking into the central pore cavity involving the following channels: four cases from rabbit Ca_V_1.1, one case from human Ca_V_3.1, one case from rat Na_V_1.5, and one case from TRPV2. The central pore cavity is a broad target, with small molecules reported to traverse through the gate or fenestration to reach their binding site and modulate channel activity via the pore blockade or allosteric mechanism (Hille, 1977; Hockerman et al., 1997; Jiang et al., 2020). Drugs targeting this region can act as vasodilators (dihydropyridines, benzothiazepines, and phenylalkylamines), antiarrhythmics (benzothiazepines, phenylalkylamines, and flecainide), antiepileptics (Z944 and cannabidiol), or local anesthetics (flecainide) (Zhao et al., 2019a; Zhao et al., 2019b; Pumroy et al., 2019; Jiang et al., 2020).

We docked nifedipine (dihydropyridine channel blocker), (S)-(−)-Bay K 8644 (dihydropyridine channel activator), diltiazem (benzothiazepine channel blocker), and two conformations of verapamil (phenylalkylamine channel blocker) into rabbit Ca_V_1.1. The number of rotatable bonds was 5, 3, 7, and 13, respectively (Table 1; Figure 1).

**FIGURE 5:**
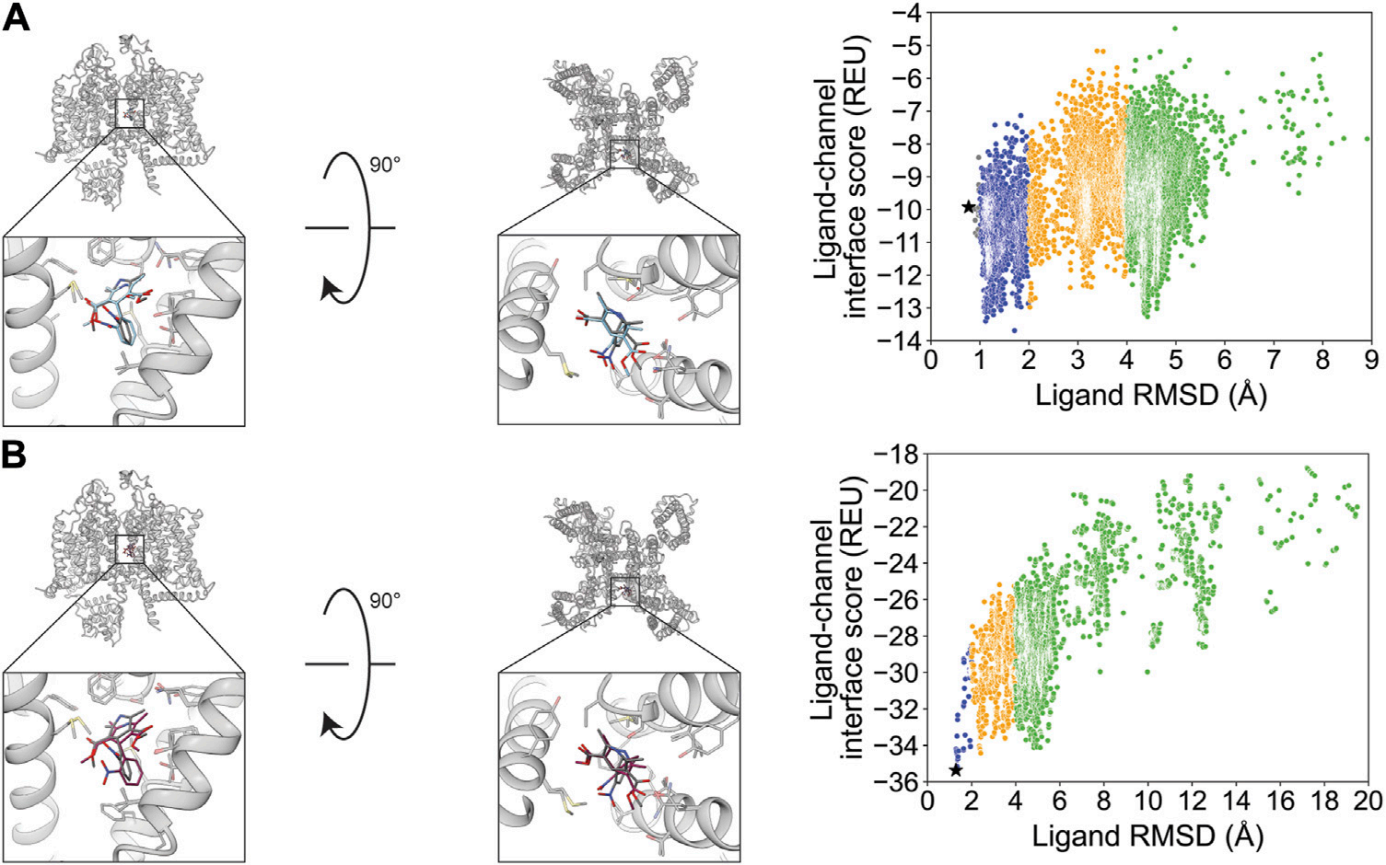
Nifedipine docking to the rabbit Ca_V_1.1 central pore cavity (PDB: 6JP5). **(A)** RosettaLigand (RMSD_Min_ = 0.77 Å and P_Near_ = 0.28) and **(B)** GALigandDock (RMSD_Min_ = 1.3 Å and P_Near_ = 0.29). Left: transmembrane view. Middle: extracellular view. Right: ligand RMSD vs. interface energy distribution of the top 10% of total energy poses. Gray dots: <1 Å; blue dots: 1–2 Å; yellow dots: 2–4 Å; and green dots: >4 Å. Carbon atoms of RosettaLigand molecules are shown in cyan, GALigandDock in dark pink, and experimental structure in dark gray. Non-carbon atoms match the Corey–Pauling–Koltun coloring scheme. Black star indicates the RMSD_Min_ pose from the entire population.

**FIGURE 6:**
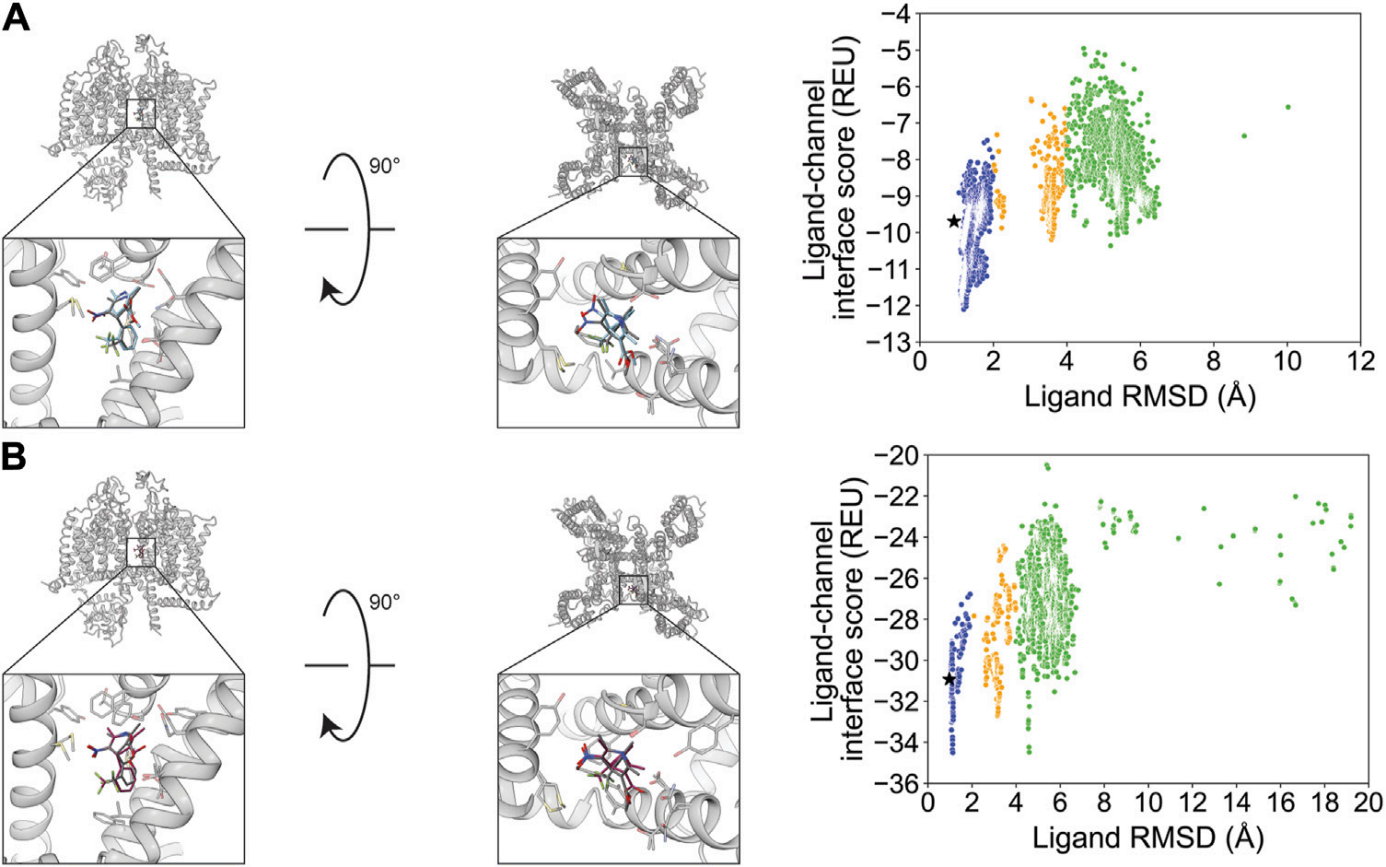
(S)-(−)-Bay K 8644 docking to the rabbit Ca_V_1.1 central pore cavity (PDB: 6JP8). **(A)** RosettaLigand (RMSD_Min_ = 0.93 Å and P_Near_ = 0.66) and **(B)** GALigandDock (RMSD_Min_ = 0.97 Å and P_Near_ = 0.61). Left: transmembrane view. Middle: extracellular view. Right: ligand RMSD vs. interface energy distribution of the top 10% of total energy poses. Gray dots: <1 Å; blue dots: 1–2 Å; yellow dots: 2–4 Å; and green dots: >4 Å. Carbon atoms of RosettaLigand molecules are shown in cyan, GALigandDock in dark pink, and experimental structure in dark gray. Non-carbon atoms match the Corey–Pauling–Koltun coloring scheme. Black star indicates the RMSD_Min_ pose from the entire population.

Nifedipine docking resulted in a RosettaLigand RMSD_Min_ value of 0.77 Å and a GALigandDock RMSD_Min_ value of 1.3 Å (Figure 5; Table 2). The calculated P_Near_ suggested little energy funnel convergence (RosettaLigand P_Near_ = 0.28 and GALigandDock P_Near_ = 0.29) (Table 3). This lack of energy convergence is exemplified by the interaction energy distributions containing multiple low-energy minima of RMSD 1–2 Å and 4–6 Å away from the experimental ligand coordinates (Figure 5). The lowest 10 interface energy poses with RosettaLigand contained poses between RMSD 1.1 and 4.4 Å, with most poses being 1.7 Å or less; 8 of the 10 poses converged to the experimental ligand coordinates with slight variation in rotamers, while 2 of the 10 poses flipped the position of the dihydropyridine and aromatic ring relative to the experimental ligand coordinates (Supplementary Figure S16.1). With GALigandDock, the lowest 10 interface energy poses were at RMSD 1.3 Å, in a similar position and orientation to the low RMSD conformations from RosettaLigand (Supplementary Figure S16.2).

(S)-(−)-Bay K 8644 docking resulted in a RosettaLigand RMSD_Min_ value of 0.93 Å and GALigandDock RMSD_Min_ value of 0.97 Å (Figure 6; Table 2). The calculated RosettaLigand P_Near_ was 0.66, and the GALigandDock P_Near_ was 0.61 (Table 3). The P_Near_ values, paired with the interaction energy distribution data, indicate well-converged energy funnels. Furthermore, the lowest-interaction energy poses with RosettaLigand were within 0.3 Å of the RMSD_Min_ pose (Supplementary Table S2.1), and for GALigandDock, were within 0.2 Å of the RMSD_Min_ pose (Supplementary Table S2.5). With RosettaLigand, the lowest 10 interface energy poses were between RMSD 1.2 and 1.3 Å, with all 10 poses converged to the experimental ligand coordinates with slight variation in rotamers (Supplementary Figure S17.1). With GALigandDock, the lowest 10 interface energy poses were at RMSD 1.1 Å or 4.6 Å. Eight of the 10 poses had RMSD 1.1 Å with a slight deviation in position to experimental ligand coordinates, while 2 of 10 poses had RMSD 4.6 Å with the dihydropyridine in the correct position, but the aromatic ring flipped 180° such that the trifluoromethyl group was pointed in the opposite direction (Supplementary Figure S17.2).

**FIGURE 7:**
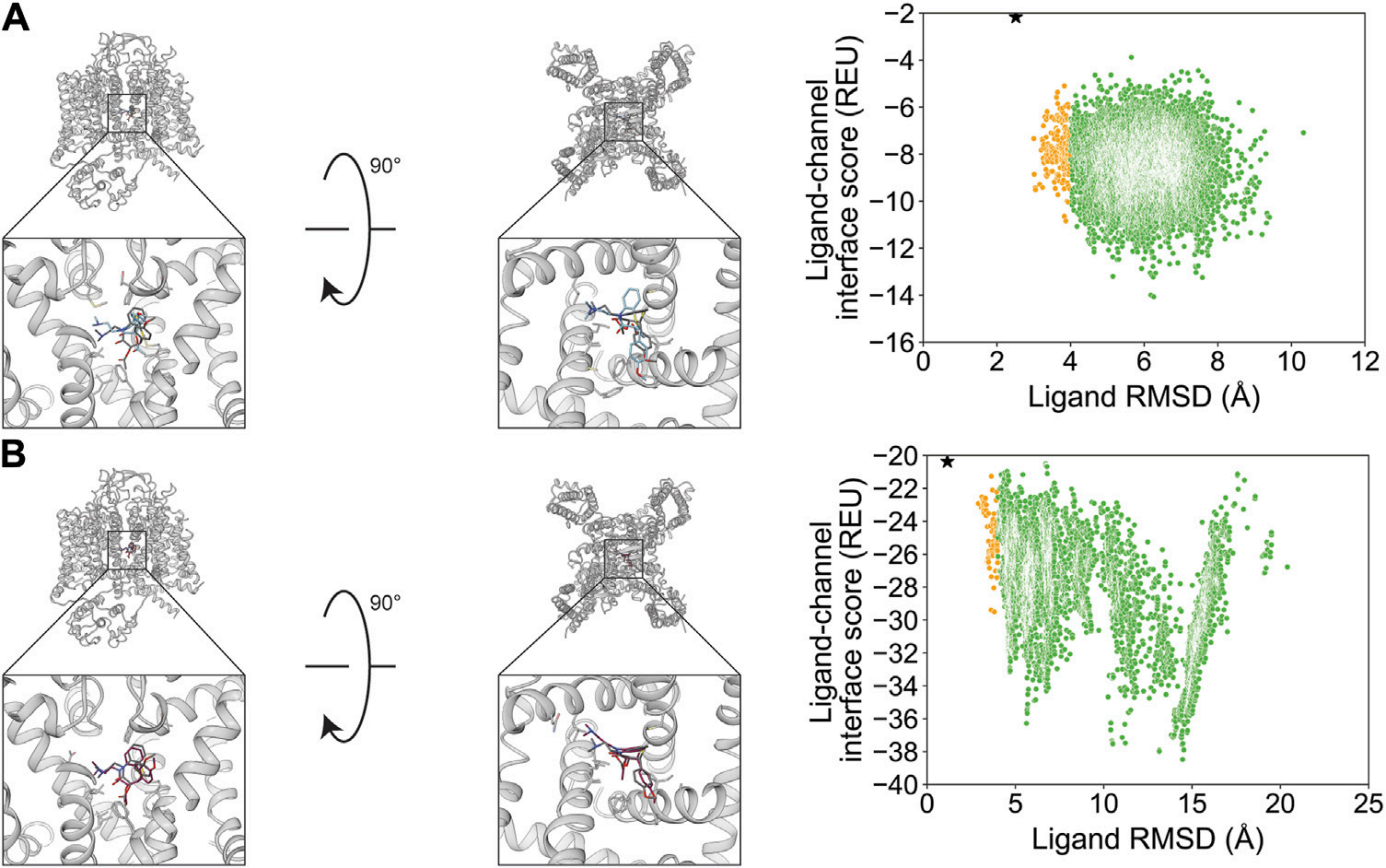
Diltiazem docking to the rabbit Ca_V_1.1 central pore cavity (PDB: 6JPB). **(A)** RosettaLigand (RMSD_Min_ = 2.5 Å and P_Near_ = 6.4•10^−4^) and **(B)** GALigandDock (RMSD_Min_ = 1.1 Å and P_Near_ = 7.8•10^−6^). Left: transmembrane view. Middle: extracellular view. Right: ligand RMSD vs. interface energy distribution of the top 10% of total energy poses. Yellow dots: 2–4 Å; green dots: >4 Å. Carbon atoms of RosettaLigand molecules are shown in cyan, GALigandDock in dark pink, and experimental structure in dark gray. Non-carbon atoms match the Corey–Pauling–Koltun coloring scheme. Black star indicates the RMSD_Min_ pose from the entire population.

Diltiazem docking resulted in a RosettaLigand RMSD_Min_ value of 2.5 Å and a GALigandDock RMSD_Min_ value of 1.1 Å (Figure 7; Table 2). The calculated P_Near_ suggested no energy funnel convergence for either method (RosettaLigand P_Near_ = 6.4•10^−4^ and GALigandDock P_Near_ = 7.8•10^−6^) (Table 3). This lack of energy funnel convergence is exemplified by the interaction energy distributions containing local minima RMSD 5–14 Å away from the experimental ligand coordinates (Figure 7). With RosettaLigand, the lowest 10 interface energy poses were between RMSD 5.2 and 7.6 Å, with all 10 poses at a similar channel depth at the pore center but with no convergence in local position or rotamer placement (Supplementary Figure S20.1). With GALigandDock, the lowest 10 interface energy poses were between RMSD 10.5 and 14.5 Å. Rather than being positioned in the pore center, all 10 poses were in a channel depth similar to the experimental ligand coordinates but positioned in fenestration with no convergence in local position or rotamer placement (Supplementary Figure S20.2).

**FIGURE 8:**
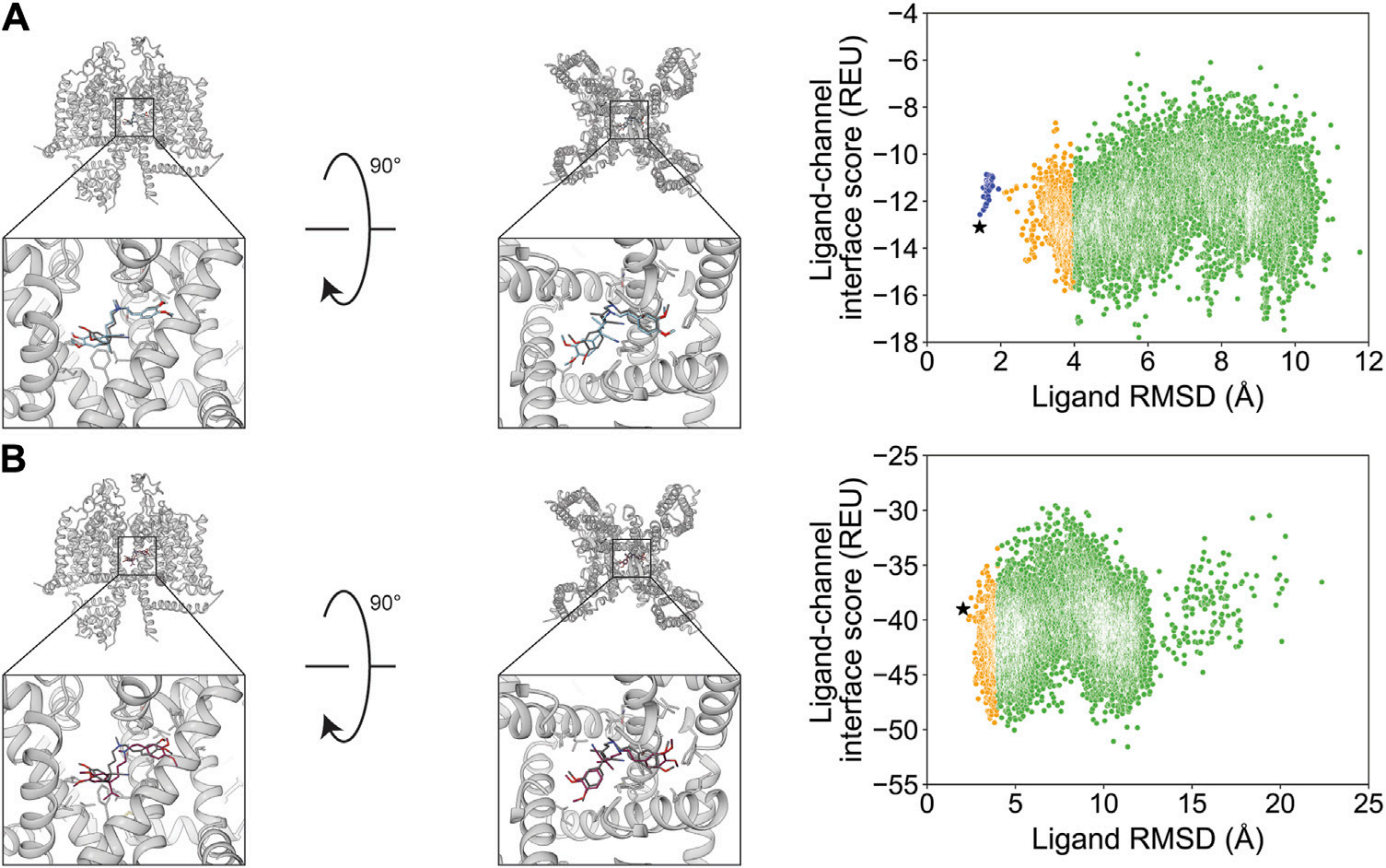
Verapamil docking in its first orientation to the rabbit Ca_V_1.1 central pore cavity (PDB: 6JPA). **(A)** RosettaLigand (RMSD_Min_ = 1.4 Å and P_Near_ = 4.3•10^−3^) and **(B)** GALigandDock (RMSD_Min_ = 2.0 Å and P_Near_ = 2.8•10^−3^). Left: transmembrane view. Middle: extracellular view. Right: ligand RMSD vs. interface energy distribution of the top 10% of total energy poses. Blue dots: 1–2 Å; yellow dots: 2–4 Å; and green dots: >4 Å. Carbon atoms of RosettaLigand molecules are shown in cyan, GALigandDock in dark pink, and experimental structure in dark gray. Non-carbon atoms match the Corey–Pauling–Koltun coloring scheme. Black star indicates the RMSD_Min_ pose from the entire population.

Verapamil was previously resolved in a complex with rabbit Ca_V_1.1 in two binding modes with flipped orientations (Zhao et al., 2019a). We evaluated whether RosettaLigand and GALigandDock sampling favored a particular orientation. For the first orientation, docking resulted in a RosettaLigand RMSD_Min_ value of 1.4 Å and a GALigandDock RMSD_Min_ value of 2.0 Å (Figure 8; Table 2). The calculated P_Near_ suggested no energy funnel convergence (RosettaLigand P_Near_ = 4.3•10^−3^ and GALigandDock P_Near_ = 2.8•10^−3^) (Table 3). This nonexistent energy funnel is evident by local interaction energy minima beyond RMSD 4 Å from the experimental ligand coordinates (Figure 8). With RosettaLigand, the lowest 10 interface energy poses were between RMSD 4.1 and 9.7 Å, with all 10 poses at a similar channel depth, and some poses converging in local position and rotamer placement at RMSD 5.7 and 9.2 Å (Supplementary Figure S18.1). With GALigandDock, the lowest 10 interface energy poses were between RMSD 3.8 and 11.9 Å. All 10 poses were at different channel depths, positioned in the pore center or at the pore center–fenestration interface, and did not converge in local position or rotamer placement (Supplementary Figure S18.2).

**FIGURE 9:**
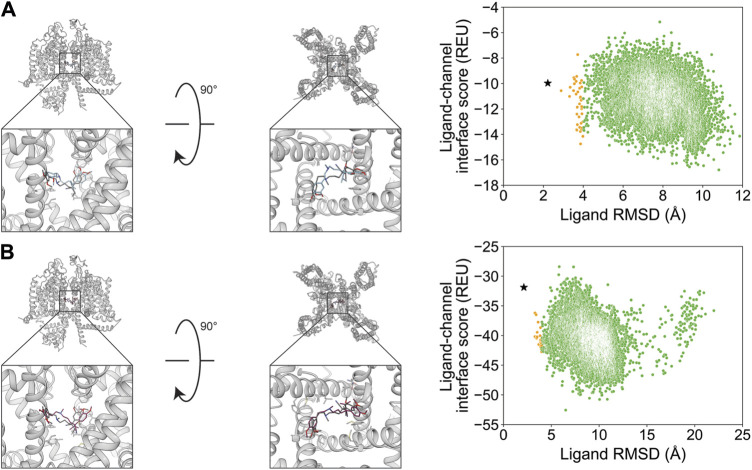
Verapamil docking in its second orientation to the rabbit Ca_V_1.1 central pore cavity (PDB: 6JPA). **(A)** RosettaLigand (RMSD_Min_ = 2.2 Å and P_Near_ = 1.2•10^−4^) and **(B)** GALigandDock (RMSD_Min_ = 2.1 Å and P_Near_ = 1.9•10^−5^). Left: transmembrane view. Middle: extracellular view. Right: ligand RMSD vs. interface energy distribution of the top 10% of total energy poses. Yellow dots: 2–4 Å; green dots: >4 Å. Carbon atoms of RosettaLigand molecules are shown in cyan, GALigandDock in dark pink, and experimental structure in dark gray. Non-carbon atoms match the Corey–Pauling–Koltun coloring scheme. Black star indicates the RMSD_Min_ pose from the entire population.

For the second orientation, docking resulted in a RosettaLigand RMSD_Min_ value of 2.2 Å and a GALigandDock RMSD_Min_ value of 2.1 Å (Figure 9; Table 2). The calculated P_Near_ values suggest no energy funnel convergence (RosettaLigand P_Near_ = 1.2•10^−4^ and GALigandDock P_Near_ = 1.9•10^−5^) (Table 3). Similar to the first orientation, the interaction energy distribution for the second orientation yields local energy minima greater than RMSD 4 Å from the experimental ligand coordinates (Figure 9). With RosettaLigand, the lowest 10 interface energy poses were between RMSD 8.5 and 10.8 Å, with all 10 poses at a similar channel depth, and some poses converging in local positions, similar to the docking set for the first structural orientation of verapamil (Supplementary Figure S19.1). With GALigandDock, the lowest 10 interface energy poses were between RMSD 6.5 and 10.8 Å. All 10 poses were at different channel depths, positioned in the pore center or at the pore center–fenestration interface, and did not converge in local position or rotamer placement, similar to the docking set for the first structural orientation of verapamil (Supplementary Figure S19.2).

**FIGURE 10:**
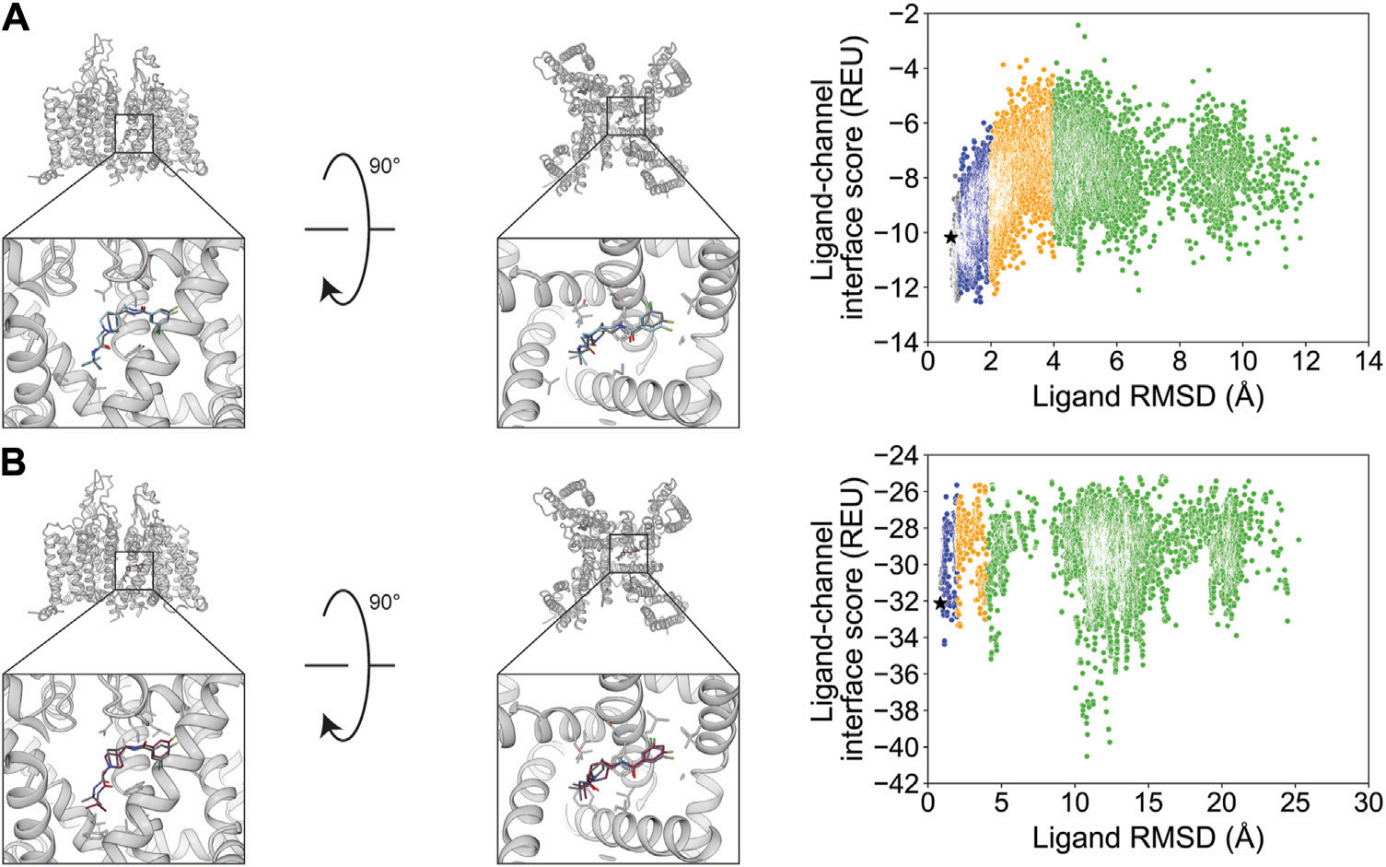
Z944 docking to the human Ca_V_3.1 central pore cavity (PDB: 6KZP). **(A)** RosettaLigand (RMSD_Min_ = 0.73 Å and P_Near_ = 0.62) and **(B)** GALigandDock (RMSD_Min_ = 0.84 Å and P_Near_ = 1.1•10^−4^). Left: transmembrane view. Middle: extracellular view. Right: ligand RMSD vs. interface energy distribution of the top 10% of total energy poses. Gray dots: <1 Å; blue dots: 1–2 Å; yellow dots: 2–4 Å; and green dots: >4 Å. Carbon atoms of RosettaLigand molecules are shown in cyan, GALigandDock in dark pink, and experimental structure in dark gray. Non-carbon atoms match the Corey–Pauling–Koltun coloring scheme. Black star indicates the RMSD_Min_ pose from the entire population.

The small molecule Z944 (channel blocker) has six rotatable bonds and is resolved bound to human Ca_V_3.1. Docking with RosettaLigand and GALigandDock both resulted in RMSD_Min_ values of 0.73 and 0.84 Å, respectively (Figure 10; Table 2). Interestingly, the calculated P_Near_ value suggests good energy funnel convergence for RosettaLigand (P_Near_ = 0.62) but no energy funnel convergence for GALigandDock (P_Near_ = 1.1•10^−4^) (Table 3). For RosettaLigand, the interaction energy produced an energy funnel between 1 and 2 Å, while for GALigandDock, the interaction produced an energy funnel between 10 and 15 Å (Figure 10). With RosettaLigand, the lowest 10 interface energy poses were between RMSD 0.90 and 1.7 Å, with all 10 poses converged to the experimental ligand coordinates with slight variation in rotamers (Supplementary Figure S21.1). With GALigandDock, the lowest 10 interface energy poses were between RMSD 10.6 and 12.4 Å, with 4 sets of unique poses identified. The first set, containing 3 of the 10 poses, is in a slightly lower channel depth and in a similar orientation to the experimental ligand coordinates but rotated approximately 180° about the pore center. The second set is one pose and is in a similar location as the first set but is rotated such that the phenyl ring and tertiary butylamine positions are flipped. The third group, containing four poses, is similar to the second set but rotated approximately 180° about the pore center. The last group, containing two poses, is in a similar channel depth and location to the experimental ligand coordinates, but the phenyl ring and the tertiary butylamine positions are flipped (Supplementary Figure S21.2).

**FIGURE 11:**
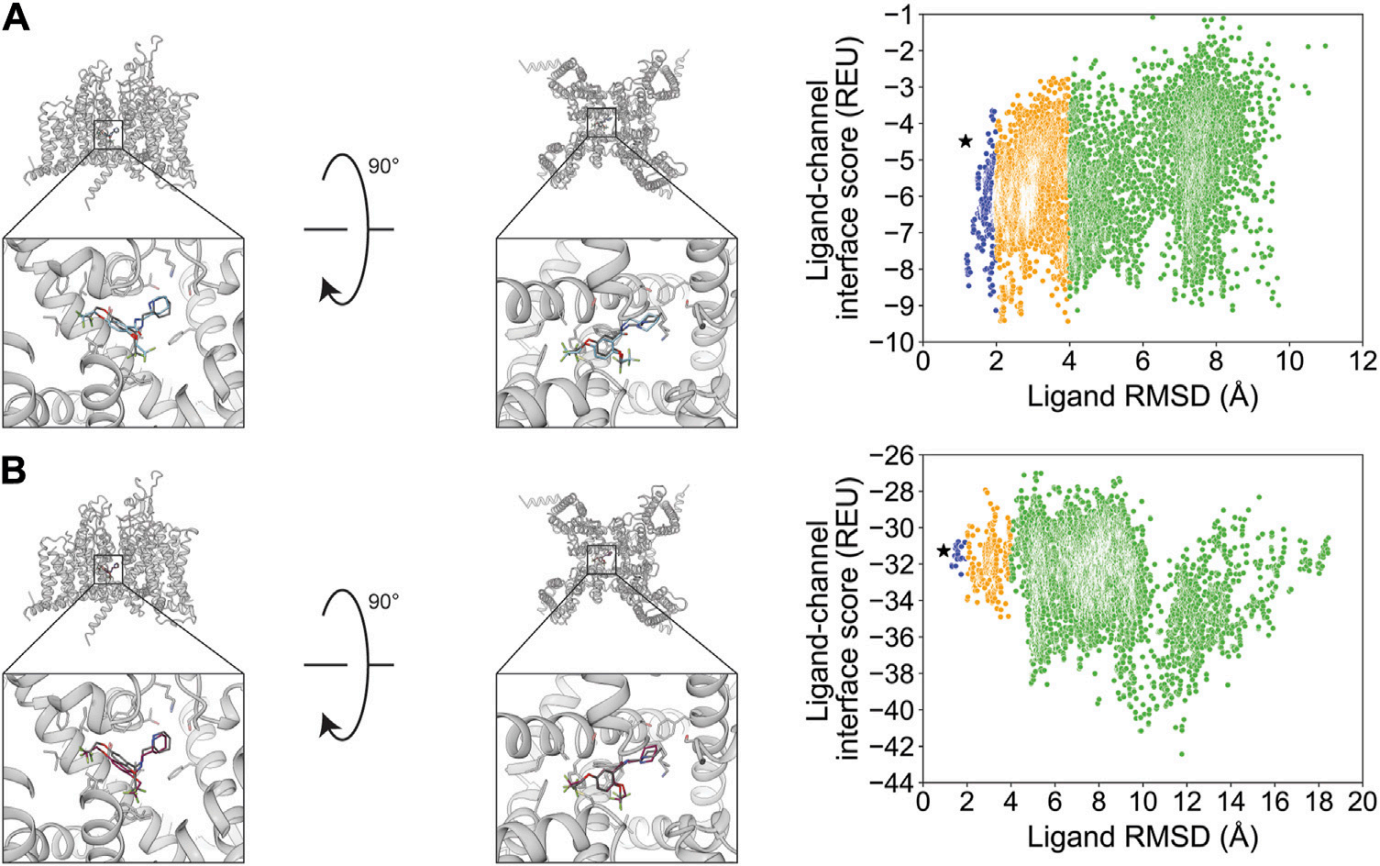
Flecainide docking to the rat Na_V_1.5 central pore cavity (PDB: 6UZ0). **(A)** RosettaLigand (RMSD_Min_ = 1.2 Å and P_Near_ = 0.12) and **(B)** GALigandDock (RMSD_Min_ = 0.94 Å and P_Near_ = 2.9•10^−5^). Left: transmembrane view. Middle: extracellular view. Right: ligand RMSD vs. interface energy distribution of the top 10% of total energy poses. Blue dots: 1–2 Å; yellow dots: 2–4 Å; and green dots: >4 Å. Carbon atoms of RosettaLigand molecules are indicated in cyan, GALigandDock in dark pink, and experimental structure in dark gray. Non-carbon atoms match the Corey–Pauling–Koltun coloring scheme. Black star indicates the RMSD_Min_ pose from the entire population.

Flecainide (channel blocker) has seven rotatable bonds and is resolved bound to rat Na_V_1.5. RosettaLigand docking resulted in an RMSD_Min_ value of 1.2 Å, while GALigandDock resulted in an RMSD_Min_ value of 0.94 Å (Figure 11; Table 2). The calculated P_Near_ values suggest no energy funnel convergence with either method (RosettaLigand P_Near_ = 0.12, GALigandDock P_Near_ = 2.9•10^−5^) (Table 3). The interaction energy distribution from RosettaLigand produced an energy minimum between 2–4 Å and 7–10 Å, while GALigandDock produced energy minima between 10 and 12 Å (Figure 11). With RosettaLigand, the lowest 10 interface energy poses were between RMSD 2.1 and 4.0 Å, with all 10 poses in the same channel depth as the experimental ligand coordinates; however, for each pose, both sets of trifluoromethyl groups are at relatively the same channel depth, compared to the experimental ligand coordinates where one trifluoromethyl is lower in the channel than in the other group (Supplementary Figure S23.1). In one pose, piperidin-2-yl-methylamine was positioned lower into the channel than the rest of the ligand and the experimental ligand coordinates, while the rest of the ligand pose was positioned similar to other poses (Supplementary Figure S23.1). With GALigandDock, the lowest 10 interface energy poses were between RMSD 9.7 and 11.9 Å, with all 10 poses positioned at the fenestration in the same channel depth as the experimental ligand coordinates but oriented outside the pore, with one of the trifluoromethyl groups pointing toward the pore center but positioned at the exterior of the channel fenestration (Supplementary Figure S23.2).

The TRPV subfamily of cation channels broadly plays roles in thermo-sensation and thermoregulation in response to heat (>53 °C), notably in noxious heat-sensing (Nilius, 2007; Gees et al., 2012). TRPV2 channels are widely expressed, with identification, in dorsal root ganglion neurons and the brain, heart, and smooth muscle tissue (Gees et al., 2012). The TRPV2 channel has been implicated in thermal pain-sensing, muscular dystrophy, and cardiomyopathy, among other diseases (Nilius, 2007; Gees et al., 2012). Structurally, TRPV2 channels contain six transmembrane segments and commonly assemble as a homotetramer (Zheng and Trudeau, 2015). They resemble canonical voltage-gated ion channels with a pore domain and a weak voltage sensor domain, while their gating is regulated by heat and a diverse set of agonists such as 2-aminoethoxydiphenyl borate and cannabidiol (Gees et al., 2012; Zheng and Trudeau, 2015; Pumroy et al., 2019).

**FIGURE 12:**
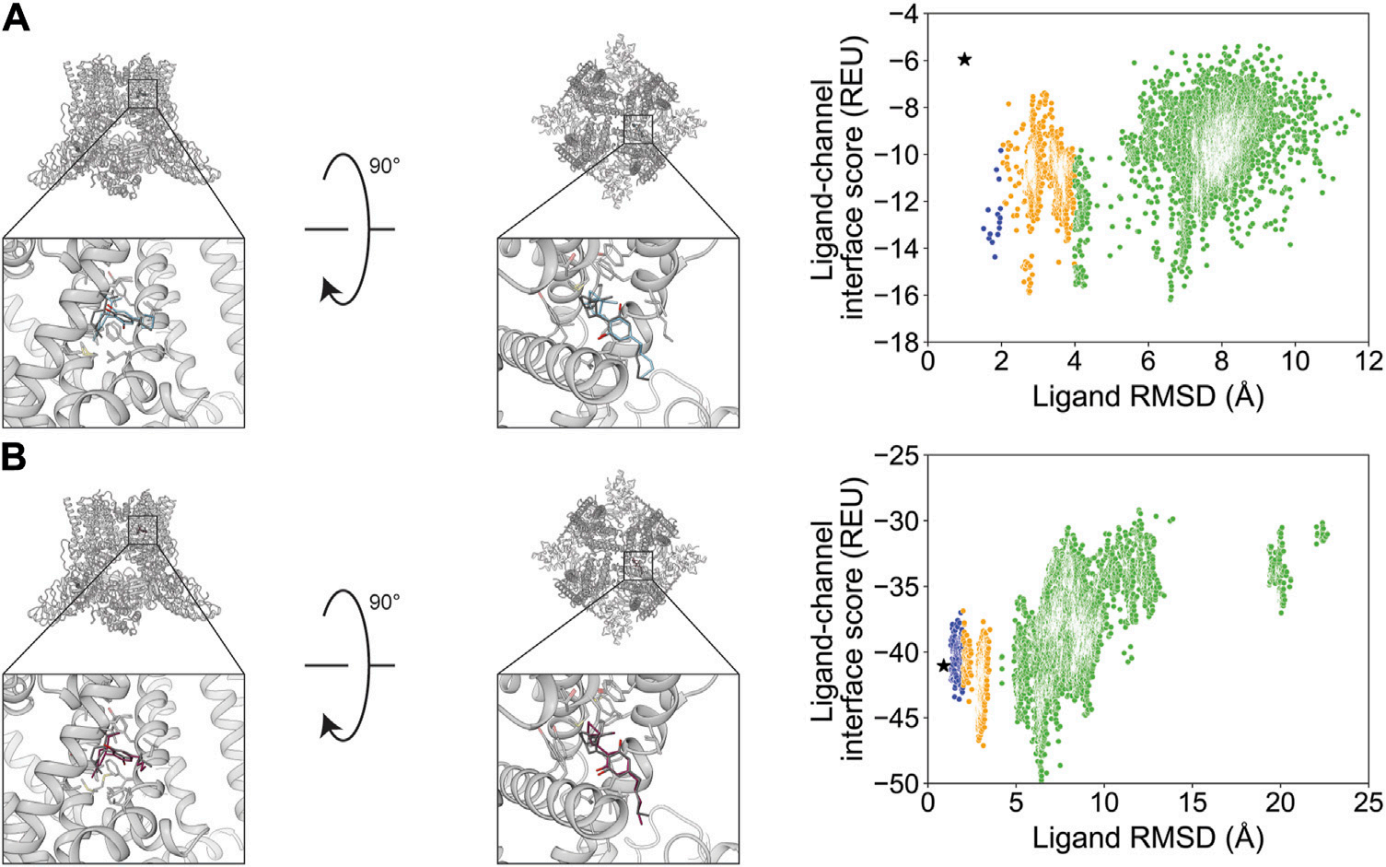
Cannabidiol docking to the rat TRPV2 central pore cavity (PDB: 6U88). **(A)** RosettaLigand (RMSD_Min_ = 1.0 Å and P_Near_ = 4.3•10^−2^) and **(B)** GALigandDock (RMSD_Min_ = 0.90 Å and P_Near_ = 3.8•10^−3^). Left: transmembrane view. Middle: extracellular view. Right: ligand RMSD vs. interface energy distribution of the top 10% of total energy poses. Blue dots: 1–2 Å; yellow dots: 2–4 Å; and green dots: >4 Å. Carbon atoms of RosettaLigand molecules are shown in cyan, GALigandDock in dark pink, and experimental structure in dark gray. Non-carbon atoms match the Corey–Pauling–Koltun coloring scheme. Black star indicates the RMSD_Min_ pose from the entire population.

Cannabidiol (channel activator) has six rotatable bonds and is resolved bound to rat TRPV2. Docking with RosettaLigand resulted in an RMSD_Min_ value of 1.0 Å, while GALigandDock resulted in an RMSD_Min_ value of 0.90 Å (Figure 12; Table 2). The calculated P_Near_ values suggest no energy funnel convergence for both methods (RosettaLigand P_Near_ = 4.3•10^−2^ and GALigandDock P_Near_ = 3.8•10^−3^) (Table 3). For RosettaLigand, the interaction energy distribution did not demonstrate a clear minimum, with minima observed between 2 and 8 Å (Figure 12). For GALigandDock, there is a clear minimum between 6 and 7 Å (Figure 12). With RosettaLigand, the lowest 10 interface energy poses were between 2.8 and 7.0 Å RMSD, with two groups of conformations in the same channel depth and interfaced with the S5 and S6 helices of adjacent TRPV2 monomers like the experimental ligand coordinates. In one group, cannabidiol is positioned parallel to the S6 helical segment, with the cyclohexene at the lowest channel depth. In the second group, the poses are positioned in the same orientation as the experimental ligand coordinates, with the pentyl chain facing away from the pore center, but the overall position of cannabidiol is slightly elevated relative to the experimental ligand coordinates (Supplementary Figure S22.1). With GALigandDock, the lowest 10 interface energy poses were either RMSD 6.4 or 6.7 Å, with all 10 poses at fenestration in the same channel depth and interfacing with the S5 and S6 helices of adjacent TRPV2 monomers like the experimental ligand coordinates but with the ligand rotated around the cyclohexene plane such that the pentyl chain is oriented parallel to the S6 helix rather than pointing outward (Supplementary Figure S22.2).

## Discussion

Previous studies have underscored the importance of ligand docking methods for generating ion channel structure-based hypotheses (Yang et al., 2013; Shim et al., 2019). Furthermore, ligand docking methods when combined with high-resolution structures can aid in rational drug design (Wang et al., 2007; Liu et al., 2018; Wulff et al., 2019; Maia et al., 2020). Notably, RosettaLigand has been extensively used to predict the molecular mechanisms of ligand–ion channel interactions (Yang et al., 2013; Yang et al., 2015; Nguyen et al., 2017; Nguyen et al., 2019; Shim et al., 2019; Craig 2^nd^ et al., 2020; Vu et al., 2020; Maly et al., 2022; Pumroy et al., 2022). While GALigandDock has not yet been tested on ion channel structure–function relationships, it utilizes a new generalized energy function tailored for small molecules while sampling ligand conformations with a genetic algorithm, making it an attractive complement to RosettaLigand.

Indeed, this study demonstrates that high-resolution structures paired with RosettaLigand and GALigandDock can be useful tools for formulating structural hypotheses and predicting binding modes for drug discovery. Notably, both Rosetta methods can produce ligand poses near the experimental ligand coordinates. Using a standard RMSD_Min_ cutoff of 2 Å (Maia et al., 2020; Park et al., 2021), the RosettaLigand docking set yielded an average RMSD_Min_ value of 1.2 ± 0.6 Å, and the GALigandDock docking set yielded an average RMSD_Min_ value of 1.1 ± 0.5 Å (Table 2). However, the ability to discriminate the RMSD_Min_ pose remains challenging and highlights important practical considerations when applying RosettaLigand docking methods to a chosen ion channel target.

When performing RosettaLigand docking, the features of the ligand conformer library, the size of the receptor site, and prior knowledge about critical functional residues need to be considered to determine the appropriate amount of pose sampling. For our study, we generated either 20,000 poses or 100,000 poses per docking run. Although we did observe a statistically significant difference in RMSD_Min_ between 20,000 and 100,000 poses, the difference was within a few sub-Angstroms. Furthermore, another RosettaLigand benchmarking study of the CASF-2016 dataset generated a total of 1,000 poses and sufficiently sampled an RMSD_Min_ value below 2 Å (Su et al., 2019; Smith and Meiler, 2020). Thus, for ion channel docking, we suggest sampling in the ones to tens of thousands, especially when docking in large receptor sites like the central pore cavity.

Overall, our docking data achieved a P_Near_ value greater than 0.5 for two test cases for each docking method when using the top 10% of total-scoring poses. Both methods achieved P_Near_ greater than 0.5 for (S)-(−)-Bay K 8644 to the rabbit Ca_V_1.1 central pore cavity, while RosettaLigand achieved P_Near_ greater than 0.5 for Z944 to the human Ca_V_3.1 central pore cavity, and GALigandDock achieved P_Near_ greater than 0.5 for GX-936 docking to hNa_V_1.7-Na_V_Ab VSD4 (Table 3). The reasons for a low P_Near_ value in most test cases are possibly due to 1) improper scoring by Rosetta score functions to discriminate low-RMSD poses by energy; 2) multiple favorable ligand-binding states in the receptor site that have not been structurally resolved; and/or 3) insufficient pose sampling.

Currently, we suggest that Rosetta score functions are unable to reliably score poses by energy in ion channel docking, especially for the central pore cavity. For each docking run, comparing the RMSD_Min_ pose and the lowest interface energy pose indicates that the RMSD_Min_ pose is underscored (Supplementary Tables S2.1–S2.5). The only notable exception is the case where we docked to the voltage-sensing domain (PDB: 5EK0). Conversely, a previous RosettaLigand study using the CASF-2016 dataset (containing 285 crystal structures of protein–ligand complexes with an overall resolution <2.5 Å and an R-factor <0.25) and 1,000 poses per docking run frequently achieved P_Near_ values between 0.8 and 1.0 (Su et al., 2019; Smith and Meiler, 2020), suggesting that the Rosetta score functions can be utilized for other docking studies but should be verified for accuracy with test cases. Furthermore, the CASF-2016 dataset does not contain ion channels, while the sample size per docking run was sufficiently lower in the RosettaLigand docking study using CASF-2016 than those in our study, suggesting that sampling is not the predominant issue, but rather, the scoring of poses is the primary issue.

For the voltage-sensing domains, GX-936 in complex with VSD4 of the hNa_V_1.7-Na_V_Ab chimera (PDB: 5EK0) was the only small molecule docked yet that was consistently near or below RMSD_Min_ 1 Å for each docking set regardless of the method (Supplementary Tables S1.1, S1.2). This may be due to the VSD4 receptor site being the narrowest binding pocket tested, thereby limiting the number of binding configurations. This suggests that VSDs are generally well suited for RosettaLigand docking since the receptor is constrained to allow shape complementarity between the ligand and protein while reducing the required sampling compared to pore-forming receptor sites. Notably, the RosettaLigand docking methods employed did not use any implicit membrane parameters, while GX-936 in a biologically realistic context is partially exposed to a lipid head group (Ahuja et al., 2015). Thus, in this case, docking without an implicit membrane energy function still achieved the experimental structurally resolved position; however, artifactual, low-interface energy poses where GX-936 would be overlapping the phospholipid space are present (Supplementary Figures S13.1, S13.2). Furthermore, GALigandDock consistently achieved P_Near_ > 0.5 when using a padding of 2.0, 4.0, or 7.0 Å, suggesting that ligands docked to VSDs could be screened by energy funnel convergence (Supplementary Tables S4.5–S4.10). More small molecules structurally resolved and docked to the VSD are needed to validate this observation.

For the outer pore, TTX and STX were docked to hNa_V_1.7 as they are canonical small molecule channel blockers used when studying the Na_V_ family. TTX and STX had the fewest rotatable bonds in this study, suggesting that little conformer sampling would be needed to generate a pose with a low RMSD to the experimental ligand coordinates. Both TTX and STX docking achieved sub-Angstrom RMSD_Min_, except STX docking with GALigandDock at a padding of 7 Å (RMSD_Min_ = 2.3 Å, padding 7 Å/100,000 poses vs. RMSD_Min_ = 1.0 Å, padding 4 Å/100,000 poses; Supplementary Table S1.2). However, all docking methods employed were unable to achieve P_Near_ > 0.5, suggesting that 1) STX and TTX could have alternative binding conformations; 2) the docking methods employed have wrongfully scored alternative low-energy conformations; or 3) that inherent loop flexibility in the outer pore is a requirement for docking an energy-optimized, induced-fit conformation. It should be noted that the hNa_V_1.7 selectivity filter is lined with polar residues that could contribute to a vast hydrogen-bonding network, in addition to water hydrating the selectivity filter and the filter opening. Thus, it is unclear whether the lowest-energy poses, with potential salt bridge interactions, are possible alternate binding modes. It is thus possible that the sum of hydrogen bonding interactions and water-bridging effects could bias the energetic potential to a certain state, such as the one structurally resolved. Thus, further experimental characterization is needed to test these structural hypotheses.

Docking to the central pore cavity produced the most pose variability due to it having the largest sampled area compared to the outer pore and the voltage-sensing domain. For example, the second orientation of verapamil positioned primarily in the central cavity of rabbit Ca_V_1.1 achieved an RMSD_Min_ value of 1.4 Å for RosettaLigand and 2.0 Å for GALigandDock (Table 2). However, Z944 bound to hCa_V_3.1, with the wide aromatic end of Z944 in the narrow fenestration and the narrow amide end in the wide cavity, achieved an RMSD_Min_ value of 0.73 Å for RosettaLigand and 0.84 Å for GALigandDock (Table 2). Furthermore, RosettaLigand achieved a P_Near_ value greater than 0.5 under some docking conditions for Z944 (Table 3; Supplementary Tables S4.1–S4.4), suggesting that the Rosetta docking methods could prove useful when docking similar ligands that bridge between the fenestration and central pore and target the narrow fenestration with an aromatic moiety.

Small molecules docked in the central cavity were bound to the central pore (6JPA, orientation 2, and 6JPB), the channel fenestration region (6JP5, 6JP8, and 6U88), or both regions (6JPA, orienation 1, 6KZP, and 6UZ0). It appears that those bound in the channel fenestration produced a lower RMSD_Min_, those primarily bound in the pore center produced the largest RMSD_Min_, while those bound to both regions produced intermediate RMSD_Min_ values, with Z944 docked to the human Ca_V_3.1 central pore cavity (6KZP) being an exception. The same trend was roughly observed for P_Near_, where fenestration-only cases have P_Near_ values of orders of magnitude greater than pore-center cases (Supplementary Table S5). Due to the limited number of cases for each sub-classification, further studies will need to be performed to assess a correlation.

It appears that molecules with predominantly planar aromatic rings and space-filling, “bulky” structures targeting the fenestration can be scored and ranked effectively; both (S)-(−)-Bay K 8644 (6JP8) and Z944 (6KZP) possess these features in contrast to other small molecules that possess aromatic or aliphatic rings but are generally more linear and “floppy,” targeting fenestration, like flecainide (6UZ0) and the first orientation of verapamil (6JPA) (Supplementary Table S5). This general trend in increased RMSD_Min_ values for non-aromatic rings containing small molecules when the receptor site is solely the central pore could be due to 1) greater ligand flexibility within a larger area, which requires increased sampling; 2) a bulkier ligand having fewer conformations and orientations than the channel; 3) the absence of explicit water molecules and ions that would crowd the pore or are hypothesized to directly bind to the ligand (Dilmac et al., 2003; Tikhonov and Zhorov, 2008); and 4) the inability to model intrusive lipids that could interact with the ligand at the fenestration region (Tikhonov and Zhorov, 2022; Chen et al., 2023). Furthermore, if the ligand is expected to bind to a central cavity motif present on multiple subunits, then *post hoc* tools implementing symmetry to discriminate unique binding modes will be necessary to calculate an appropriate adjusted RMSD_Min_.

## Conclusion

In this study, we aimed to assess whether 1) the RosettaLigand and GALigandDock molecular docking methodologies can recapitulate structurally resolved ion channel–small molecule binding orientations with known pharmacological significance; 2) their scoring functions can be used to accurately rank small-molecule binding orientation for the purpose of blindly screening small molecules; and 3) there are practical considerations when docking to specific domains of ion channels. With an RMSD_Min_ value of 2.0 Å RMSD_Min_ as a performance cutoff (Maia et al., 2020; Park et al., 2021), our results demonstrate that both RosettaLigand and GALigandDock can frequently sample the experimentally resolved ligand-binding mode with less than RMSD 2.0 Å. However, when using an estimate of the Boltzmann probability for energy “funnel-likeness” (P_Near_) as a scoring function assessment, we currently perceive Rosetta score functions as being unable to reliably score poses accurately in ion channel docking; from this study, small molecules targeting voltage-sensing domains and bulky small molecules primarily composed of aromatic rings targeting fenestration regions appear to be most suited for score-based ranking. Thus, when performing an ion channel virtual drug-discovery campaign, special consideration should be given to sufficient pose sampling to account for multiple rotameric and conformational states, identify the size of the sample required for sufficient interface scoring of the receptor site, identify the appropriate state of the ion channel, identify inherent channel flexibility that could influence ligand binding, and potentially identify specific chemical functional groups known experimentally to influence binding to the target when selecting candidate conformations. Recent advances in deep learning methods for protein–ligand structural prediction, such as RoseTTAFold All-Atom (Krishna et al., 2024), also allow alternatives to traditional physics-based docking algorithms, while new prediction categories in the Critical Assessment of Structure Prediction reflect the ever-increasing accuracy of biomolecular modeling (Kryshtafovych et al., 2023). Indeed, in the most recent 2022 CASP15 assessment, an updated protein–ligand complex category was introduced, with the goal of predicting entirely *de novo* protein–small molecule complexes from sequence, protein stoichiometry, and ligand SMILES codes alone (Robin et al., 2023), with evaluation metrics being the binding site superposed, symmetry-corrected pose RMSD (BiSyRMSD), and the local distance difference test for protein–ligand interactions (lDDT-PLI) to assess whether reference contacts are reproduced. Future studies should assess the performance of these emerging methods on the currently resolved ion channel–ligand complexes, generate predictions of known ion channel–ligand interactions that have not been structurally resolved, and then acquire such structures.

## Data Availability

RosettaLigand inputs, GALigandDock inputs, text-readable score files from all test conditions, and models of the top ten RMSD and interface score poses for each test case are available in the Dryad database: https://doi.org/10.5061/dryad.m63xsj49v.
