# Supplementary material for: Toward high-resolution modeling of small molecule–ion channel interactions
Source: Front Pharmacol. 2024 Jun 11;15:1411428. doi: 10.3389/fphar.2024.1411428 (PMC11196768; doi:10.3389/fphar.2024.1411428)
Supplement: Supplementary file 1 [file DataSheet1.PDF]

Supplementary Material for:

Towards high-resolution modeling of small molecule – ion channel interactions

Brandon J. Harris[1,2], Phuong T. Nguyen[1], Guangfeng Zhou[3], Heike Wulff[4], Frank DiMaio[3], Vladimir Yarov-Yarovoy[1,2,5]*

[1]Department of Physiology and Membrane Biology, University of California Davis, Davis, California, United States

[2]Biophysics Graduate Group, University of California Davis, Davis, California, United States

[3]Department of Biochemistry, University of Washington, Seattle, Washington, USA; Institute for Protein Design, University of Washington, Seattle, Washington, USA.

[4]Department of Pharmacology, School of Medicine, University of California Davis, Davis, California, United States

[5]Department of Anesthesiology and Pain Medicine, University of California Davis, Davis, California, United States

*** Correspondence:**
Vladimir Yarov-Yarovoy
yarovoy@ucdavis.edu

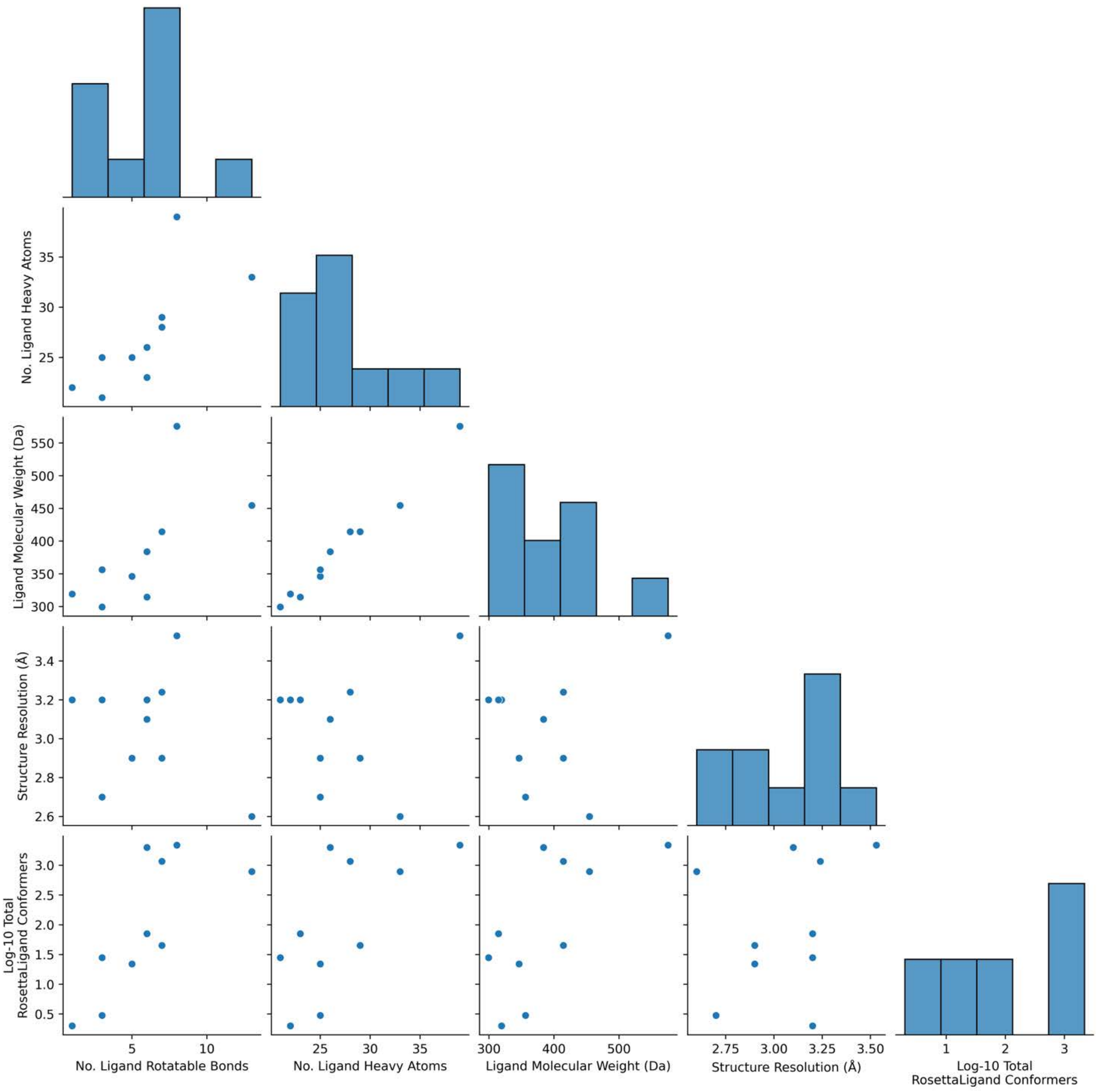

Figure S1. Pair plots and histograms of covariates. The logarithm base-10 of the total number of conformers is used in RosettaLigand only.

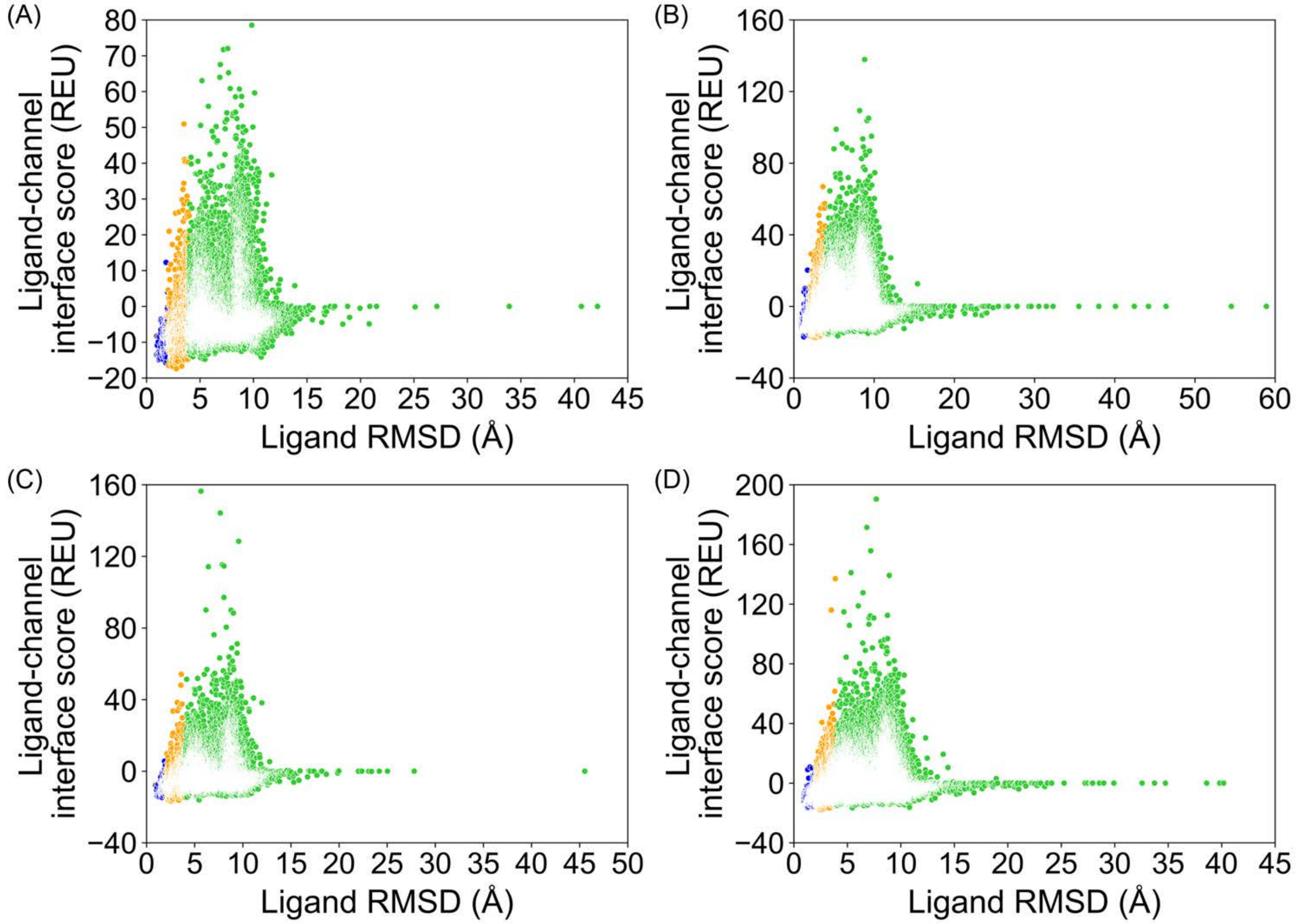

Figure S2.1 PDB 5EK0, RosettaLigand, all poses from docking. **(A)** 20,000 total poses, all ligand atom interface, **(B)** 100,000 total poses, all ligand atom interface, **(C)** 20,000 total poses, ligand neighbor atom interface, **(D)** 100,000 total poses, ligand neighbor atom interface.

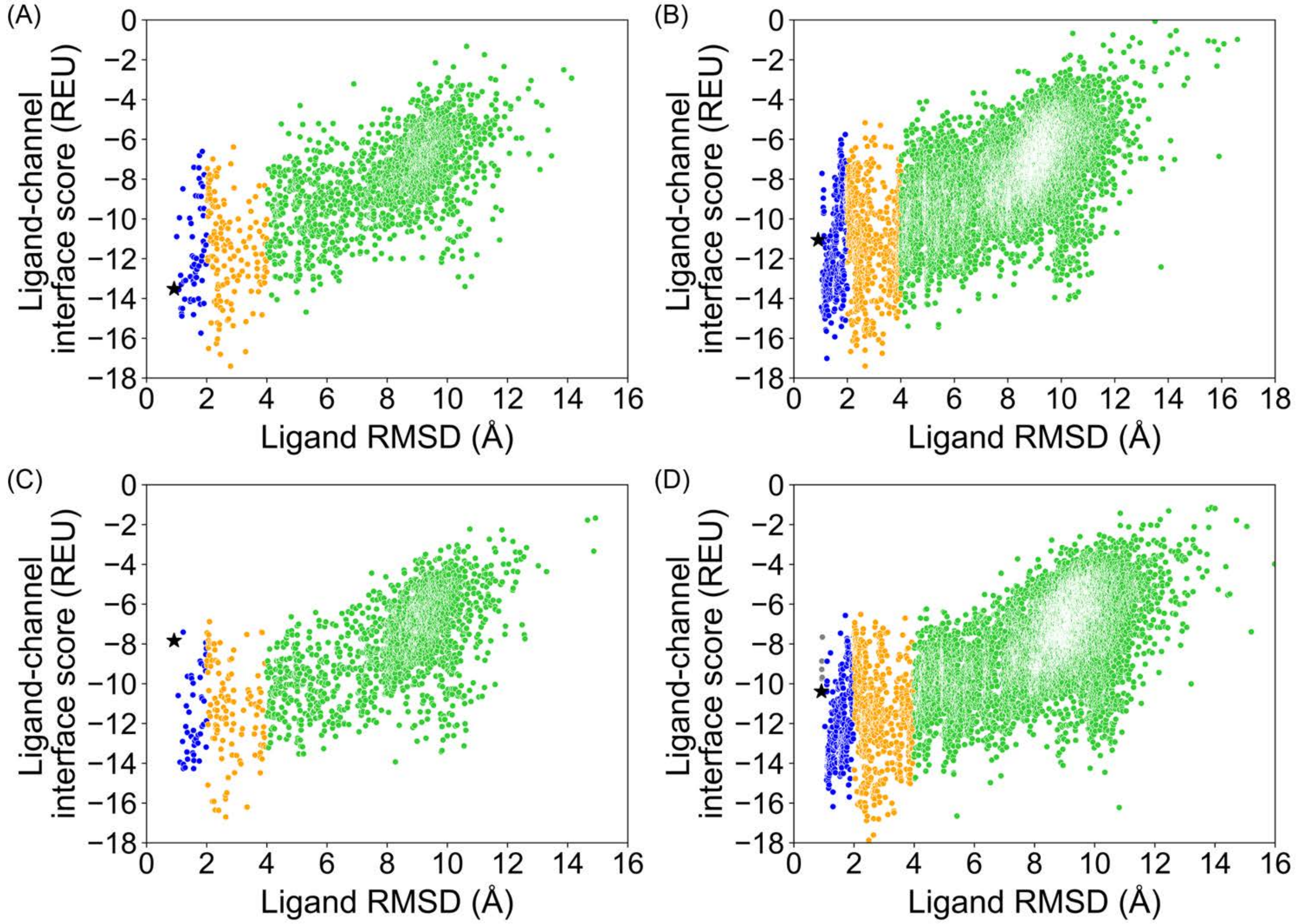

Figure S2.2 PDB 5EK0, RosettaLigand, top 10 % of total poses by total energy (`total_score`). **(A)** 20,000 total poses, all ligand atom interface, **(B)** 100,000 total poses, all ligand atom interface, **(C)** 20,000 total poses, ligand neighbor atom interface, **(D)** 100,000 total poses, ligand neighbor atom interface.

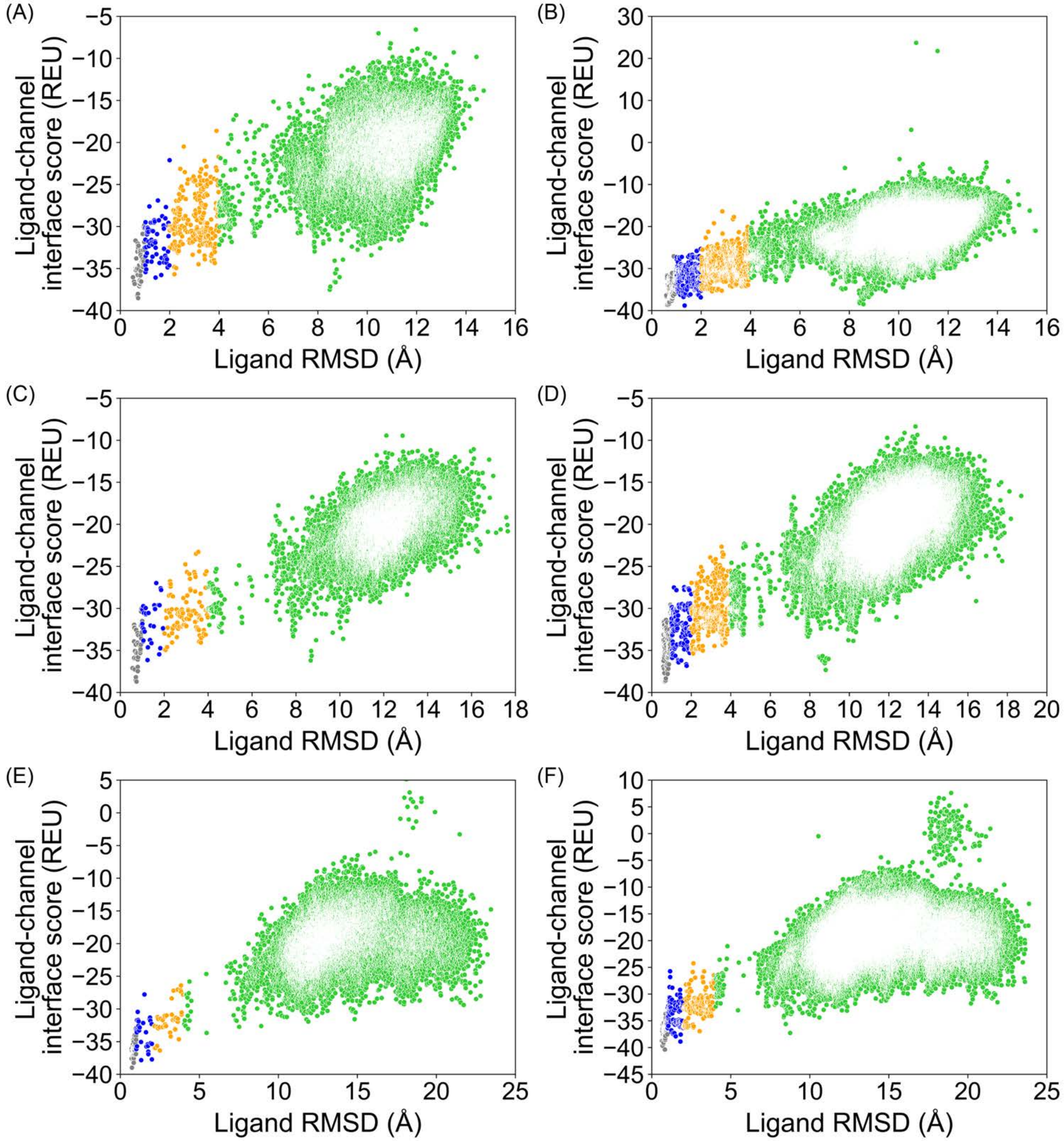

Figure S2.3 PDB 5EK0, GALigandDock, all poses from docking. **(A)** 20,000 total poses, padding 2 Å, **(B)** 100,000 total poses, padding 2 Å, **(C)** 20,000 total poses, padding 4 Å, **(D)** 100,000 total poses, padding 4 Å, **(E)** 20,000 total poses, padding 7 Å, **(F)** 100,000 total poses, padding 7 Å.

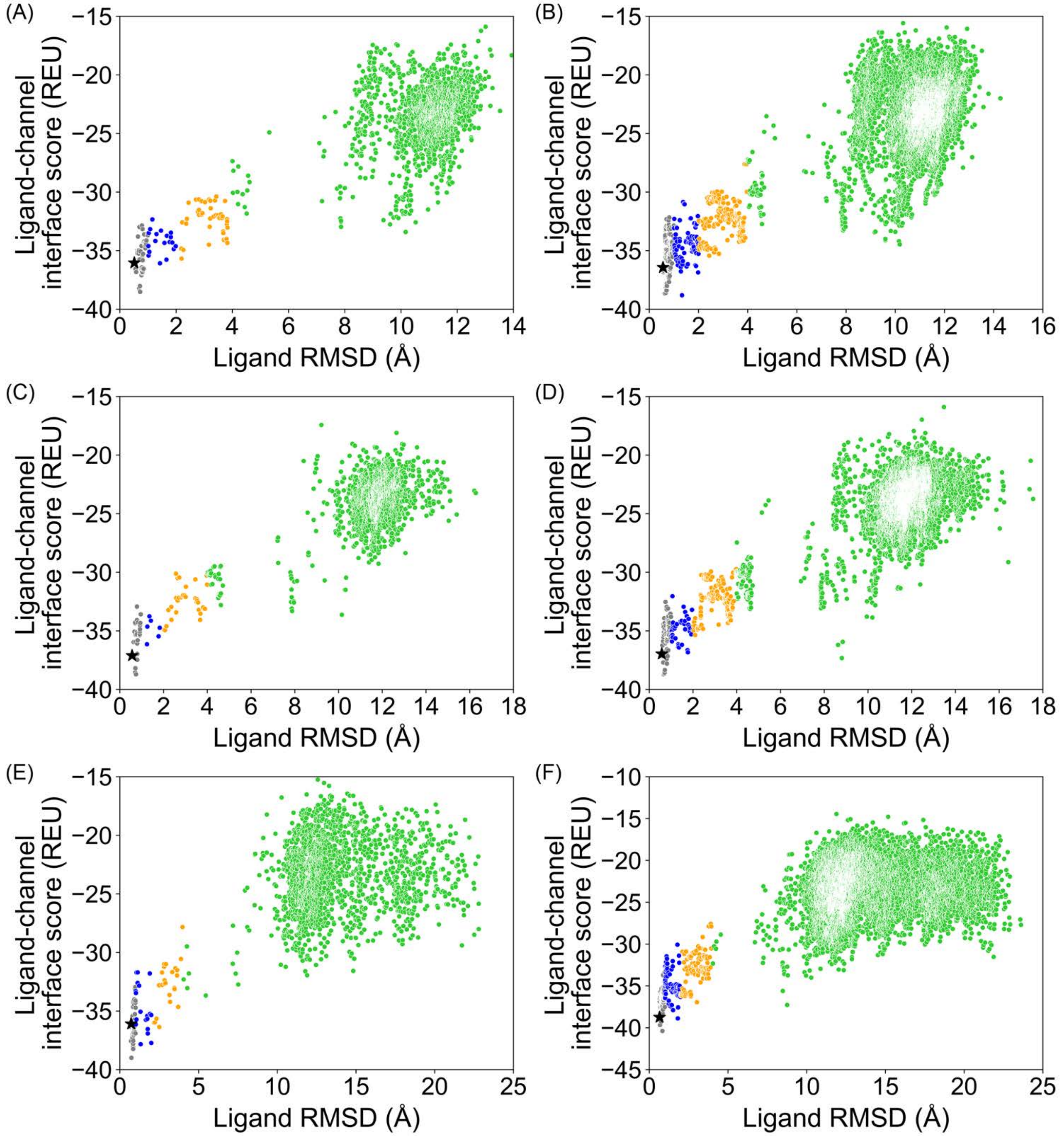

Figure S2.4 PDB 5EK0, GALigandDock, top 10 % of total poses by total energy (`total_score`). **(A)** 20,000 total poses, padding 2 Å, **(B)** 100,000 total poses, padding 2 Å, **(C)** 20,000 total poses, padding 4 Å, **(D)** 100,000 total poses, padding 4 Å, **(E)** 20,000 total poses, padding 7 Å, **(F)** 100,000 total poses, padding 7 Å.

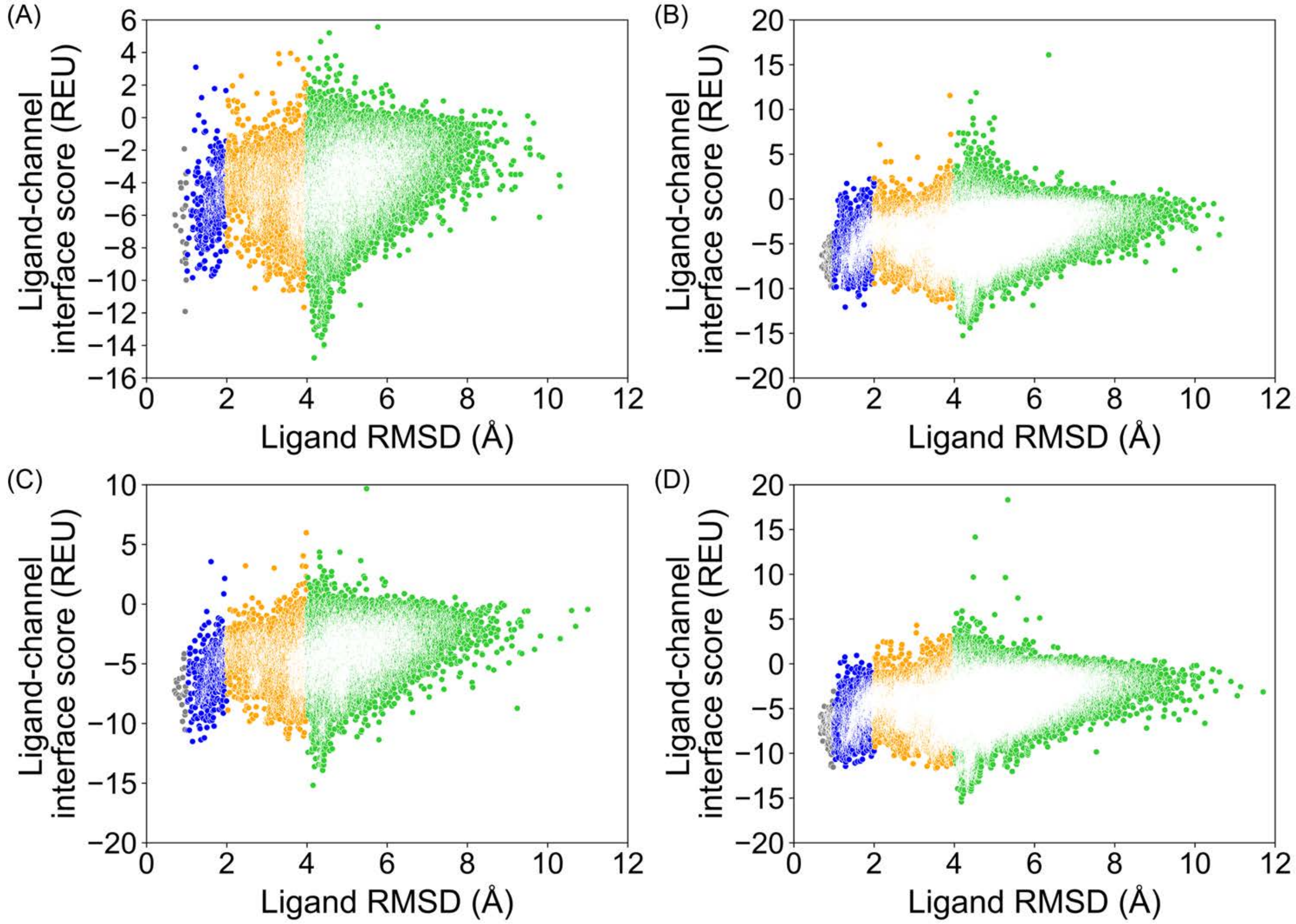

Figure S3.1 PDB 6J8G, RosettaLigand, all poses from docking. **(A)** 20,000 total poses, all ligand atom interface, **(B)** 100,000 total poses, all ligand atom interface, **(C)** 20,000 total poses, ligand neighbor atom interface, **(D)** 100,000 total poses, ligand neighbor atom interface.

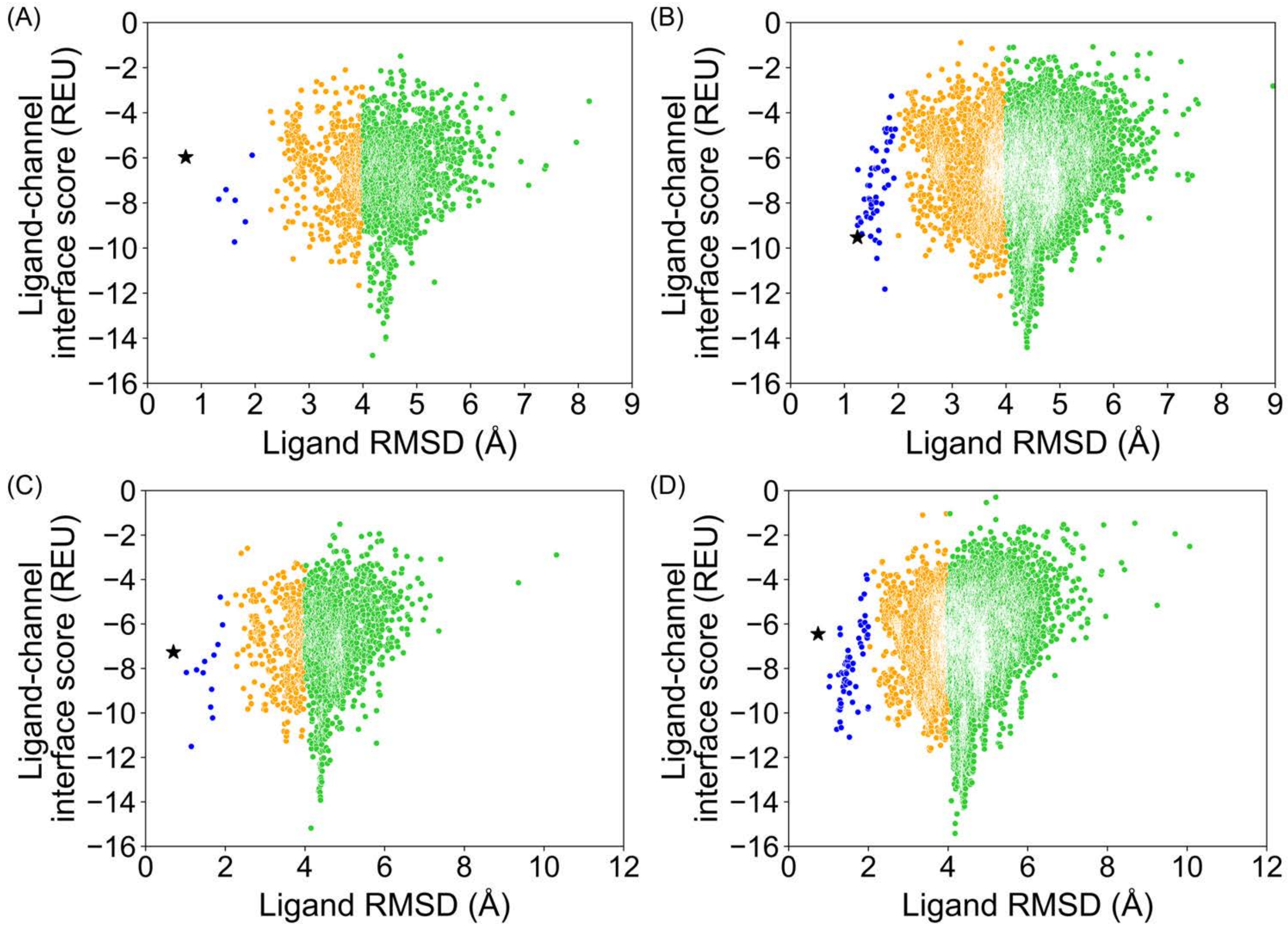

Figure S3.2 PDB 6J8G, RosettaLigand, top 10 % of total poses by total energy (`total_score`). **(A)** 20,000 total poses, all ligand atom interface, **(B)** 100,000 total poses, all ligand atom interface, **(C)** 20,000 total poses, ligand neighbor atom interface, **(D)** 100,000 total poses, ligand neighbor atom interface.

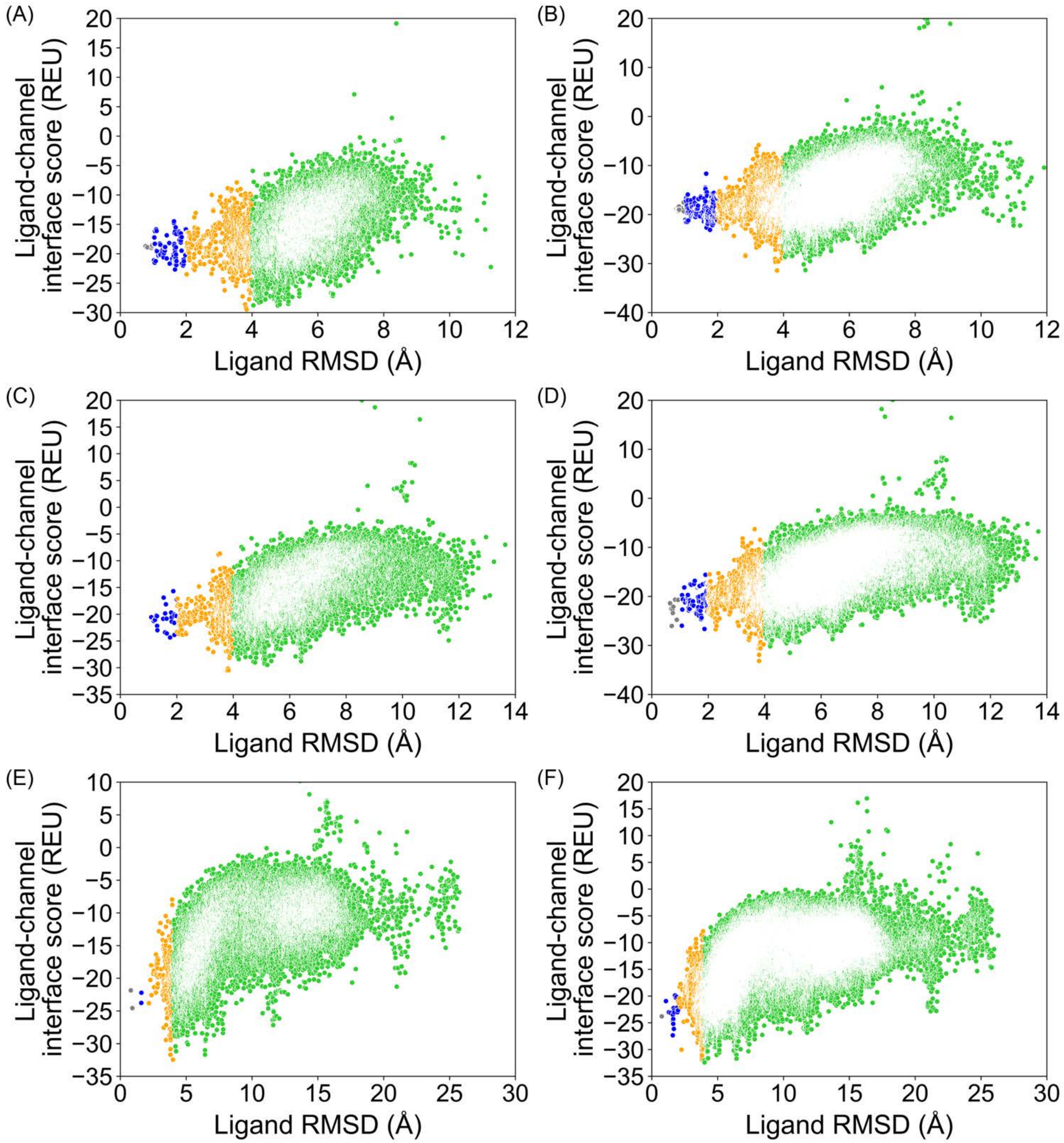

Figure S3.3 PDB 6J8G, GALigandDock, all poses from docking. **(A)** 20,000 total poses, padding 2 Å, **(B)** 100,000 total poses, padding 2 Å, **(C)** 20,000 total poses, padding 4 Å, **(D)** 100,000 total poses, padding 4 Å, **(E)** 20,000 total poses, padding 7 Å, **(F)** 100,000 total poses, padding 7 Å.

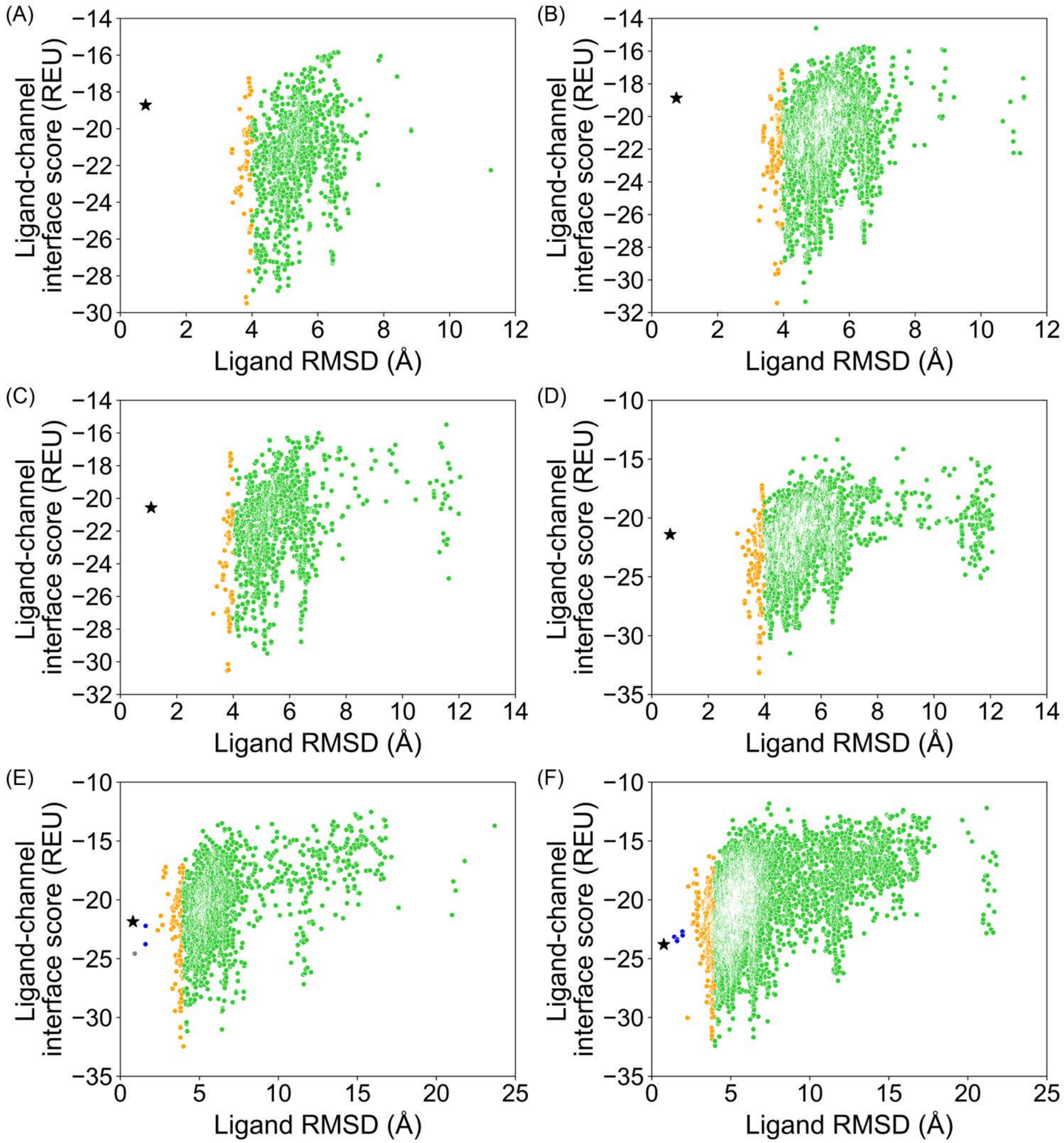

Figure S3.4 PDB 6J8G, GALigandDock, top 10 % of total poses by total energy (`total_score`). **(A)** 20,000 total poses, padding 2 Å, **(B)** 100,000 total poses, padding 2 Å, **(C)** 20,000 total poses, padding 4 Å, **(D)** 100,000 total poses, padding 4 Å, **(E)** 20,000 total poses, padding 7 Å, **(F)** 100,000 total poses, padding 7 Å.

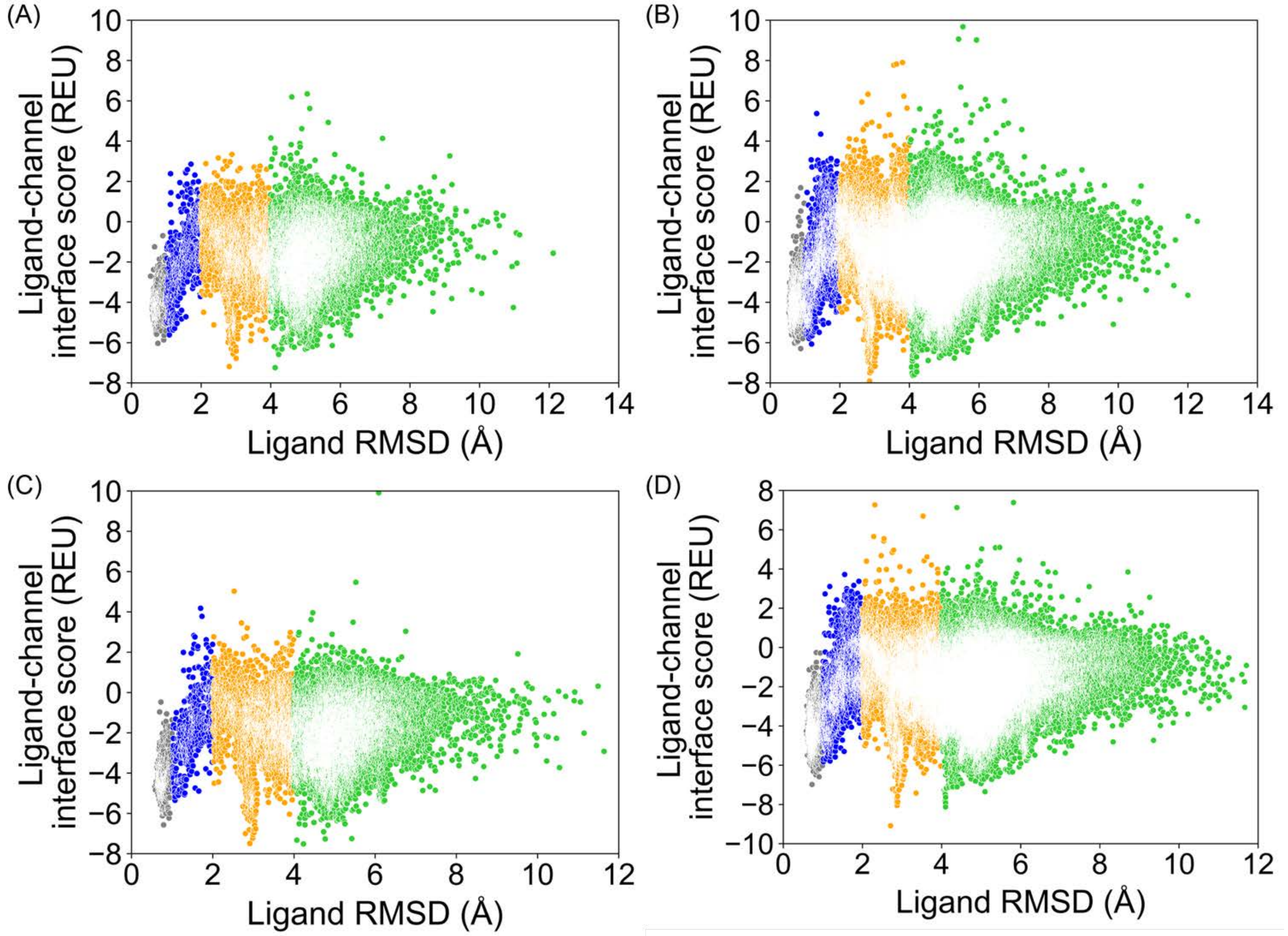

Figure S4.1 PDB 6J8I, RosettaLigand, all poses from docking. **(A)** 20,000 total poses, all ligand atom interface, **(B)** 100,000 total poses, all ligand atom interface, **(C)** 20,000 total poses, ligand neighbor atom interface, **(D)** 100,000 total poses, ligand neighbor atom interface.

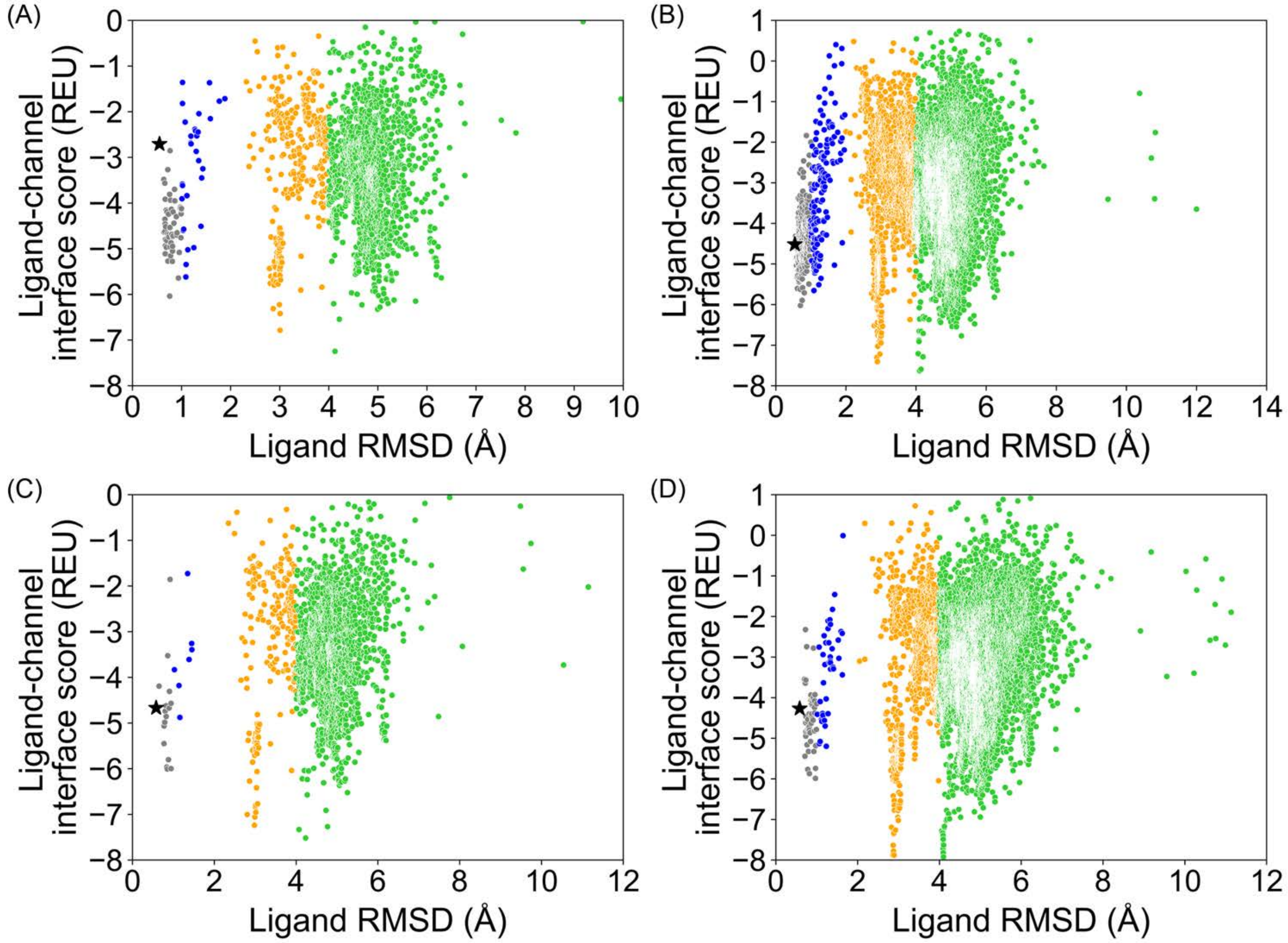

Figure S4.2 PDB 6J8I, RosettaLigand, top 10 % of total poses by total energy (`total_score`). **(A)** 20,000 total poses, all ligand atom interface, **(B)** 100,000 total poses, all ligand atom interface, **(C)** 20,000 total poses, ligand neighbor atom interface, **(D)** 100,000 total poses, ligand neighbor atom interface.

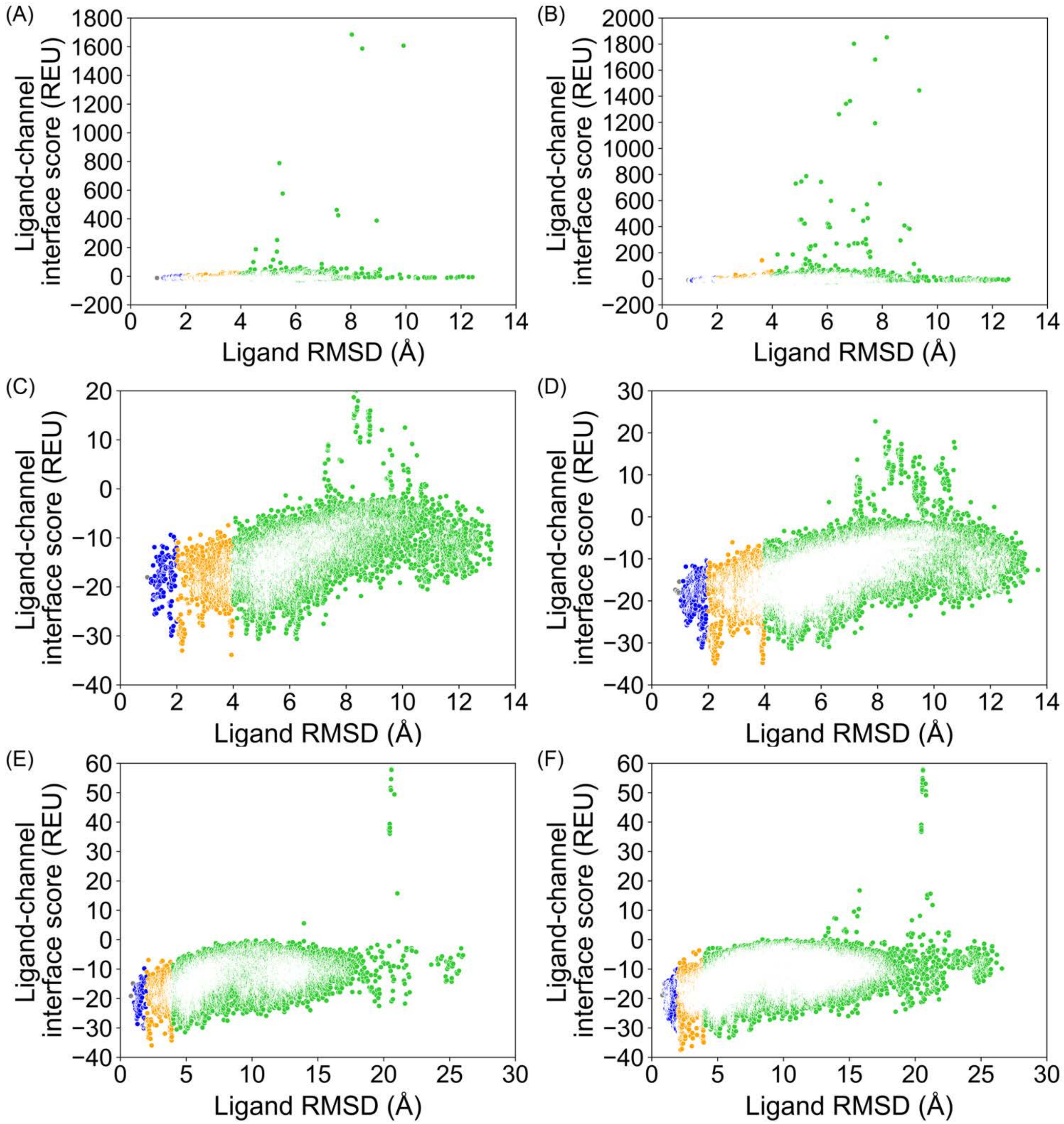

Figure S4.3 PDB 6J8I, GALigandDock, all poses from docking. **(A)** 20,000 total poses, padding 2 Å, **(B)** 100,000 total poses, padding 2 Å, **(C)** 20,000 total poses, padding 4 Å, **(D)** 100,000 total poses, padding 4 Å, **(E)** 20,000 total poses, padding 7 Å, **(F)** 100,000 total poses, padding 7 Å.

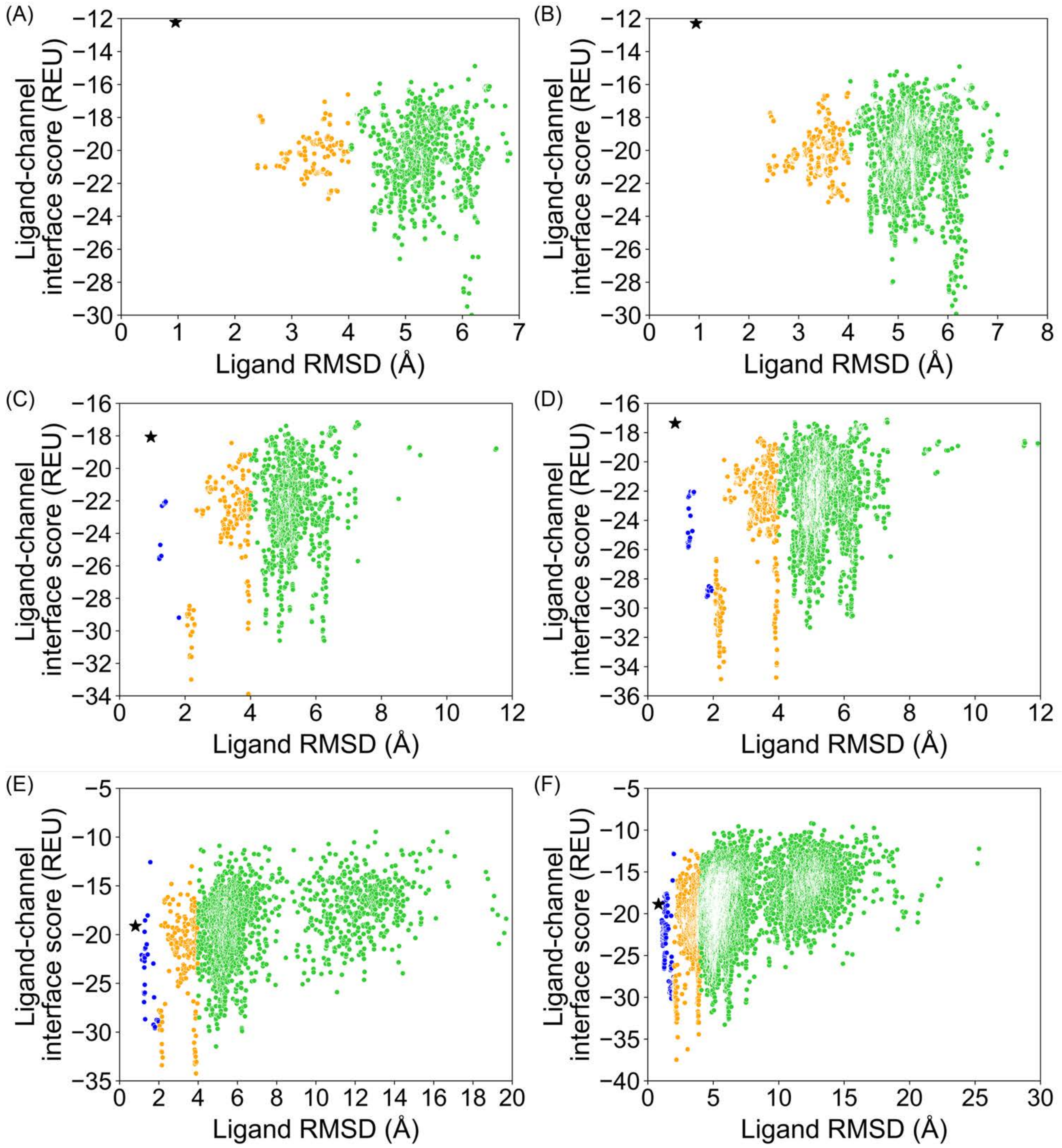

Figure S4.4 PDB 6J8I, GALigandDock, top 10 % of total poses by total energy (`total_score`). **(A)** 20,000 total poses, padding 2 Å, **(B)** 100,000 total poses, padding 2 Å, **(C)** 20,000 total poses, padding 4 Å, **(D)** 100,000 total poses, padding 4 Å, **(E)** 20,000 total poses, padding 7 Å, **(F)** 100,000 total poses, padding 7 Å.

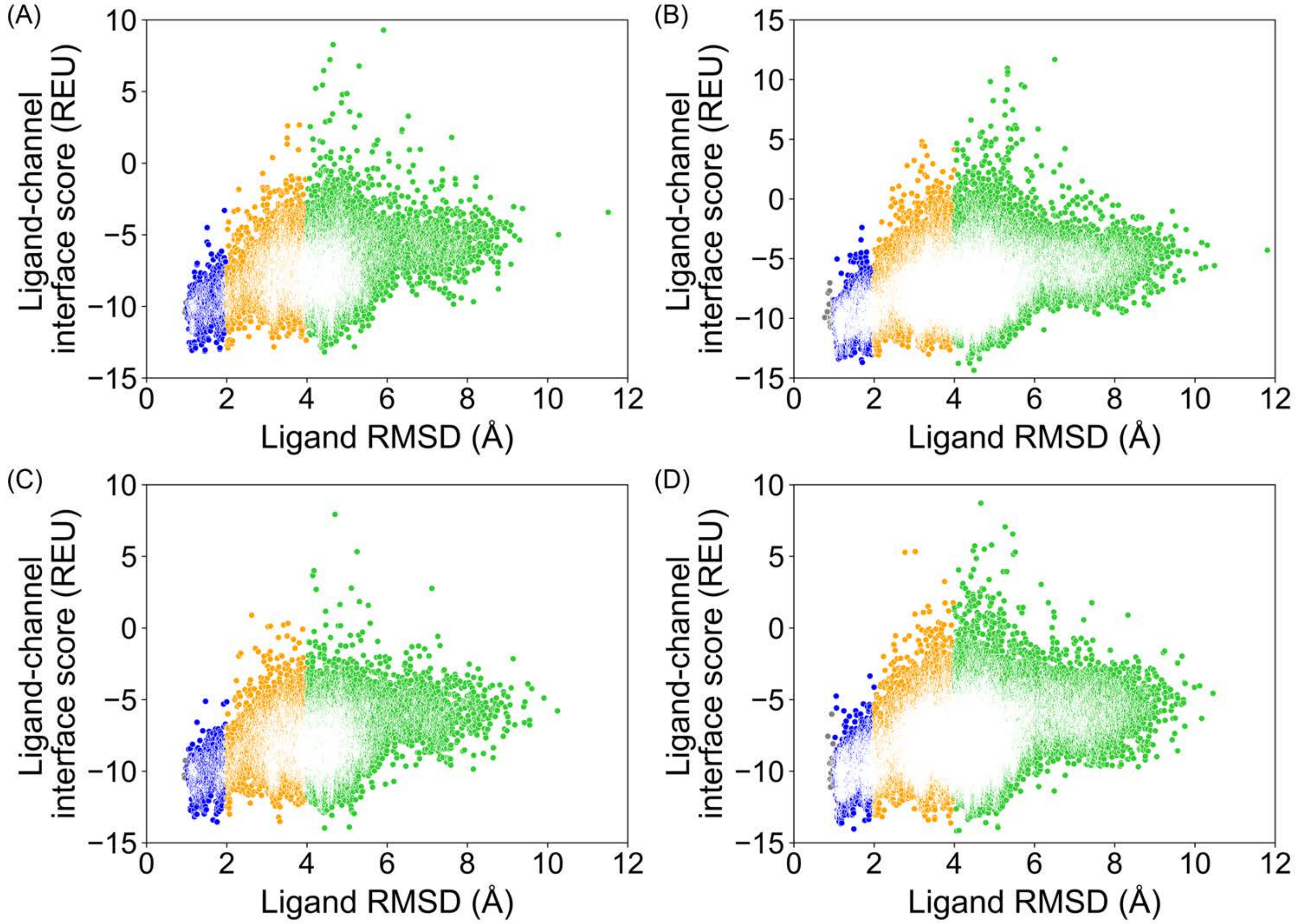

Figure S5.1 PDB 6JP5, RosettaLigand, all poses from docking. **(A)** 20,000 total poses, all ligand atom interface, **(B)** 100,000 total poses, all ligand atom interface, **(C)** 20,000 total poses, ligand neighbor atom interface, **(D)** 100,000 total poses, ligand neighbor atom interface.

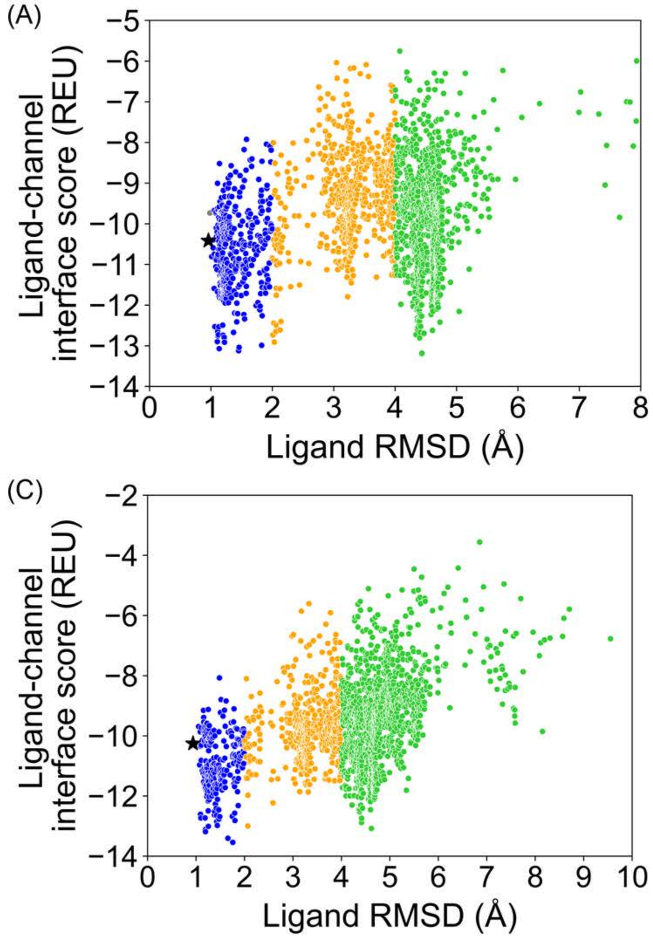

Figure S5.2 PDB 6JP5, RosettaLigand, top 10 % of total poses by total energy (`total_score`). **(A)** 20,000 total poses, all ligand atom interface, **(B)** 100,000 total poses, all ligand atom interface, **(C)** 20,000 total poses, ligand neighbor atom interface, **(D)** 100,000 total poses, ligand neighbor atom interface.

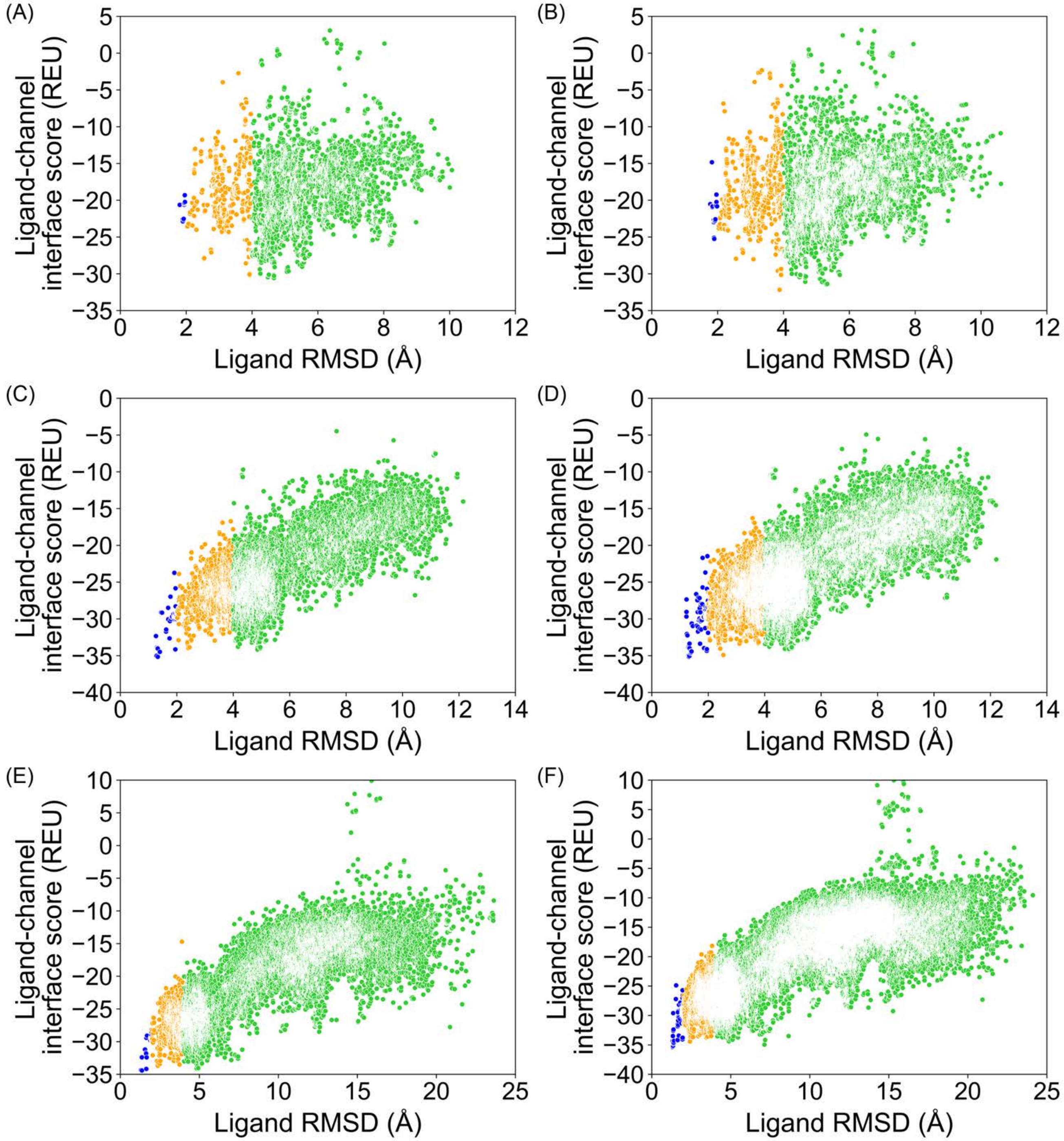

Figure S5.3 PDB 6JP5, GALigandDock, all poses from docking. **(A)** 20,000 total poses, padding 2 Å, **(B)** 100,000 total poses, padding 2 Å, **(C)** 20,000 total poses, padding 4 Å, **(D)** 100,000 total poses, padding 4 Å, **(E)** 20,000 total poses, padding 7 Å, **(F)** 100,000 total poses, padding 7 Å.

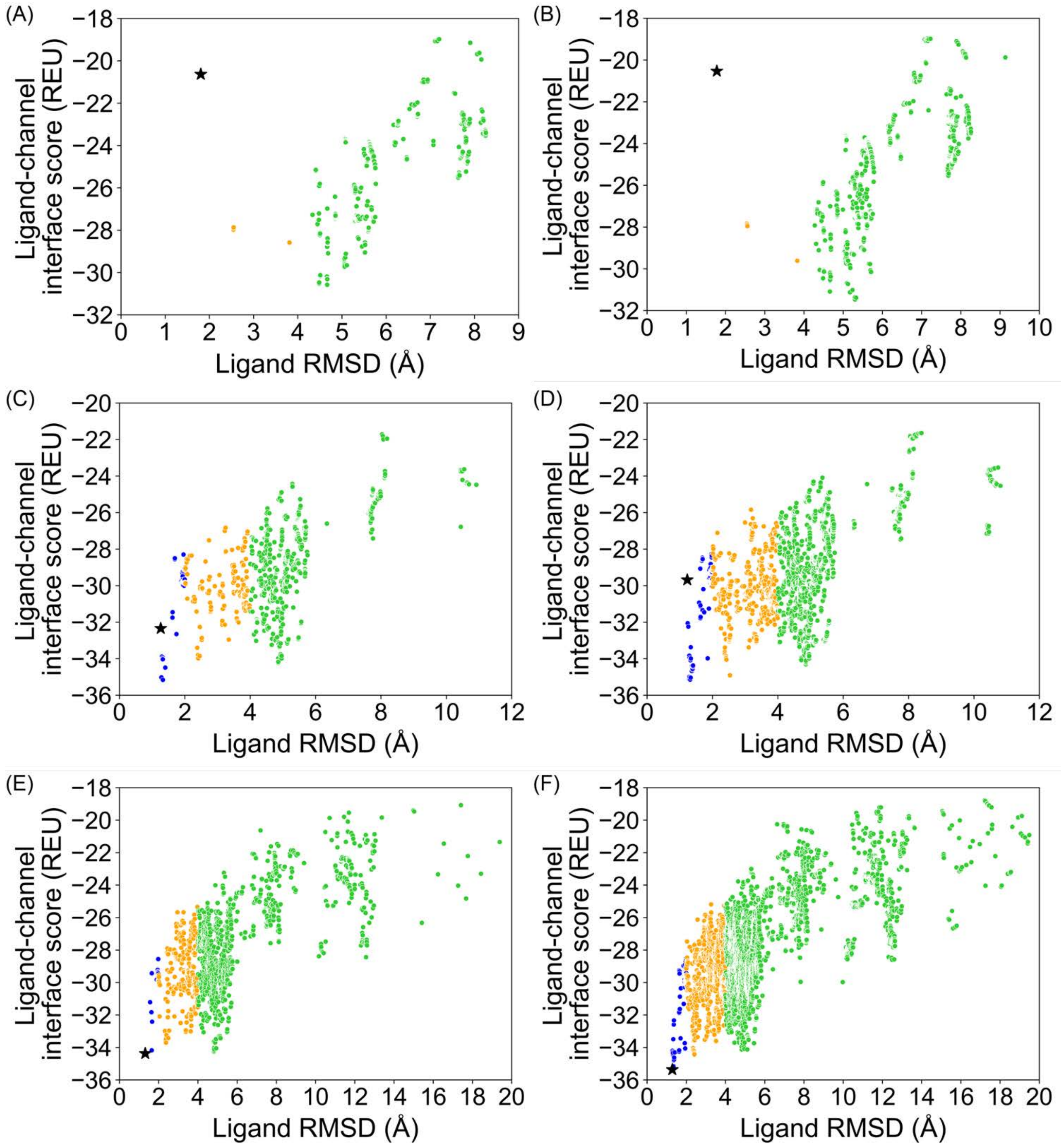

Figure S5.4 PDB 6JP5, GALigandDock, top 10 % of total poses by total energy (`total_score`). **(A)** 20,000 total poses, padding 2 Å, **(B)** 100,000 total poses, padding 2 Å, **(C)** 20,000 total poses, padding 4 Å, **(D)** 100,000 total poses, padding 4 Å, **(E)** 20,000 total poses, padding 7 Å, **(F)** 100,000 total poses, padding 7 Å.

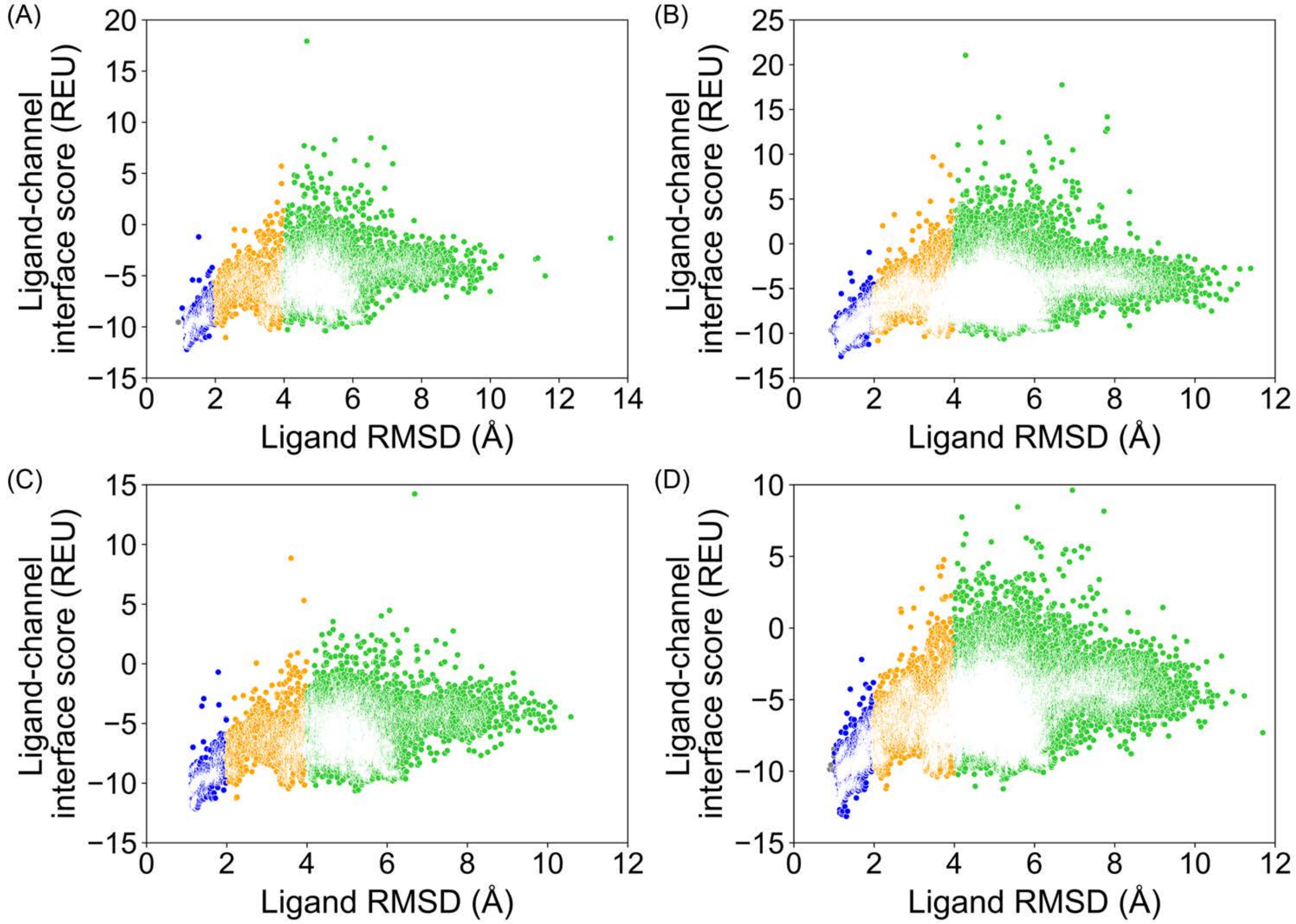

Figure S6.1 PDB 6JP8, RosettaLigand, all poses from docking. **(A)** 20,000 total poses, all ligand atom interface, **(B)** 100,000 total poses, all ligand atom interface, **(C)** 20,000 total poses, ligand neighbor atom interface, **(D)** 100,000 total poses, ligand neighbor atom interface.

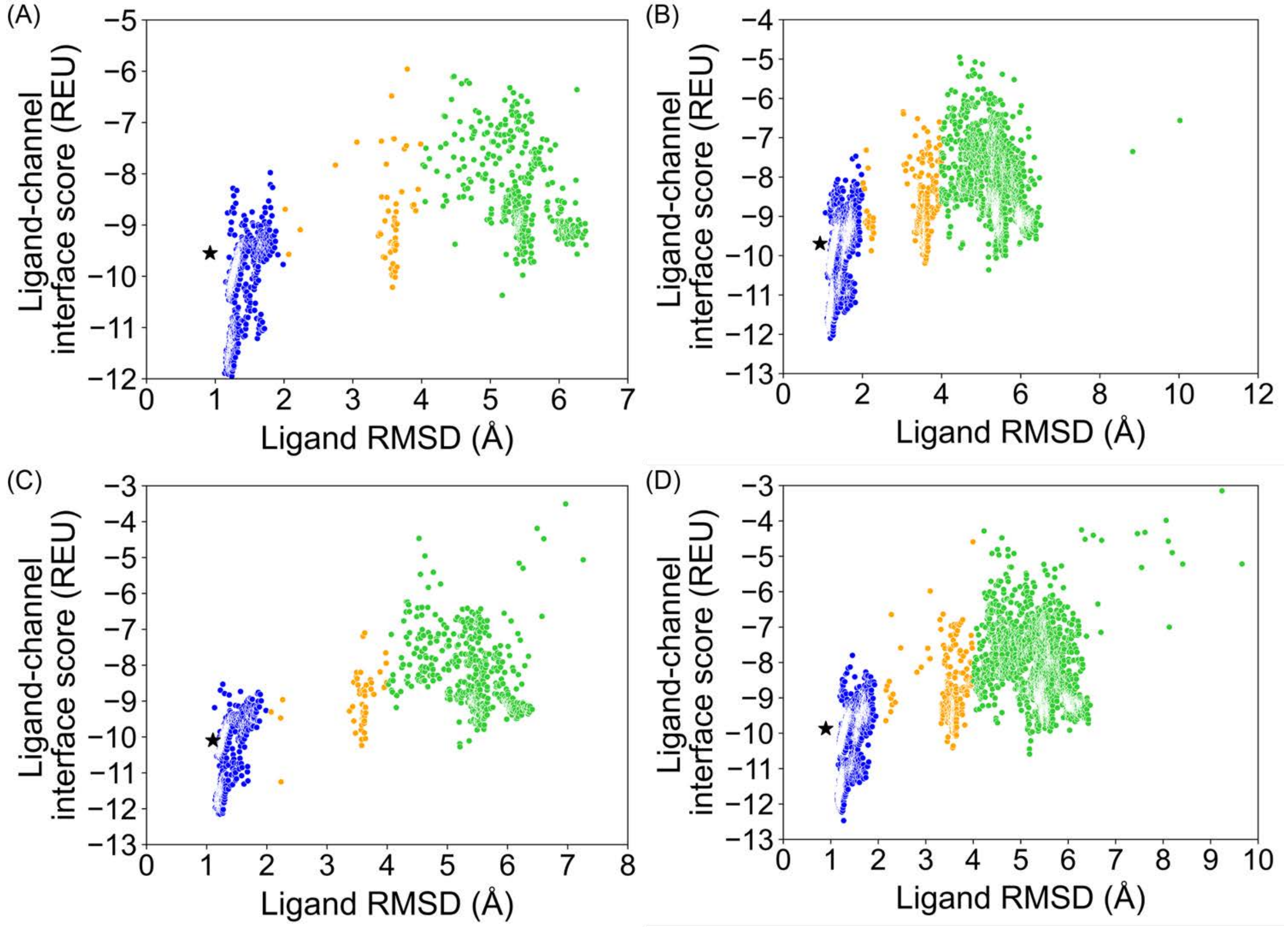

Figure S6.2 PDB 6JP8, RosettaLigand, top 10 % of total poses by total energy (`total_score`). **(A)** 20,000 total poses, all ligand atom interface, **(B)** 100,000 total poses, all ligand atom interface, **(C)** 20,000 total poses, ligand neighbor atom interface, **(D)** 100,000 total poses, ligand neighbor atom interface.

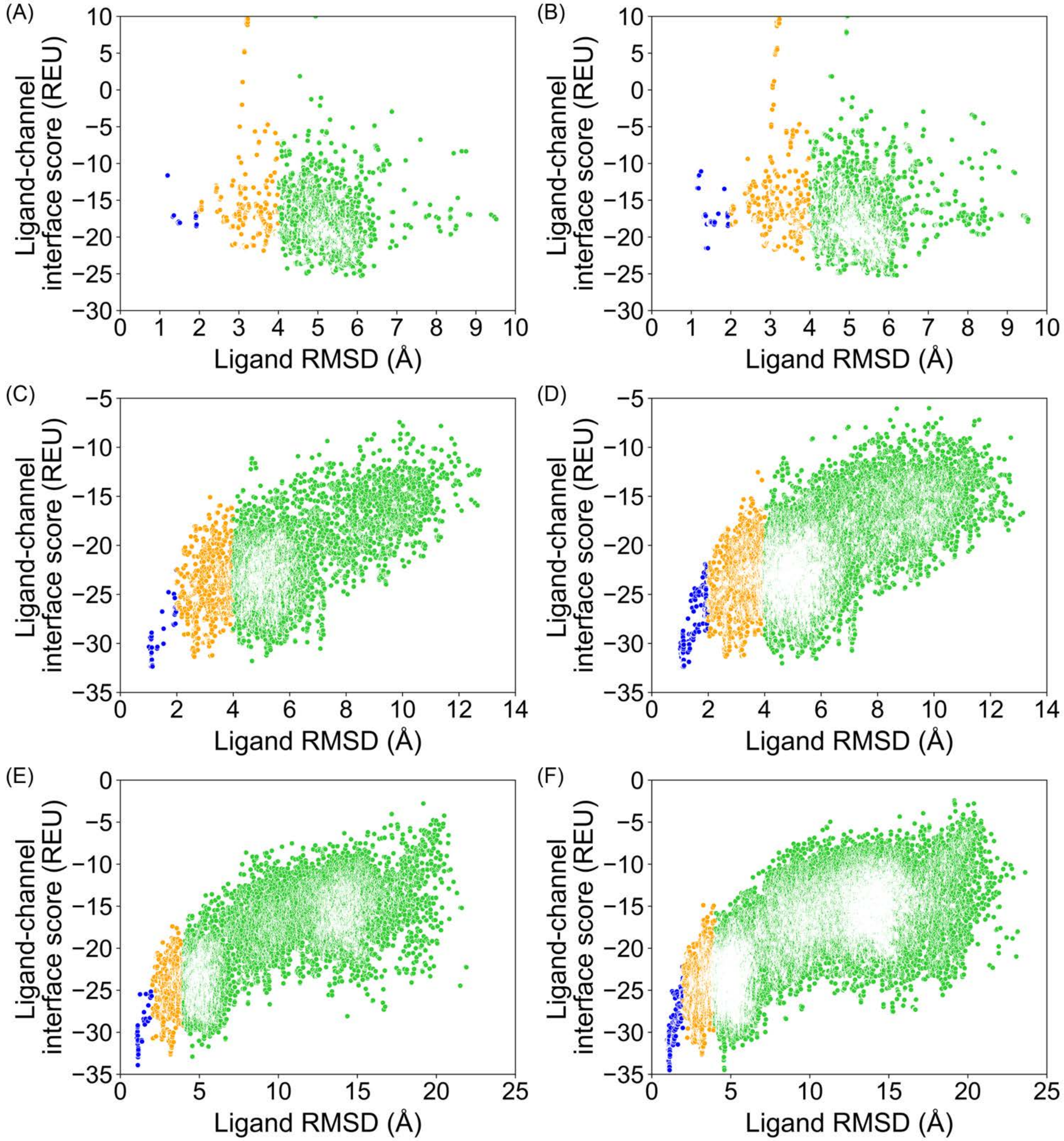

Figure S6.3 PDB 6JP8, GALigandDock, all poses from docking. **(A)** 20,000 total poses, padding 2 Å, **(B)** 100,000 total poses, padding 2 Å, **(C)** 20,000 total poses, padding 4 Å, **(D)** 100,000 total poses, padding 4 Å, **(E)** 20,000 total poses, padding 7 Å, **(F)** 100,000 total poses, padding 7 Å.

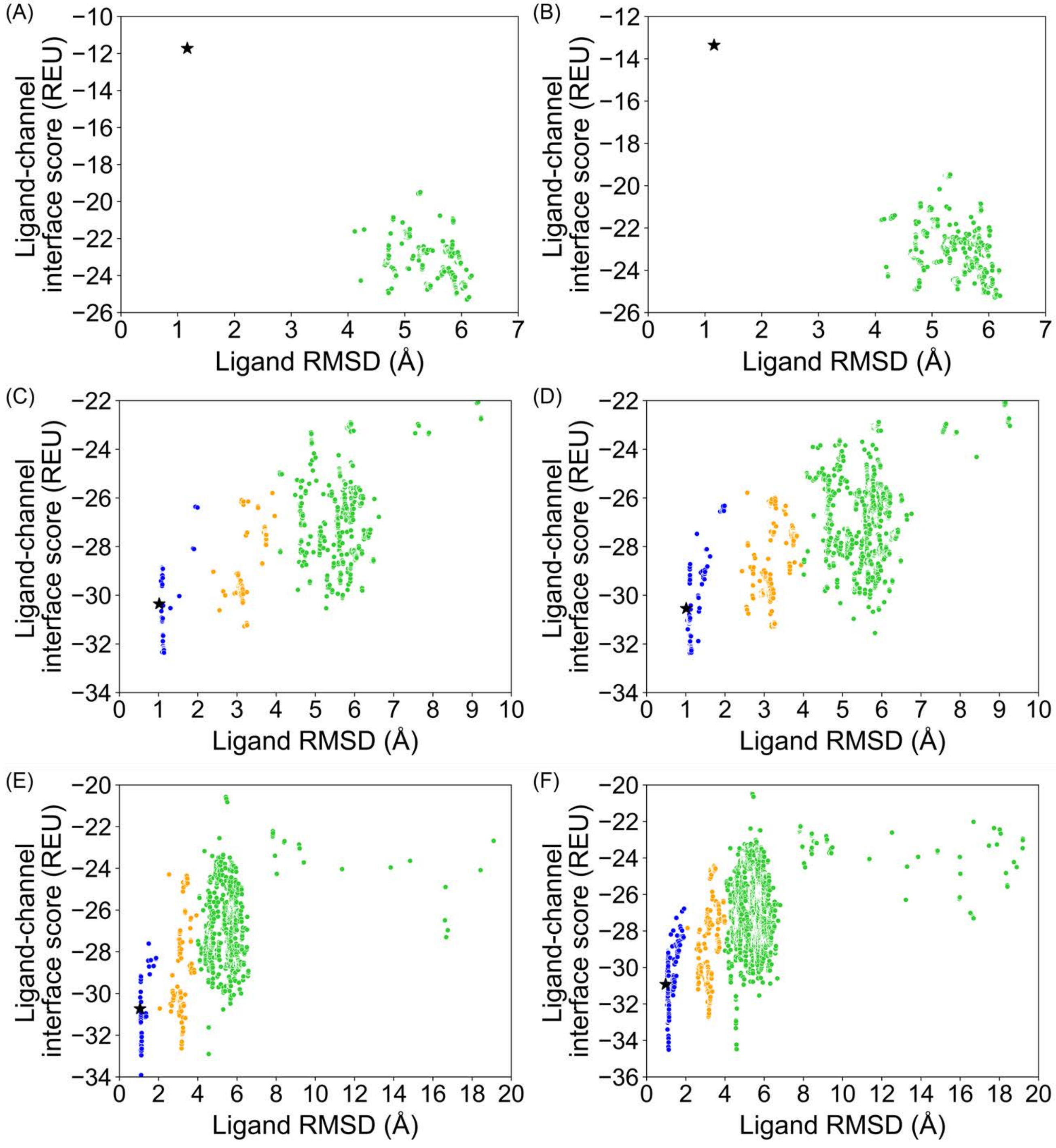

Figure S6.4 PDB 6JP8, GALigandDock, top 10 % of total poses by total energy (`total_score`). **(A)** 20,000 total poses, padding 2 Å, **(B)** 100,000 total poses, padding 2 Å, **(C)** 20,000 total poses, padding 4 Å, **(D)** 100,000 total poses, padding 4 Å, **(E)** 20,000 total poses, padding 7 Å, **(F)** 100,000 total poses, padding 7 Å.

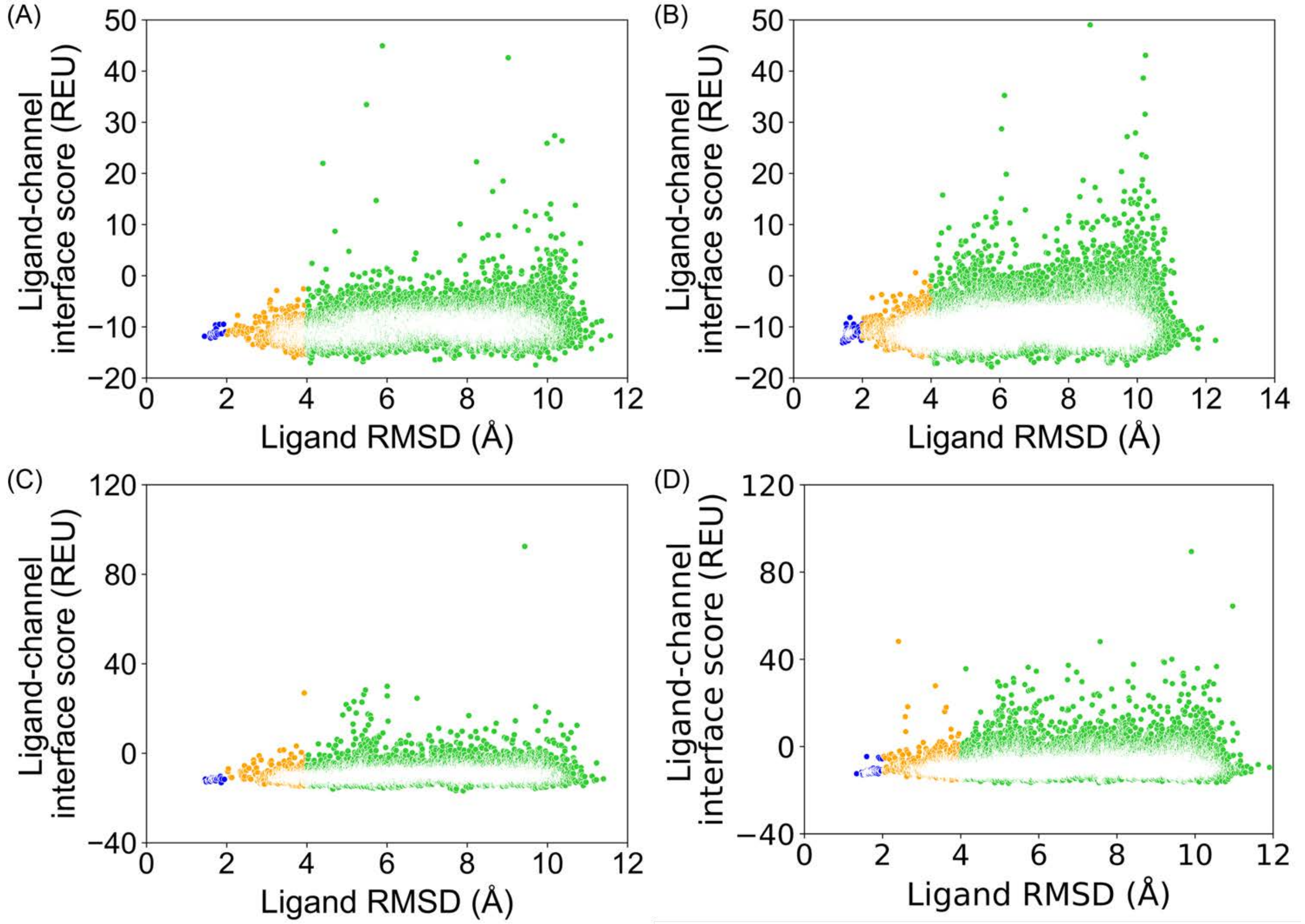

Figure S7.1 PDB 6JPAV1, RosettaLigand, all poses from docking. **(A)** 20,000 total poses, all ligand atom interface, **(B)** 100,000 total poses, all ligand atom interface, **(C)** 20,000 total poses, ligand neighbor atom interface, **(D)** 100,000 total poses, ligand neighbor atom interface.

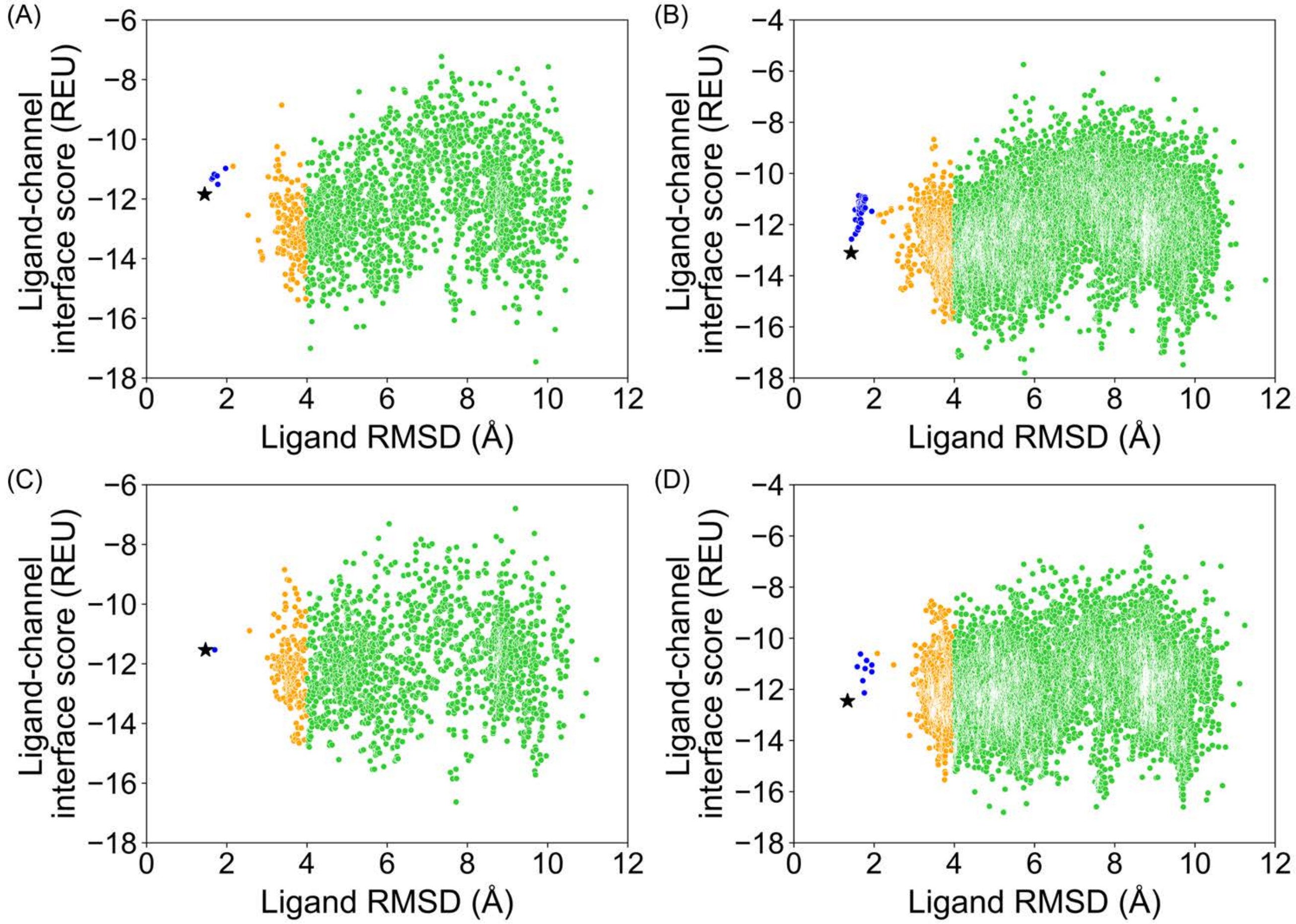

Figure S7.2 PDB 6JPAV1, RosettaLigand, top 10 % of total poses by total energy (`total_score`). **(A)** 20,000 total poses, all ligand atom interface, **(B)** 100,000 total poses, all ligand atom interface, **(C)** 20,000 total poses, ligand neighbor atom interface, **(D)** 100,000 total poses, ligand neighbor atom interface.

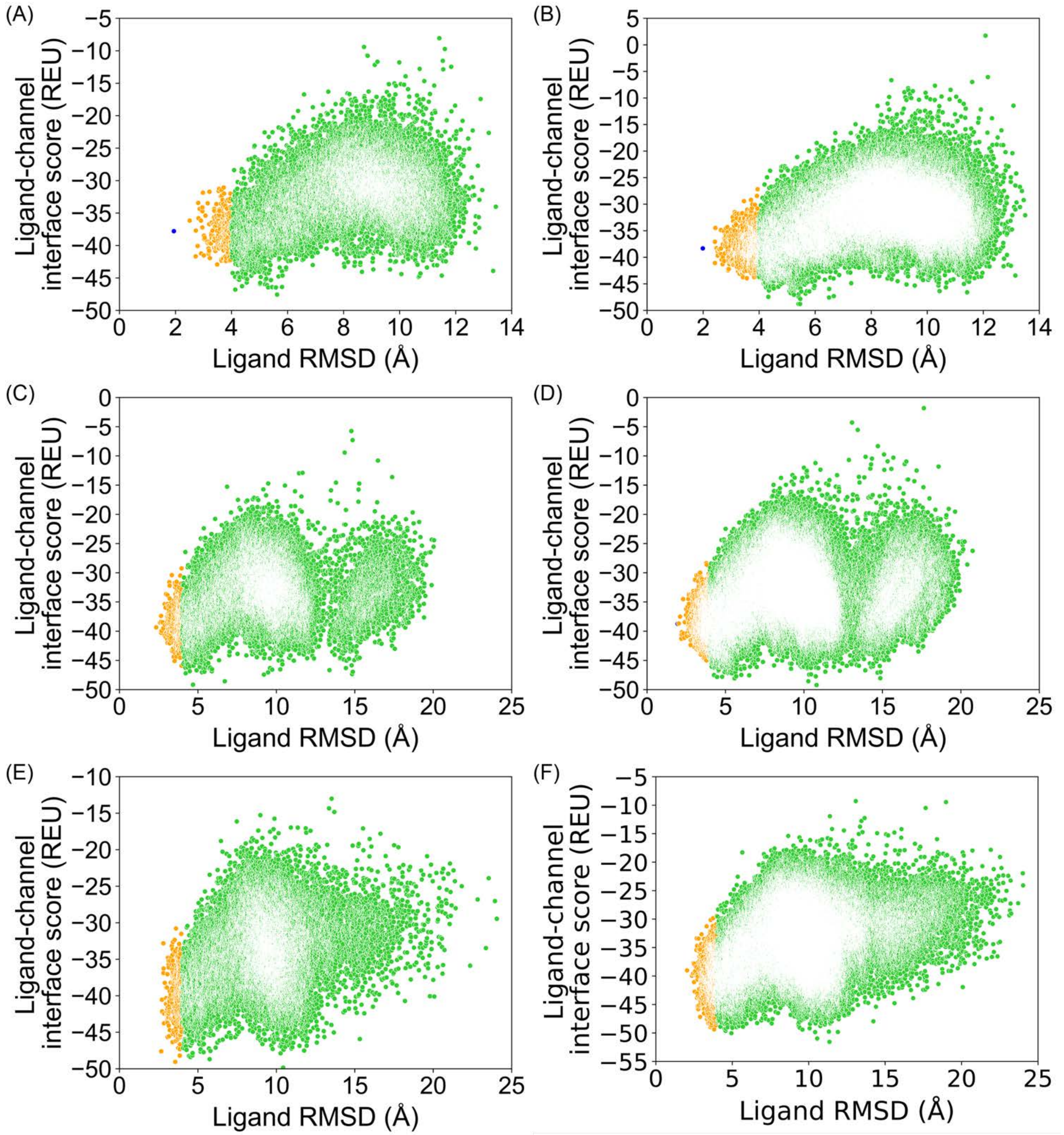

Figure S7.3 PDB 6JPAV1, GALigandDock, all poses from docking. **(A)** 20,000 total poses, padding 2 Å, **(B)** 100,000 total poses, padding 2 Å, **(C)** 20,000 total poses, padding 4 Å, **(D)** 100,000 total poses, padding 4 Å, **(E)** 20,000 total poses, padding 7 Å, **(F)** 100,000 total poses, padding 7 Å.

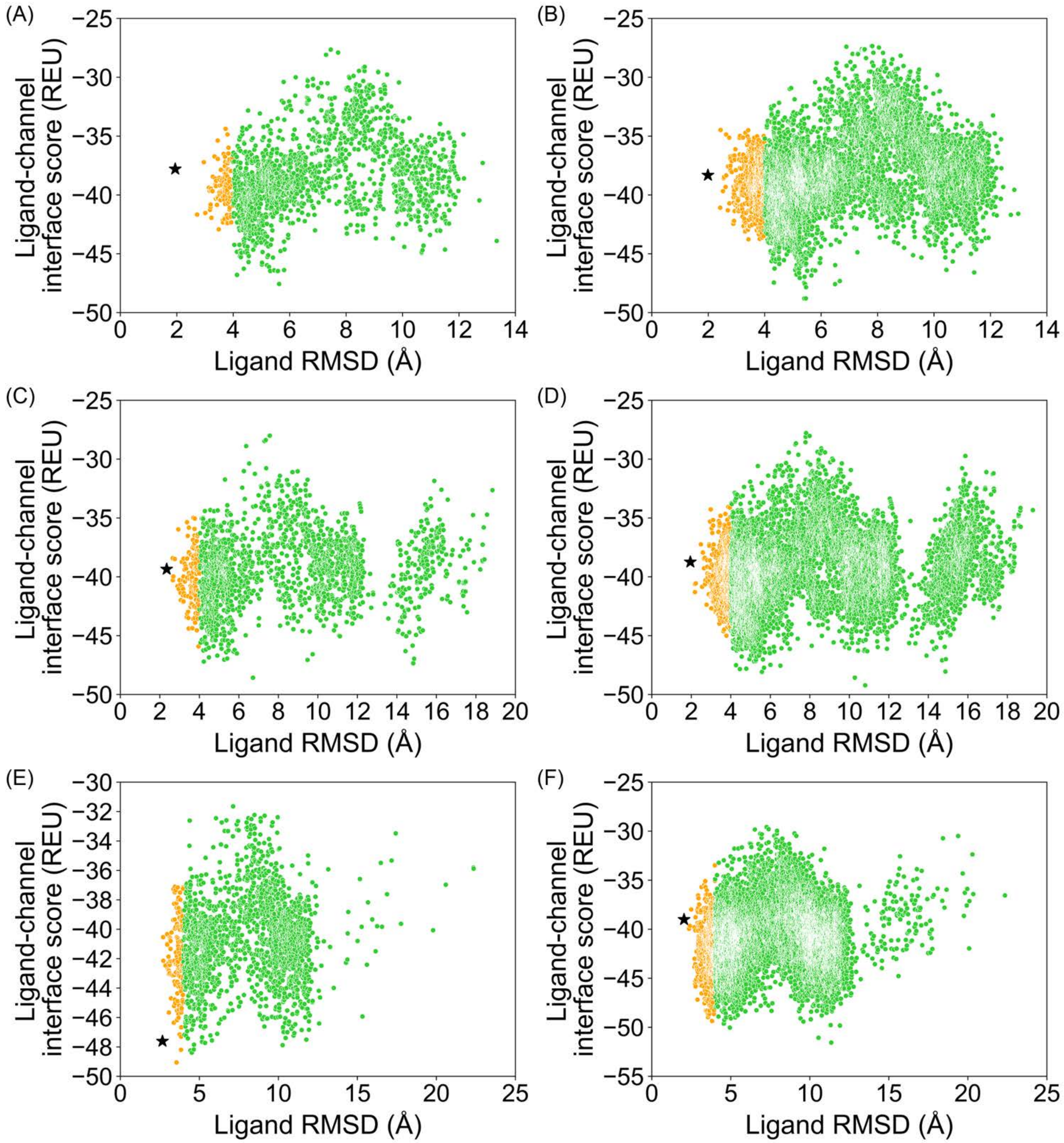

Figure S7.4 PDB 6JPAV1, GALigandDock, top 10 % of total poses by total energy (`total_score`). **(A)** 20,000 total poses, padding 2 Å, **(B)** 100,000 total poses, padding 2 Å, **(C)** 20,000 total poses, padding 4 Å, **(D)** 100,000 total poses, padding 4 Å, **(E)** 20,000 total poses, padding 7 Å, **(F)** 100,000 total poses, padding 7 Å.

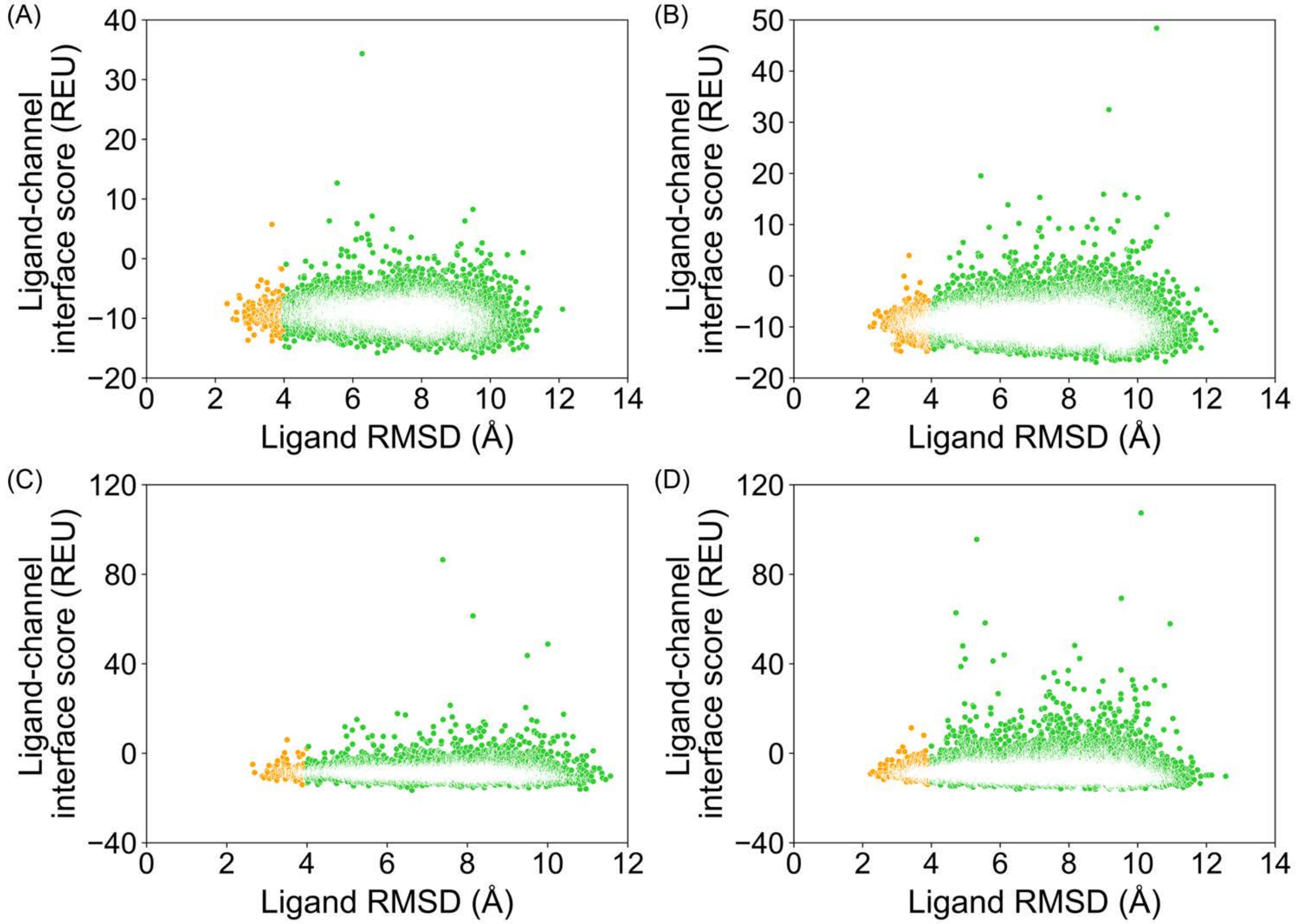

Figure S8.1 PDB 6JPAV2, RosettaLigand, all poses from docking. **(A)** 20,000 total poses, all ligand atom interface, **(B)** 100,000 total poses, all ligand atom interface, **(C)** 20,000 total poses, ligand neighbor atom interface, **(D)** 100,000 total poses, ligand neighbor atom interface.

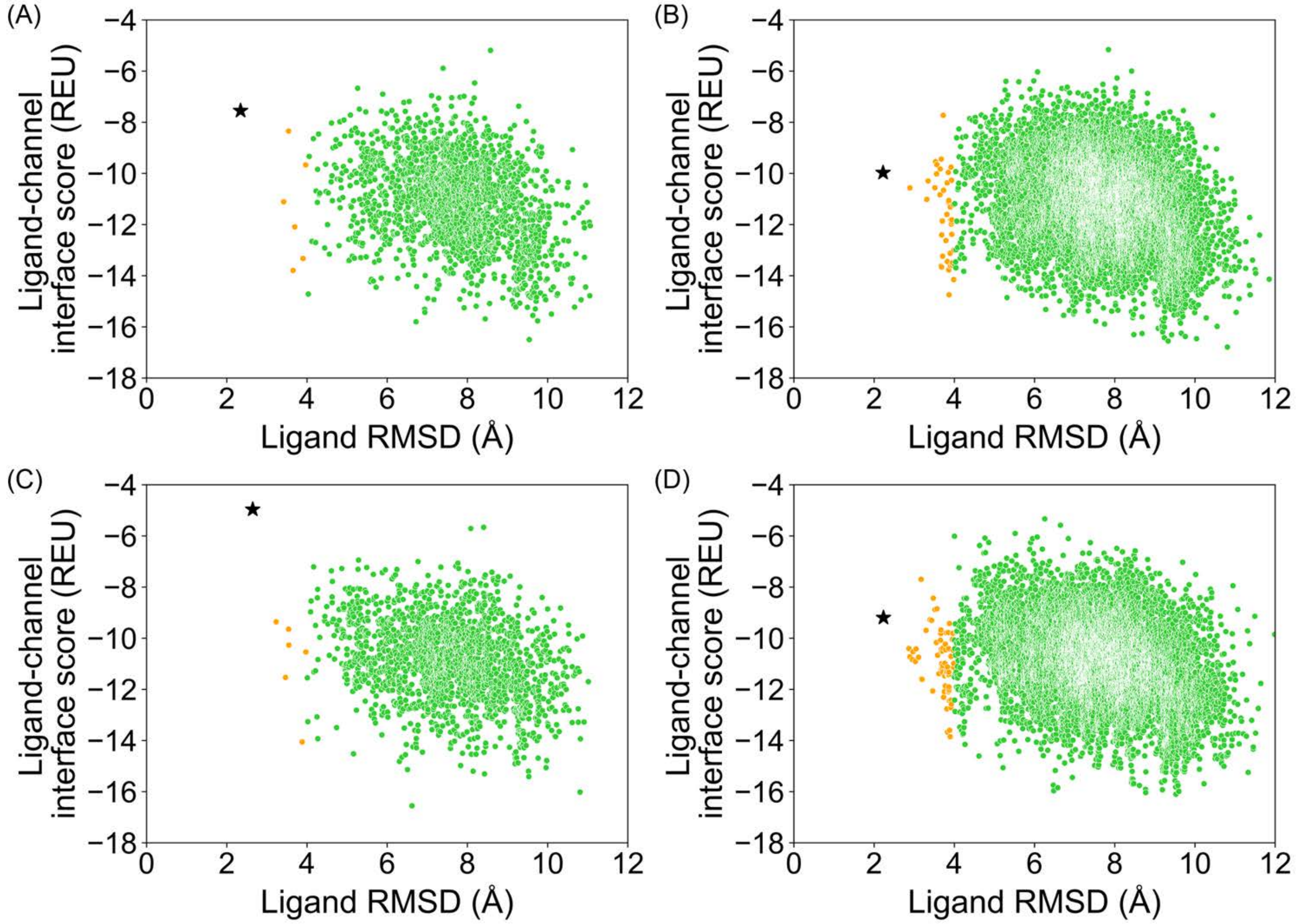

Figure S8.2 PDB 6JPAV2, RosettaLigand, top 10 % of total poses by total energy (`total_score`). **(A)** 20,000 total poses, all ligand atom interface, **(B)** 100,000 total poses, all ligand atom interface, **(C)** 20,000 total poses, ligand neighbor atom interface, **(D)** 100,000 total poses, ligand neighbor atom interface.

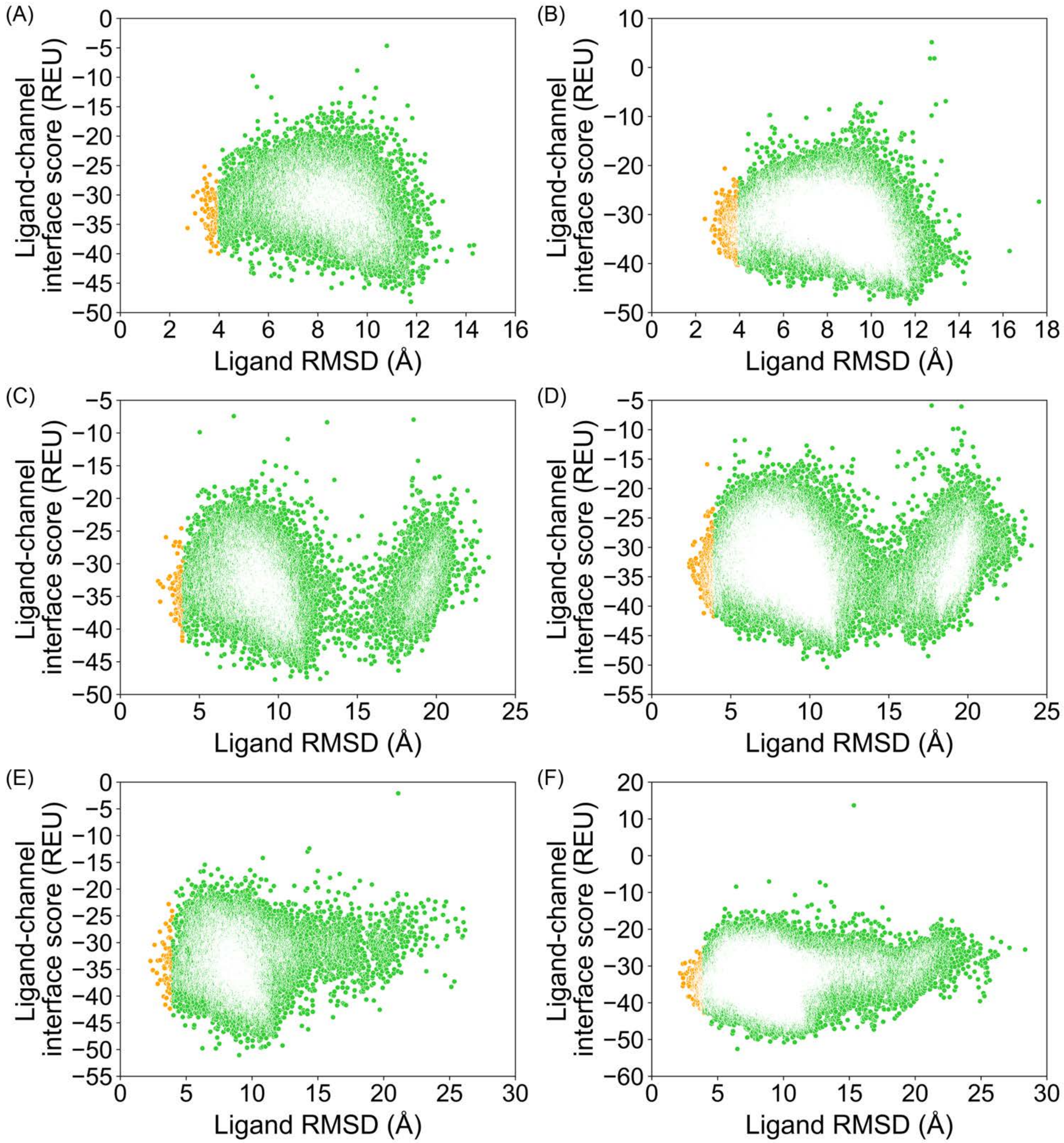

Figure S8.3 PDB 6JPAV2, GALigandDock, all poses from docking. **(A)** 20,000 total poses, padding 2 Å, **(B)** 100,000 total poses, padding 2 Å, **(C)** 20,000 total poses, padding 4 Å, **(D)** 100,000 total poses, padding 4 Å, **(E)** 20,000 total poses, padding 7 Å, **(F)** 100,000 total poses, padding 7 Å.

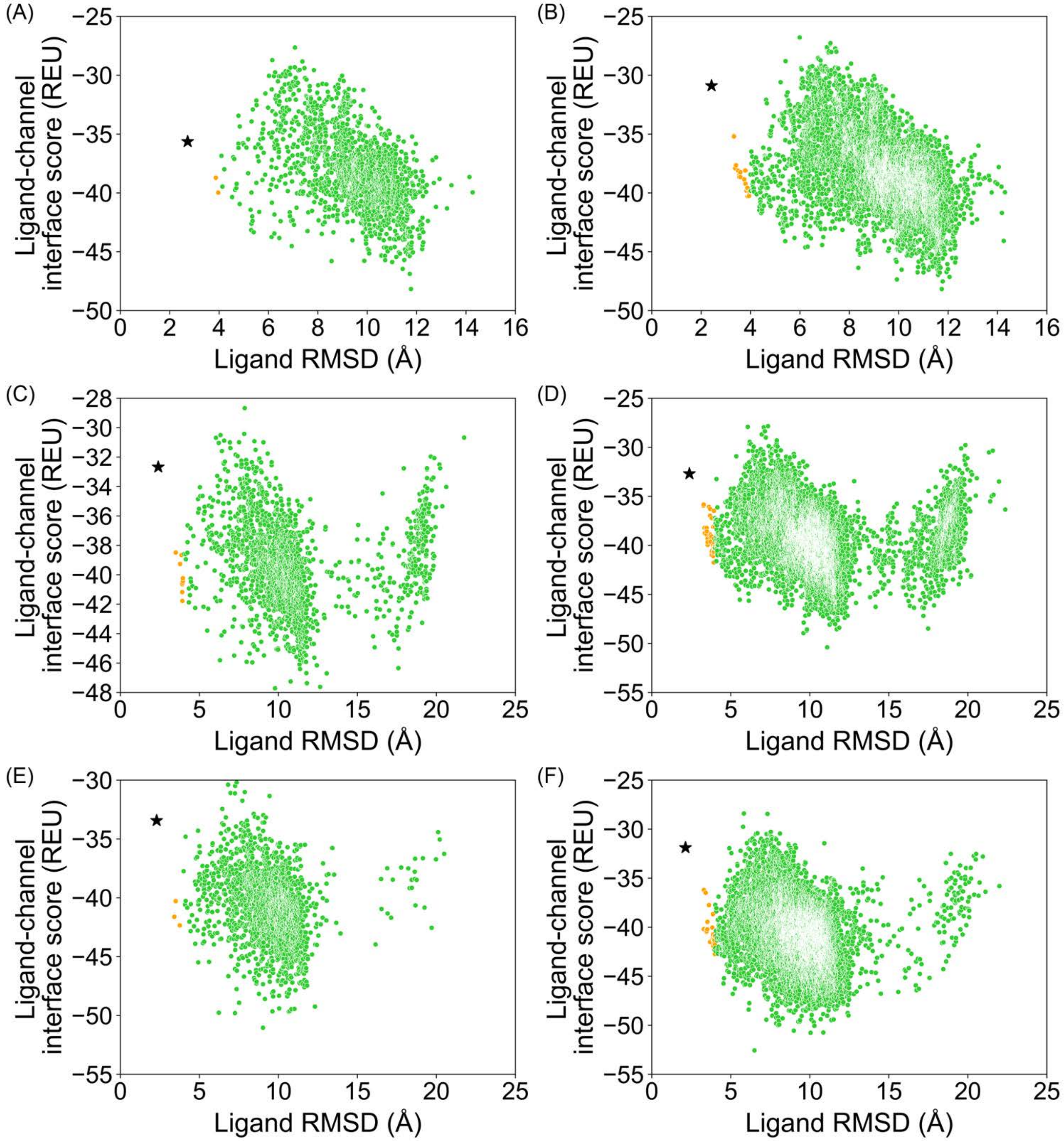

Figure S8.4 PDB 6JPAV2, GALigandDock, top 10 % of total poses by total energy (`total_score`). **(A)** 20,000 total poses, padding 2 Å, **(B)** 100,000 total poses, padding 2 Å, **(C)** 20,000 total poses, padding 4 Å, **(D)** 100,000 total poses, padding 4 Å, **(E)** 20,000 total poses, padding 7 Å, **(F)** 100,000 total poses, padding 7 Å.

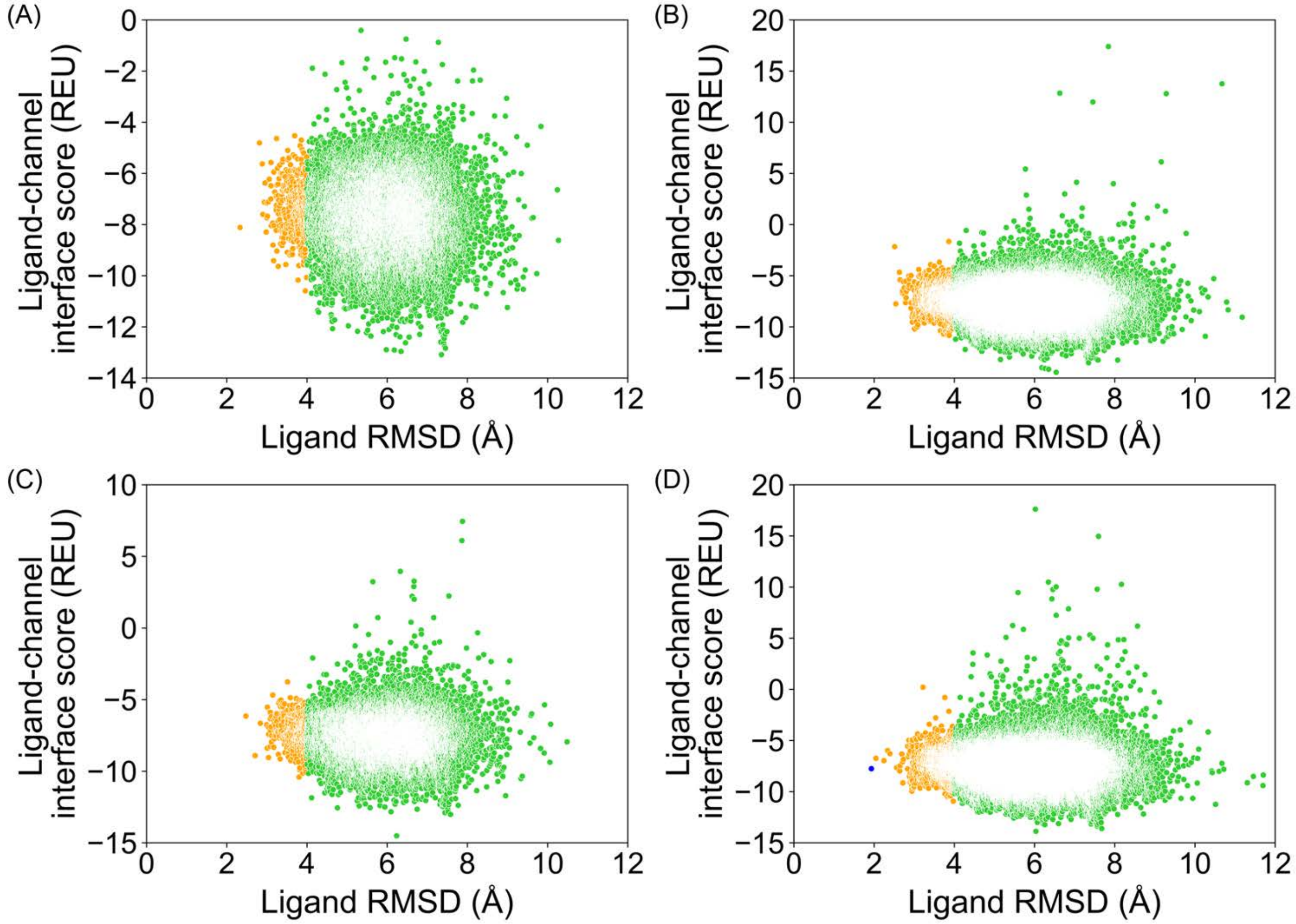

Figure S9.1 PDB 6JPB, RosettaLigand, all poses from docking. **(A)** 20,000 total poses, all ligand atom interface, **(B)** 100,000 total poses, all ligand atom interface, **(C)** 20,000 total poses, ligand neighbor atom interface, **(D)** 100,000 total poses, ligand neighbor atom interface.

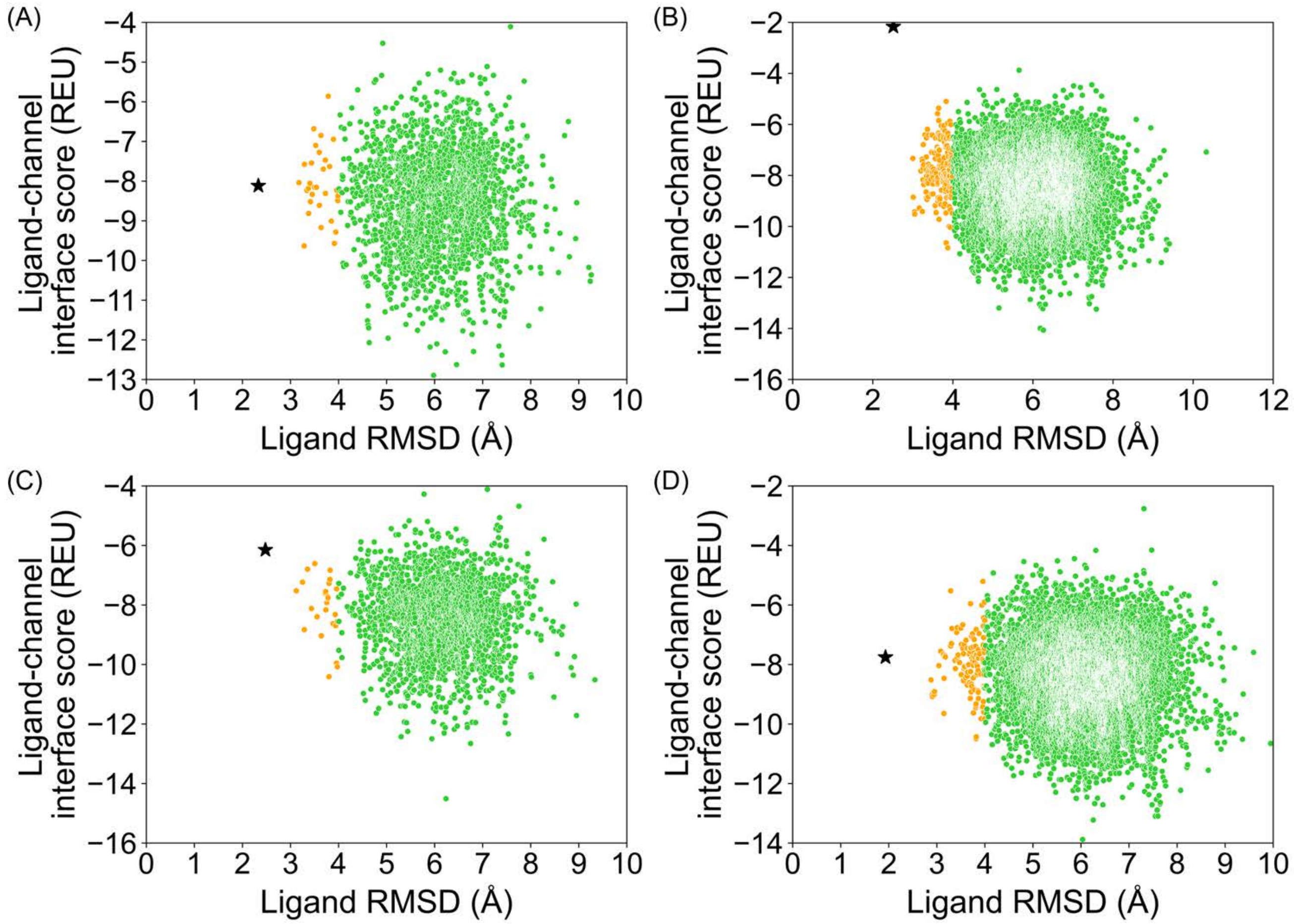

Figure S9.2 PDB 6JPB, RosettaLigand, top 10 % of total poses by total energy (`total_score`). **(A)** 20,000 total poses, all ligand atom interface, **(B)** 100,000 total poses, all ligand atom interface, **(C)** 20,000 total poses, ligand neighbor atom interface, **(D)** 100,000 total poses, ligand neighbor atom interface.

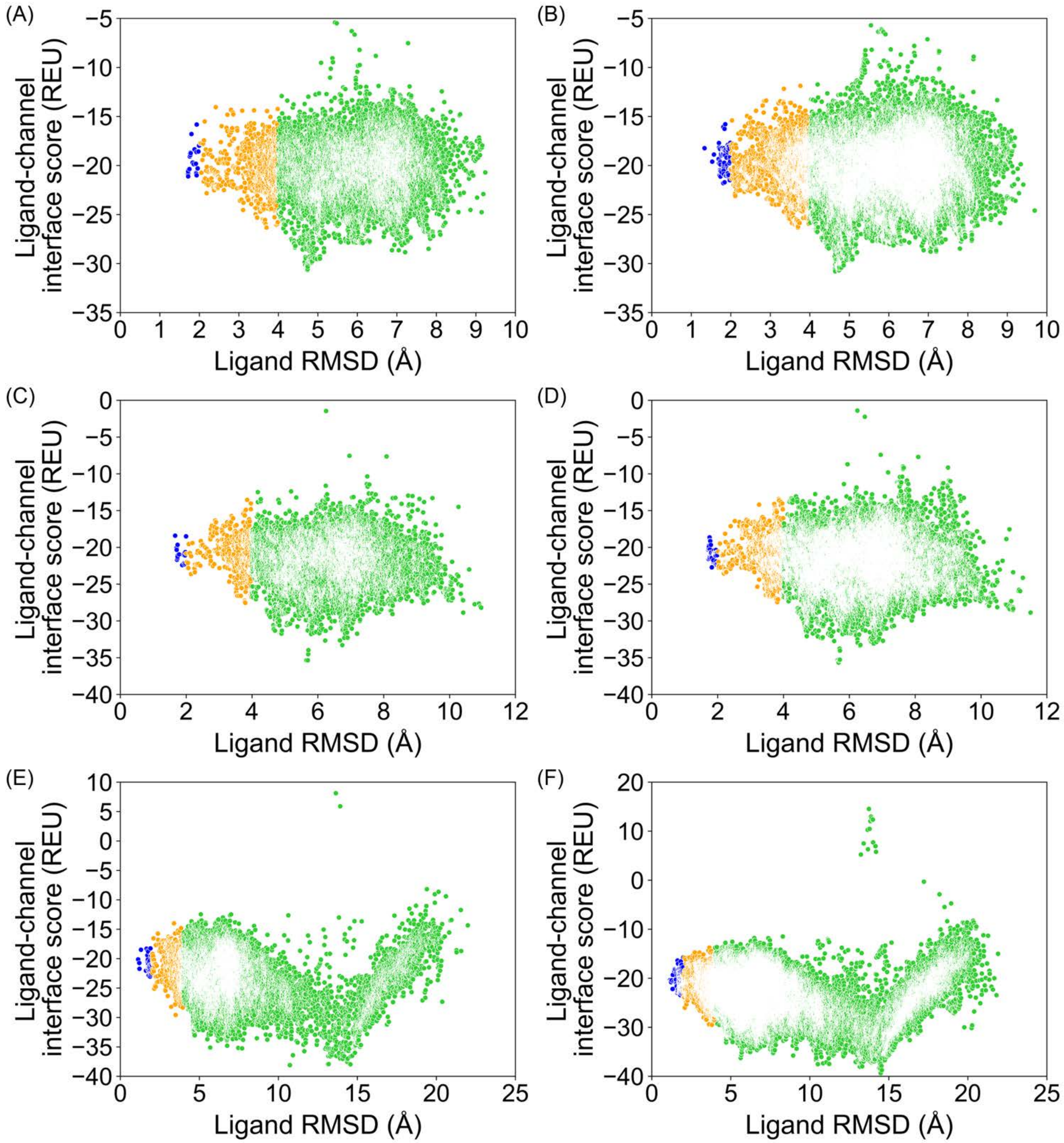

Figure S9.3 PDB 6JPB, GALigandDock, all poses from docking. **(A)** 20,000 total poses, padding 2 Å, **(B)** 100,000 total poses, padding 2 Å, **(C)** 20,000 total poses, padding 4 Å, **(D)** 100,000 total poses, padding 4 Å, **(E)** 20,000 total poses, padding 7 Å, **(F)** 100,000 total poses, padding 7 Å.

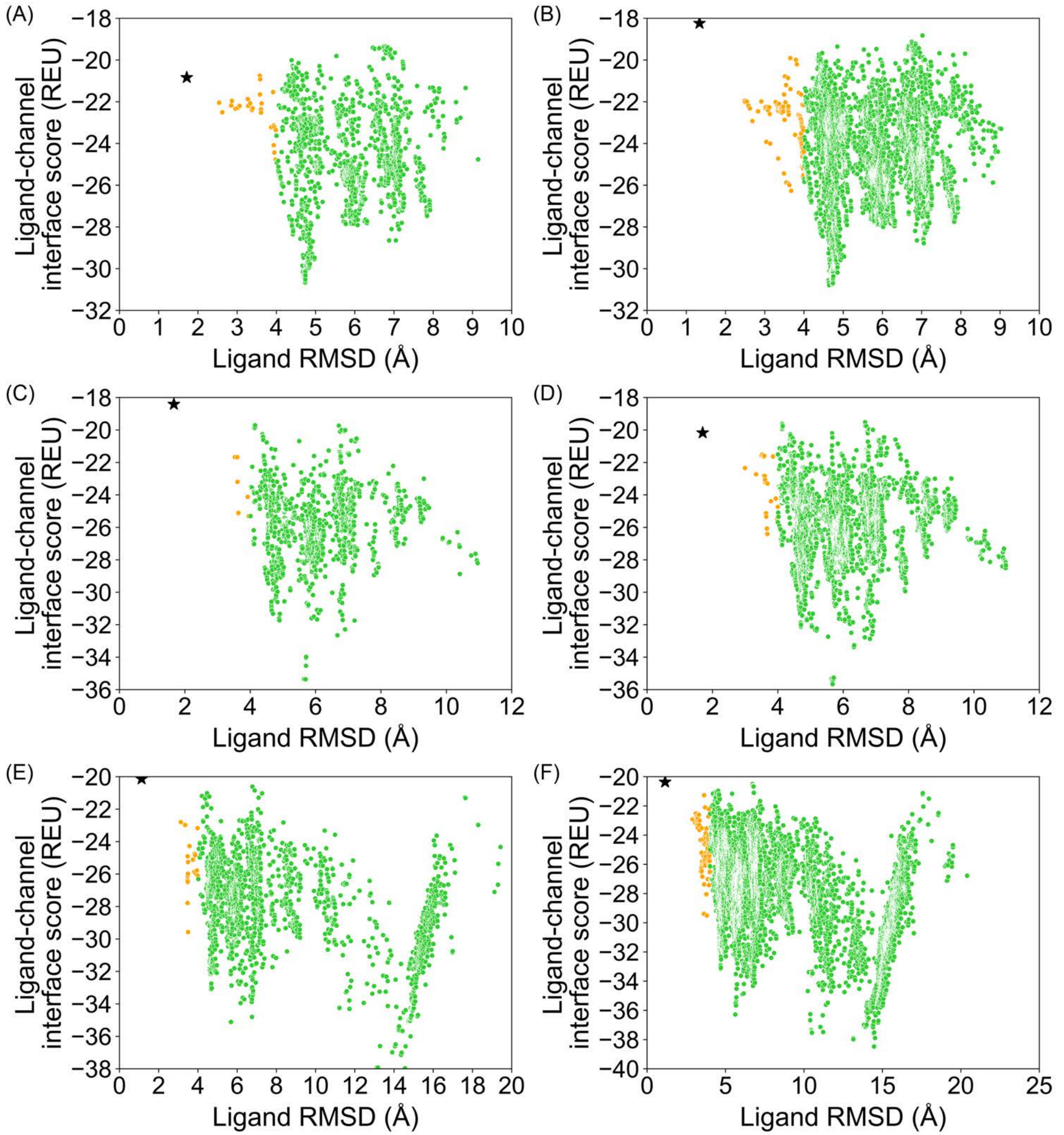

Figure S9.4 PDB 6JPB, GALigandDock, top 10 % of total poses by total energy (`total_score`). **(A)** 20,000 total poses, padding 2 Å, **(B)** 100,000 total poses, padding 2 Å, **(C)** 20,000 total poses, padding 4 Å, **(D)** 100,000 total poses, padding 4 Å, **(E)** 20,000 total poses, padding 7 Å, **(F)** 100,000 total poses, padding 7 Å.

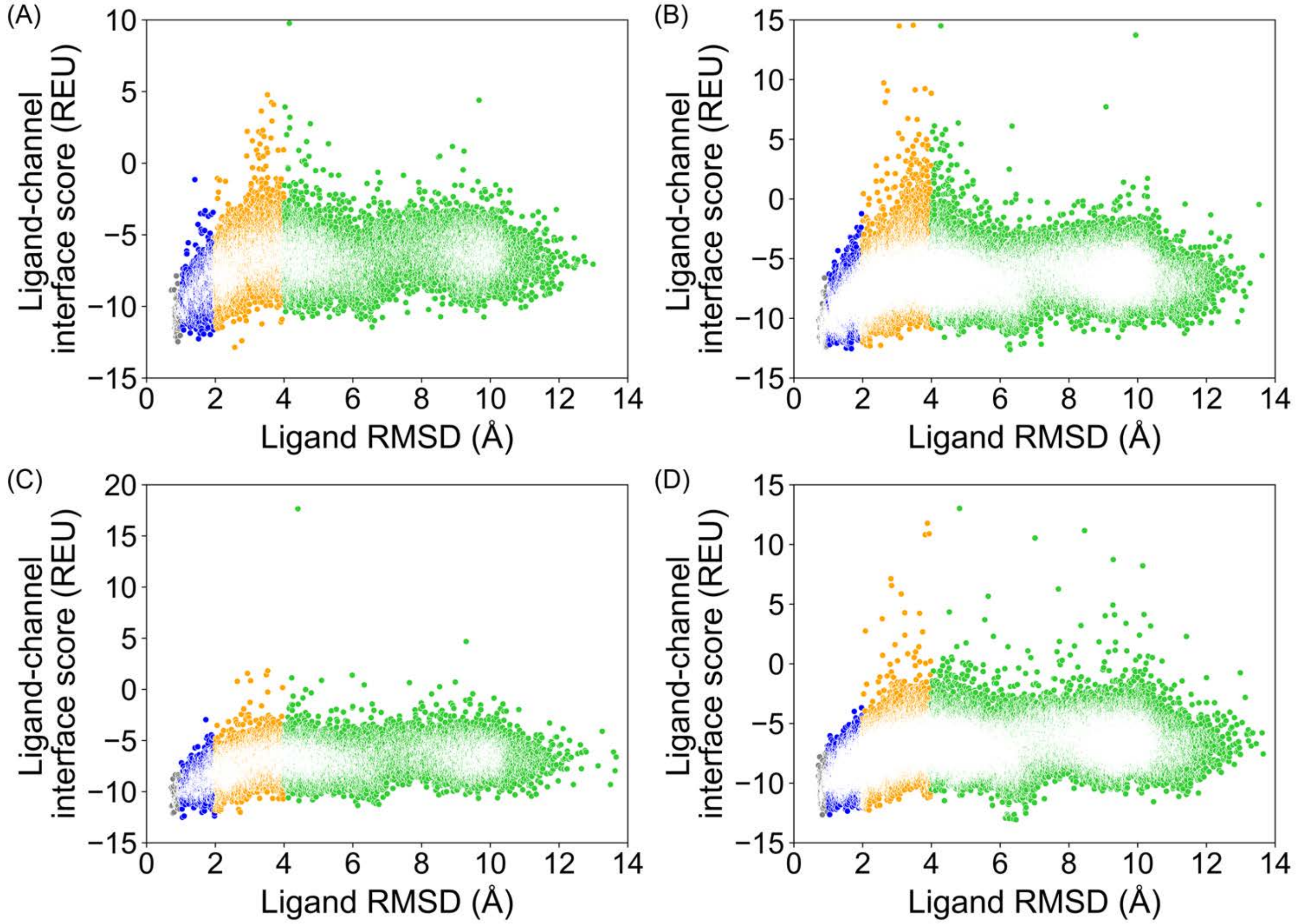

Figure S10.1 PDB 6KZP, RosettaLigand, all poses from docking. **(A)** 20,000 total poses, all ligand atom interface, **(B)** 100,000 total poses, all ligand atom interface, **(C)** 20,000 total poses, ligand neighbor atom interface, **(D)** 100,000 total poses, ligand neighbor atom interface.

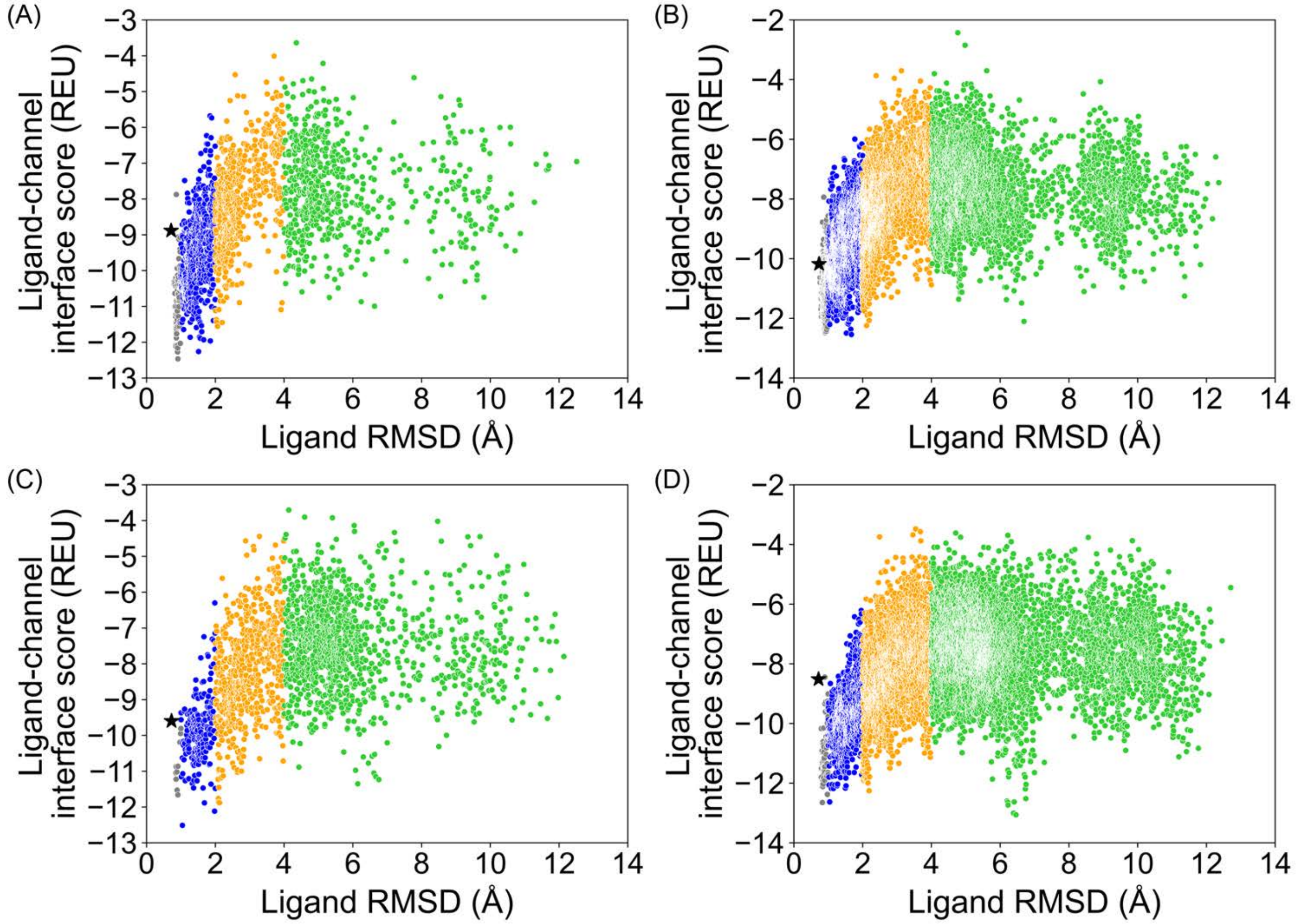

Figure S10.2 PDB 6KZP, RosettaLigand, top 10 % of total poses by total energy (`total_score`). **(A)** 20,000 total poses, all ligand atom interface, **(B)** 100,000 total poses, all ligand atom interface, **(C)** 20,000 total poses, ligand neighbor atom interface, **(D)** 100,000 total poses, ligand neighbor atom interface.

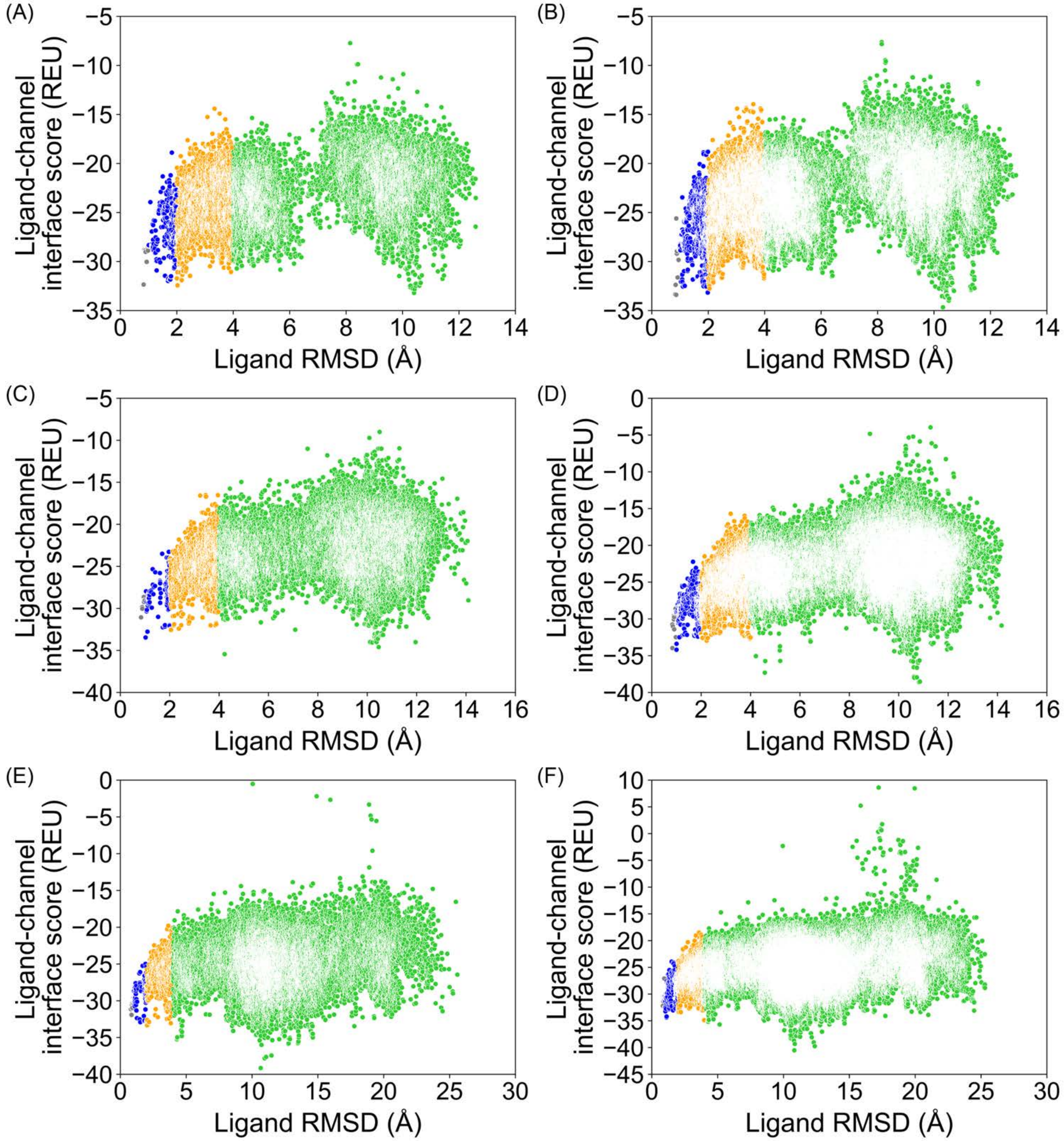

Figure S10.3 PDB 6KZP, GALigandDock, all poses from docking. **(A)** 20,000 total poses, padding 2 Å, **(B)** 100,000 total poses, padding 2 Å, **(C)** 20,000 total poses, padding 4 Å, **(D)** 100,000 total poses, padding 4 Å, **(E)** 20,000 total poses, padding 7 Å, **(F)** 100,000 total poses, padding 7 Å.

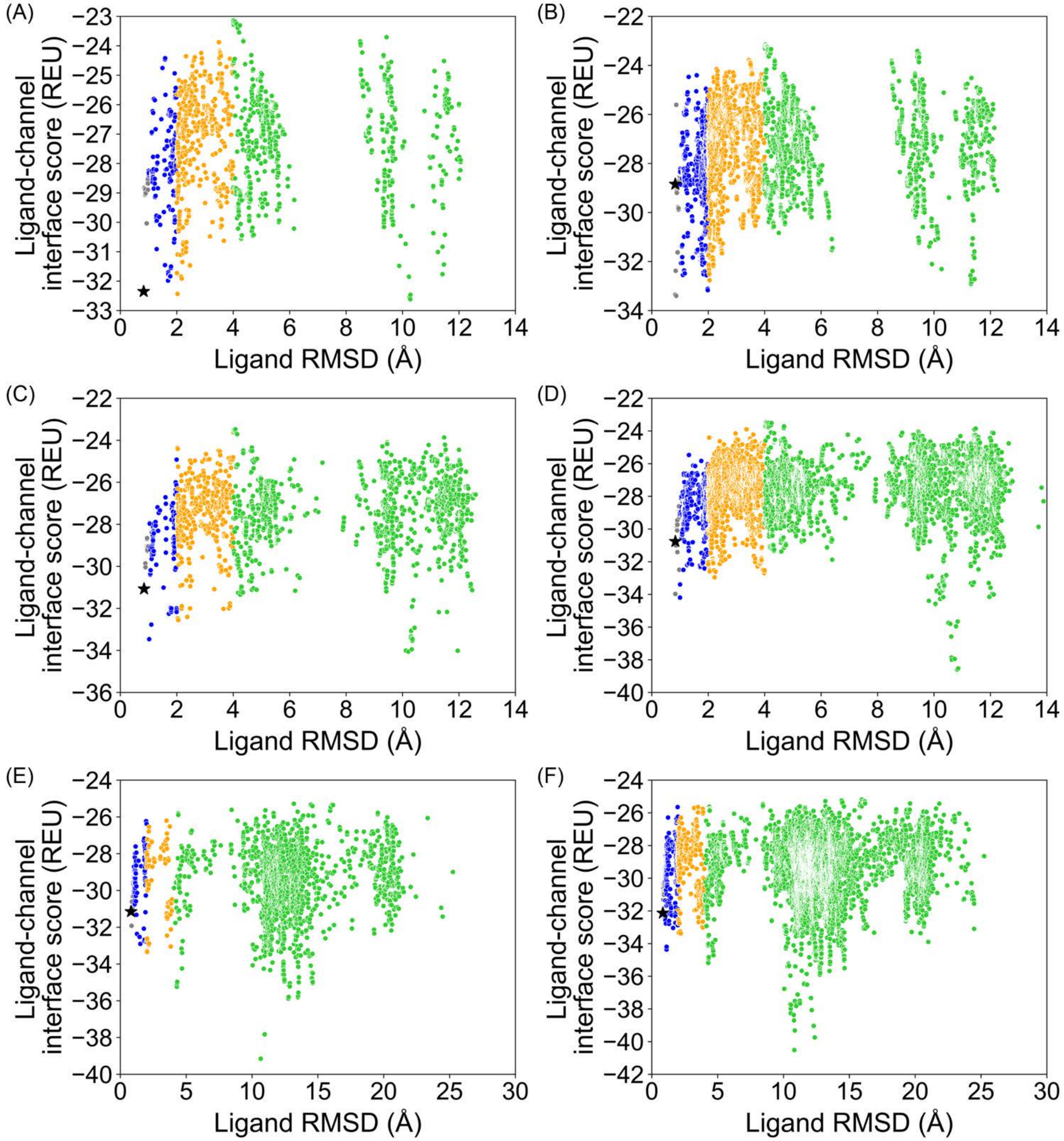

Figure S10.4 PDB 6KZP, GALigandDock, top 10 % of total poses by total energy (`total_score`). **(A)** 20,000 total poses, padding 2 Å, **(B)** 100,000 total poses, padding 2 Å, **(C)** 20,000 total poses, padding 4 Å, **(D)** 100,000 total poses, padding 4 Å, **(E)** 20,000 total poses, padding 7 Å, **(F)** 100,000 total poses, padding 7 Å.

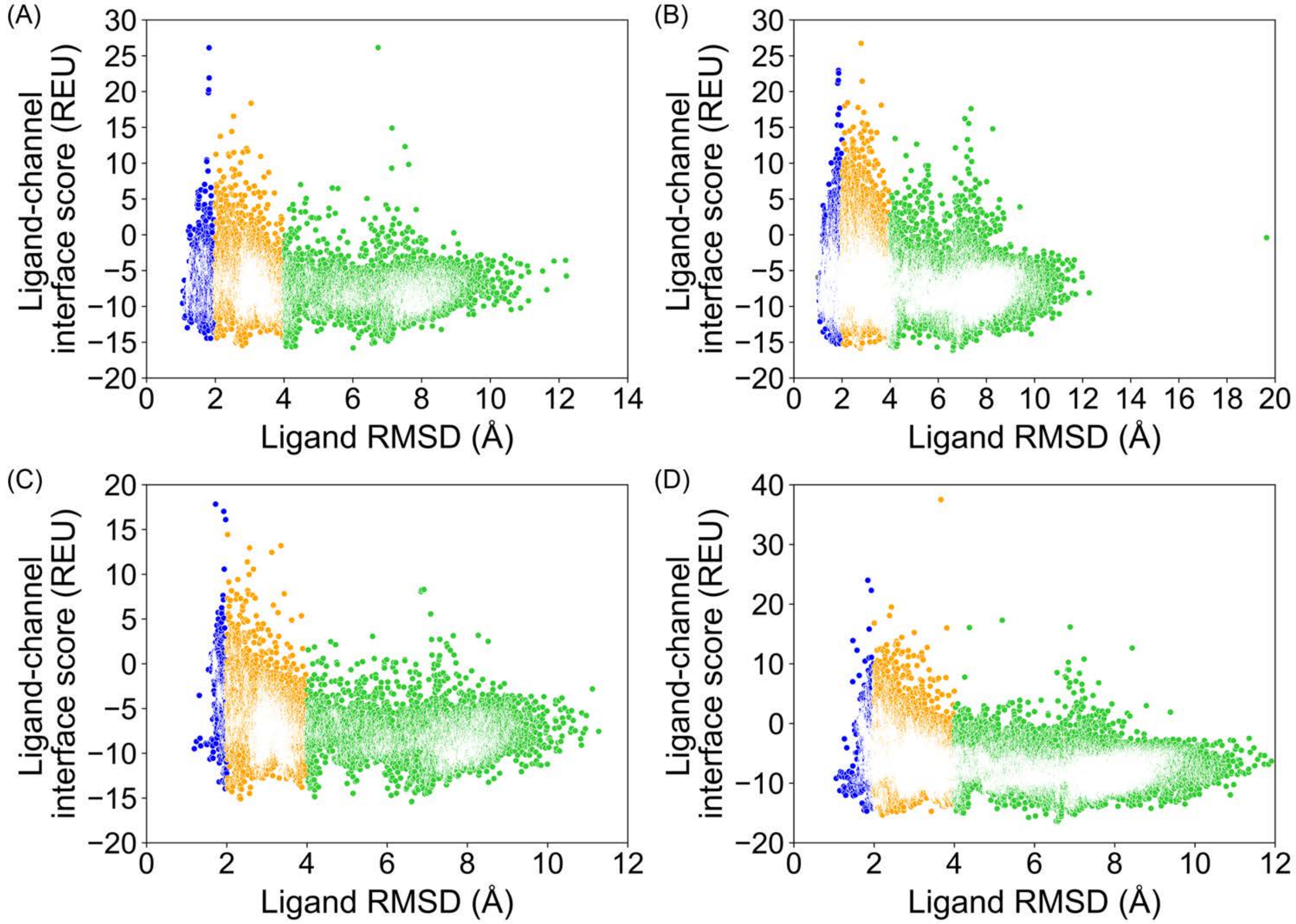

Figure S11.1 PDB 6U88, RosettaLigand, all poses from docking. **(A)** 20,000 total poses, all ligand atom interface, **(B)** 100,000 total poses, all ligand atom interface, **(C)** 20,000 total poses, ligand neighbor atom interface, **(D)** 100,000 total poses, ligand neighbor atom interface.

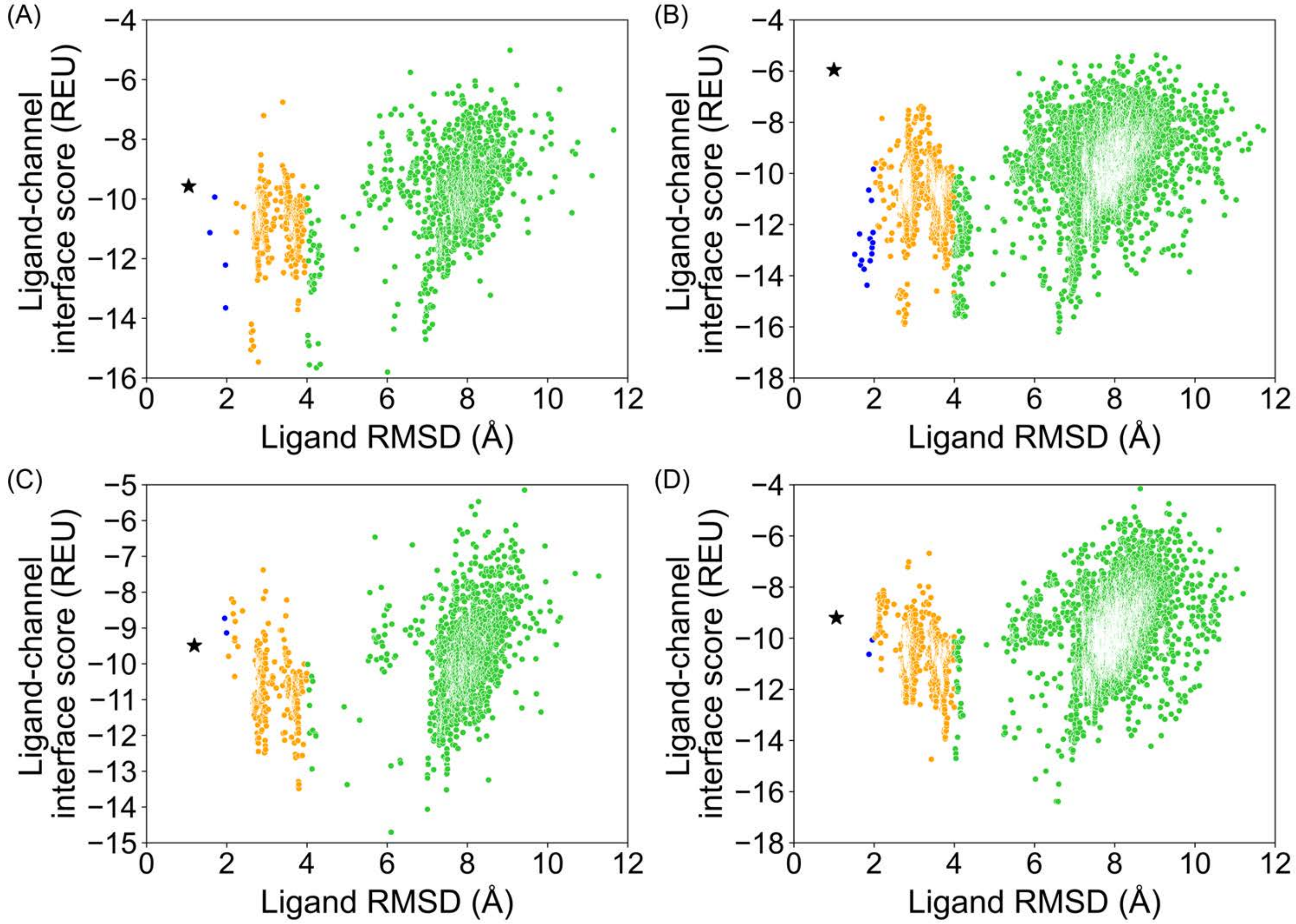

Figure S11.2 PDB 6U88, RosettaLigand, top 10 % of total poses by total energy (`total_score`). **(A)** 20,000 total poses, all ligand atom interface, **(B)** 100,000 total poses, all ligand atom interface, **(C)** 20,000 total poses, ligand neighbor atom interface, **(D)** 100,000 total poses, ligand neighbor atom interface.

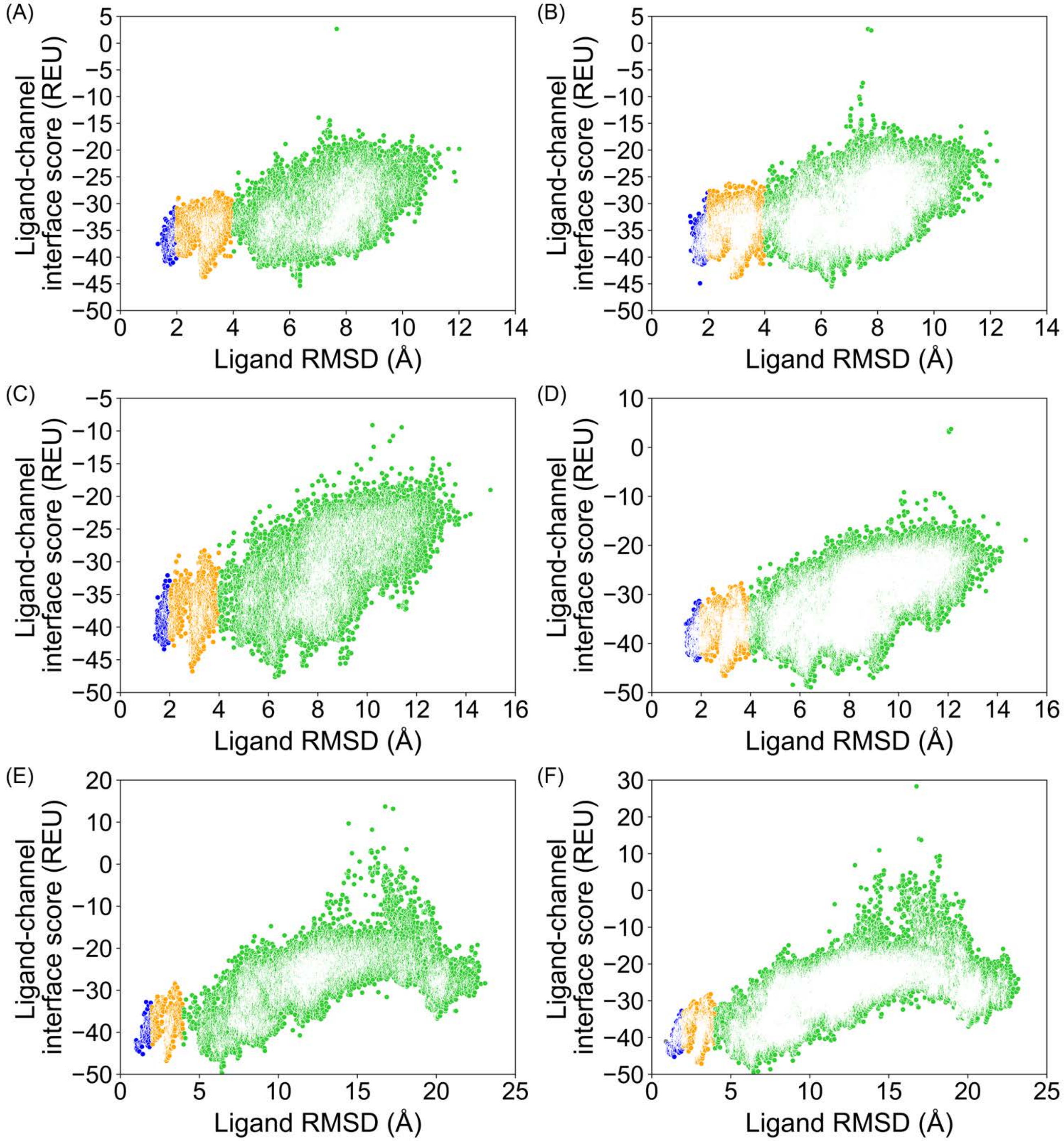

Figure S11.3 PDB 6U88, GALigandDock, all poses from docking. **(A)** 20,000 total poses, padding 2 Å, **(B)** 100,000 total poses, padding 2 Å, **(C)** 20,000 total poses, padding 4 Å, **(D)** 100,000 total poses, padding 4 Å, **(E)** 20,000 total poses, padding 7 Å, **(F)** 100,000 total poses, padding 7 Å.

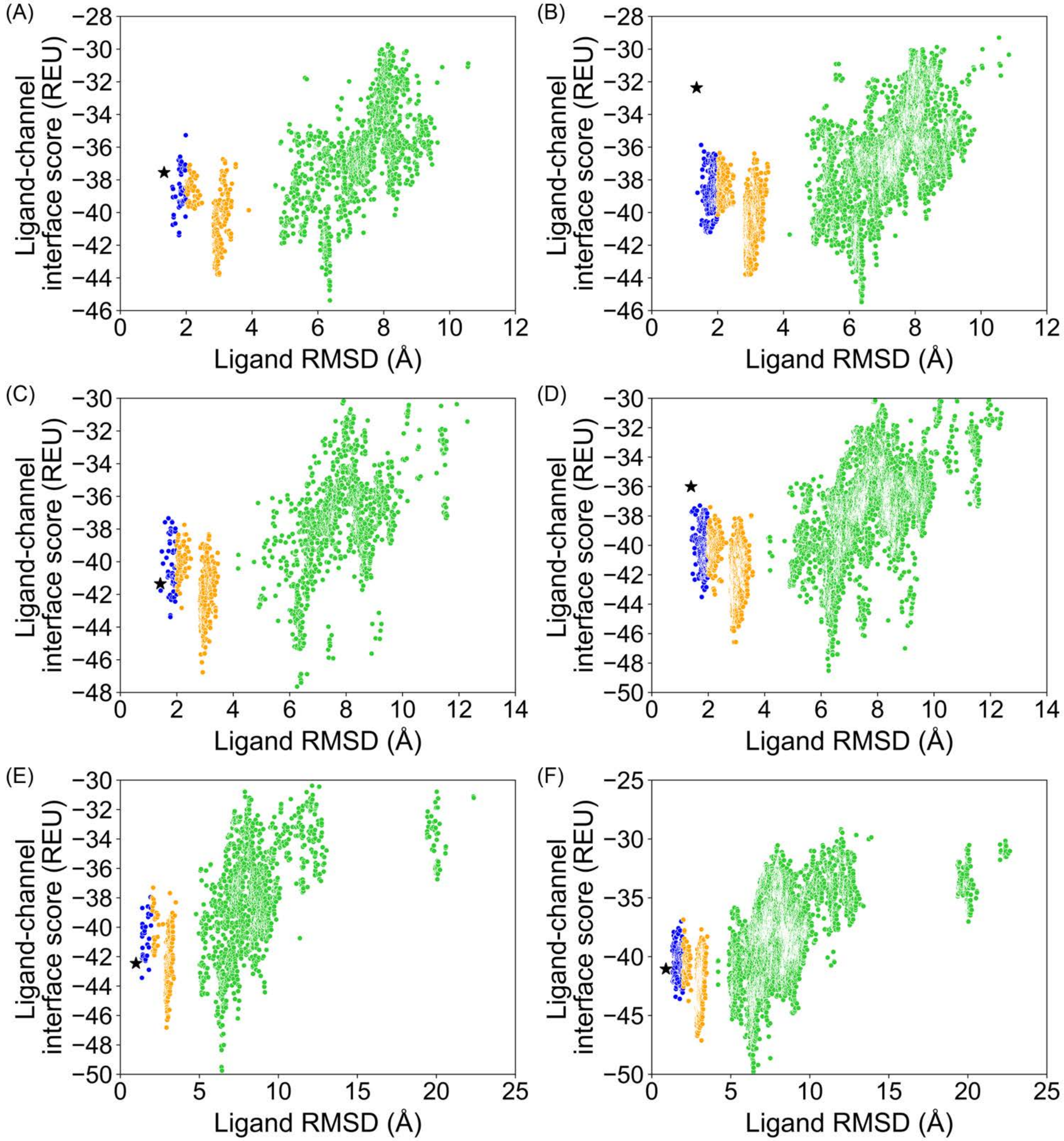

Figure S11.4 PDB 6U88, GALigandDock, top 10 % of total poses by total energy (`total_score`). **(A)** 20,000 total poses, padding 2 Å, **(B)** 100,000 total poses, padding 2 Å, **(C)** 20,000 total poses, padding 4 Å, **(D)** 100,000 total poses, padding 4 Å, **(E)** 20,000 total poses, padding 7 Å, **(F)** 100,000 total poses, padding 7 Å.

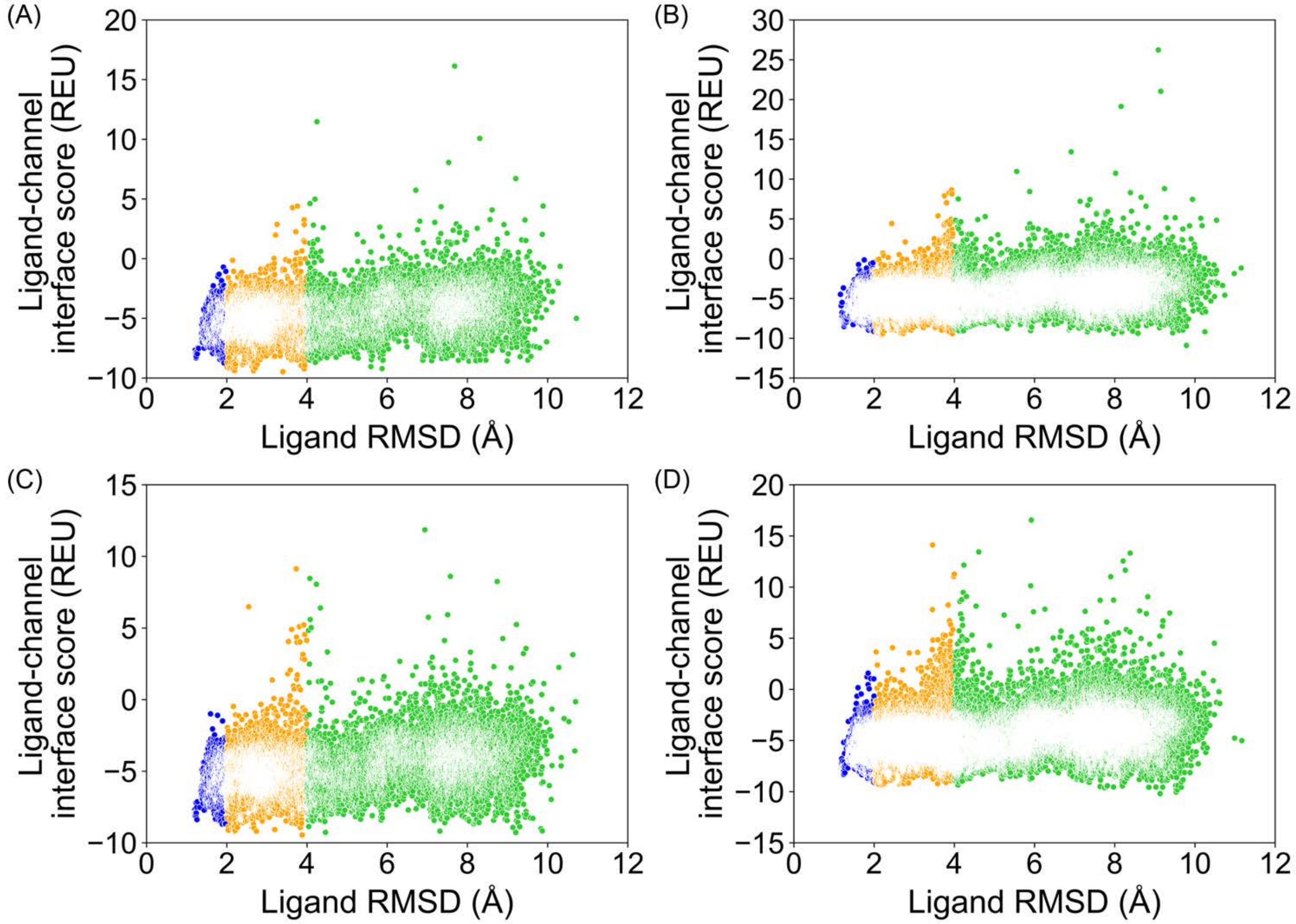

Figure S12.1 PDB 6UZ0, RosettaLigand, all poses from docking. **(A)** 20,000 total poses, all ligand atom interface, **(B)** 100,000 total poses, all ligand atom interface, **(C)** 20,000 total poses, ligand neighbor atom interface, **(D)** 100,000 total poses, ligand neighbor atom interface.

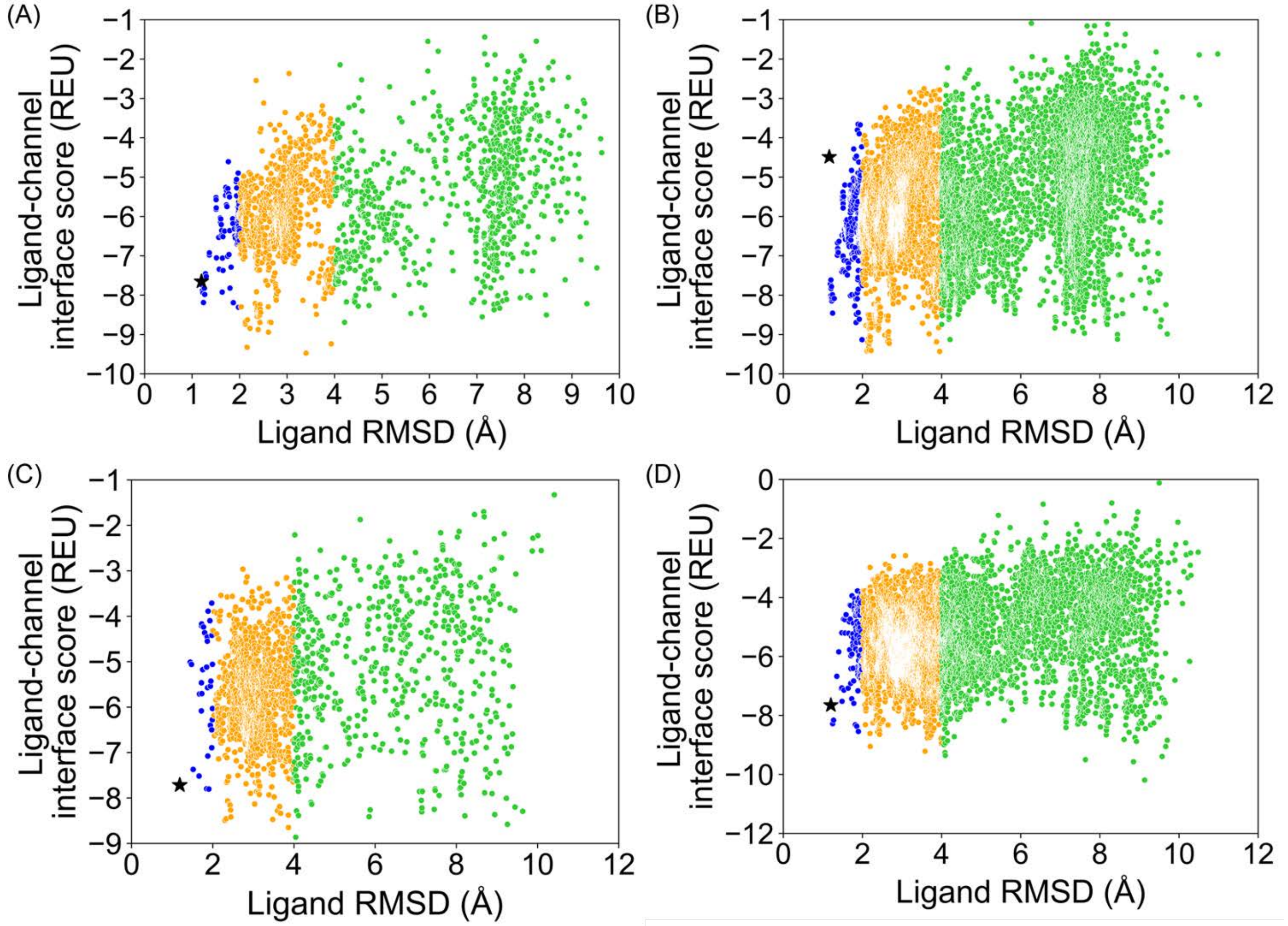

Figure S12.2 PDB 6UZ0, RosettaLigand, top 10 % of total poses by total energy (`total_score`). **(A)** 20,000 total poses, all ligand atom interface, **(B)** 100,000 total poses, all ligand atom interface, **(C)** 20,000 total poses, ligand neighbor atom interface, **(D)** 100,000 total poses, ligand neighbor atom interface.

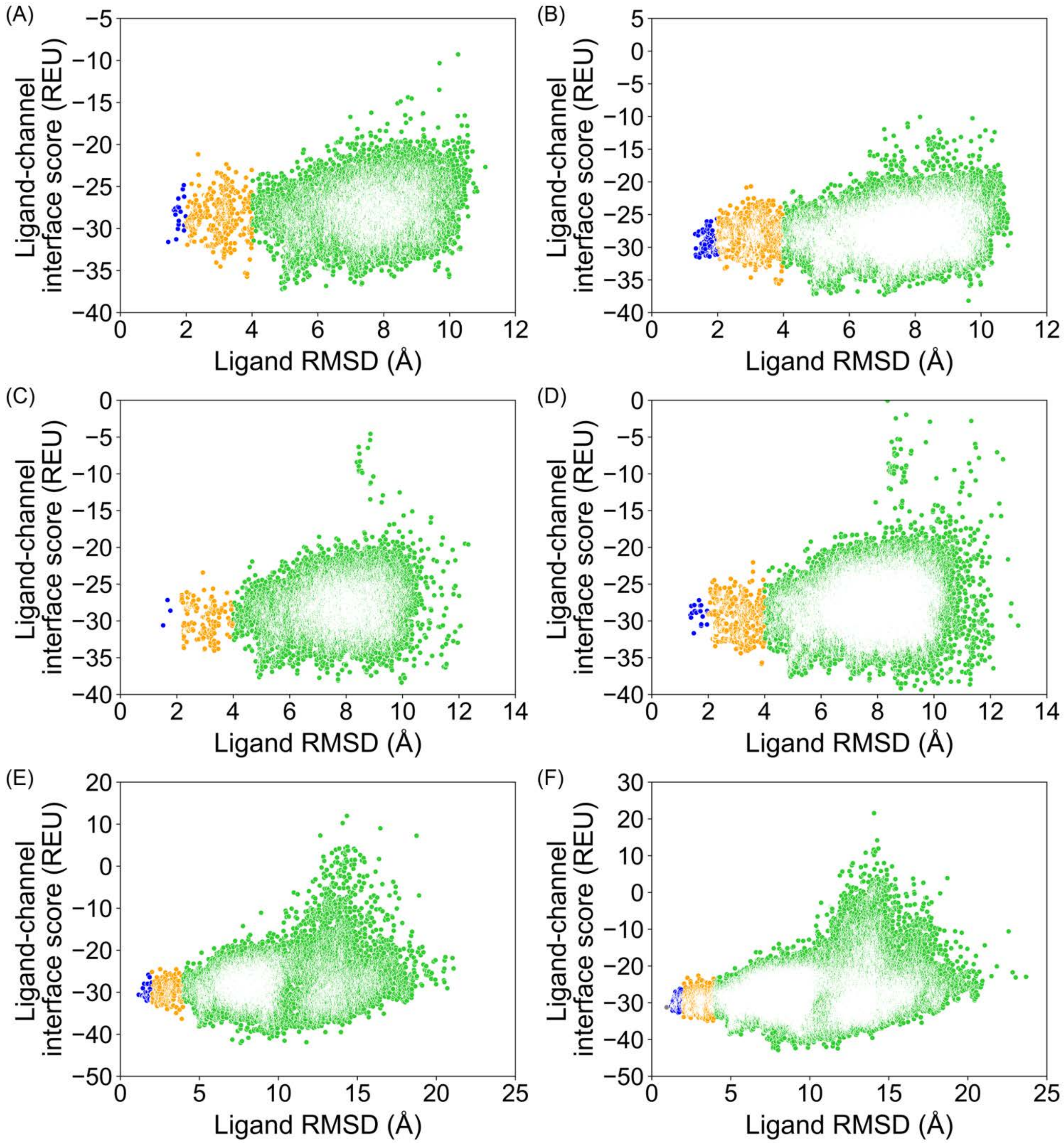

Figure S12.3 PDB 6UZ0, GALigandDock, all poses from docking. **(A)** 20,000 total poses, padding 2 Å, **(B)** 100,000 total poses, padding 2 Å, **(C)** 20,000 total poses, padding 4 Å, **(D)** 100,000 total poses, padding 4 Å, **(E)** 20,000 total poses, padding 7 Å, **(F)** 100,000 total poses, padding 7 Å.

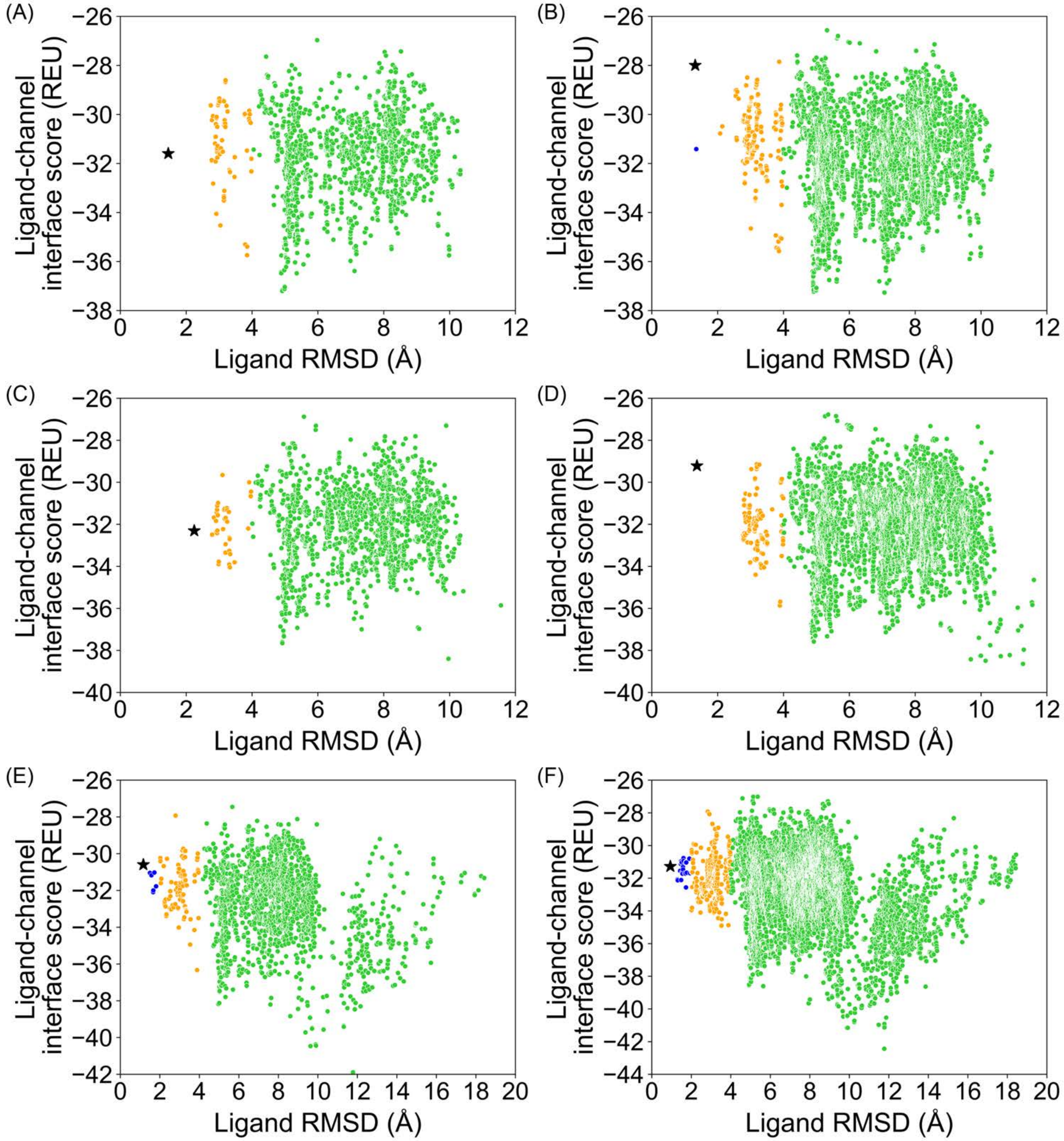

Figure S12.4 PDB 6UZ0, GALigandDock, top 10 % of total poses by total energy (`total_score`). **(A)** 20,000 total poses, padding 2 Å, **(B)** 100,000 total poses, padding 2 Å, **(C)** 20,000 total poses, padding 4 Å, **(D)** 100,000 total poses, padding 4 Å, **(E)** 20,000 total poses, padding 7 Å, **(F)** 100,000 total poses, padding 7 Å.

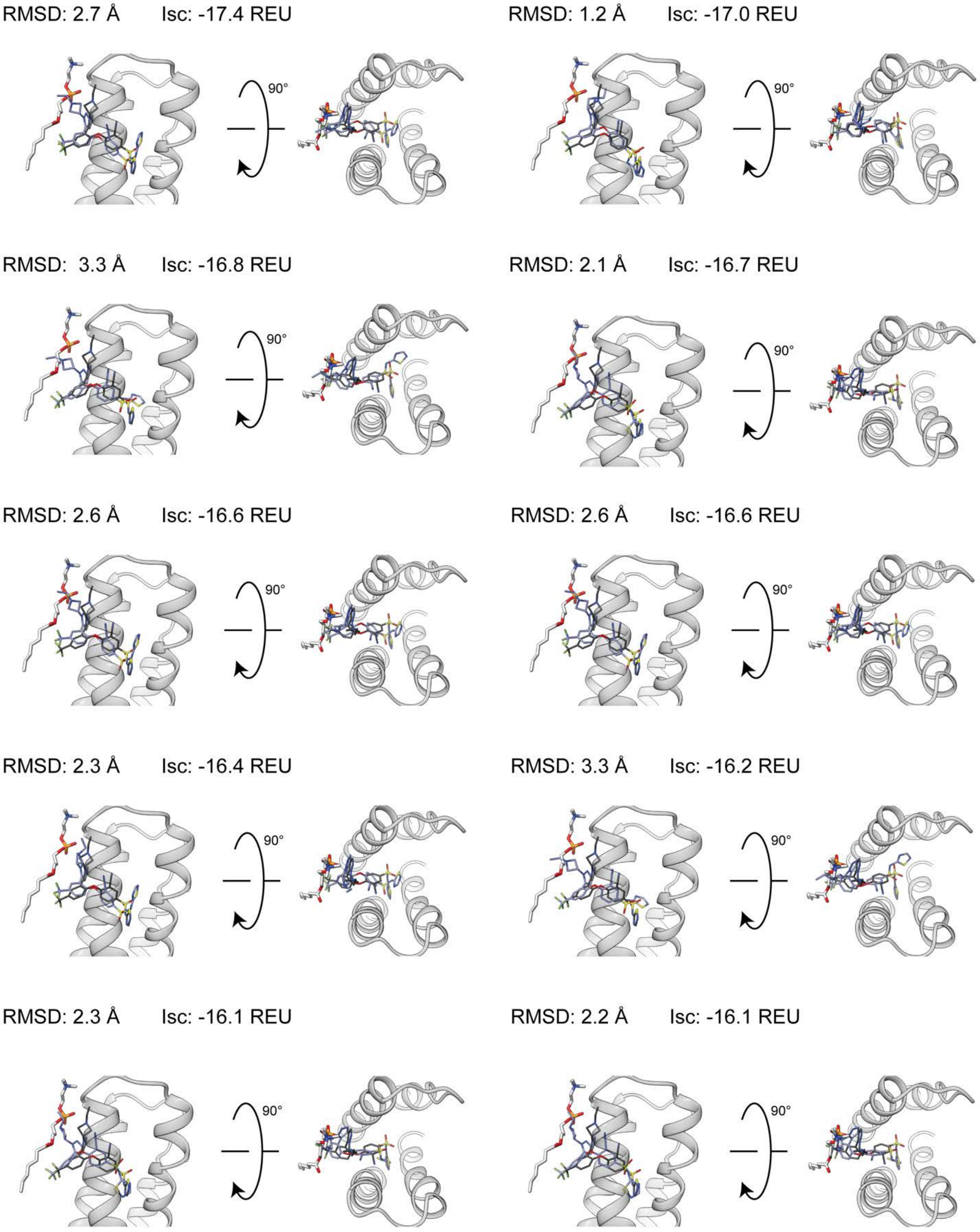

Figure S13.1. PDB 5EK0. Top 10 interface-scoring (`interface_delta_X`) poses from RosettaLigand, 100,000 total poses, all atom ligand interface.

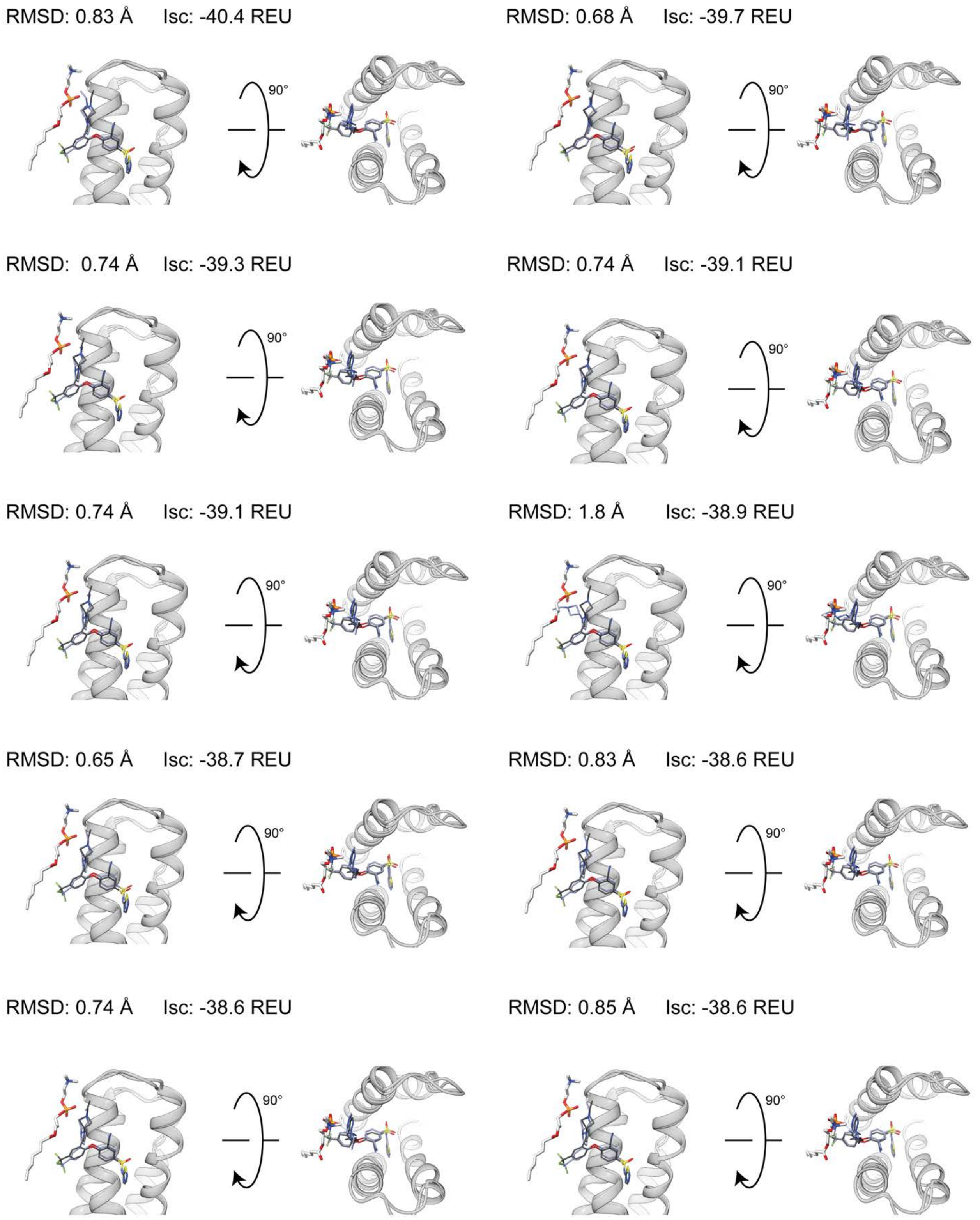

Figure S13.2. PDB 5EK0. Top 10 interface-scoring (`LigInterface`) poses from GALigandDock, 100,000 total poses, padding 7 Å.

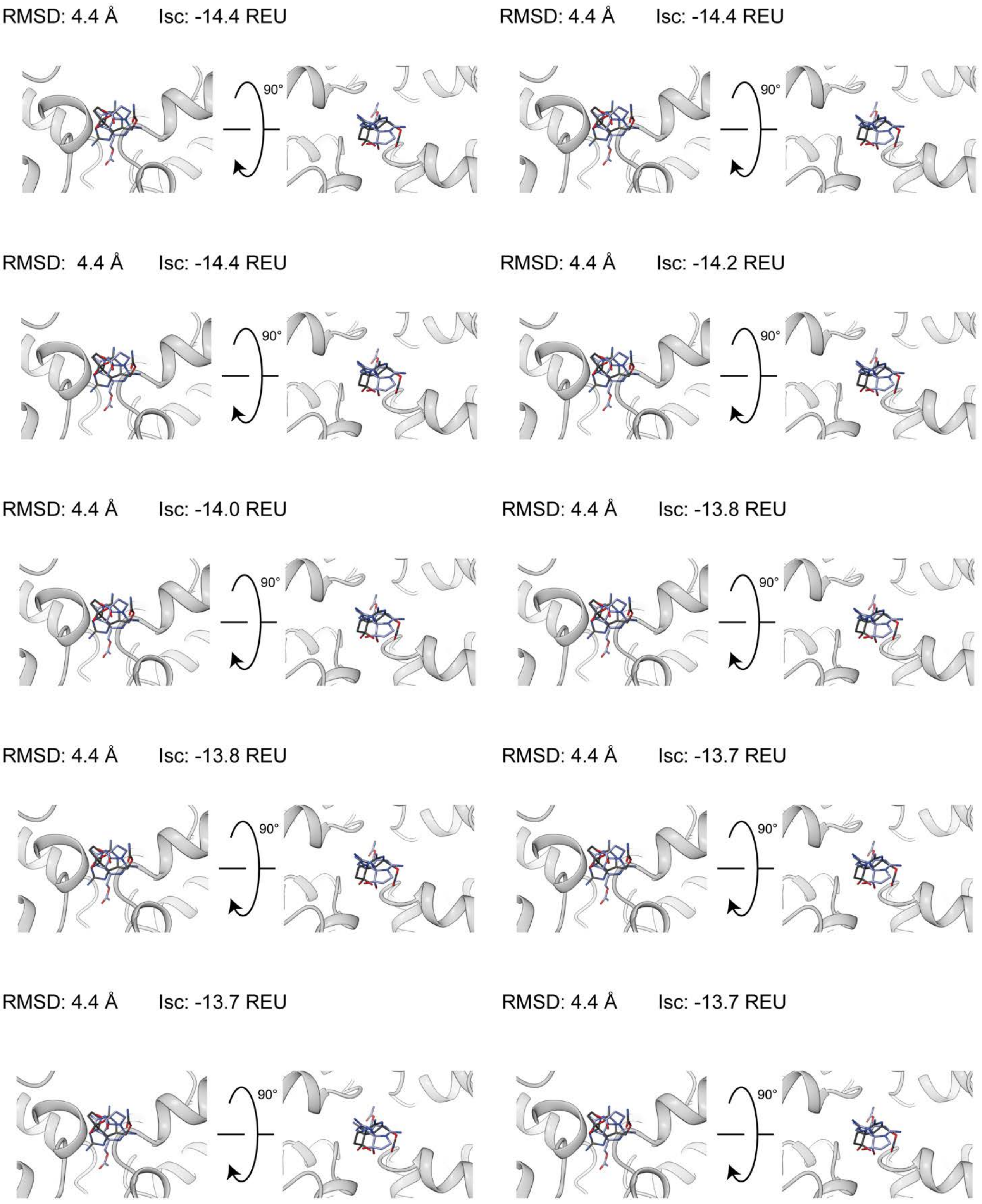

Figure S14.1. PDB 6J8G. Top 10 interface-scoring (`interface_delta_X`) poses from RosettaLigand, 100,000 total poses, all atom ligand interface.

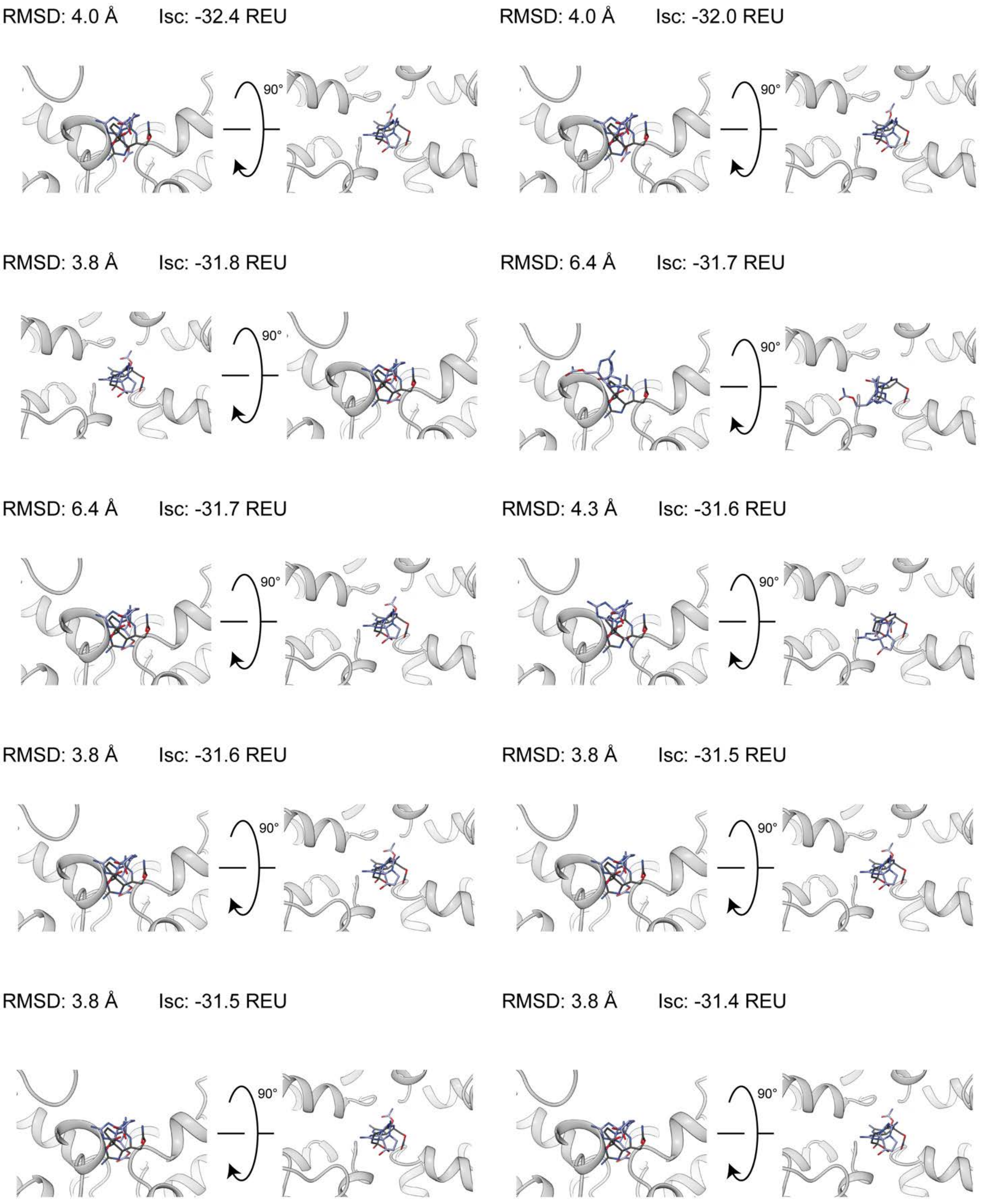

Figure S14.2. PDB 6J8G. Top 10 interface-scoring (`LigInterface`) poses from GALigandDock, 100,000 total poses, padding 7 Å.

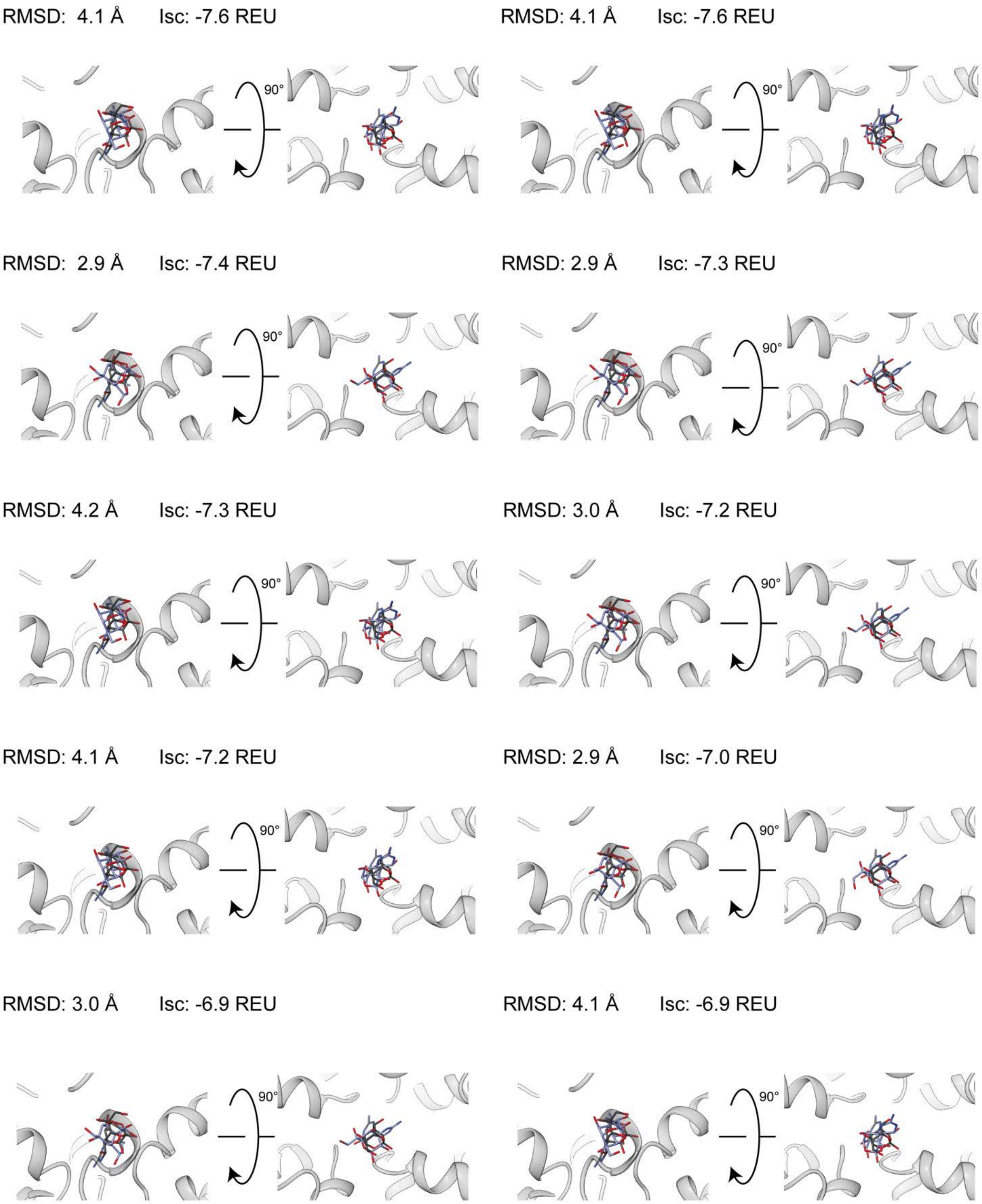

Figure S15.1. PDB 6J8I. Top 10 interface-scoring (`interface_delta_X`) poses from RosettaLigand, 100,000 total poses, all atom ligand interface.

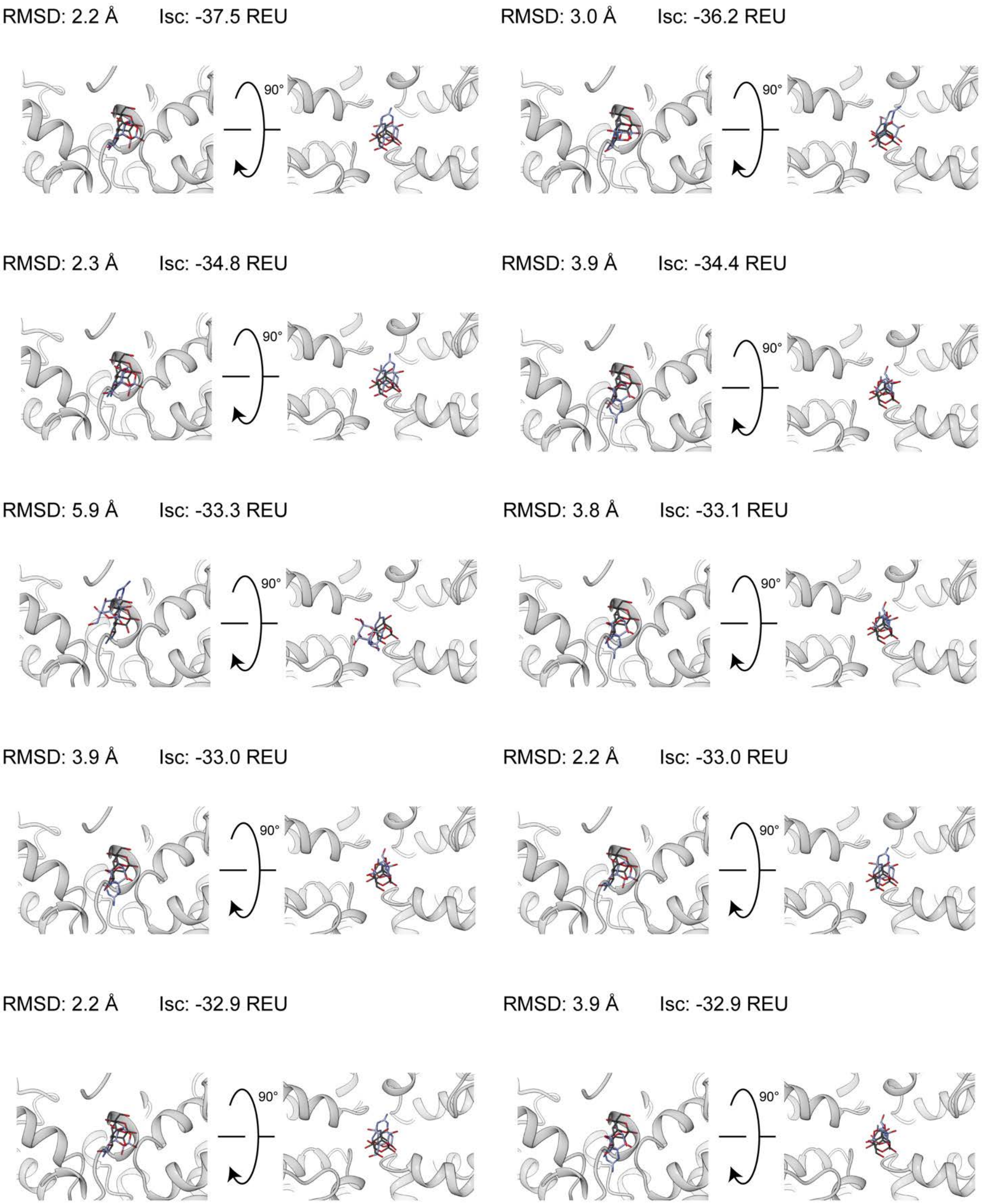

Figure S15.2. PDB 6J8I. Top 10 interface-scoring (`LigInterface`) poses from GALigandDock, 100,000 total poses, padding 7 Å.

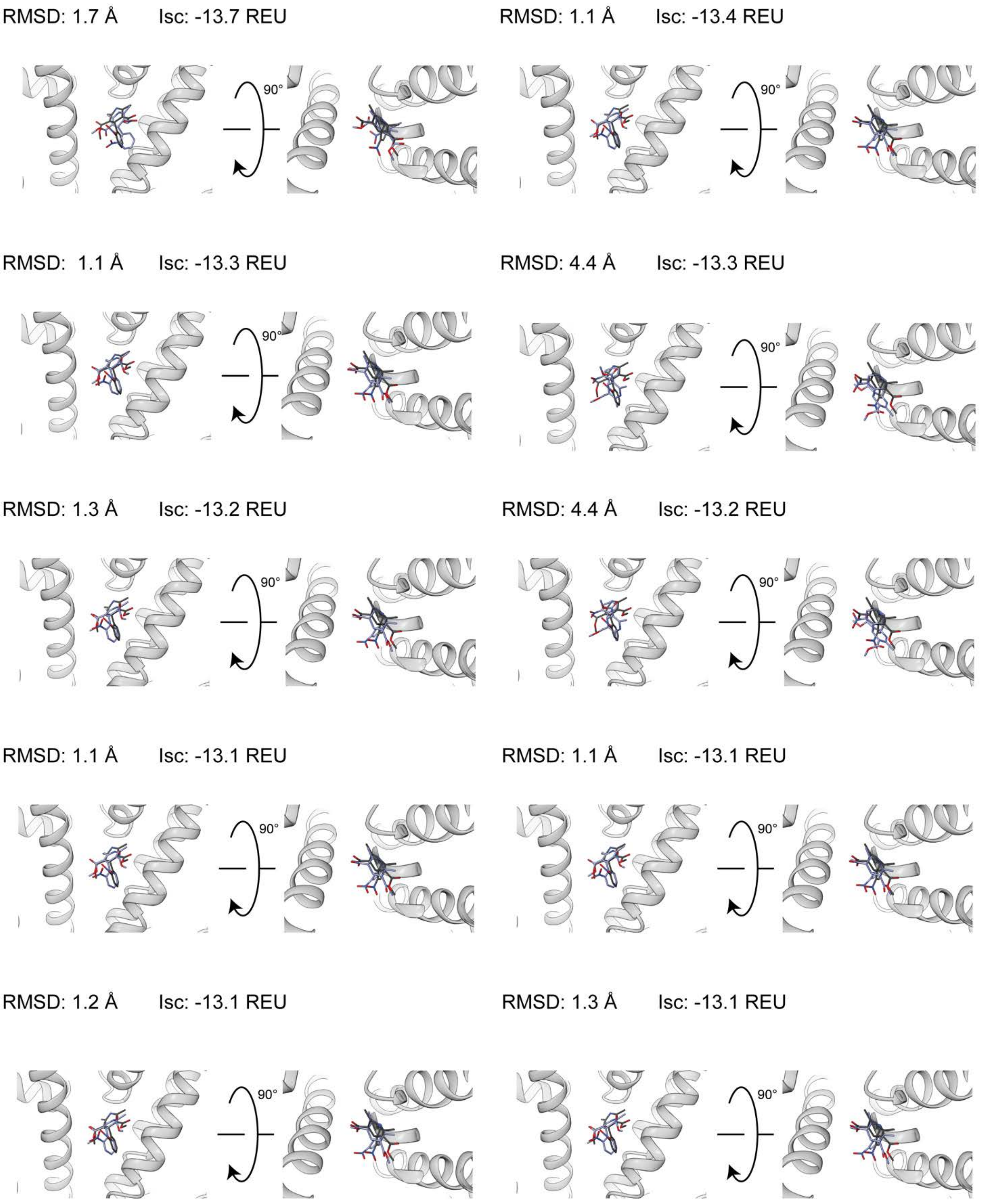

Figure S16.1. PDB 6JP5. Top 10 interface-scoring (`interface_delta_X`) poses from RosettaLigand, 100,000 total poses, all atom ligand interface.

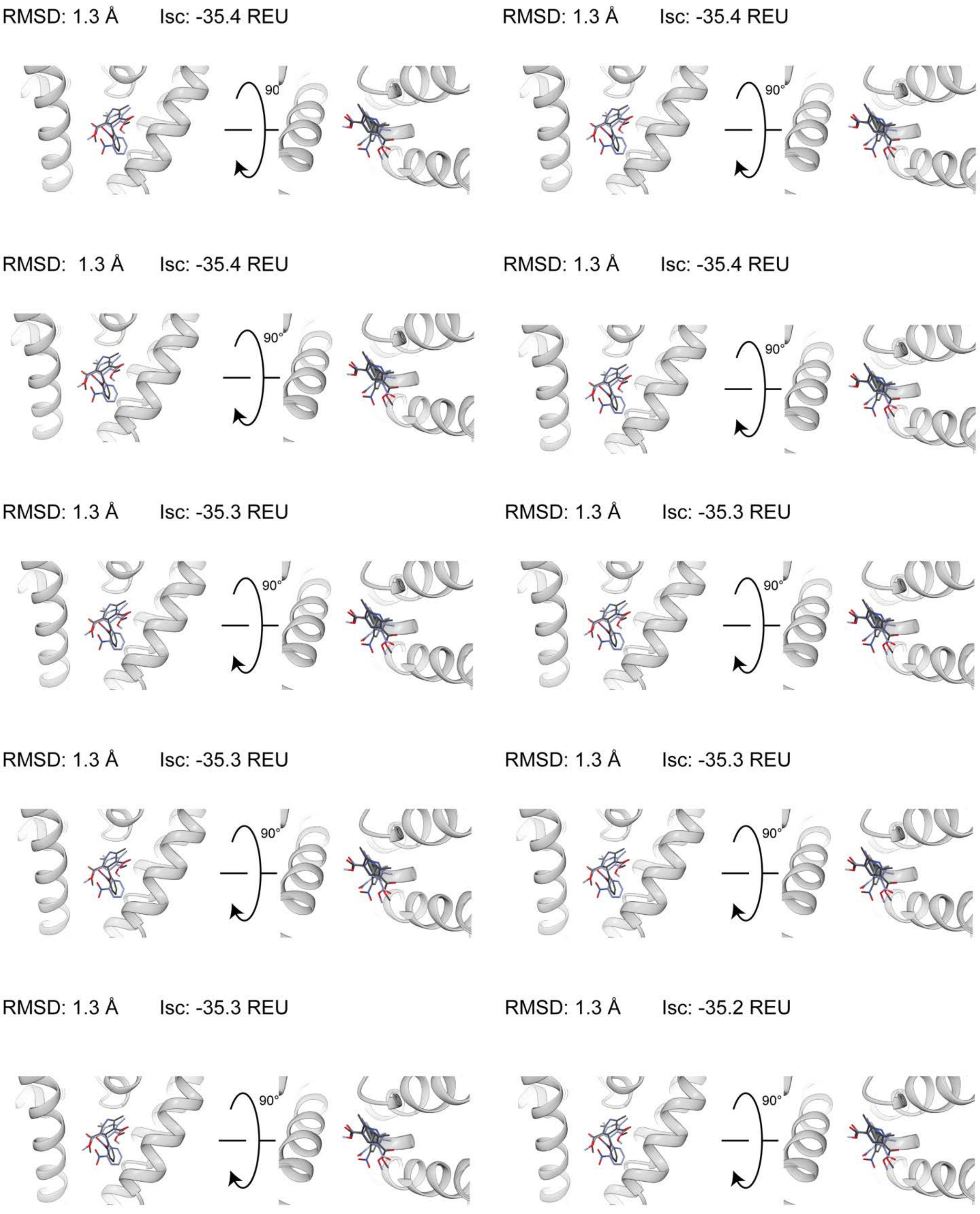

Figure S16.2. PDB 6JP5. Top 10 interface-scoring (`LigInterface`) poses from GALigandDock, 100,000 total poses, padding 7 Å.

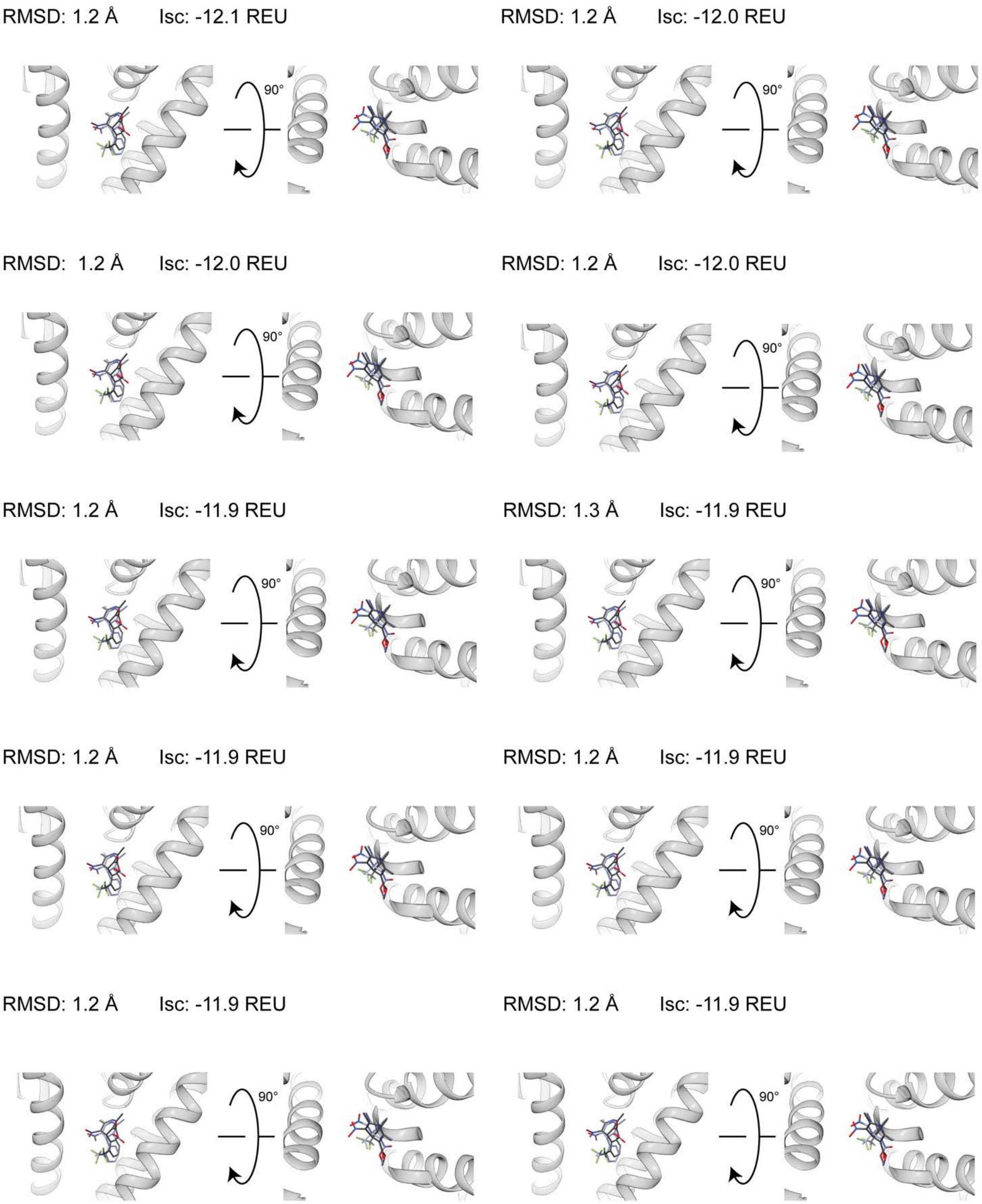

Figure S17.1. PDB 6JP8. Top 10 interface-scoring (`interface_delta_X`) poses from RosettaLigand, 100,000 total poses, all atom ligand interface.

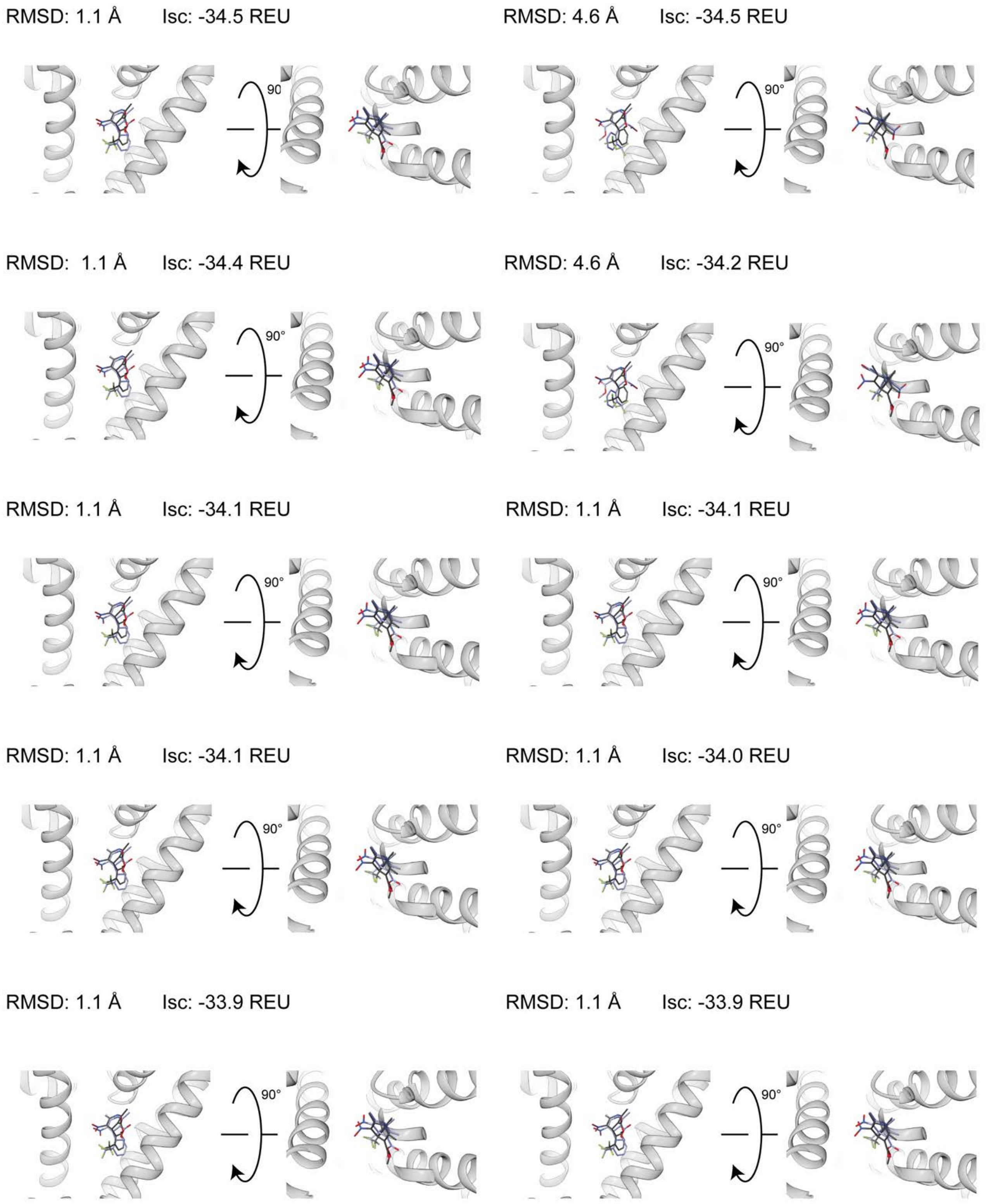

Figure S17.2. PDB 6JP8. Top 10 interface-scoring (`LigInterface`) poses from GALigandDock, 100,000 total poses, padding 7 Å.

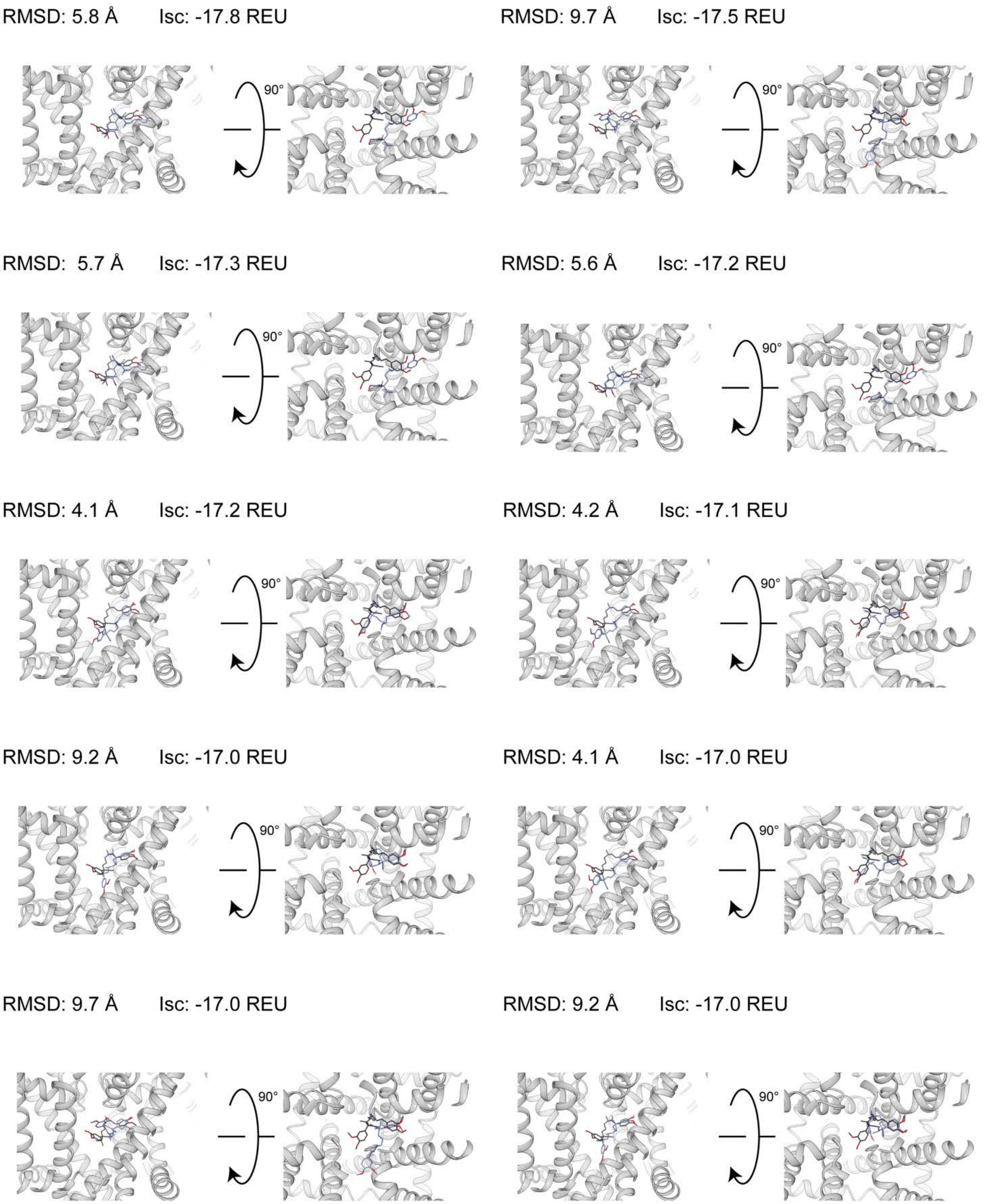

Figure S18.1. PDB 6JPA-1. Top 10 interface-scoring (`interface_delta_X`) poses from RosettaLigand, 100,000 total poses, all atom ligand interface.

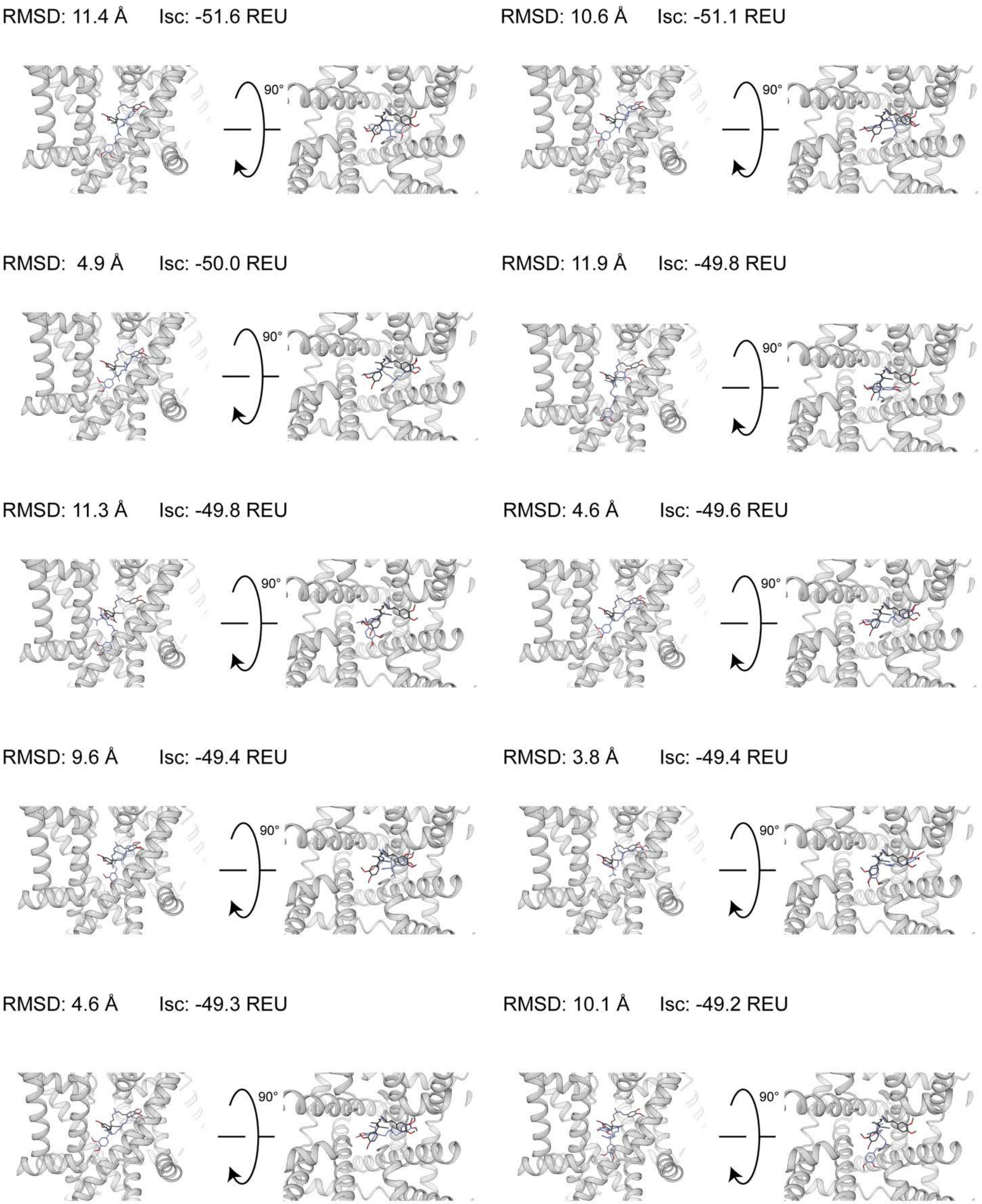

Figure S18.2. PDB 6JPA-1. Top 10 interface-scoring (`LigInterface`) poses from GALigandDock, 100,000 total poses, padding 7 Å.

RMSD: 10.8 Å Isc: -16.8 REU

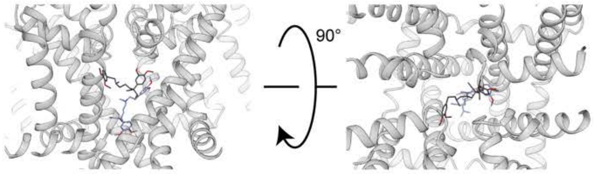

RMSD: 9.3 Å Isc: -16.6 REU

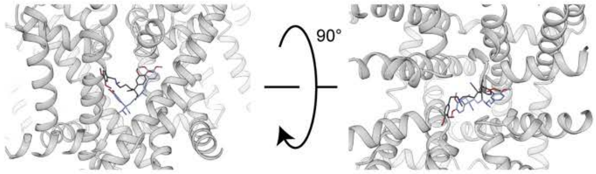

RMSD: 10.0 Å Isc: -16.5 REU

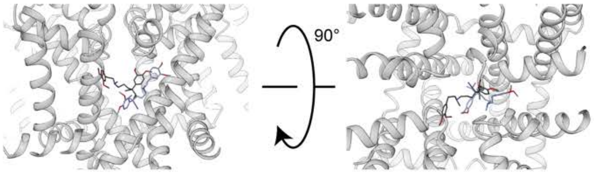

RMSD: 9.2 Å Isc: -16.4 REU

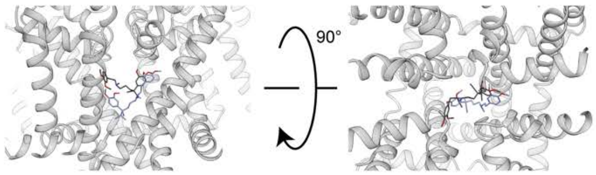

RMSD: 9.8 Å Isc: -16.4 REU

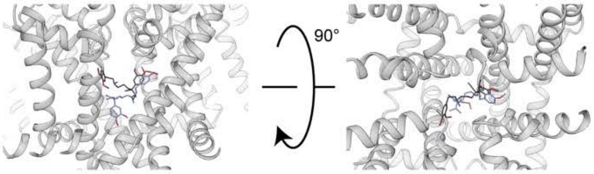

RMSD: 9.4 Å Isc: -16.3 REU

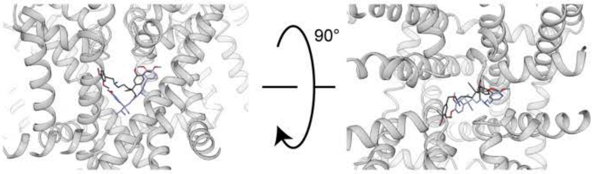

RMSD: 8.5 Å Isc: -16.3 REU

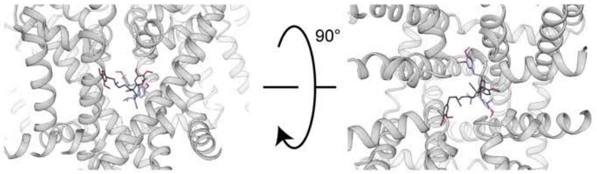

RMSD: 8.7 Å Isc: -16.3 REU

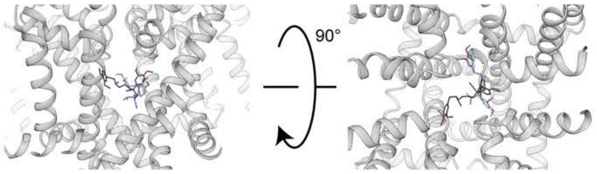

RMSD: 9.7 Å Isc: -16.2 REU

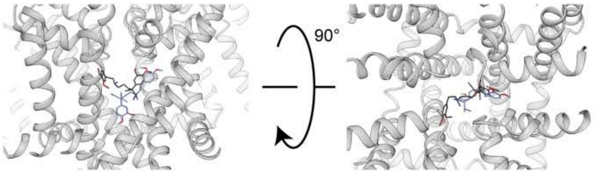

RMSD: 9.2 Å Isc: -16.2 REU

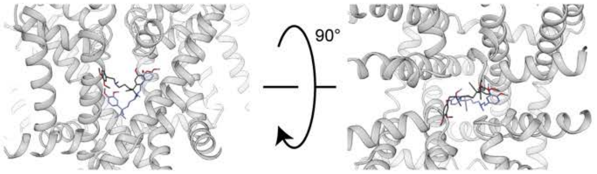

Figure S19.1. PDB 6JPA-2. Top 10 interface-scoring (`interface_delta_X`) poses from RosettaLigand, 100,000 total poses, all atom ligand interface.

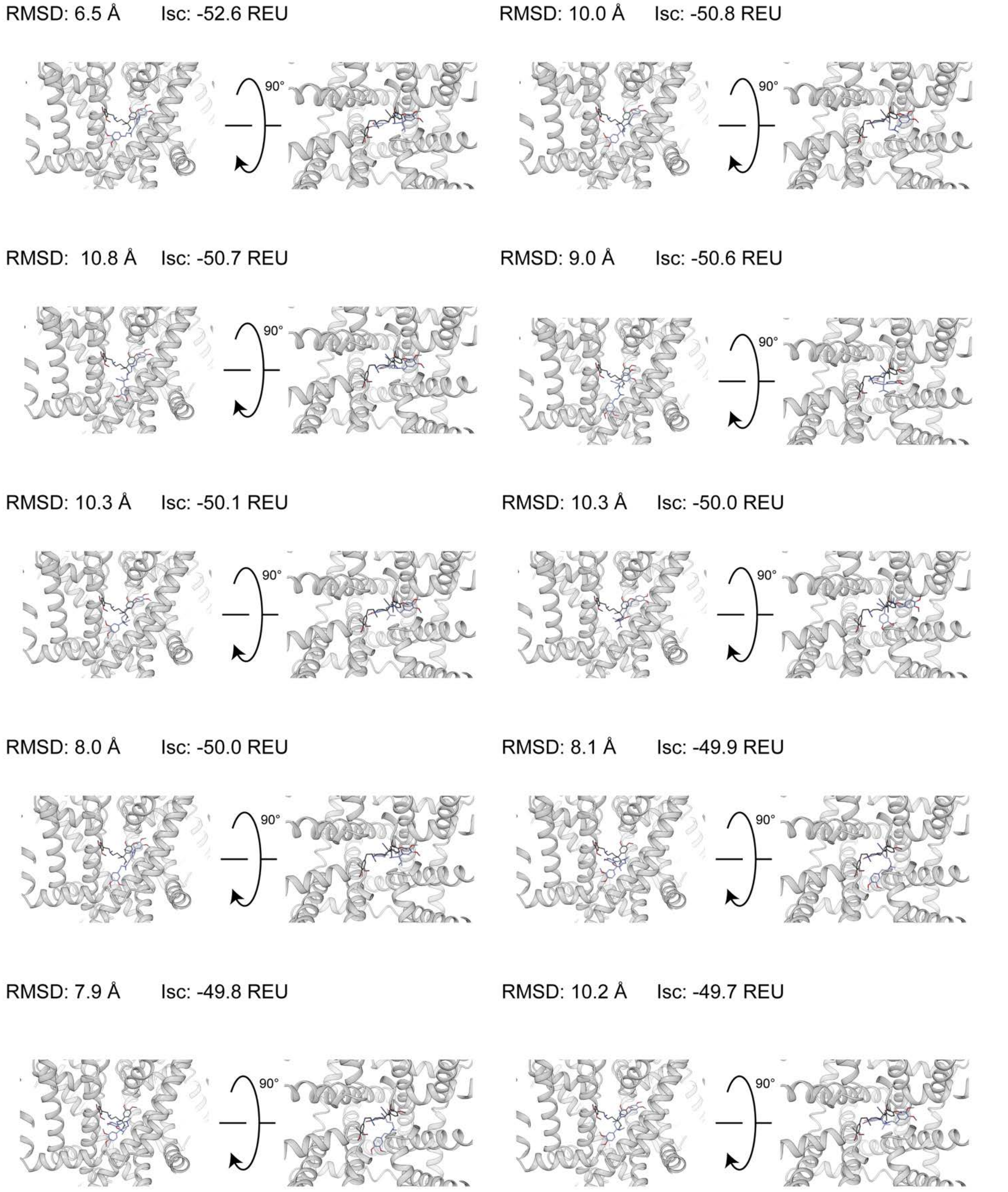

Figure S19.2. PDB 6JPA-2. Top 10 interface-scoring (`LigInterface`) poses from GALigandDock, 100,000 total poses, padding 7 Å.

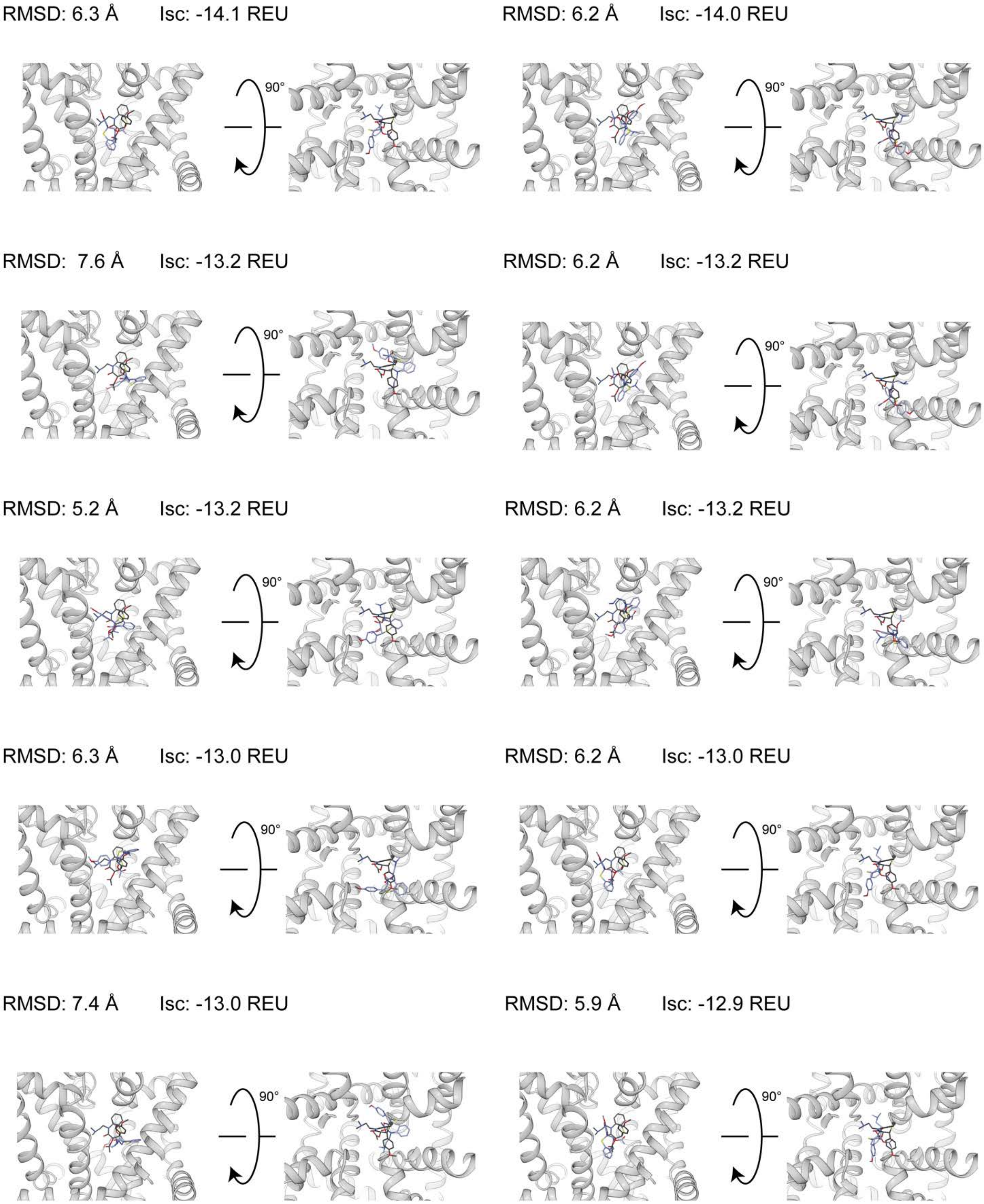

Figure S20.1. PDB 6JPB. Top 10 interface-scoring (`interface_delta_X`) poses from RosettaLigand, 100,000 total poses, all atom ligand interface.

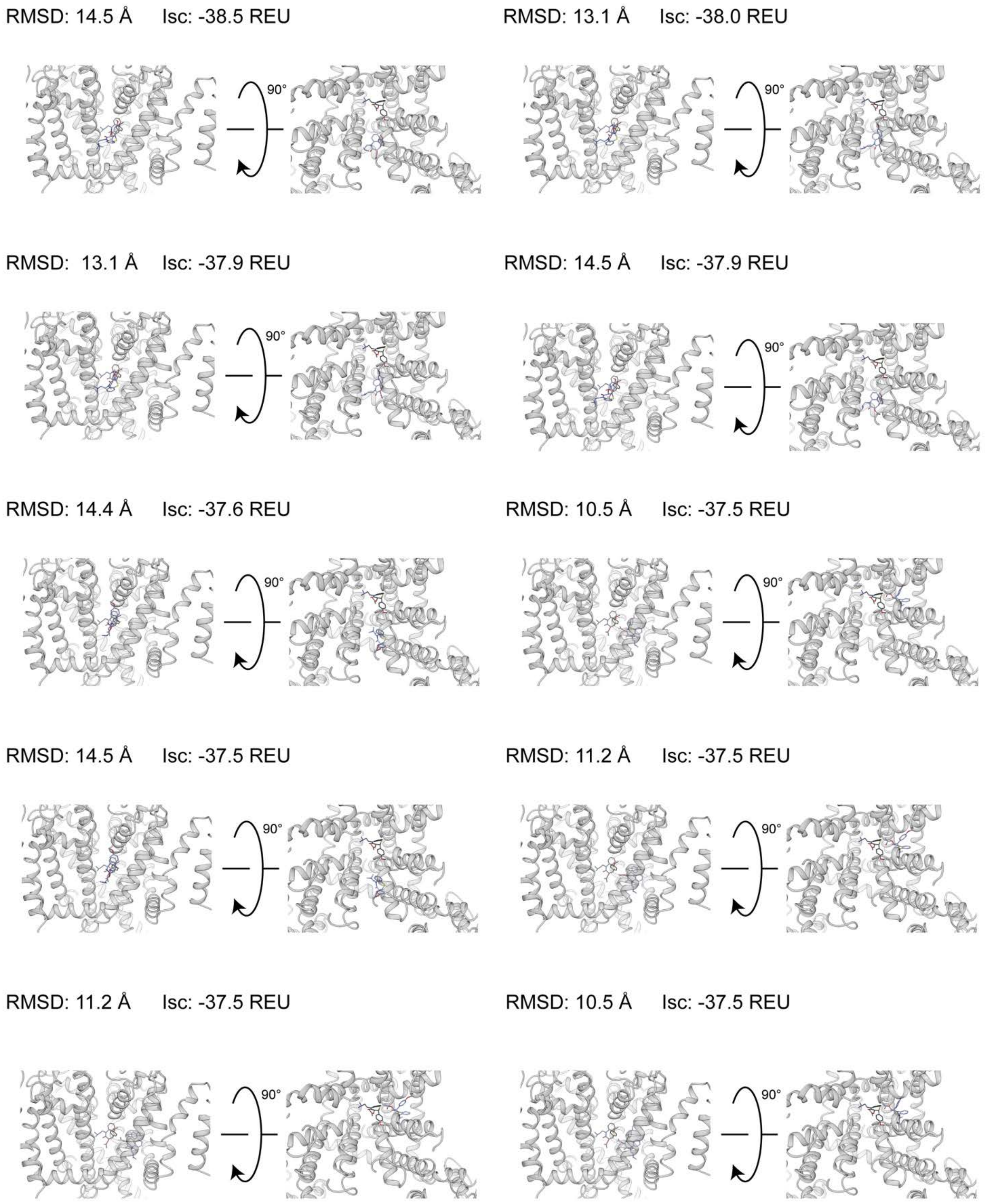

Figure S20.2. PDB 6JPB. Top 10 interface-scoring (`LigInterface`) poses from GALigandDock, 100,000 total poses, padding 7 Å.

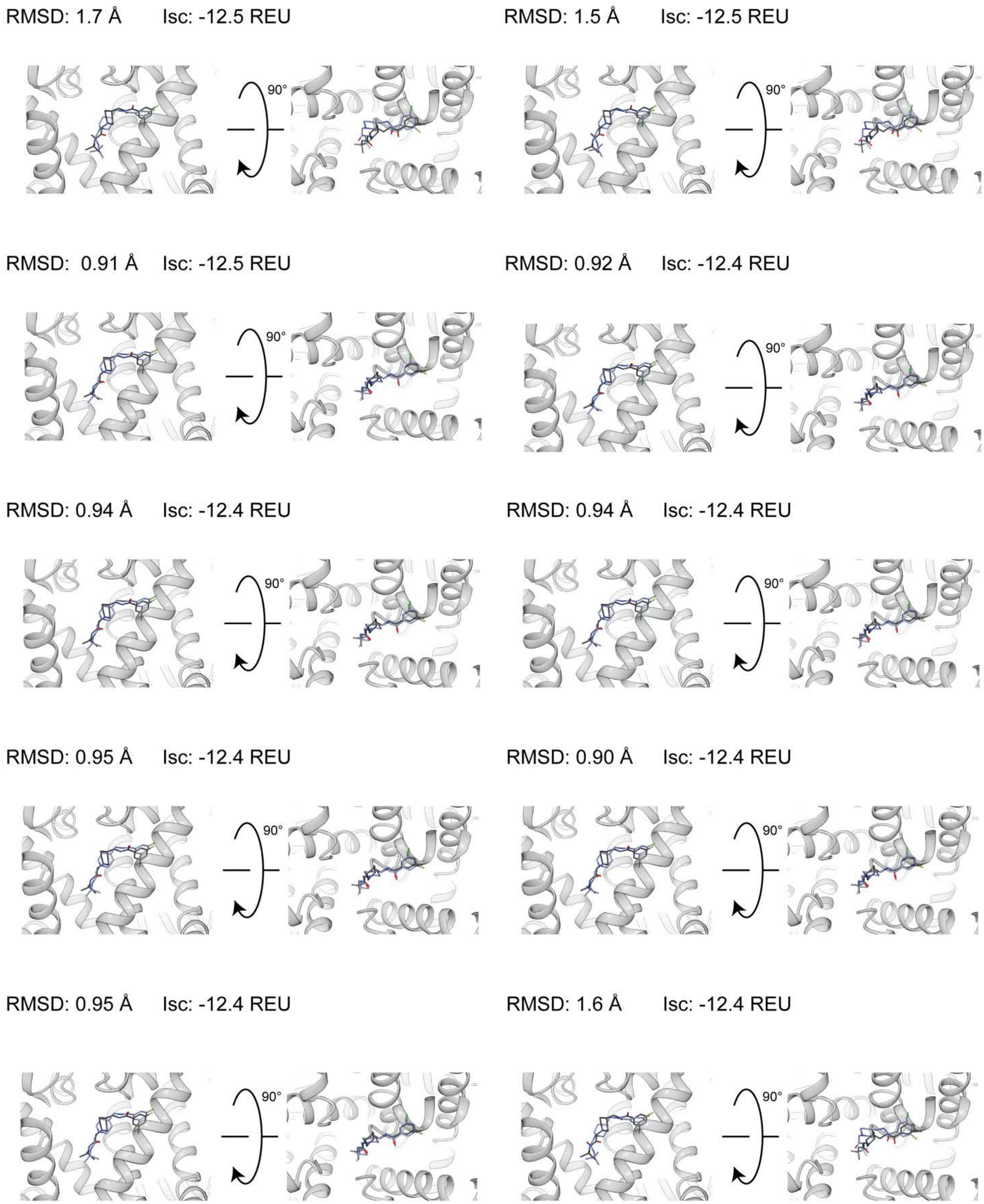

Figure S21.1. PDB 6KZP. Top 10 interface-scoring (`interface_delta_X`) poses from RosettaLigand, 100,000 total poses, all atom ligand interface.

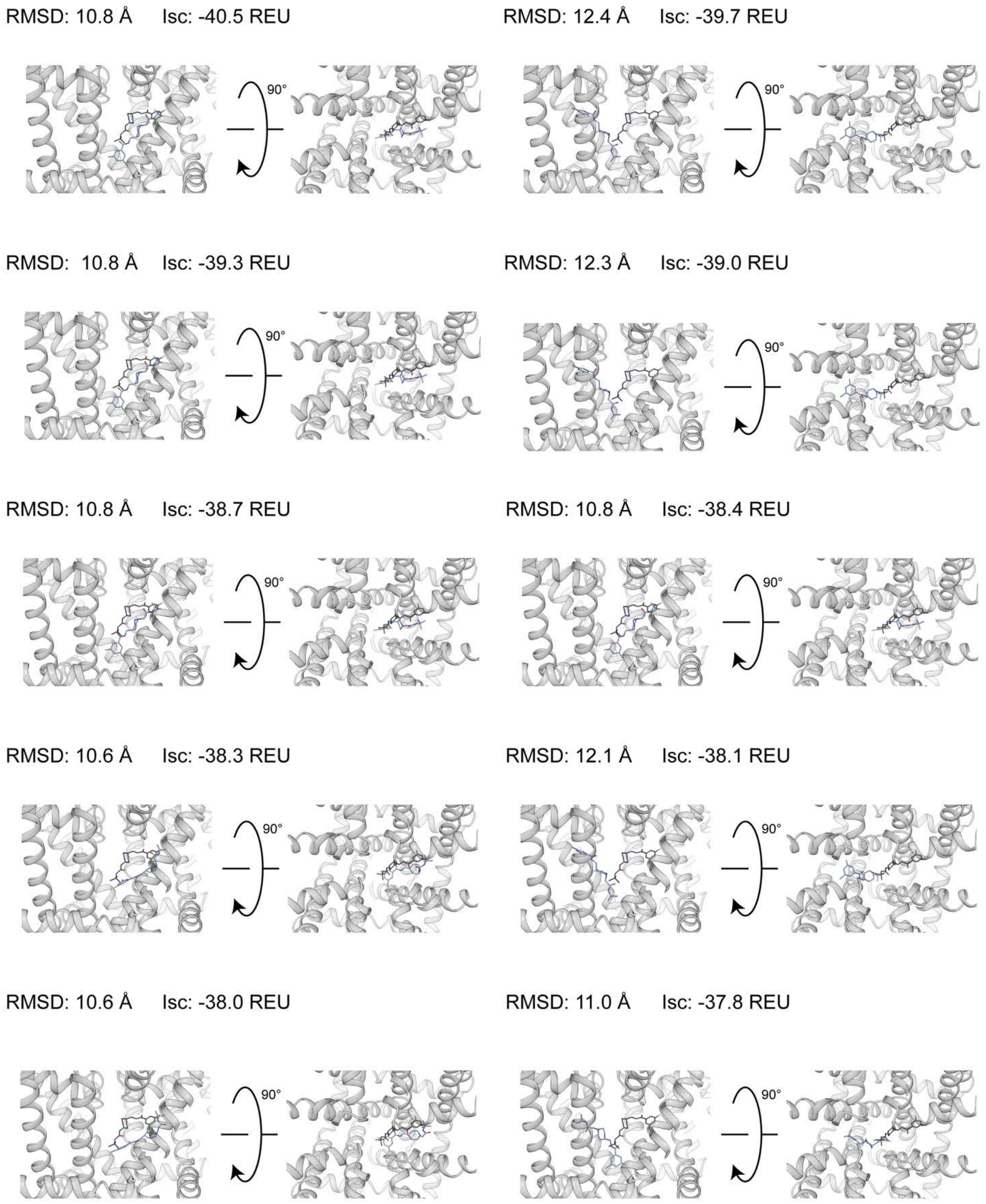

Figure S21.2. PDB 6KZP. Top 10 interface-scoring (`LigInterface`) poses from GALigandDock, 100,000 total poses, padding 7 Å.

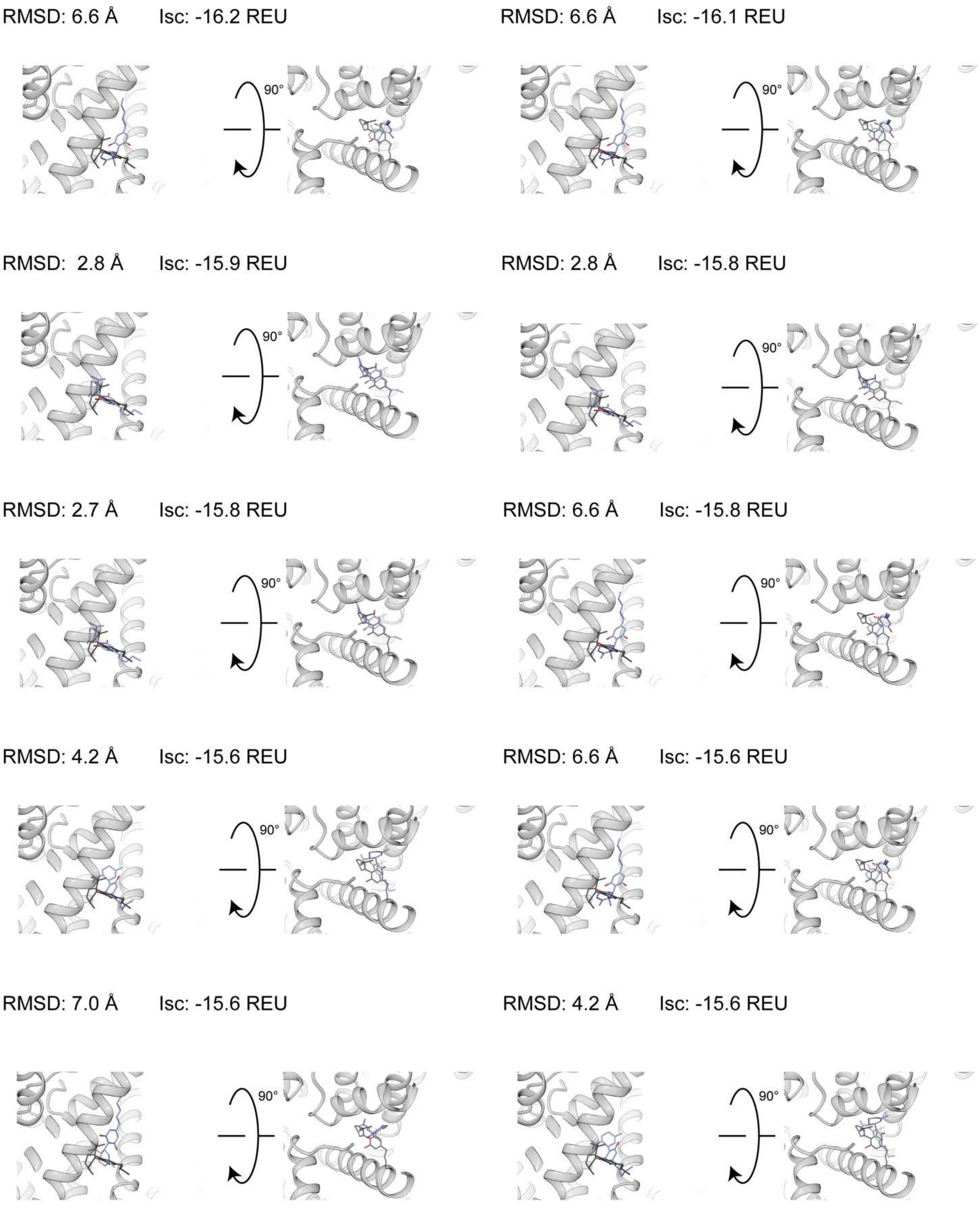

Figure S22.1. PDB 6U88. Top 10 interface-scoring (`interface_delta_X`) poses from RosettaLigand, 100,000 total poses, all atom ligand interface.

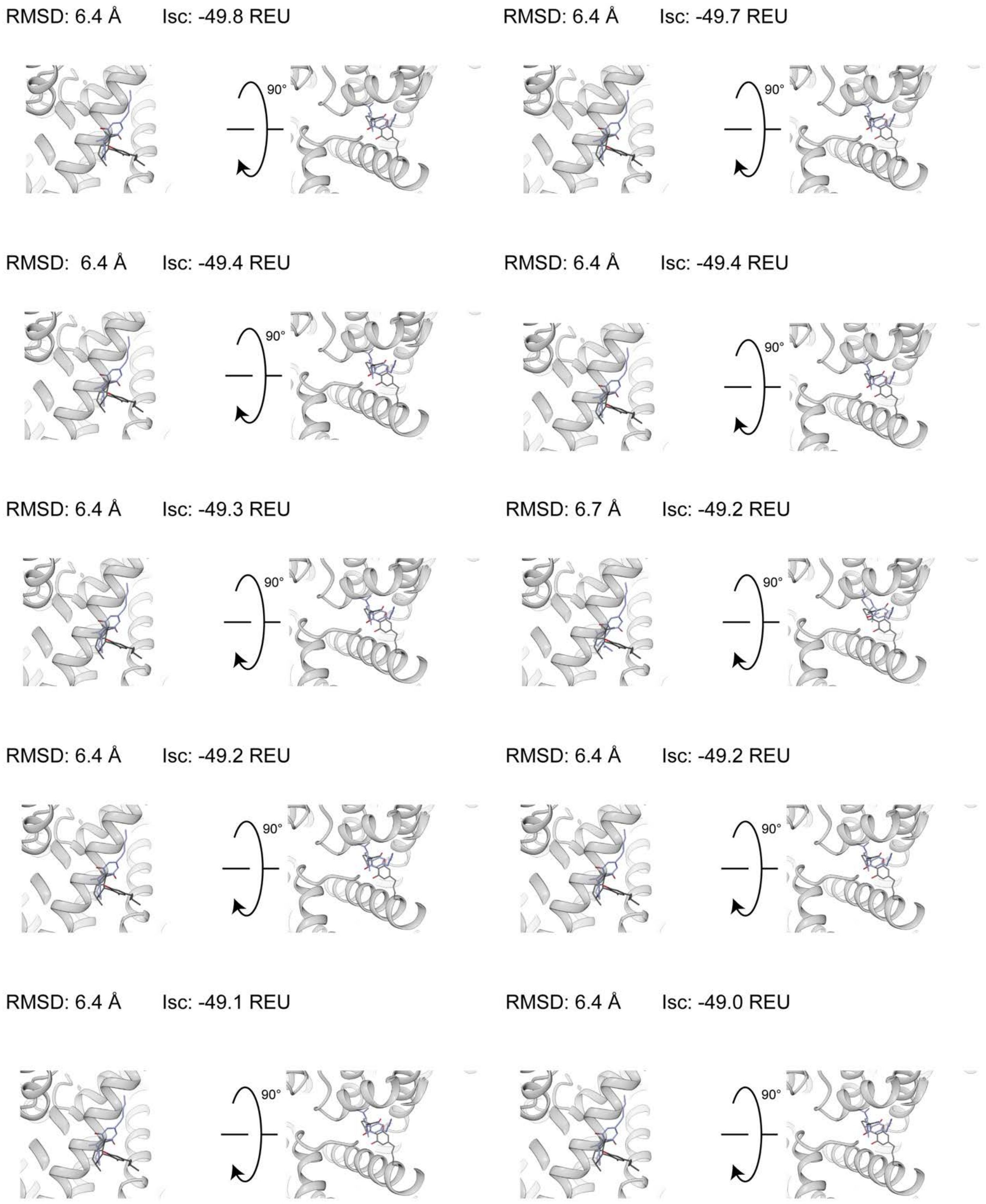

Figure S22.2. PDB 6U88. Top 10 interface-scoring (`LigInterface`) poses from GALigandDock, 100,000 total poses, padding 7 Å.

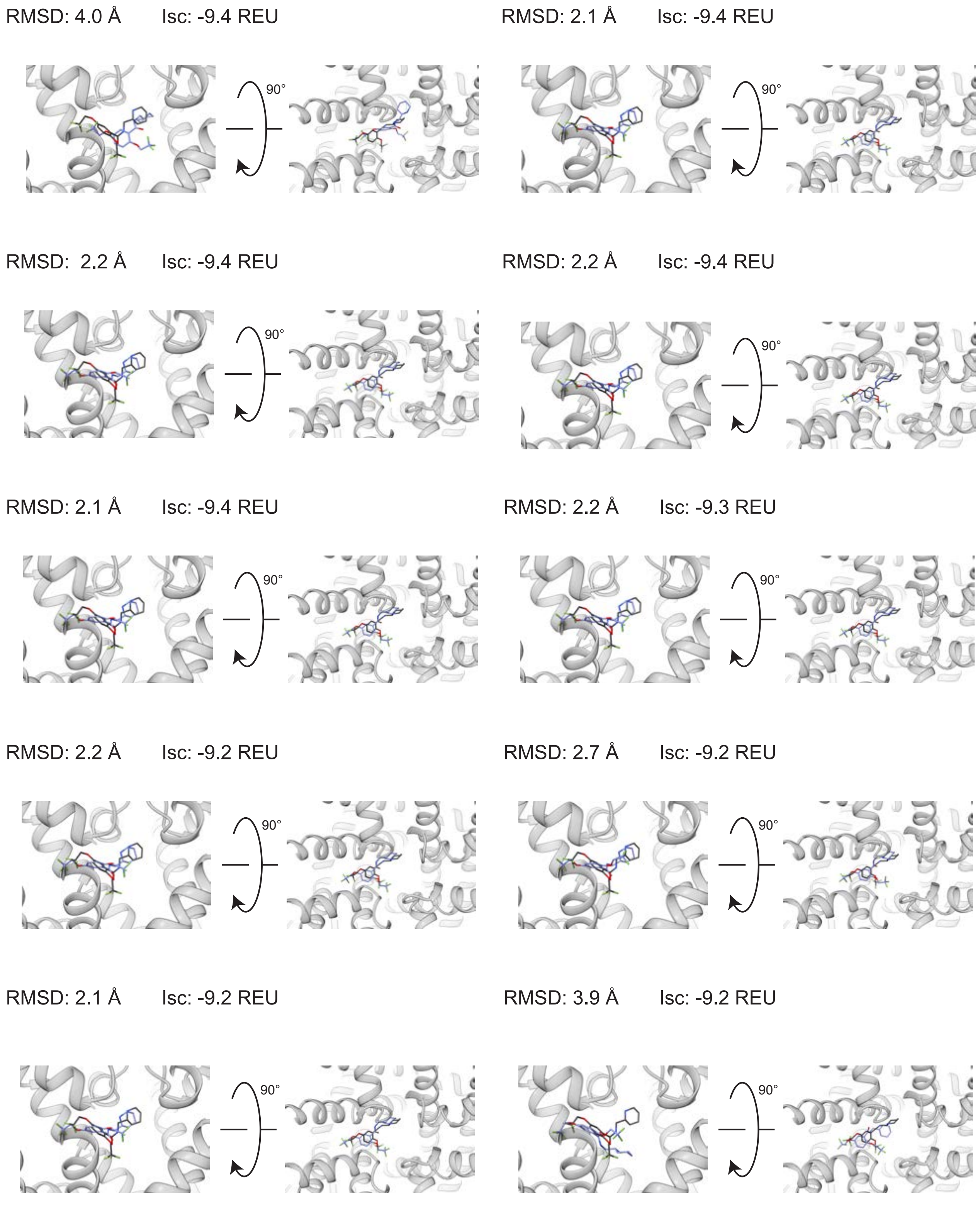

Figure S23.1. PDB 6UZ0. Top 10 interface-scoring (`interface_delta_X`) poses from RosettaLigand, 100,000 total poses, all atom ligand interface.

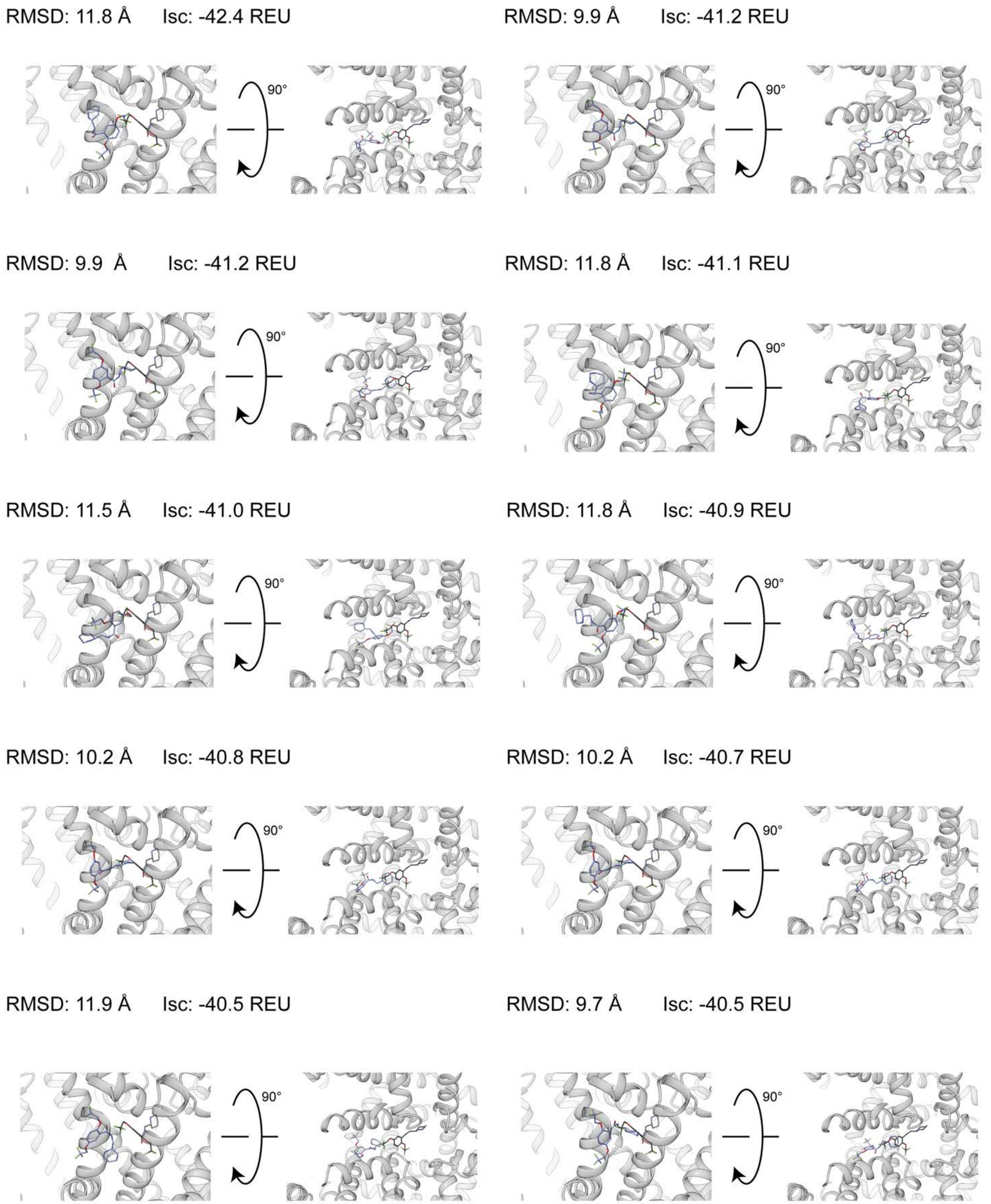

Figure S23.2. PDB 6UZ0. Top 10 interface-scoring (`LigInterface`) poses from GALigandDock, 100,000 total poses, padding 7 Å.

Table S1.1. Summary of minimum RMSD (Å) pose from each RosettaLigand docking set.

	All ligand atom cutoff		Neighbor atom cutoff	
**PDB ID**	**20,000 poses**	**100,000 poses**	**20,000 poses**	**100,000 poses**
**5EK0**	0.92	0.91	0.92	0.88
**6J8G**	0.71	0.70	0.70	0.66
**6J8I**	0.55	0.54	0.58	0.58
**6JP5**	0.96	0.77	0.94	0.85
**6JP8**	0.92	0.93	1.1	0.89
**6JPA-1**	1.4	1.4	1.5	1.3
**6JPA-2**	2.3	2.2	2.6	2.2
**6JPB**	2.3	2.5	2.5	1.9
**6KZP**	0.71	0.73	0.73	0.72
**6U88**	1.0	1.0	1.2	1.1
**6UZ0**	1.2	1.2	1.2	1.2
**Average**	1.2 ± 0.6	1.2 ± 0.6	1.3 ± 0.7	1.1 ± 0.5
**Median**	1.0	0.93	1.1	0.89

Table S1.2. Summary of minimum RMSD (Å) pose from each GALigandDock docking set.

	Padding 2 Å		Padding 4 Å		Padding 7 Å	
**PDB ID**	**20,000 poses**	**100,000 poses**	**20,000 poses**	**100,000 poses**	**20,000 poses**	**100,000 poses**
**5EK0**	0.51	0.56	0.56	0.57	0.71	0.65
**6J8G**	0.77	0.75	1.1	0.65	0.8	0.76
**6J8I**	0.96	0.94	0.96	0.83	0.81	0.81
**6JP5**	1.8	1.8	1.3	1.2	1.3	1.3
**6JP8**	1.2	1.2	1.0	1.0	1.1	0.97
**6JPA-1**	1.9	2.0	2.3	2.0	2.7	2.0
**6JPA-2**	2.7	2.4	2.4	2.4	2.3	2.1
**6JPB**	1.7	1.3	1.7	1.7	1.1	1.1
**6KZP**	0.83	0.83	0.84	0.83	0.81	0.84
**6U88**	1.3	1.4	1.4	1.4	0.99	0.90
**6UZ0**	1.5	1.3	1.5	1.4	1.2	0.94
**Average**	1.4 ± 0.6	1.3 ± 0.6	1.4 ± 0.6	1.3 ± 0.6	1.3 ± 0.6	1.1 ± 0.5
**Median**	1.3	1.3	1.3	1.2	1.1	0.94

Table S2.1. RMSD (Å) of closest pose to native structural coordinates and lowest interface energy (`interface_delta_X`) pose of the top 10 % of poses by total energy (`total_score`) for RosettaLigand, using all ligand atoms for interface scoring cutoff. †

	**All ligand atom cutoff, 20,000 poses**				**All ligand atom cutoff, 100,000 poses**			
	**Closest pose to native**		**Lowest Interface Energy**		**Closest pose to native**		**Lowest Interface Energy**	
**PDB ID**	**RMSD (Å)**	**Interface energy (REU)**	**RMSD (Å)**	**Interface energy (REU)**	**RMSD (Å)**	**Interface energy (REU)**	**RMSD (Å)**	**Interface energy (REU)**
**5EK0**	0.92	-13.5	2.8	-17.4	0.91	-11.1	2.7	-17.4
**6J8G**	0.71	-6.0	4.2	-14.8	0.7	-7.6	4.4	-14.4
**6J8I**	0.55	-2.7	4.1	-7.2	0.54	-4.5	4.1	-7.6
**6JP5**	0.96	-10.4	4.4	-13.2	0.77	-9.9	1.7	-13.7
**6JP8**	0.92	-9.5	1.2	-12.0	0.93	-9.7	1.2	-12.1
**6JPA-1**	1.4	-11.8	9.7	-17.5	1.4	-13.1	5.8	-17.8
**6JPA-2**	2.3	-7.5	9.5	-16.5	2.2	-10.0	10.8	-16.8
**6JPB**	2.3	-8.1	6.0	-12.9	2.5	-2.2	6.3	-14.1
**6KZP**	0.71	-8.9	0.91	-12.5	0.73	-10.2	1.7	-12.5
**6U88**	1.0	-9.6	6.0	-15.8	1.0	-6.0	6.6	-16.2
**6UZ0**	1.2	-7.7	3.4	-9.5	1.2	-4.5	4.0	-9.4
**Average**	1.2 ± 0.6	--	4.7 ± 2.9	--	1.2 ± 0.6	--	4.5 ± 2.8	--
**Median**	1.0	--	4.2	--	0.93	--	4.1	--

† Interface energy statistics were not computed since they are not standardized like RMSD.

Table S2.2. RMSD (Å) of closest pose to native structural coordinates and lowest interface energy (`interface_delta_X`) pose of the top 10 % of poses by total energy (`total_score`) from RosettaLigand with ligand neighbor atom for interface scoring cutoff. †

	Neighbor atom cutoff, 20,000 poses				Neighbor atom cutoff, 100,000 poses			
	Closest pose to native		Lowest Interface Energy		Closest pose to native		Lowest Interface Energy	
PDB ID	RMSD (Å)	Interface energy (REU)	RMSD (Å)	Interface energy (REU)	RMSD (Å)	Interface energy (REU)	RMSD (Å)	Interface energy (REU)
**5EK0**	0.92	-7.8	2.6	-16.7	0.88	-7.7	2.5	-17.9
**6J8G**	0.70	-7.3	4.2	-15.2	0.66	-5.9	4.2	-15.4
**6J8I**	0.58	-4.7	4.2	-7.5	0.58	-4.3	4.1	-7.9
**6JP5**	0.94	-10.3	1.8	-13.5	0.85	-7.6	1.5	-14.0
**6JP8**	1.1	-10.1	1.2	-12.2	0.89	-9.9	1.3	-12.5
**6JPA-1**	1.5	-11.5	7.7	-16.6	1.3	-12.5	5.2	-16.8
**6JPA-2**	2.6	-5.0	6.6	-16.6	2.2	-9.2	9.5	-16.1
**6JPB**	2.5	-6.1	6.2	-14.5	1.9	-7.8	6.0	-13.9
**6KZP**	0.73	-9.6	1.0	-12.5	0.72	-8.5	6.5	-13.1
**6U88**	1.2	-9.5	6.1	-14.7	1.1	-9.2	6.6	-16.4
**6UZ0**	1.2	-7.7	4.0	-8.9	1.2	-7.8	9.1	-10.2
**Average**	1.3 ± 0.7	--	4.1 ± 2.3	--	1.1 ± 0.5	--	5.1 ± 2.8	--
**Median**	1.1	--	4.2	--	0.89	--	5.2	--

† Interface energy statistics were not computed since they are not standardized like RMSD.

Table S2.3. RMSD (Å) of closest pose to native structural coordinates and lowest interface energy (`LigInterface`) pose from GALigandDock, using the top 10 % of poses by total energy (`total_score`) and 2 Å padding. †

	2 Å padding, 20,000 poses				2 Å padding, 100,000 poses			
	Closest pose to native		Lowest Interface Energy		Closest pose to native		Lowest Interface Energy	
**PDB ID**	**RMSD (Å)**	**Interface energy (REU)**	**RMSD (Å)**	**Interface energy (REU)**	**RMSD (Å)**	**Interface energy (REU)**	**RMSD (Å)**	**Interface energy (REU)**
**5EK0**	0.51	-36.0	0.72	-38.5	0.56	-36.4	1.3	-38.8
**6J8G**	0.77	-18.7	3.8	-29.5	0.75	-18.9	3.8	-31.4
**6J8I**	0.96	-12.2	6.2	-30.0	0.94	-12.3	6.2	-29.9
**6JP5**	1.8	-20.6	4.7	-30.6	1.8	-20.5	5.3	-31.5
**6JP8**	1.2	-11.7	6.1	-25.3	1.2	-13.4	6.1	-25.3
**6JPA-1**	1.9	-37.8	5.6	-47.6	2.0	-38.3	5.4	-48.8
**6JPA-2**	2.7	-35.6	11.8	-48.2	2.4	-30.9	11.7	-48.2
**6JPB**	1.7	-20.8	4.7	-30.7	1.3	-18.2	4.6	-30.8
**6KZP**	0.83	-32.4	10.3	-32.6	0.83	-33.4	0.86	-33.4
**6U88**	1.3	-37.6	6.4	-45.4	1.4	-32.4	6.4	-45.5
**6UZ0**	1.5	-31.6	4.9	-37.2	1.3	-28.0	7.1	-37.3
**Average**	1.4 ± 0.6	--	5.9 ± 3.0	--	1.3 ± 0.6	--	5.3 ± 2.9	--
**Median**	1.3	--	5.6	--	1.3	--	5.4	--

† Interface energy statistics were not computed since they are not standardized like RMSD.

Table S2.4. RMSD (Å) of closest pose to native structural coordinates and lowest interface energy (`LigInterface`) pose from GALigandDock, using the top 10 % of poses by total energy (`total_score`) and 4 Å padding. †

PDB ID	4 Å padding, 20,000 poses				4 Å padding, 100,000 poses			
	Closest pose to native		Lowest Interface Energy		Closest pose to native		Lowest Interface Energy	
	RMSD (Å)	Interface energy (REU)	RMSD (Å)	Interface energy (REU)	RMSD (Å)	Interface energy (REU)	RMSD (Å)	Interface energy (REU)
**5EK0**	0.56	-37.1	0.74	-38.7	0.57	-37.0	0.66	-38.7
**6J8G**	1.1	-20.6	3.8	-30.6	0.65	-21.4	3.8	-33.2
**6J8I**	0.96	-18.1	3.9	-33.9	0.83	-17.4	2.2	-34.9
**6JP5**	1.3	-32.3	1.3	-35.2	1.2	-29.7	1.3	-35.1
**6JP8**	1.0	-30.4	1.1	-32.4	1.0	-30.5	1.1	-32.4
**6JPA-1**	2.3	-39.3	6.7	-48.6	2.0	-38.7	10.8	-49.2
**6JPA-2**	2.4	-32.7	9.8	-47.7	2.4	-32.7	11.1	-50.4
**6JPB**	1.7	-18.4	5.7	-35.4	1.7	-20.2	5.7	-35.7
**6KZP**	0.84	-31.1	10.2	-34.1	0.83	-30.8	10.8	-38.6
**6U88**	1.4	-41.4	6.3	-47.6	1.4	-36.0	6.3	-48.5
**6UZ0**	1.5	-30.6	10.0	-38.4	1.4	-29.2	11.3	-38.6
**Average**	1.4 ± 0.6	--	5.4 ± 3.6	--	1.3 ± 0.6	--	5.9 ± 4.4	--
**Median**	1.3	--	5.7	--	1.2	--	5.7	--

† Interface energy statistics were not computed since they are not standardized like RMSD.

Table S2.5. RMSD (Å) of closest pose to native structural coordinates and lowest interface energy (`LigInterface`) pose from GALigandDock, using the top 10 % of poses by total energy (`total_score`) and 7 Å padding. †

	7 Å padding, 20,000 poses				7 Å padding, 100,000 poses			
	Closest pose to native		Lowest Interface Energy		Closest pose to native		Lowest Interface Energy	
**PDB ID**	**RMSD (Å)**	**Interface energy (REU)**	**RMSD (Å)**	**Interface energy (REU)**	**RMSD (Å)**	**Interface energy (REU)**	**RMSD (Å)**	**Interface energy (REU)**
**5EK0**	0.71	-36.1	0.74	-39.0	0.65	-38.7	0.83	-40.4
**6J8G**	0.80	-21.9	4.0	-32.5	0.76	-23.8	4.0	-32.4
**6J8I**	0.81	-19.1	3.9	-34.2	0.81	-18.9	2.2	-37.5
**6JP5**	1.3	-34.4	1.3	-34.4	1.3	-35.4	1.3	-35.4
**6JP8**	1.1	-30.7	1.1	-33.9	0.97	-30.9	1.1	-34.5
**6JPA-1**	2.7	-47.6	3.6	-49.1	2.0	-39.0	11.4	-51.6
**6JPA-2**	2.3	-33.4	9.0	-51.0	2.1	-31.9	6.5	-52.6
**6JPB**	1.1	-20.1	14.6	-38.0	1.1	-20.4	14.5	-38.5
**6KZP**	0.81	-31.2	10.7	-39.2	0.84	-32.1	10.8	-40.5
**6U88**	0.99	-42.5	6.4	-49.8	0.9	-41.1	6.4	-49.8
**6UZ0**	1.2	-30.6	11.8	-41.9	0.94	-31.3	11.8	-42.4
**Average**	1.3 ± 0.6	--	6.1 ± 4.8	--	1.1 ± 0.5	--	6.4 ± 5.0	--
**Median**	1.1	--	4.0	--	0.94	--	6.4	--

† Interface energy statistics were not computed since they are not standardized like RMSD.

Table S3. Pearson's correlation coefficient (r), confidence interval, and associated two-tailed p-values for covariates compared. Null hypothesis: there is no correlation between covariate 1 and covariate 2. n=10, α=0.05. Statistically significant values denoted with asterisk (*).

Covariate 1	Covariate 2	Pearson's r	Confidence Interval	p-value
Total ligand rotatable bonds	Total ligand heavy atoms	0.73	[0.19, 0.93]	0.016*
Total ligand rotatable bonds	Ligand molecular weight (Da)	0.66	[0.06, 0.91]	0.036*
Total ligand rotatable bonds	Structure resolution (Å)	-0.26	[-0.76, 0.45]	0.47
Total ligand rotatable bonds	Log10 RosettaLigand conformers	0.74	[0.21, 0.93]	0.014*
Total ligand heavy atoms	Ligand molecular weight (Da)	0.99	[0.96, 1.0]	$3.9 \bullet 10^{-8}$*
Total ligand heavy atoms	Structure resolution (Å)	0.12	[-0.55, 0.7]	0.74
Total ligand heavy atoms	Log10 RosettaLigand conformers	0.67	[0.07, 0.91]	0.035*
Ligand molecular weight (Da)	Structure resolution (Å)	0.21	[-0.48, 0.74]	0.56
Ligand molecular weight (Da)	Log10 RosettaLigand conformers	0.68	[0.10, 0.92]	0.029*
Structure resolution (Å)	Log10 RosettaLigand conformers	0.29	[-0.41, 0.78]	0.41

Table S4.1. P_{Near} of RMSD vs `interface_delta_X` for RosettaLigand, all poses, all ligand atom mode.

	$k_BT=1.0$						$k_BT=0.62$					
	**20,000 poses**			**100,000 poses**			**20,000 poses**			**100,000 poses**		
**PDB ID**	**λ=1.0**	**λ=1.5**	**λ=2.0**	**λ=1.0**	**λ=1.5**	**λ=2.0**	**λ=1.0**	**λ=1.5**	**λ=2.0**	**λ=1.0**	**λ=1.5**	**λ=2.0**
**5EK0**	$1.8\cdot10^{-2}$	$8.2\cdot10^{-2}$	0.19	$2.5\cdot10^{-2}$	$8.9\cdot10^{-2}$	0.18	$1.0\cdot10^{-2}$	$7.2\cdot10^{-2}$	0.20	$2.6\cdot10^{-2}$	0.10	0.22
**6J8G**	$4.2\cdot10^{-3}$	$1.0\cdot10^{-2}$	$2.5\cdot10^{-2}$	$2.8\cdot10^{-3}$	$9.0\cdot10^{-3}$	$2.4\cdot10^{-2}$	$1.6\cdot10^{-3}$	$3.2\cdot10^{-3}$	$1.4\cdot10^{-2}$	$5.2\cdot10^{-4}$	$1.8\cdot10^{-3}$	$1.2\cdot10^{-2}$
**6J8I**	$5.0\cdot10^{-2}$	$7.9\cdot10^{-2}$	0.11	$4.9\cdot10^{-2}$	$7.7\cdot10^{-2}$	0.11	$5.3\cdot10^{-2}$	$8.4\cdot10^{-2}$	0.12	$5.0\cdot10^{-2}$	$8.0\cdot10^{-2}$	0.12
**6JP5**	$3.3\cdot10^{-2}$	$9.4\cdot10^{-2}$	0.16	$3.3\cdot10^{-2}$	$9.4\cdot10^{-2}$	0.16	$5.0\cdot10^{-2}$	0.14	0.21	$5.1\cdot10^{-2}$	0.14	0.21
**6JP8**	0.14	0.37	0.51	0.14	0.36	0.51	0.19	0.45	0.62	0.19	0.45	0.62
**6JPA-1**	$6.3\cdot10^{-5}$	$8.0\cdot10^{-4}$	$6.5\cdot10^{-3}$	$8.7\cdot10^{-5}$	$9.1\cdot10^{-4}$	$6.6\cdot10^{-3}$	$1.3\cdot10^{-5}$	$3.9\cdot10^{-4}$	$5.2\cdot10^{-3}$	$1.9\cdot10^{-5}$	$4.0\cdot10^{-4}$	$4.7\cdot10^{-3}$
**6JPA-2**	$3.2\cdot10^{-7}$	$5.5\cdot10^{-5}$	$6.3\cdot10^{-4}$	$3.8\cdot10^{-7}$	$6.8\cdot10^{-5}$	$7.0\cdot10^{-4}$	$1.8\cdot10^{-7}$	$3.6\cdot10^{-5}$	$4.5\cdot10^{-4}$	$2.9\cdot10^{-7}$	$5.7\cdot10^{-5}$	$5.0\cdot10^{-4}$
**6JPB**	$1.9\cdot10^{-7}$	$4.4\cdot10^{-5}$	$1.1\cdot10^{-3}$	$8.9\cdot10^{-8}$	$4.4\cdot10^{-5}$	$1.1\cdot10^{-3}$	$5.1\cdot10^{-8}$	$2.1\cdot10^{-5}$	$7.7\cdot10^{-4}$	$3.1\cdot10^{-8}$	$1.9\cdot10^{-5}$	$6.8\cdot10^{-4}$
**6KZP**	0.10	0.23	0.34	$9.8\cdot10^{-2}$	0.23	0.34	0.18	0.36	0.50	0.17	0.36	0.50
**6U88**	$5.0\cdot10^{-3}$	$2.9\cdot10^{-2}$	$7.9\cdot10^{-2}$	$5.3\cdot10^{-3}$	$3.0\cdot10^{-2}$	$8.1\cdot10^{-2}$	$2.5\cdot10^{-3}$	$2.2\cdot10^{-2}$	$7.0\cdot10^{-2}$	$3.9\cdot10^{-3}$	$2.9\cdot10^{-2}$	$8.3\cdot10^{-2}$
**6UZ0**	$7.4\cdot10^{-3}$	$5.3\cdot10^{-2}$	0.14	$7.4\cdot10^{-3}$	$5.3\cdot10^{-2}$	0.14	$8.1\cdot10^{-3}$	$5.8\cdot10^{-2}$	0.15	$8.0\cdot10^{-3}$	$5.6\cdot10^{-2}$	0.14

Table S4.2. P_{Near} of RMSD vs `interface_delta_X` for RosettaLigand, 10% lowest poses by `total_score`, all ligand atom mode.

	k_BT=1.0						k_BT=0.62					
	20,000 poses			100,000 poses			20,000 poses			100,000 poses		
**PDB ID**	**λ=1.0**	**λ=1.5**	**λ=2.0**	**λ=1.0**	**λ=1.5**	**λ=2.0**	**λ=1.0**	**λ=1.5**	**λ=2.0**	**λ=1.0**	**λ=1.5**	**λ=2.0**
**5EK0**	$2.1\cdot10^{-2}$	$9.5\cdot10^{-2}$	0.21	$4.1\cdot10^{-2}$	0.13	0.24	$1.0\cdot10^{-2}$	$7.3\cdot10^{-2}$	0.20	$4.0\cdot10^{-2}$	0.13	0.26
**6J8G**	$1.4\cdot10^{-4}$	$1.3\cdot10^{-3}$	$1.3\cdot10^{-2}$	$3.7\cdot10^{-4}$	$2.1\cdot10^{-3}$	$1.4\cdot10^{-2}$	$1.3\cdot10^{-5}$	$4.0\cdot10^{-4}$	$1.0\cdot10^{-2}$	$9.1\cdot10^{-5}$	$6.8\cdot10^{-4}$	$9.3\cdot10^{-3}$
**6J8I**	$3.0\cdot10^{-2}$	$4.7\cdot10^{-2}$	$7.1\cdot10^{-2}$	$3.2\cdot10^{-2}$	$5.1\cdot10^{-2}$	$7.8\cdot10^{-2}$	$2.9\cdot10^{-2}$	$4.7\cdot10^{-2}$	$7.5\cdot10^{-2}$	$2.7\cdot10^{-2}$	$4.4\cdot10^{-2}$	$7.8\cdot10^{-2}$
**6JP5**	$6.4\cdot10^{-2}$	0.17	0.25	$6.2\cdot10^{-2}$	0.16	0.24	$7.5\cdot10^{-2}$	0.19	0.28	$7.6\cdot10^{-2}$	0.19	0.28
**6JP8**	0.18	0.45	0.62	0.18	0.45	0.62	0.20	0.48	0.66	0.20	0.48	0.66
**6JPA-1**	$2.3\cdot10^{-5}$	$5.6\cdot10^{-4}$	$6.2\cdot10^{-3}$	$5.9\cdot10^{-5}$	$6.5\cdot10^{-4}$	$5.9\cdot10^{-3}$	$3.1\cdot10^{-6}$	$3.1\cdot10^{-4}$	$5.0\cdot10^{-3}$	$1.0\cdot10^{-5}$	$2.9\cdot10^{-4}$	$4.3\cdot10^{-3}$
**6JPA-2**	$5.2\cdot10^{-9}$	$1.3\cdot10^{-5}$	$2.6\cdot10^{-4}$	$6.5\cdot10^{-9}$	$9.7\cdot10^{-6}$	$2.1\cdot10^{-4}$	$3.2\cdot10^{-9}$	$9.6\cdot10^{-6}$	$2.0\cdot10^{-4}$	$2.0\cdot10^{-9}$	$5.7\cdot10^{-6}$	$1.2\cdot10^{-4}$
**6JPB**	$3.7\cdot10^{-8}$	$3.2\cdot10^{-5}$	$9.2\cdot10^{-4}$	$5.3\cdot10^{-8}$	$3.3\cdot10^{-5}$	$9.6\cdot10^{-4}$	$1.2\cdot10^{-8}$	$1.5\cdot10^{-5}$	$6.6\cdot10^{-4}$	$1.6\cdot10^{-8}$	$1.4\cdot10^{-5}$	$6.4\cdot10^{-4}$
**6KZP**	0.19	0.40	0.55	0.18	0.39	0.54	0.25	0.48	0.63	0.24	0.47	0.62
**6U88**	$3.0\cdot10^{-4}$	$9.4\cdot10^{-3}$	$4.2\cdot10^{-2}$	$4.8\cdot10^{-4}$	$8.9\cdot10^{-3}$	$3.9\cdot10^{-2}$	$2.7\cdot10^{-4}$	$1.0\cdot10^{-2}$	$4.6\cdot10^{-2}$	$4.1\cdot10^{-4}$	$9.8\cdot10^{-3}$	$4.3\cdot10^{-2}$
**6UZ0**	$6.9\cdot10^{-3}$	$4.9\cdot10^{-2}$	0.13	$4.5\cdot10^{-3}$	$4.1\cdot10^{-2}$	0.11	$7.4\cdot10^{-3}$	$5.3\cdot10^{-2}$	0.14	$4.9\cdot10^{-3}$	$4.5\cdot10^{-2}$	0.12

Table S4.3. P_{Near} of RMSD vs `interface_delta_X` for RosettaLigand, all poses, neighbor ligand atom mode.

	k_BT=1.0						k_BT=0.62					
	20,000 poses			100,000 poses			20,000 poses			100,000 poses		
**PDB ID**	**λ=1.0**	**λ=1.5**	**λ=2.0**	**λ=1.0**	**λ=1.5**	**λ=2.0**	**λ=1.0**	**λ=1.5**	**λ=2.0**	**λ=1.0**	**λ=1.5**	**λ=2.0**
**5EK0**	$9.2\cdot10^{-3}$	$6.0\cdot10^{-2}$	0.16	$1.7\cdot10^{-2}$	$7.5\cdot10^{-2}$	0.17	$5.5\cdot10^{-3}$	$6.2\cdot10^{-2}$	0.19	$1.0\cdot10^{-2}$	$6.9\cdot10^{-2}$	0.19
**6J8G**	$5.6\cdot10^{-3}$	$1.5\cdot10^{-2}$	$3.3\cdot10^{-2}$	$4.0\cdot10^{-3}$	$1.1\cdot10^{-2}$	$2.7\cdot10^{-2}$	$1.1\cdot10^{-3}$	$3.1\cdot10^{-3}$	$1.5\cdot10^{-2}$	$7.4\cdot10^{-4}$	$2.0\cdot10^{-3}$	$1.2\cdot10^{-2}$
**6J8I**	$6.1\cdot10^{-2}$	$9.4\cdot10^{-2}$	0.12	$5.9\cdot10^{-2}$	$9.0\cdot10^{-2}$	0.12	$6.8\cdot10^{-2}$	0.10	0.14	$5.9\cdot10^{-2}$	$9.2\cdot10^{-2}$	0.13
**6JP5**	$2.5\cdot10^{-2}$	$7.6\cdot10^{-2}$	0.13	$2.6\cdot10^{-2}$	$7.7\cdot10^{-2}$	0.13	$3.6\cdot10^{-2}$	0.11	0.18	$4.1\cdot10^{-2}$	0.11	0.18
**6JP8**	0.14	0.36	0.51	0.15	0.37	0.51	0.19	0.46	0.62	0.19	0.46	0.63
**6JPA-1**	$2.2\cdot10^{-4}$	$1.4\cdot10^{-3}$	$6.7\cdot10^{-3}$	$1.6\cdot10^{-4}$	$1.1\cdot10^{-3}$	$5.8\cdot10^{-3}$	$8.7\cdot10^{-5}$	$6.8\cdot10^{-4}$	$4.2\cdot10^{-3}$	$5.3\cdot10^{-5}$	$4.8\cdot10^{-4}$	$3.5\cdot10^{-3}$
**6JPA-2**	$5.8\cdot10^{-8}$	$2.2\cdot10^{-5}$	$3.6\cdot10^{-4}$	$2.1\cdot10^{-7}$	$2.8\cdot10^{-5}$	$3.8\cdot10^{-4}$	$1.3\cdot10^{-8}$	$9.6\cdot10^{-6}$	$2.0\cdot10^{-4}$	$3.7\cdot10^{-8}$	$8.5\cdot10^{-6}$	$1.7\cdot10^{-4}$
**6JPB**	$1.0\cdot10^{-7}$	$3.8\cdot10^{-5}$	$1.0\cdot10^{-3}$	$2.8\cdot10^{-7}$	$4.7\cdot10^{-5}$	$1.1\cdot10^{-3}$	$2.6\cdot10^{-8}$	$1.5\cdot10^{-5}$	$5.8\cdot10^{-4}$	$7.7\cdot10^{-8}$	$1.9\cdot10^{-5}$	$6.3\cdot10^{-4}$
**6KZP**	$6.2\cdot10^{-2}$	0.17	0.28	$5.7\cdot10^{-2}$	0.16	0.27	0.11	0.26	0.40	$9.4\cdot10^{-2}$	0.24	0.37
**6U88**	$1.2\cdot10^{-3}$	$1.9\cdot10^{-2}$	$6.5\cdot10^{-2}$	$1.4\cdot10^{-3}$	$2.0\cdot10^{-2}$	$6.5\cdot10^{-2}$	$1.7\cdot10^{-3}$	$2.7\cdot10^{-2}$	$7.9\cdot10^{-2}$	$2.0\cdot10^{-3}$	$2.5\cdot10^{-2}$	$7.0\cdot10^{-2}$
**6UZ0**	$7.4\cdot10^{-3}$	$5.0\cdot10^{-2}$	0.13	$6.9\cdot10^{-3}$	$4.8\cdot10^{-2}$	0.13	$8.0\cdot10^{-3}$	$5.3\cdot10^{-2}$	0.13	$7.2\cdot10^{-3}$	$4.9\cdot10^{-2}$	0.13

Table S4.4. P_{Near} of RMSD vs `interface_delta_X` for RosettaLigand, 10% lowest poses by `total_score`, neighbor atom mode.

	$k_BT=1.0$						$k_BT=0.62$					
	**20,000 poses**			**100,000 poses**			**20,000 poses**			**100,000 poses**		
**PDB ID**	**λ=1.0**	**λ=1.5**	**λ=2.0**	**λ=1.0**	**λ=1.5**	**λ=2.0**	**λ=1.0**	**λ=1.5**	**λ=2.0**	**λ=1.0**	**λ=1.5**	**λ=2.0**
**5EK0**	$1.5\cdot10^{-2}$	$7.8\cdot10^{-2}$	0.18	$3.1\cdot10^{-2}$	0.11	0.22	$7.4\cdot10^{-3}$	$6.8\cdot10^{-2}$	0.20	$1.6\cdot10^{-2}$	$8.2\cdot10^{-2}$	0.21
**6J8G**	$1.4\cdot10^{-3}$	$4.1\cdot10^{-3}$	$1.6\cdot10^{-2}$	$5.5\cdot10^{-4}$	$2.2\cdot10^{-3}$	$1.3\cdot10^{-2}$	$4.0\cdot10^{-4}$	$1.3\cdot10^{-3}$	$1.2\cdot10^{-2}$	$9.1\cdot10^{-5}$	$5.8\cdot10^{-4}$	$1.0\cdot10^{-2}$
**6J8I**	$1.1\cdot10^{-2}$	$2.0\cdot10^{-2}$	$3.8\cdot10^{-2}$	$5.5\cdot10^{-3}$	$1.1\cdot10^{-2}$	$2.7\cdot10^{-2}$	$1.2\cdot10^{-2}$	$2.3\cdot10^{-2}$	$5.0\cdot10^{-2}$	$4.5\cdot10^{-3}$	$1.2\cdot10^{-2}$	$3.8\cdot10^{-2}$
**6JP5**	$4.5\cdot10^{-2}$	0.13	0.21	$4.8\cdot10^{-2}$	0.13	0.20	$6.3\cdot10^{-2}$	0.18	0.28	$7.0\cdot10^{-2}$	0.18	0.27
**6JP8**	0.18	0.45	0.62	0.18	0.45	0.62	0.20	0.49	0.66	0.20	0.48	0.66
**6JPA-1**	$7.3\cdot10^{-6}$	$3.8\cdot10^{-4}$	$5.1\cdot10^{-3}$	$7.6\cdot10^{-6}$	$3.3\cdot10^{-4}$	$4.5\cdot10^{-3}$	$1.5\cdot10^{-6}$	$1.9\cdot10^{-4}$	$3.2\cdot10^{-3}$	$1.4\cdot10^{-6}$	$1.7\cdot10^{-4}$	$2.8\cdot10^{-3}$
**6JPA-2**	$4.4\cdot10^{-9}$	$9.9\cdot10^{-6}$	$2.5\cdot10^{-4}$	$2.9\cdot10^{-8}$	$1.3\cdot10^{-5}$	$2.6\cdot10^{-4}$	$1.6\cdot10^{-9}$	$6.8\cdot10^{-6}$	$1.7\cdot10^{-4}$	$4.8\cdot10^{-9}$	$5.1\cdot10^{-6}$	$1.3\cdot10^{-4}$
**6JPB**	$1.6\cdot10^{-8}$	$1.9\cdot10^{-5}$	$6.4\cdot10^{-4}$	$8.8\cdot10^{-8}$	$2.6\cdot10^{-5}$	$6.9\cdot10^{-4}$	$2.7\cdot10^{-9}$	$5.8\cdot10^{-6}$	$3.0\cdot10^{-4}$	$2.9\cdot10^{-8}$	$1.1\cdot10^{-5}$	$4.1\cdot10^{-4}$
**6KZP**	$7.1\cdot10^{-2}$	0.19	0.31	$7.9\cdot10^{-2}$	0.20	0.31	0.11	0.27	0.41	0.12	0.26	0.38
**6U88**	$6.4\cdot10^{-5}$	$4.7\cdot10^{-3}$	$2.6\cdot10^{-2}$	$4.4\cdot10^{-5}$	$3.3\cdot10^{-3}$	$2.0\cdot10^{-2}$	$3.6\cdot10^{-5}$	$3.1\cdot10^{-3}$	$1.9\cdot10^{-2}$	$1.2\cdot10^{-5}$	$1.2\cdot10^{-3}$	$8.5\cdot10^{-3}$
**6UZ0**	$1.3\cdot10^{-3}$	$2.2\cdot10^{-2}$	$8.6\cdot10^{-2}$	$1.6\cdot10^{-3}$	$2.3\cdot10^{-2}$	$8.7\cdot10^{-2}$	$1.3\cdot10^{-3}$	$2.0\cdot10^{-2}$	$7.7\cdot10^{-2}$	$1.9\cdot10^{-3}$	$2.1\cdot10^{-2}$	$7.4\cdot10^{-2}$

Table S4.5. P_{Near} of RMSD vs `LigInterface` for GALigandDock, padding 2 Å. Full population of poses.

	k_BT=1.0						k_BT=0.62					
	20,000 poses			100,000 poses			20,000 poses			100,000 poses		
**PDB ID**	**λ=1.0**	**λ=1.5**	**λ=2.0**	**λ=1.0**	**λ=1.5**	**λ=2.0**	**λ=1.0**	**λ=1.5**	**λ=2.0**	**λ=1.0**	**λ=1.5**	**λ=2.0**
**5EK0**	0.46	0.63	0.71	0.42	0.60	0.68	0.53	0.71	0.78	0.46	0.63	0.72
**6J8G**	$9.1\cdot10^{-5}$	$8.4\cdot10^{-4}$	$1.1\cdot10^{-2}$	$8.2\cdot10^{-5}$	$1.0\cdot10^{-3}$	$1.2\cdot10^{-2}$	$1.8\cdot10^{-6}$	$6.5\cdot10^{-4}$	$1.4\cdot10^{-2}$	$3.3\cdot10^{-6}$	$8.9\cdot10^{-4}$	$1.5\cdot10^{-2}$
**6J8I**	$4.6\cdot10^{-5}$	$2.3\cdot10^{-4}$	$1.3\cdot10^{-3}$	$6.7\cdot10^{-5}$	$2.9\cdot10^{-4}$	$1.3\cdot10^{-3}$	$1.3\cdot10^{-7}$	$1.3\cdot10^{-6}$	$1.2\cdot10^{-4}$	$4.8\cdot10^{-7}$	$2.9\cdot10^{-6}$	$1.7\cdot10^{-4}$
**6JP5**	$3.1\cdot10^{-6}$	$4.7\cdot10^{-4}$	$9.9\cdot10^{-3}$	$3.1\cdot10^{-6}$	$4.7\cdot10^{-4}$	$9.9\cdot10^{-3}$	$5.7\cdot10^{-7}$	$4.9\cdot10^{-4}$	$1.1\cdot10^{-2}$	$3.7\cdot10^{-7}$	$4.9\cdot10^{-4}$	$1.1\cdot10^{-2}$
**6JP8**	$1.2\cdot10^{-5}$	$1.9\cdot10^{-4}$	$2.4\cdot10^{-3}$	$1.7\cdot10^{-5}$	$2.0\cdot10^{-4}$	$2.4\cdot10^{-3}$	$4.3\cdot10^{-7}$	$4.7\cdot10^{-5}$	$1.6\cdot10^{-3}$	$1.6\cdot10^{-6}$	$4.9\cdot10^{-5}$	$1.5\cdot10^{-3}$
**6JPA-1**	$5.0\cdot10^{-7}$	$1.5\cdot10^{-4}$	$3.9\cdot10^{-3}$	$5.3\cdot10^{-7}$	$1.4\cdot10^{-4}$	$3.1\cdot10^{-3}$	$3.0\cdot10^{-8}$	$8.1\cdot10^{-5}$	$2.9\cdot10^{-3}$	$1.9\cdot10^{-8}$	$2.3\cdot10^{-5}$	$1.2\cdot10^{-3}$
**6JPA-2**	$6.3\cdot10^{-10}$	$5.2\cdot10^{-7}$	$1.6\cdot10^{-5}$	$6.6\cdot10^{-10}$	$9.7\cdot10^{-7}$	$2.8\cdot10^{-5}$	$2.3\cdot10^{-12}$	$7.2\cdot10^{-9}$	$7.5\cdot10^{-7}$	$5.6\cdot10^{-12}$	$2.4\cdot10^{-8}$	$1.5\cdot10^{-6}$
**6JPB**	$8.7\cdot10^{-7}$	$6.4\cdot10^{-5}$	$2.6\cdot10^{-3}$	$9.5\cdot10^{-7}$	$6.2\cdot10^{-5}$	$2.7\cdot10^{-3}$	$9.1\cdot10^{-9}$	$4.1\cdot10^{-5}$	$3.2\cdot10^{-3}$	$8.9\cdot10^{-9}$	$4.3\cdot10^{-5}$	$3.3\cdot10^{-3}$
**6KZP**	$2.7\cdot10^{-2}$	$8.3\cdot10^{-2}$	0.15	$3.1\cdot10^{-2}$	$9.3\cdot10^{-2}$	0.16	$2.4\cdot10^{-2}$	$8.1\cdot10^{-2}$	0.15	$3.1\cdot10^{-2}$	$8.7\cdot10^{-2}$	0.15
**6U88**	$7.6\cdot10^{-4}$	$1.1\cdot10^{-2}$	$4.4\cdot10^{-2}$	$1.1\cdot10^{-3}$	$1.2\cdot10^{-2}$	$4.5\cdot10^{-2}$	$1.2\cdot10^{-4}$	$4.8\cdot10^{-3}$	$2.5\cdot10^{-2}$	$9.5\cdot10^{-4}$	$8.9\cdot10^{-3}$	$3.2\cdot10^{-2}$
**6UZ0**	$2.0\cdot10^{-5}$	$3.3\cdot10^{-4}$	$2.1\cdot10^{-3}$	$2.5\cdot10^{-5}$	$3.3\cdot10^{-4}$	$1.9\cdot10^{-3}$	$1.8\cdot10^{-6}$	$7.1\cdot10^{-5}$	$1.6\cdot10^{-3}$	$2.1\cdot10^{-6}$	$5.1\cdot10^{-5}$	$1.3\cdot10^{-3}$

Table S4.6. P_{Near} of RMSD vs `LigInterface` for GALigandDock, padding 2 Å. Lowest 10% of poses by `total_score`.

	k_BT=1.0						k_BT=0.62					
	20,000 poses			100,000 poses			20,000 poses			100,000 poses		
**PDB ID**	**λ=1.0**	**λ=1.5**	**λ=2.0**	**λ=1.0**	**λ=1.5**	**λ=2.0**	**λ=1.0**	**λ=1.5**	**λ=2.0**	**λ=1.0**	**λ=1.5**	**λ=2.0**
**5EK0**	0.53	0.73	0.83	0.51	0.71	0.81	0.58	0.78	0.87	0.55	0.75	0.85
**6J8G**	$7.2\bullet10^{-8}$	$4.0\bullet10^{-4}$	$9.9\bullet10^{-3}$	$9.2\bullet10^{-8}$	$4.1\bullet10^{-4}$	$9.9\bullet10^{-3}$	$1.2\bullet10^{-7}$	$6.1\bullet10^{-4}$	$1.3\bullet10^{-2}$	$1.8\bullet10^{-7}$	$6.9\bullet10^{-4}$	$1.4\bullet10^{-2}$
**6J8I**	$6.0\bullet10^{-7}$	$5.7\bullet10^{-5}$	$8.5\bullet10^{-4}$	$8.5\bullet10^{-7}$	$5.4\bullet10^{-5}$	$8.0\bullet10^{-4}$	$3.9\bullet10^{-9}$	$8.1\bullet10^{-7}$	$1.1\bullet10^{-4}$	$7.8\bullet10^{-9}$	$1.3\bullet10^{-6}$	$1.7\bullet10^{-4}$
**6JP5**	$1.9\bullet10^{-6}$	$1.4\bullet10^{-4}$	$4.2\bullet10^{-3}$	$8.7\bullet10^{-7}$	$9.4\bullet10^{-5}$	$3.9\bullet10^{-3}$	$7.0\bullet10^{-7}$	$1.1\bullet10^{-4}$	$4.7\bullet10^{-3}$	$3.0\bullet10^{-7}$	$8.8\bullet10^{-5}$	$4.4\bullet10^{-3}$
**6JP8**	$1.3\bullet10^{-9}$	$3.4\bullet10^{-5}$	$1.7\bullet10^{-3}$	$1.2\bullet10^{-9}$	$3.1\bullet10^{-5}$	$1.6\bullet10^{-3}$	$1.3\bullet10^{-9}$	$3.1\bullet10^{-5}$	$1.5\bullet10^{-3}$	$1.2\bullet10^{-9}$	$2.9\bullet10^{-5}$	$1.4\bullet10^{-3}$
**6JPA-1**	$2.8\bullet10^{-7}$	$1.4\bullet10^{-4}$	$3.9\bullet10^{-3}$	$4.2\bullet10^{-7}$	$1.2\bullet10^{-4}$	$2.9\bullet10^{-3}$	$2.5\bullet10^{-8}$	$8.1\bullet10^{-5}$	$2.9\bullet10^{-3}$	$1.7\bullet10^{-8}$	$2.1\bullet10^{-5}$	$1.2\bullet10^{-3}$
**6JPA-2**	$1.6\bullet10^{-11}$	$1.5\bullet10^{-7}$	$9.1\bullet10^{-6}$	$1.7\bullet10^{-10}$	$5.9\bullet10^{-7}$	$1.9\bullet10^{-5}$	$2.5\bullet10^{-13}$	$4.2\bullet10^{-9}$	$7.0\bullet10^{-7}$	$2.8\bullet10^{-12}$	$1.9\bullet10^{-8}$	$1.4\bullet10^{-6}$
**6JPB**	$1.6\bullet10^{-8}$	$3.9\bullet10^{-5}$	$2.8\bullet10^{-3}$	$1.1\bullet10^{-8}$	$4.1\bullet10^{-5}$	$2.9\bullet10^{-3}$	$4.0\bullet10^{-10}$	$4.2\bullet10^{-5}$	$3.3\bullet10^{-3}$	$3.7\bullet10^{-10}$	$4.5\bullet10^{-5}$	$3.5\bullet10^{-3}$
**6KZP**	$4.8\bullet10^{-2}$	0.14	0.25	$5.7\bullet10^{-2}$	0.17	0.28	$4.5\bullet10^{-2}$	0.15	0.27	$7.9\bullet10^{-2}$	0.22	0.36
**6U88**	$4.1\bullet10^{-4}$	$9.3\bullet10^{-3}$	$4.3\bullet10^{-2}$	$4.8\bullet10^{-4}$	$9.2\bullet10^{-3}$	$4.2\bullet10^{-2}$	$7.8\bullet10^{-5}$	$4.6\bullet10^{-3}$	$2.4\bullet10^{-2}$	$9.8\bullet10^{-5}$	$4.7\bullet10^{-3}$	$2.5\bullet10^{-2}$
**6UZ0**	$9.6\bullet10^{-7}$	$1.6\bullet10^{-4}$	$2.1\bullet10^{-3}$	$2.9\bullet10^{-6}$	$1.1\bullet10^{-4}$	$1.7\bullet10^{-3}$	$2.5\bullet10^{-7}$	$6.6\bullet10^{-5}$	$2.0\bullet10^{-3}$	$2.9\bullet10^{-7}$	$3.5\bullet10^{-5}$	$1.6\bullet10^{-3}$

Table S4.7. P_{Near} of RMSD vs `LigInterface` for GALigandDock, padding 4 Å. Full population of poses.

	k_BT=1.0						k_BT=0.62					
	20,000 poses			100,000 poses			20,000 poses			100,000 poses		
**PDB ID**	**λ=1.0**	**λ=1.5**	**λ=2.0**	**λ=1.0**	**λ=1.5**	**λ=2.0**	**λ=1.0**	**λ=1.5**	**λ=2.0**	**λ=1.0**	**λ=1.5**	**λ=2.0**
**5EK0**	0.53	0.72	0.81	0.45	0.64	0.73	0.59	0.78	0.87	0.56	0.75	0.83
**6J8G**	$5.5\bullet10^{-5}$	$9.4\bullet10^{-4}$	$1.3\bullet10^{-2}$	$1.9\bullet10^{-4}$	$1.5\bullet10^{-3}$	$1.7\bullet10^{-2}$	$2.3\bullet10^{-6}$	$1.0\bullet10^{-3}$	$1.8\bullet10^{-2}$	$5.7\bullet10^{-6}$	$1.5\bullet10^{-3}$	$2.5\bullet10^{-2}$
**6J8I**	$5.2\bullet10^{-3}$	$6.3\bullet10^{-2}$	0.16	$4.1\bullet10^{-3}$	$5.8\bullet10^{-2}$	0.15	$3.2\bullet10^{-3}$	$4.5\bullet10^{-2}$	0.12	$3.3\bullet10^{-3}$	$5.1\bullet10^{-2}$	0.14
**6JP5**	$1.7\bullet10^{-2}$	$5.2\bullet10^{-2}$	$9.1\bullet10^{-2}$	$1.7\bullet10^{-2}$	$5.4\bullet10^{-2}$	$9.5\bullet10^{-2}$	$4.7\bullet10^{-2}$	0.13	0.20	$5.1\bullet10^{-2}$	0.15	0.22
**6JP8**	0.15	0.30	0.39	0.14	0.29	0.38	0.24	0.48	0.62	0.23	0.47	0.61
**6JPA-1**	$2.1\bullet10^{-7}$	$1.4\bullet10^{-4}$	$3.4\bullet10^{-3}$	$7.9\bullet10^{-7}$	$1.4\bullet10^{-4}$	$3.0\bullet10^{-3}$	$1.1\bullet10^{-8}$	$5.6\bullet10^{-5}$	$2.8\bullet10^{-3}$	$3.1\bullet10^{-8}$	$3.3\bullet10^{-5}$	$1.3\bullet10^{-3}$
**6JPA-2**	$1.2\bullet10^{-9}$	$1.0\bullet10^{-6}$	$2.6\bullet10^{-5}$	$7.2\bullet10^{-10}$	$7.0\bullet10^{-7}$	$1.7\bullet10^{-5}$	$8.0\bullet10^{-12}$	$3.7\bullet10^{-8}$	$1.7\bullet10^{-6}$	$5.0\bullet10^{-12}$	$7.3\bullet10^{-9}$	$4.1\bullet10^{-7}$
**6JPB**	$1.6\bullet10^{-7}$	$1.2\bullet10^{-5}$	$7.7\bullet10^{-4}$	$1.3\bullet10^{-7}$	$1.3\bullet10^{-5}$	$8.0\bullet10^{-4}$	$1.6\bullet10^{-10}$	$1.6\bullet10^{-6}$	$3.8\bullet10^{-4}$	$1.1\bullet10^{-10}$	$1.6\bullet10^{-6}$	$3.8\bullet10^{-4}$
**6KZP**	$1.4\bullet10^{-2}$	$4.2\bullet10^{-2}$	$7.4\bullet10^{-2}$	$4.8\bullet10^{-3}$	$1.4\bullet10^{-2}$	$2.3\bullet10^{-2}$	$8.0\bullet10^{-3}$	$2.2\bullet10^{-2}$	$4.0\bullet10^{-2}$	$2.6\bullet10^{-4}$	$6.2\bullet10^{-4}$	$1.2\bullet10^{-3}$
**6U88**	$4.8\bullet10^{-4}$	$9.2\bullet10^{-3}$	$4.2\bullet10^{-2}$	$3.7\bullet10^{-4}$	$6.6\bullet10^{-3}$	$3.0\bullet10^{-2}$	$7.8\bullet10^{-5}$	$4.7\bullet10^{-3}$	$2.5\bullet10^{-2}$	$2.9\bullet10^{-5}$	$1.5\bullet10^{-3}$	$8.0\bullet10^{-3}$
**6UZ0**	$1.8\bullet10^{-5}$	$3.6\bullet10^{-4}$	$1.6\bullet10^{-3}$	$1.7\bullet10^{-5}$	$3.3\bullet10^{-4}$	$1.5\bullet10^{-3}$	$1.5\bullet10^{-6}$	$3.7\bullet10^{-5}$	$6.4\bullet10^{-4}$	$9.1\bullet10^{-7}$	$2.3\bullet10^{-5}$	$4.4\bullet10^{-4}$

Table S4.8. P_{Near} of RMSD vs `LigInterface` for GALigandDock, padding 4 Å. Lowest 10% of poses by `total_score`.

	k_BT=1.0						k_BT=0.62					
	20,000 poses			100,000 poses			20,000 poses			100,000 poses		
**PDB ID**	**λ=1.0**	**λ=1.5**	**λ=2.0**	**λ=1.0**	**λ=1.5**	**λ=2.0**	**λ=1.0**	**λ=1.5**	**λ=2.0**	**λ=1.0**	**λ=1.5**	**λ=2.0**
**5EK0**	0.55	0.75	0.84	0.48	0.67	0.77	0.59	0.79	0.88	0.57	0.76	0.85
**6J8G**	$2.2\bullet10^{-7}$	$6.4\bullet10^{-4}$	$1.2\bullet10^{-2}$	$3.5\bullet10^{-7}$	$9.1\bullet10^{-4}$	$1.7\bullet10^{-2}$	$3.1\bullet10^{-7}$	$1.0\bullet10^{-3}$	$1.8\bullet10^{-2}$	$4.7\bullet10^{-7}$	$1.5\bullet10^{-3}$	$2.5\bullet10^{-2}$
**6J8I**	$4.3\bullet10^{-3}$	$5.9\bullet10^{-2}$	0.15	$3.9\bullet10^{-3}$	$5.7\bullet10^{-2}$	0.15	$3.1\bullet10^{-3}$	$4.4\bullet10^{-2}$	0.12	$3.3\bullet10^{-3}$	$5.0\bullet10^{-2}$	0.14
**6JP5**	$2.3\bullet10^{-2}$	$6.8\bullet10^{-2}$	0.11	$2.3\bullet10^{-2}$	$6.6\bullet10^{-2}$	0.11	$5.4\bullet10^{-2}$	0.15	0.22	$5.8\bullet10^{-2}$	0.16	0.23
**6JP8**	0.21	0.41	0.53	0.20	0.40	0.52	0.26	0.52	0.67	0.26	0.52	0.66
**6JPA-1**	$2.1\bullet10^{-7}$	$1.5\bullet10^{-4}$	$3.5\bullet10^{-3}$	$6.1\bullet10^{-7}$	$1.4\bullet10^{-4}$	$3.1\bullet10^{-3}$	$1.6\bullet10^{-8}$	$5.5\bullet10^{-5}$	$1.8\bullet10^{-3}$	$2.6\bullet10^{-8}$	$3.4\bullet10^{-5}$	$1.4\bullet10^{-3}$
**6JPA-2**	$1.4\bullet10^{-10}$	$7.3\bullet10^{-7}$	$2.0\bullet10^{-5}$	$1.7\bullet10^{-10}$	$4.3\bullet10^{-7}$	$1.3\bullet10^{-5}$	$5.2\bullet10^{-12}$	$3.3\bullet10^{-8}$	$1.5\bullet10^{-6}$	$1.0\bullet10^{-12}$	$5.2\bullet10^{-9}$	$3.8\bullet10^{-7}$
**6JPB**	$8.9\bullet10^{-11}$	$9.6\bullet10^{-6}$	$9.2\bullet10^{-4}$	$1.1\bullet10^{-10}$	$9.8\bullet10^{-6}$	$9.4\bullet10^{-4}$	$4.8\bullet10^{-12}$	$1.6\bullet10^{-6}$	$3.9\bullet10^{-4}$	$4.1\bullet10^{-12}$	$1.6\bullet10^{-6}$	$3.9\bullet10^{-4}$
**6KZP**	$2.9\bullet10^{-2}$	$8.6\bullet10^{-2}$	0.15	$6.4\bullet10^{-3}$	$1.8\bullet10^{-2}$	$3.0\bullet10^{-2}$	$2.3\bullet10^{-2}$	$6.3\bullet10^{-2}$	0.10	$2.9\bullet10^{-4}$	$6.6\bullet10^{-4}$	$1.0\bullet10^{-3}$
**6U88**	$3.7\bullet10^{-4}$	$1.2\bullet10^{-2}$	$5.7\bullet10^{-2}$	$2.4\bullet10^{-4}$	$8.4\bullet10^{-3}$	$4.2\bullet10^{-2}$	$7.8\bullet10^{-5}$	$6.1\bullet10^{-3}$	$3.2\bullet10^{-2}$	$2.9\bullet10^{-5}$	$2.4\bullet10^{-3}$	$1.3\bullet10^{-2}$
**6UZ0**	$1.8\bullet10^{-6}$	$9.8\bullet10^{-5}$	$1.4\bullet10^{-3}$	$2.8\bullet10^{-7}$	$5.7\bullet10^{-5}$	$1.1\bullet10^{-3}$	$1.9\bullet10^{-7}$	$2.3\bullet10^{-5}$	$1.2\bullet10^{-3}$	$3.5\bullet10^{-8}$	$1.5\bullet10^{-5}$	$9.4\bullet10^{-4}$

Table S4.9. P_{Near} of RMSD vs `LigInterface` for GALigandDock, padding 7 Å. Full population of poses.

	k_BT=1.0						k_BT=0.62					
	20,000 poses			100,000 poses			20,000 poses			100,000 poses		
**PDB ID**	**λ=1.0**	**λ=1.5**	**λ=2.0**	**λ=1.0**	**λ=1.5**	**λ=2.0**	**λ=1.0**	**λ=1.5**	**λ=2.0**	**λ=1.0**	**λ=1.5**	**λ=2.0**
**5EK0**	0.45	0.68	0.80	0.46	0.69	0.81	0.47	0.70	0.81	0.50	0.72	0.83
**6J8G**	$4.0 \cdot 10^{-5}$	$7.1 \cdot 10^{-4}$	$1.2 \cdot 10^{-2}$	$7.1 \cdot 10^{-5}$	$1.2 \cdot 10^{-3}$	$1.3 \cdot 10^{-2}$	$8.2 \cdot 10^{-7}$	$7.3 \cdot 10^{-4}$	$1.5 \cdot 10^{-2}$	$2.1 \cdot 10^{-5}$	$1.1 \cdot 10^{-3}$	$1.6 \cdot 10^{-2}$
**6J8I**	$3.9 \cdot 10^{-3}$	$6.5 \cdot 10^{-2}$	0.19	$5.1 \cdot 10^{-3}$	$8.1 \cdot 10^{-2}$	0.22	$3.5 \cdot 10^{-3}$	$7.5 \cdot 10^{-2}$	0.22	$6.3 \cdot 10^{-3}$	$1.0 \cdot 10^{-1}$	0.27
**6JP5**	$6.1 \cdot 10^{-3}$	$2.3 \cdot 10^{-2}$	$4.7 \cdot 10^{-2}$	$1.9 \cdot 10^{-2}$	$5.9 \cdot 10^{-2}$	0.10	$1.3 \cdot 10^{-2}$	$4.2 \cdot 10^{-2}$	$7.2 \cdot 10^{-2}$	$5.8 \cdot 10^{-2}$	0.16	0.24
**6JP8**	0.17	0.33	0.44	0.18	0.36	0.47	0.23	0.46	0.60	0.23	0.47	0.60
**6JPA-1**	$1.1 \cdot 10^{-5}$	$1.0 \cdot 10^{-3}$	$9.0 \cdot 10^{-3}$	$1.3 \cdot 10^{-6}$	$4.2 \cdot 10^{-4}$	$5.5 \cdot 10^{-3}$	$9.4 \cdot 10^{-6}$	$1.0 \cdot 10^{-3}$	$9.3 \cdot 10^{-3}$	$2.4 \cdot 10^{-7}$	$1.6 \cdot 10^{-4}$	$2.4 \cdot 10^{-3}$
**6JPA-2**	$4.5 \cdot 10^{-10}$	$4.1 \cdot 10^{-7}$	$1.4 \cdot 10^{-5}$	$2.9 \cdot 10^{-10}$	$3.1 \cdot 10^{-7}$	$1.7 \cdot 10^{-5}$	$2.0 \cdot 10^{-12}$	$7.9 \cdot 10^{-9}$	$4.0 \cdot 10^{-6}$	$3.8 \cdot 10^{-13}$	$8.5 \cdot 10^{-9}$	$1.8 \cdot 10^{-5}$
**6JPB**	$1.8 \cdot 10^{-8}$	$8.7 \cdot 10^{-7}$	$4.1 \cdot 10^{-5}$	$2.1 \cdot 10^{-8}$	$8.2 \cdot 10^{-7}$	$4.2 \cdot 10^{-5}$	$5.4 \cdot 10^{-12}$	$2.4 \cdot 10^{-8}$	$2.2 \cdot 10^{-6}$	$4.6 \cdot 10^{-12}$	$2.0 \cdot 10^{-8}$	$2.8 \cdot 10^{-6}$
**6KZP**	$1.4 \cdot 10^{-3}$	$3.8 \cdot 10^{-3}$	$6.6 \cdot 10^{-3}$	$1.2 \cdot 10^{-3}$	$3.0 \cdot 10^{-3}$	$4.8 \cdot 10^{-3}$	$2.9 \cdot 10^{-5}$	$1.2 \cdot 10^{-4}$	$2.9 \cdot 10^{-4}$	$2.5 \cdot 10^{-5}$	$6.7 \cdot 10^{-5}$	$1.2 \cdot 10^{-4}$
**6U88**	$3.1 \cdot 10^{-3}$	$1.1 \cdot 10^{-2}$	$2.9 \cdot 10^{-2}$	$3.9 \cdot 10^{-3}$	$1.3 \cdot 10^{-2}$	$3.2 \cdot 10^{-2}$	$2.2 \cdot 10^{-4}$	$1.1 \cdot 10^{-3}$	$4.1 \cdot 10^{-3}$	$3.7 \cdot 10^{-4}$	$1.5 \cdot 10^{-3}$	$5.0 \cdot 10^{-3}$
**6UZ0**	$3.9 \cdot 10^{-6}$	$3.8 \cdot 10^{-5}$	$1.6 \cdot 10^{-4}$	$4.6 \cdot 10^{-6}$	$3.8 \cdot 10^{-5}$	$1.6 \cdot 10^{-4}$	$1.7 \cdot 10^{-8}$	$3.1 \cdot 10^{-7}$	$6.7 \cdot 10^{-6}$	$2.2 \cdot 10^{-8}$	$2.9 \cdot 10^{-7}$	$5.9 \cdot 10^{-6}$

Table S4.10. P_{Near} of RMSD vs `LigInterface` for GALigandDock, padding 7 Å. Lowest 10% of poses by `total_score`.

	k_BT=1.0						k_BT=0.62					
	20,000 poses			100,000 poses			20,000 poses			100,000 poses		
**PDB ID**	**λ=1.0**	**λ=1.5**	**λ=2.0**	**λ=1.0**	**λ=1.5**	**λ=2.0**	**λ=1.0**	**λ=1.5**	**λ=2.0**	**λ=1.0**	**λ=1.5**	**λ=2.0**
**5EK0**	0.45	0.68	0.80	0.46	0.69	0.81	0.47	0.70	0.81	0.50	0.72	0.83
**6J8G**	$4.9\cdot10^{-5}$	$9.1\cdot10^{-4}$	$1.6\cdot10^{-2}$	$3.5\cdot10^{-5}$	$1.2\cdot10^{-3}$	$1.4\cdot10^{-2}$	$1.0\cdot10^{-6}$	$9.1\cdot10^{-4}$	$1.8\cdot10^{-2}$	$2.1\cdot10^{-5}$	$1.2\cdot10^{-3}$	$1.6\cdot10^{-2}$
**6J8I**	$3.0\cdot10^{-3}$	$3.5\cdot10^{-2}$	$1.0\cdot10^{-1}$	$5.7\cdot10^{-3}$	$8.3\cdot10^{-2}$	0.22	$1.8\cdot10^{-3}$	$2.6\cdot10^{-2}$	$7.9\cdot10^{-2}$	$7.1\cdot10^{-3}$	0.10	0.27
**6JP5**	$5.9\cdot10^{-3}$	$2.5\cdot10^{-2}$	$5.3\cdot10^{-2}$	$3.0\cdot10^{-2}$	$8.8\cdot10^{-2}$	0.14	$1.0\cdot10^{-2}$	$3.5\cdot10^{-2}$	$6.3\cdot10^{-2}$	$7.1\cdot10^{-2}$	0.19	0.29
**6JP8**	0.19	0.38	0.50	0.20	0.40	0.52	0.23	0.47	0.61	0.24	0.47	0.61
**6JPA-1**	$1.4\cdot10^{-5}$	$1.3\cdot10^{-3}$	$1.2\cdot10^{-2}$	$1.5\cdot10^{-6}$	$4.8\cdot10^{-4}$	$6.2\cdot10^{-3}$	$1.8\cdot10^{-5}$	$1.9\cdot10^{-3}$	$1.8\cdot10^{-2}$	$2.9\cdot10^{-7}$	$1.9\cdot10^{-4}$	$2.8\cdot10^{-3}$
**6JPA-2**	$1.7\cdot10^{-10}$	$2.1\cdot10^{-7}$	$9.5\cdot10^{-6}$	$6.0\cdot10^{-11}$	$2.1\cdot10^{-7}$	$1.5\cdot10^{-5}$	$1.3\cdot10^{-12}$	$5.2\cdot10^{-9}$	$4.0\cdot10^{-6}$	$1.8\cdot10^{-13}$	$8.3\cdot10^{-9}$	$1.9\cdot10^{-5}$
**6JPB**	$1.9\cdot10^{-10}$	$9.5\cdot10^{-7}$	$6.4\cdot10^{-5}$	$4.9\cdot10^{-11}$	$9.9\cdot10^{-7}$	$8.0\cdot10^{-5}$	$1.5\cdot10^{-12}$	$3.7\cdot10^{-8}$	$3.3\cdot10^{-6}$	$4.6\cdot10^{-13}$	$5.9\cdot10^{-8}$	$7.8\cdot10^{-6}$
**6KZP**	$2.0\cdot10^{-3}$	$5.4\cdot10^{-3}$	$9.1\cdot10^{-3}$	$1.4\cdot10^{-3}$	$3.5\cdot10^{-3}$	$5.6\cdot10^{-3}$	$3.3\cdot10^{-5}$	$1.4\cdot10^{-4}$	$3.2\cdot10^{-4}$	$2.4\cdot10^{-5}$	$6.6\cdot10^{-5}$	$1.1\cdot10^{-4}$
**6U88**	$2.4\cdot10^{-4}$	$4.7\cdot10^{-3}$	$2.3\cdot10^{-2}$	$1.7\cdot10^{-4}$	$4.4\cdot10^{-3}$	$2.3\cdot10^{-2}$	$9.9\cdot10^{-6}$	$6.2\cdot10^{-4}$	$3.4\cdot10^{-3}$	$8.4\cdot10^{-6}$	$6.8\cdot10^{-4}$	$3.8\cdot10^{-3}$
**6UZ0**	$5.8\cdot10^{-6}$	$7.5\cdot10^{-5}$	$4.0\cdot10^{-4}$	$7.2\cdot10^{-6}$	$8.2\cdot10^{-5}$	$4.2\cdot10^{-4}$	$3.9\cdot10^{-8}$	$9.2\cdot10^{-7}$	$2.1\cdot10^{-5}$	$7.4\cdot10^{-8}$	$1.3\cdot10^{-6}$	$2.9\cdot10^{-5}$

Table S5. P_{Near} central pore cavity cases subclassified by position. Values reflect the primary text for RosettaLigand (all ligand atom mode with 100,000 output poses) and GALigandDock (padding=7 Å with 100,000 output poses), $k_BT=0.62$, $\lambda=2.0$.

			P_{Near}		RMSD (Å)	
**Sub-classification**	**PDB ID**	**Rotatable bonds**	**RosettaLigand**	**GALigandDock**	**RosettaLigand**	**GALigandDock**
Fenestration	6JP5	5	0.28	0.29	0.77	1.3
	6JP8	3	0.66	0.61	0.93	0.97
	6U88	6	$4.3 \cdot 10^{-2}$	$3.8 \cdot 10^{-3}$	1.0	0.90
Pore and fenestration	6JPA, position 1	13	$4.3 \cdot 10^{-3}$	$2.8 \cdot 10^{-3}$	1.4	2.0
	6KZP	6	0.62	$1.1 \cdot 10^{-4}$	0.73	0.84
	6UZ0	7	0.12	$2.9 \cdot 10^{-5}$	1.2	0.94
Pore	6JPA, position 2	13	$1.2 \cdot 10^{-4}$	$1.9 \cdot 10^{-5}$	2.2	2.1
	6JPB	7	$6.4 \cdot 10^{-4}$	$7.8 \cdot 10^{-6}$	2.5	1.1

Appendix S1. Example Antechamber input for AM1BCC ligand minimization

```
antechamber -i $ligand_input -fi mol2 -o $ligand_output -fo mol2 -c bcc -nc $net_charge -at sybyl

```

where `$ligand_input` is the .mol2 ligand input, `$ligand_output` is the .mol2 ligand output name, and `$net_charge` is the net charge of the ligand

Appendix S2. Example Bash script generating ligand conformers with OpenEye Omega and ligand parameters generation for RosettaLigand

```
#!/bin/bash
# New to OpenEye? Install the openeye toolkit with Conda. (Read the OpenEye toolkit README)
# Using Python2.7? Look for pip install packages at: https://anaconda.org/OpenEye
rosetta_src={YOUR_FOLDER_PATH}/rosetta_bin_linux_2021.07.61567_bundle/main/source
openeye_bin={YOUR_FOLDER_PATH}/openeye/bin

if [ $# -lt 1 ]; then
    echo "USAGE: generate-ligand-conformers.sh <ligand name only (input in .mol2 format)>"
    exit
fi
drug=$1
dir=$(echo ${PWD})
set -v

# Set up directory structure
mkdir -p ligand
# Make ligands
pushd ligand
```

```
mkdir -p {fa,cen}/{conf1,confs,kins,withxtal}
omega="${openeye_bin}/omega2 -includeInput -commentEnergy"
$omega -in $dir/$drug.mol2 -out $drug.omega.mol2 -prefix _$drug
python ${rosetta_src}/src/apps/public/ligand_docking/assign_charges.py
< $drug.omega.mol2 > $drug.am1bcc.mol2
python ${rosetta_src}/scripts/python/public/molfile_to_params.py -c -nX00
-p$drug -k$drug.kin $drug.am1bcc.mol2
cat ${drug}_????.fa.pdb | gzip -c > fa/withxtal/${drug}_confs.fa.pdb.gz &&
( [ -f ${drug}_0002.fa.pdb ] || cp ${drug}_0001.fa.pdb ${drug}_0002.fa.pdb )
mv ${drug}_0001.fa.pdb fa/conf1/
cat ${drug}_????.fa.pdb | gzip -c > fa/${drug}_confs.fa.pdb.gz
mv ${drug}_????.fa.pdb fa/confs/
echo "PDB_ROTAMERS ${drug}_confs.fa.pdb" >> $drug.fa.params
cp $drug.fa.params fa/withxtal/
mv $drug.fa.params fa/
mv $drug.fa.kin fa/kins/
cat ${drug}_????.cen.pdb | gzip -c > cen/withxtal/${drug}_confs.cen.pdb.gz &&
 ( [ -f ${drug}_0002.cen.pdb ] || cp ${drug}_0001.cen.pdb ${drug}_0002.cen.pdb )
mv ${drug}_0001.cen.pdb cen/conf1/
cat ${drug}_????.cen.pdb | gzip -c > cen/${drug}_confs.cen.pdb.gz
mv ${drug}_????.cen.pdb cen/confs/
echo 'PDB_ROTAMERS $drug_confs.cen.pdb' >> $drug.cen.params
```

```
cp $drug.cen.params cen/withxtal/
mv $drug.cen.params cen/
mv $drug.cen.kin cen/kins/
popd

```

where `rosetta_src` is the file path to Rosetta software, `openeye_bin` is the file path to the OpenEye directory, and `drug` is the ligand name only (input in .mol2 format).

Appendix S3. Example input for small molecule crystal structure prediction

```
#!/bin/bash
## Formatted USAGE conditions and variables, reformatted to reflect example script from
Park H. et al 2021. https://dx.doi.org/10.1021/acs.jctc.0c01184

if [ $# -lt 2 ]; then
      echo "USAGE: ./bjh-GADock-dockrigid.sh <Ligand.pdb> <File handle for ligand
params>”
      exit
fi

## For De novo prediction, sgin=random_achiral or random_chiral & random=1. For native
prediction, sgin=input & random=0

rosetta_execute={ROSETTA_PATH}/main/source/bin/rosetta_scripts.default.linuxgccrelease
rosetta_database={ROSETTA_PATH}/main/database
sgin=random_achiral
random=1
```

```
nstruct=1

## DO NOT EDIT BELOW THIS LINE ##
PDB=${1}
file_handle=${2}
genpot=${PWD}/genpot_condensed.wts
$rosetta_execute \
    -in:path:database $rosetta_database \
    -parser:protocol ${PWD}/crystdock.xml \
    -parser:script_vars sg=$sgin random=$random \
    -s ${PWD}/${PDB} \
    -nstruct $nstruct \
    -gen_potential \
    -in:file:fullatom \
    -crystal_refine \
    -overwrite \
    -fa_max_dis 10.0 \
    -elec_max_dis 10.0 \
    -extra_res_fa ./seed_files/${file_handle}.params \
    -out:file:silent ${i}_GADock-sc_${file_handle}_.silent \
    -out:file:silent_struct_type binary \
    -out:file:scorefile GADock-sc_${file_handle}.sc \

```

Appendix S4. Example Python script for determining maximum ligand atom-atom distance for a set of conformers.

```
import warnings
import math
import pandas
import os
```

```
warnings.simplefilter(action='ignore', category=FutureWarning)

# EDIT ME
base_path = 'example/path/'
seed_files_path = base_path + 'folder_containing_params/'
# END EDITS

# identify the params file
params_file = [f for f in os.listdir(seed_files_path) if f.endswith('.params')][0]
# in the params file, identify the file located on the same line as PDB_ROTAMERS
with open(seed_files_path + params_file, 'r') as f:
    for line in f:
        if 'PDB_ROTAMERS' in line:
            pdb_rotamers_file = line.split('PDB_ROTAMERS')[1].strip()
            print(pdb_rotamers_file)

# create coordinates_df dataframe
coordinates_df = pandas.DataFrame(columns=['x', 'y', 'z'])

# initialize the max_distance variable
max_distance = 0

# read the pdb_rotamers file line by line
with open(seed_files_path + pdb_rotamers_file, 'r') as f:
    for line in f:
        # if the line starts with 'HETATM', store the x, y, and z coordinates
        if line.startswith('HETATM'):
            x = float(line[30:38].strip())
            y = float(line[38:46].strip())
            z = float(line[46:54].strip())
            coordinates_df = coordinates_df.append({'x': x, 'y': y, 'z': z},
```

```
ignore_index=True)
    if line.startswith('TER'):
      # for each coordinate, compute the distance to all other coordinates in dataframe, store the largest distance
      for index, row in coordinates_df.iterrows():
        for index2, row2 in coordinates_df.iterrows():
          if index != index2:
            distance = math.sqrt((row['x'] - row2['x']) ** 2 + (row['y'] - row2['y']) ** 2 + (row['z'] - row2['z']) ** 2)
            if distance > max_distance:
              max_distance = distance
      # reset the coordinates_df for the next conformer
      coordinates_df = pandas.DataFrame(columns=['x', 'y', 'z'])

print('The max distance is ' + str(max_distance))
exit()

```

Appendix S5. Example Bash script for RosettaLigand input.

```
#!/bin/bash

if [ $# -lt 3 ]; then
    echo "USAGE: ./RosettaLigand.sh <NativePDB.pdb> <Test Protein Ligand Complex > <File handle for params>"
    exit
fi

rosetta_execute={PATH_TO_ROSETTA}/main/source/bin/rosetta_scripts.static.linuxgccrelease
rosetta_database={PATH_TO_ROSETTA}}/main/database

```

```
## DO NOT EDIT BELOW THIS LINE ##
nativepdb=${1}
Testpdb=${2}
file_handle=${3}

$rosetta_execute \
      -in:path:database $rosetta_database \
      -s ${Testpdb} \
      -parser:protocol dock.xml \
      -parser:script_vars nativepdb=${nativepdb} \
      -extra_res_fa ${file_handle}.params \
      -ex1 \
      -ex2 \
      -ex1aro \
      -extrachi_cutoff 1 \
      -no_optH false \
      -flip_HNQ true \
      -ignore_ligand_chi true \
      -nstruct 10 \
      -overwrite \
      -out:file:silent_struct_type binary \
      -out:file:scorefile _Rosetta_ligand_${file_handle}.sc \
      -out:file:silent _Rosetta_ligand_${file_handle}.silent \
      -mistakes:restore_pre_talaris_2013_behavior \
      -score:analytic_etable_evaluation true

```

Appendix S6. Example XML for RosettaLigand

```
<ROSETTASCRIPTS>
  <SCOREFXNS>
    <ScoreFunction name="ligand_soft_rep" weights="ligand_soft_rep">
      <Reweight scoretype="fa_elec" weight="0.42"/>
      <Reweight scoretype="hbond_bb_sc" weight="1.3"/>
      <Reweight scoretype="hbond_sc" weight="1.3"/>
      <Reweight scoretype="rama" weight="0.2"/>
    </ScoreFunction>
    <ScoreFunction name="hard_rep" weights="ligand">
      <Reweight scoretype="fa_intra_rep" weight="0.004"/>
      <Reweight scoretype="fa_elec" weight="0.42"/>
      <Reweight scoretype="hbond_bb_sc" weight="1.3"/>
      <Reweight scoretype="hbond_sc" weight="1.3"/>
      <Reweight scoretype="rama" weight="0.2"/>
    </ScoreFunction>
  </SCOREFXNS>

  <LIGAND_AREAS>
    <LigandArea name="inhibitor_dock_sc" chain="X" cutoff="6.0" add_nbr_radius="false" all_atom_mode="true" minimize_ligand="10"/>
    <LigandArea name="inhibitor_final_sc" chain="X" cutoff="6.0" add_nbr_radius="false" all_atom_mode="true"/>
    <LigandArea name="inhibitor_final_bb" chain="X" cutoff="7.0" add_nbr_radius="false" all_atom_mode="true" Calpha_restraints="0.3"/>
  </LIGAND_AREAS>

  <INTERFACE_BUILDERS>
    <InterfaceBuilder name="side_chain_for_docking" ligand_areas="inhibitor_dock_sc"/>
    <InterfaceBuilder name="side_chain_for_final" ligand_areas="inhibitor_final_sc"/>
```

```
      <InterfaceBuilder name="backbone" ligand_areas="inhibitor_final_bb" extension_window="3"/>
    </INTERFACE_BUILDERS>

    <MOVEMAP_BUILDERS>
      <MoveMapBuilder name="docking" sc_interface="side_chain_for_docking" minimize_water="false"/>
      <MoveMapBuilder name="final" sc_interface="side_chain_for_final" bb_interface="backbone" minimize_water="false"/>
    </MOVEMAP_BUILDERS>

    <SCORINGGRIDS ligand_chain="X" width="34">
      <ClassicGrid grid_name="ClassicGrid" weight="1.0"/>
    </SCORINGGRIDS>

    <MOVERS>
      <Transform name="transform" chain="X" box_size="7.0" move_distance="0.2" angle="20" cycles="500" repeats="1" temperature="5"/>
      <HighResDocker name="high_res_docker" cycles="6" repack_every_Nth="3" scorefxn="ligand_soft_rep" movemap_builder="docking"/>
      <FinalMinimizer name="final" scorefxn="hard_rep" movemap_builder="final"/>
      <InterfaceScoreCalculator name="add_scores" chains="X" scorefxn="hard_rep" native="%%nativepdb%%"/>

      <MultiplePoseMover name="add_filter">
        <ROSETTASCRIPTS>
        <SCOREFXNS>
          <ScoreFunction name="hard_rep" weights="ligand">
            <Reweight scoretype="fa_intra_rep" weight="0.004"/>
            <Reweight scoretype="fa_elec" weight="0.42"/>
            <Reweight scoretype="hbond_bb_sc" weight="1.3"/>
```

```
          <Reweight scoretype="hbond_sc" weight="1.3"/>
          <Reweight scoretype="rama" weight="0.2"/>
        </ScoreFunction>
      </SCOREFXNS>

      <FILTERS>
        <LigInterfaceEnergy name="LigInterface"  scorefxn="hard_rep" confidence="0.0" />
        <DSasa name="DSASA" lower_threshold="0.0" upper_threshold="1.0" confidence="0.0" />
      </FILTERS>

      <PROTOCOLS>
        <Add filter_name="DSASA" />
        <Add filter_name="LigInterface" />
      </PROTOCOLS>
      </ROSETTASCRIPTS>
    </MultiplePoseMover>
  </MOVERS>

  <PROTOCOLS>
    <Add mover_name="transform"/>
    <Add mover_name="high_res_docker"/>
    <Add mover_name="final"/>
    <Add mover_name="add_scores"/>
    <Add mover_name="add_filter"/>
  </PROTOCOLS>

</ROSETTASCRIPTS>
```

Appendix S7. Example input to convert RosettaLigand silent file to pdb file.

```
#!/bin/bash

{ROSETTA_PATH}/main/source/bin/extract_pdbs.default.macosclangrelease \
    -database {ROSETTA_PATH}/main/database \
    -in:file:silent_struct_type binary \
    -ignore_unrecognized_res 1 \
    -in:file:fullatom \
    -missing_density_to_jump true \
    -extra_res {PARAMS_FILE} \
    -in:file:silent {SILENT_FILE} \
    -tags {POSE_TAG} \
    -mistakes:restore_pre_talaris_2013_behavior \
    -score:analytic_etable_evaluation true

```

Appendix S8. Example input for GALigandDock

```
#!/bin/bash

## Formatted to reflect example script from Park H. et al 2021. https://dx.doi.org/10.1021/acs.jctc.0c01184

if [ $# -lt 3 ]; then
```

```
	echo "USAGE: GADock.sh <NativePDB.pdb> <Test Protein Ligand Complex > <File handle for params>"
	exit
fi

mkdir scorefile
mkdir results_silent
rosetta_execute={ROSETTA_PATH}/main/source/bin/rosetta_scripts.static.linuxgccrelease
rosetta_database={ROSETTA_PATH}/main/database

## DO NOT EDIT BELOW THIS LINE ##
nativepdb=${1}
TestComplex=${2}
file_handle=${3}

$rosetta_execute \
    -in:path:database $rosetta_database \
    -s ./seed_files/${TestComplex} \
    -parser:protocol ./seed_files/GA-Dock-dockflex_blind.xml \
    -parser:script_vars initial_pool=./seed_files/${TestComplex} nativepdb=./seed_files/${nativepdb} \
    -extra_res_fa ./seed_files/${file_handle}.params \
    -gen_potential \
    -overwrite \
    -crystal_refine \
    -scale_rb 10.0 \
    -score::hb_don_strength hbdon_GENERIC_SC:1.45 \
    -score::hb_acc_strength hbacc_GENERIC_SP2SC:1.19 \
    -score::hb_acc_strength hbacc_GENERIC_SP3SC:1.19 \
    -score::hb_acc_strength hbacc_GENERIC_RINGSC:1.19 \
    -no_autogen_cart_improper \
```

```
     -out:file:silent_struct_type binary \
     -out:file:scorefile ./scorefile/GADock-sc_${file_handle}.sc \
     -out:file:silent ./results_silent/GADock-sc_${file_handle}_.silent \
     -mute core basic protocols.relax

```

Appendix S9. Example XML for GALigandDock

```
<ROSETTASCRIPTS>
      <SCOREFXNS>
        <ScoreFunction name="relaxscore" weights="beta_genpot_cart"/>
        <ScoreFunction name="dockscore" weights="beta_genpot">
              <Reweight scoretype="fa_rep" weight="0.2"/>
              <Reweight scoretype="coordinate_constraint" weight="0.1"/>
        </ScoreFunction>
      </SCOREFXNS>
      <RESIDUE_SELECTORS>
      </RESIDUE_SELECTORS>
      <TASKOPERATIONS>
      </TASKOPERATIONS>
      <MOVE_MAP_FACTORIES>
      </MOVE_MAP_FACTORIES>
      <SIMPLE_METRICS>
      </SIMPLE_METRICS>
      <FILTERS>
      </FILTERS>
      <MOVERS>
        <GALigandDock name="dock" runmode="dockflex" scorefxn="dockscore"
scorefxn_relax="relaxscore" grid_step="0.25" padding="7.0" hashsize="8.0" subhash="3"
```

```
final_exact_minimize="sc" random_oversample="10" rotprob="0.9" rotEcut="100"
sidechains="aniso" initial_pool="%%initial_pool%%" nativepdb="%%nativepdb%%" >
        </GALigandDock>
        <MultiplePoseMover name="add_filter">
          <ROSETTASCRIPTS>
            <SCOREFXNS>
              <ScoreFunction name="relaxscore" weights="beta_genpot_cart"/>
            </SCOREFXNS>
            <FILTERS>
              <LigInterfaceEnergy name="LigInterface"  scorefxn="relaxscore"
confidence="0.0" />
              <DSasa name="DSASA" lower_threshold="0.0" upper_threshold="1.0"
confidence="0.0" />
            </FILTERS>
            <PROTOCOLS>
              <Add filter_name="DSASA" />
              <Add filter_name="LigInterface" />
            </PROTOCOLS>
          </ROSETTASCRIPTS>
        </MultiplePoseMover>
      </MOVERS>
      <PROTOCOLS>
          <Add mover_name="dock" />
          <Add mover_name="add_filter" />
      </PROTOCOLS>
      <OUTPUT scorefxn="relaxscore" />
</ROSETTASCRIPTS>
```

Appendix S10. Example input to convert GALigandDock silent file to pdb file.

```
#!/bin/bash

{ROSETTA_PATH}/main/source/bin/extract_pdbs.default.macosclangrelease \
    -database {ROSETTA_PATH}/main/database \
    -in:file:silent_struct_type binary \
    -ignore_unrecognized_res 1 \
    -in:file:fullatom \
    -missing_density_to_jump true \
    -extra_res {PARAMS_FILE} \
    -in:file:silent {SILENT_FILE} \
    -tags {POSE_TAG} \
    -beta \
```
